# Is it feasible to conduct a randomised controlled trial of pretransplant exercise (prehabilitation) for patients with multiple myeloma awaiting autologous haematopoietic stem cell transplantation? Protocol for the PREeMPT study

**DOI:** 10.1136/bmjopen-2017-021333

**Published:** 2018-03-09

**Authors:** Carol Keen, Julie Skilbeck, Helen Ross, Lauren Smith, Karen Collins, Joanne Dixey, Stephen Walters, Diana M Greenfield, John A Snowden, Susan Mawson

**Affiliations:** 1 Acute Therapy Services, Sheffield Teaching Hospitals NHS Foundation Trust, Sheffield, UK; 2 Department of Nursing, Sheffield Hallam University, Sheffield, UK; 3 ScHARR, University of Sheffield, Sheffield, UK

**Keywords:** myeloma, bone marrow transplantation, rehabilitation medicine

## Abstract

**Introduction:**

While myeloma is an incurable malignancy, developments in disease management have led to increased life expectancy in recent years. Treatment typically involves stem-cell transplantation. Increased survival rates equate to more patients living with the burden of both the disease and its treatment for increasing number of years, rendering myeloma a long-term condition.

Evidence exists to demonstrate the benefits of exercise for patients recovering from stem-cell transplantation, and prehabilitation—exercise before treatment—has been shown to be effective in other disease areas. To date there has been no research into prehabilitation in patients with myeloma awaiting transplantation treatment.

Our objective is to determine whether it is feasible to conduct a randomised controlled trial into pretransplant exercise for patients with multiple myeloma who are awaiting autologous stem-cell transplantation.

**Methods and analysis:**

This mixed methods study identifies patients with diagnosis of multiple myeloma who have been assigned to the autologous transplantation list and invites them to participate in six weekly sessions of individualised, supervised exercise while awaiting transplantation.

Quantitative data to determine feasibility targets include rates of recruitment, adherence and adverse events, and outcome measures including 6 min walking distance test and quality of life.

Qualitative interviews are undertaken with a purposive sample of patients to capture their experiences of the study and the intervention.

**Ethics and dissemination:**

Ethics committee approval has been obtained. Dissemination will be through open-access publications and presentations and will seek to reach multiprofessional bases as well as patients and carer groups, addressing the widespread interest in this area of research.

**Trial registration number:**

NCT03135925; Pre-results.

**Strengths and limitations of this study**

- The sample size for the qualitative aspect of this study is likely to be small—it is intended to inform future study design rather than provide definitive understanding.
- For practical reasons and to encourage patient recruitment, time points for data collection are aligned with clinical interventions, rather than specifically for research purposes. They are therefore subject to variations, and not within the control of the study team.
- As a feasibility study, this will not provide evidence of the effectiveness of prehabilitation, but will inform future study design for evaluating effectiveness.

## Introduction

Myeloma is an incurable malignancy of antibody producing B lymphocytes and plasma cells. Equating to seven new cases per 100 000 population in the UK, it represents 10% of all new haematological cancers.^1^ Disease symptoms include anaemia and hypercalcaemia causing fatigue and weakness, immunosuppression and lytic lesions of bone increasing pathological fracture risk.^2^

Due to developments in disease management, life expectancy has increased significantly in the last 10 years.^3^ The 5-year relative survival rate for England was 42.2% in 2011,^4^ and is set to increase further due to earlier interventions in the disease process, more effective chemotherapies and increased use of autologous stem-cell transplantation.^5^

Following diagnosis of multiple myeloma, the standard treatment for younger patients (generally, but not exclusively, under the age of 70) with adequate fitness consists of an intensive pathway starting with induction treatment using a variety of regimens delivered as an outpatient or day case given to control disease until maximum response is achieved (usually reflected by a plateau in serum paraprotein).^6–8^ This response is then consolidated with autologous stem-cell transplantation, which permits the administration of high-dose myeloablative melphalan chemotherapy, a procedure typically requiring around 3 weeks inpatient care, after which patients take several months to make a functional recovery.^6–8^ The procedure is non-curative and relapse/progression of myeloma occurs after an average of 2–3 years, which requires reinstitution of induction treatment, and, in many patients, consolidation with a second autologous transplant procedure.^9 10^

### Rationale for the study

Increased survival rates equate to more patients living with the burden of both the disease and its treatment for increasing number of years, rendering myeloma a long-term condition.^11^ The cumulative effects of the disease, compounded with the debilitating toxic nature of the treatment, impact significantly the quality of life of patients beyond the end of treatment, with late-effects symptoms including infection, fatigue, metabolic, neurological and cardiovascular disorders, as well as pain, physical fitness and psychological concerns.^12^

Only 20% of patients with myeloma meet national physical activity guidelines post-treatment,^12^and activity declines through treatment due to perceived barriers to exercise including pain, fear of injury and fatigue.^13^ Although research evidence in physical activity has been demonstrated to be limited,^14^ evidence exists to demonstrate the benefits of exercise for patients recovering from stem-cell transplantation.^15^ Prehabilitation after treatment in patients with myeloma has been shown to improve symptoms of physical performance, muscle strength, aerobic capacity, psychological outcomes, immunological function and fatigue.^16^ Exercise training for myeloma survivors has been shown to be safe and feasible during treatment with high attendance and adherence^17^ and has been implemented widely in clinical practice.

Studies demonstrate that pretransplant patients have reduced exercise capacity and increased comorbidities compared with a normal population, yet most rehabilitative interventions occur during and after treatment.^15^ Thus, while exercise rehabilitation after treatment for myeloma can be effective, we must also consider rehabilitative interventions prior to the start of treatment: prehabilitation, defined as,

a process on the continuum of care that occurs between the time of cancer diagnosis and the beginning of acute treatment … provides targeted interventions that improve a patient’s health to reduce the incidence and the severity of current and future impairments.^18^

Examples of prehabilitation exist in other clinical specialties: it has been used for some time in orthopaedic surgery to improve outcomes and postoperative recovery,^19^ and its economic benefits have been demonstrated within colorectal surgery.^20^ A review of prehabilitation in patients with presurgical cancer demonstrated the effective use of aerobic interventions in the management of patients undergoing thoracic surgery for lung cancer, identified the potential for its use in other oncology settings and called for further research to evaluate prehabilitation for wider groups of patients with cancer.^19^

Guidelines for the management of late and long-term effects of myeloma recommend that regular physical activity, including prehabilitation and rehabilitation, and aspiration to a general healthy lifestyle, are integral to patient care pathways.^12^

Autologous stem-cell transplantation has become the most common treatment in myeloma, with, for example, over 1400 performed in the UK annually, and procedures are performed in what is normally considered an elderly patient population, many with comorbidities and frailty. It is an intensive toxic procedure, with a recovery period of at least 6 months and strategies to improve recovery are warranted, including prehabilitation. A window of opportunity—usually a period of 4–6 months exists to offer prehabilitation between diagnosis or relapse and the commencement of the autologous stem-cell transplantation process. Coleman *et al*^21^ studied 24 patients with multiple myeloma undergoing a home-based exercise programme during chemotherapy and stem-cell transplantation and identified that no patient injured themselves and that the intervention had positive effects on lean body weight, fatigue and sleep disturbance. Despite this, no evidence currently exists regarding the use of prehabilitation exercise interventions in multiple myeloma.

This article describes the protocol for a study under way investigating the feasibility of research into the provision of an exercise intervention in patients with myeloma who are due to receive autologous stem-cell transplantation.

## Aims and objectives

The aim of this study is to determine whether it is feasible to conduct a randomised controlled trial into pretransplant exercise for patients with multiple myeloma who are awaiting autologous stem-cell transplantation.

We will determine this through completion of the following objectives:

1. Assess the acceptability of the study to patients by measuring recruitment and retention to the study and through qualitative interview responses.
2. Explore reasons for non-consent to study participation.
3. Establish whether a target cohort of patients exists.
4. Determine the most appropriate recruitment points postdiagnosis through steering group feedback, recruitment rate when compared with numbers invited to join the study and qualitative interview reports.
5. Assess the suitability of inclusion and exclusion criteria by examining recruitment data.
6. Assess the acceptability of the intervention through qualitative interviews and retention rates during the study.
7. Determine duration of the intervention before transplantation starts by monitoring point of recruitment to the study and time to transplant.
8. Explore the appropriateness of outcome measures/completeness by qualitative interview responses, completion rates, time to complete.

## Methods and analysis

### Methodology

Mixed methods, combining qualitative and quantitative data collection and analysis, are used to achieve the described aims and objectives.

### Design

**Figure 1:**
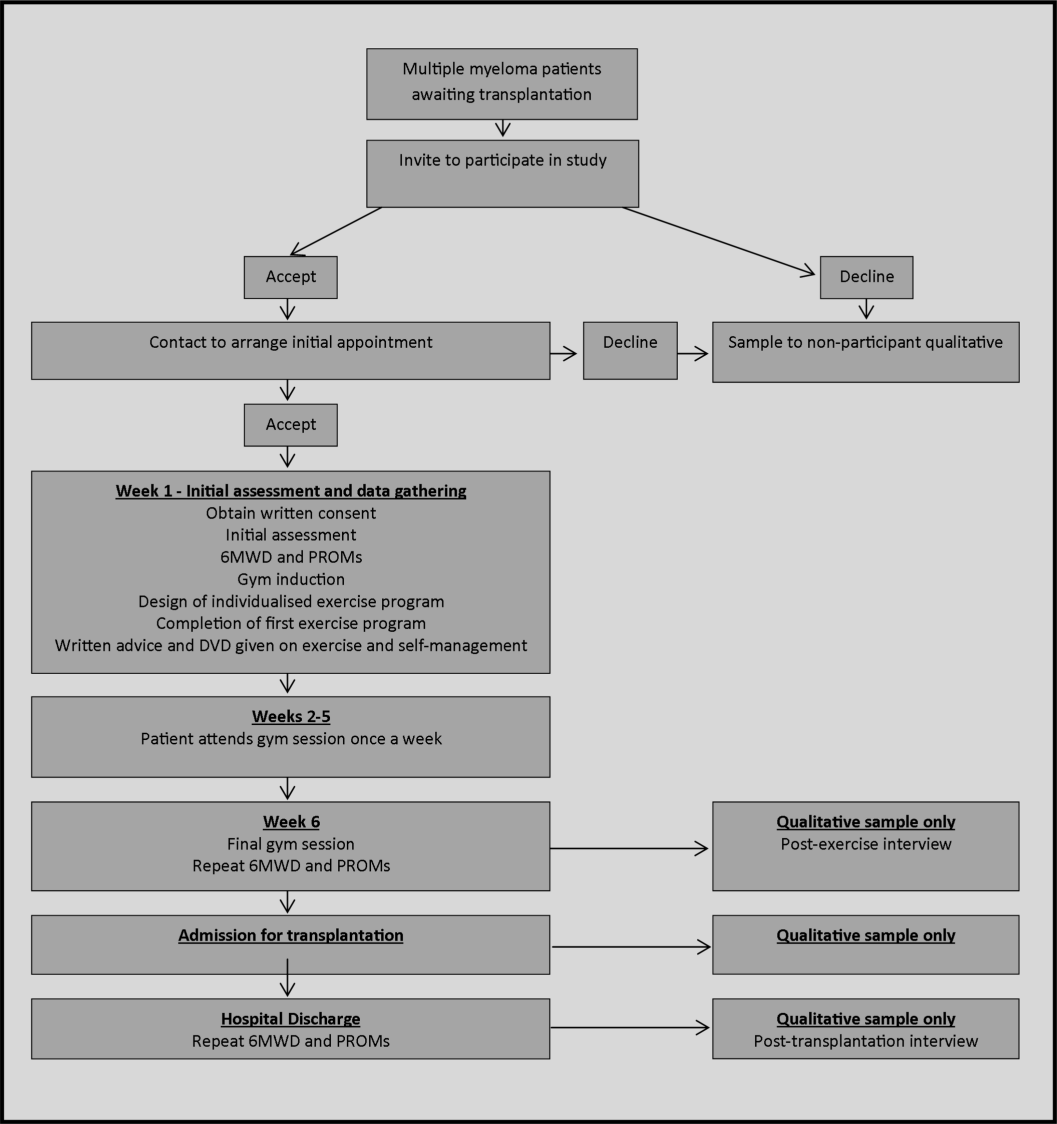
Recruitment and intervention flow chart. 6MWD, 6 min walking distance; DVD, digital versatile disc; PROMs, patient-reported outcome measures.

This is a prospective feasibility study (see figure 1 for study flow chart).

### Setting

Assessments and exercise sessions take place in the physiotherapy outpatient department in an acute hospital trust, which is a regional specialist centre for haematological services. Patient interviews take place in private rooms in the physiotherapy department or over the telephone for patient convenience.

### Feasibility

The feasibility of the intervention is determined through the following targets:

- Recruitment: based on patient numbers at the study site, the recruitment target is 24 patients in a 12-month period (ie, two patients per month).
- Attendance: minimum average attendance at exercise sessions of 66% of the scheduled/invited sessions.
- Retention: 80% patient retention to 6-week follow-up assessment.
- Adverse events: adverse events are closely monitored and used to inform decisions to proceed.

Acceptability of the intervention to patients is also determined through the qualitative data collection and analysis, described in a later section.

### Quantitative data collection and analysis

Data collection will take place between September 2016 and February 2018.

#### Sampling

Consecutive sampling is used to recruit patients to this study who have a diagnosis of multiple myeloma and have been assigned to the autologous transplantation list. The recruiting centre transplants approximately 70 patients with myeloma per year: sampling all patients over a 12-month period will indicate study recruitment feasibility. This feasibility study did not have a formal sample size calculation to determine a priori the number of participants to recruit; it aimed to recruit for a fixed period of time (12 months) at a single centre, and one of the outcomes was to estimate the recruitment rate per month.

#### Inclusion criteria

All patients with a diagnosis of multiple myeloma, assigned to the autologous transplantation waiting list for either a first or second transplant.^22^

#### Exclusion criteria

To allow safe completion of initial objective assessments, patients with a history of unstable angina or heart attack in the previous month are excluded.^23^ Medical stability is a prerequisite for transplantation, therefore no patients are excluded on this basis.

#### Recruitment

Patients are screened at clinic appointments by the bone marrow transplant team during their preparation for transplant. Patients meeting the inclusion criteria are provided with verbal and written information and invited to be involved in the study. Follow-up takes place after 48 hours via a phone call from a study physiotherapist: any remaining questions are discussed, and if the patient agrees to take part then written consent is obtained and an initial assessment appointment is made.

Patients who choose not to join the study are invited to take part in a qualitative interview to explore their reasoning (figure 1). This is described in more detail under Qualitative data collection and analysis.

#### Intervention

##### Initial assessment

Patients attend an initial assessment with a study physiotherapist who undertakes the following:

- explanation of the prehabilitation programme
- documentation of written consent
- subjective history including comorbidities and patient goals
- induction to the gym area equipment
- provision of booklet and digital versatile disc with physical activity advice
- baseline objective assessment (table 1)
- design of individualised gym programme in line with patient abilities and goals
- completion of an initial gym circuit with close supervision.

##### Weeks 2–5

Patients attend weekly 1-hour physiotherapist-led group gym sessions and complete their individualised programmes. Supervision is available as required and programmes are progressed in line with patient ability and performance.

##### Week 6

Completion of final gym circuit and repeat of objective assessments (table 1).

##### Follow-up

Patients are followed up on admission for transplant, and again on transplant discharge, for further repeat of objective assessments (table 1).

**Table 1:** Study data collection

	Recruitment	Initial assessment	Weeks 2–5	Week 6	Transplant admission	Transplant discharge
Screening data	✓					
Demographic data		✓				
6 min walking distance test		✓		✓	✓	✓
PROMs		✓		✓	✓	✓
Activity data		✓	✓	✓	✓	✓
Adverse events		✓	✓	✓		

PROMs, patient-reported outcome measures.

#### Outcome measures

The following data are captured for study participants.

##### Screening data

Through initial screening and recruitment, data are collected on:

- number of patients meeting inclusion criteria
- patients accepting initial study information
- patients agreeing to attend for initial assessment
- reasons for non-participation.

##### Demographic data

The following demographic data are captured during the initial assessment:

- gender
- length of diagnosis
- baseline physical activity levels
- transplant history
- pretransplant therapies received
- time to transplantation from decision to transplant
- other relevant information.

##### Functional measure

Patients undertake a 6 min walking distance (6MWD) test before and after the exercise intervention. The 6MWD test is a useful field test of functional capacity, is safe to administer and although it has less correlation with peak oxygen capacity than the shuttle walk test, it is better tolerated by patients and is more reflective of activities of daily living as it is a submaximal exercise test.^23^ The 6MWD test has been found to be a valid and reliable test in patients with cancer.^24^

##### Patient reported outcome measures

As this is a feasibility study, it is useful to determine the feasibility and acceptability of outcomes to be used. For this reason, two different sets of patient reported outcome measures (PROMs) are issued to alternate patients taking part in the study (table 2). The data collected in the outcome measures and in the qualitative interviews will determine their value in any future studies.

**Table 2:** Patient reported outcome measures

Group	Category	Measure
Physical activity/fitness	Group 1	International Physical Activity Questionnaire^25^
	Group 2	Godin Leisure Time^26^
Mental well-being	Groups 1 and 2	Warwick and Edinburgh Mental Well-being Scale^27^
Quality of Life	Group 1	Functional Assessment of Cancer Therapy - Multiple Myeloma (FACT-MM)^28^
	Group 2	European Organisation of Cancer Treatment Quality of Life Questionnaire (EORTC QLQ)^29^
Self-efficacy for exercise	Groups 1 and 2	Self-Efficacy for Exercise Scale^30^

##### Activity data

The following activity data are collected for each participant:

- the number of gym attendances
- follow-up compliance
- withdrawals from the study and at which stage of the study these occur
- reasons for withdrawal or non-attendance.

##### Data collection

Table 1 shows the full data collection schedule for the study.

#### Data analysis

Flow of participants through the study is captured, and the baseline clinical and demographic characteristics of consented participants assessed with appropriate summary statistics.

The data analysis for the feasibility objectives uses descriptive statistics and focuses on CI estimation.

1. The feasibility of recruitment to main trial is assessed with the consent rate (defined as the ratio of number of consented participants/number of eligible participants) and its associated 95% CI, and the recruitment rate per month and its associated 95% CI. The target recruitment rate is a minimum of two participants per month.
2. Reporting of the number and characteristics of eligible patients approached for the study and reasons for refused consent
3. Reporting of study participant retention rates at 6-week follow-up (eg, participants with a valid 6MWD outcome— the probable primary outcome for the main trial) and its associated 95% CI. The target is a minimum of 80% retention to 6-week follow-up assessment.
4. Reporting of the number (and rate) of serious adverse events/incidents (and its associated 95% CI) experienced by the participants in the pretransplantation period. A serious adverse event (SAE) is defined as any adverse event or adverse reaction that results in death, is life-threatening, requires hospitalisation or prolongation of existing hospitalisation, results in persistent or significant disability or incapacity, or is a congenital anomaly or birth defect.
5. Reporting of the decision on primary endpoint for any main trial (current estimate suggests 80% power, two-sided, with n=610 to detect 5% (18 metre) difference in 6MWD test with 10% dropout at 12 metre).

### Qualitative data collection and analysis

#### Sampling and data collection

The aim of the qualitative data collection and analysis is to explore in greater detail patients’ perceptions of the study including its acceptability, as well as barriers and facilitators to participation.

Patients who decline to take part in the exercise trial are asked if they would undertake a short telephone interview to ascertain their reasons for not taking part in the study. Participants who have already consented to take part in the trial and are undertaking the exercise programme are approached by a member of the clinical team and asked if they would be interested in taking part in a series of face-to-face or telephone interviews (figure 1).

The interview topic guide is informed by evidence regarding acceptability and barriers and facilitators to participation from previous studies in prehabilitation and studies of exercise in patients with multiple myeloma.^17 21^ It is also tailored to match developments and areas of interest that emerged from the quantitative data collection as the study progresses. The topic guide is flexible to enable exploration of individual experiences, for example, those who had fully completed the intervention compared with those who may have had only limited participation.

Topic areas include reasons for non-participation, participants' characteristics and descriptive information regarding the nature of their disease management to date; the patient experience of the intervention, with reference to aspects that may impact the design of future study, for example, recruitment, ease or difficulty of attendance, timing and nature of data collection, suitability of outcome measures; barriers and enablers to participation in the study.

### Qualitative analysis

The framework approach is used to analyse the qualitative data.^25^ This method is appropriate for identifying, analysing, and reporting themes and patterns within data. It is a flexible and useful research tool, which can potentially provide a rich and detailed, yet simple account of data. Early on in the analysis the transcripts are repeatedly read to develop an understanding of the breadth and depth of the data. During this process, data are labelled and coded in an iterative process whereby patterns and sequences of content over time are identified within and across all the participants. Emergent themes are further developed and refined by analysing similarities and divergences between and within the participants, to form a coherent pattern.^26^

## Ethics and dissemination

### Ethical consideration

Ethical issues relating to informed consent and confidentiality are addressed throughout. It is acknowledged that patients approached and participating in this study may be physically debilitated and experiencing anxiety, having received a new cancer diagnosis and awaiting a challenging programme of treatment. Due care and diligence are taken when consenting potential subjects and the option to withdraw from the study at any point is reiterated. In particular, the nature of qualitative interviews, focusing on personal experiences of illness and treatment, may result in some distress to some participants. The researchers have relevant experience in working with patients with life-threatening illness and are skilled at talking to them, as well as being able to recognise patient distress.

### Dissemination

This study has involvement from, and relevance to, the professions of physiotherapy, medicine and nursing. Dissemination will incorporate each of these professions and reach into the wider healthcare community. We will seek to share the findings of the study through local, national and international channels.

Patient involvement in the project has been through representation in study design and on the project steering group from the North Trent Cancer Research Network Consumer Research Panel. We will liaise with this group to invite ideas regarding dissemination to study participants, patients and carers.

Where the findings of the study have implications for the provision of new or existing services to patients with myeloma, we will ensure dissemination to relevant key opinion leaders and stakeholders to support decision making.

## Discussion

It is anticipated that this study will demonstrate the feasibility of conducting research into prehabilitation physical activity programmes. Factors likely to affect feasibility may include patient perception of the role of physical activity; patient time commitments; patient wellness to take part and patient enjoyment of exercise.

If feasibility is confirmed then we will seek to establish a larger scale study to test the efficacy of the intervention. The findings from this study will be used to support power and sample size calculations and to establish suitable outcome measures for future studies.

If the feasibility criteria are not satisfied then there will be lessons to learn regarding the potential for future studies in the field, or modifications to the intervention or study design if further study is indicated. Since prehabilitation is an area of growing interest in other clinical areas, including other cancer and non-cancer pathologies, then it is anticipated that the findings of this study will also be of interest to practitioners considering prehabilitation outside of myeloma.

Establishing the feasibility of research in this field is important to explore the case for prehabilitation. The effects of bone marrow transplantation can have a high cost to the individual and to health services. There is clearly of value in exploring treatment options that may lessen the effects of treatment, particularly those with relatively low associated costs such as exercise prehabilitation.

## Supplementary Material

Reviewer comments

Author's manuscript
