# Supplementary material for: Is it feasible to conduct a randomised controlled trial of pretransplant exercise (prehabilitation) for patients with multiple myeloma awaiting autologous haematopoietic stem cell transplantation? Protocol for the PREeMPT study
Source: BMJ Open. 2018 Mar 9;8(3):e021333. doi: 10.1136/bmjopen-2017-021333 (PMC5855173; doi:10.1136/bmjopen-2017-021333)
Supplement: Author's manuscript [file bmjopen-2017-021333.draft_revisions.pdf]

BMJ Open

BMJ Open is committed to open peer review. As part of this commitment we make the peer review history of every article we publish publicly available.

When an article is published we post the peer reviewers' comments and the authors' responses online. We also post the versions of the paper that were used during peer review. These are the versions that the peer review comments apply to.

The versions of the paper that follow are the versions that were submitted during the peer review process. They are not the versions of record or the final published versions. They should not be cited or distributed as the published version of this manuscript.

BMJ Open is an open access journal and the full, final, typeset and author-corrected version of record of the manuscript is available on our site with no access controls, subscription charges or pay-per-view fees (http://bmjopen.bmj.com).

If you have any questions on BMJ Open's open peer review process please email info.bmjopen@bmj.com

BMJ Open

Pathways and cost-effectiveness of routine lung cancer inpatient care in rural Anhui, China: a retrospective cohort study protocol

Journal:	*BMJ Open*
Manuscript ID	bmjopen-2017-018519
Article Type:	Protocol
Date Submitted by the Author:	05-Jul-2017
Complete List of Authors:	Shen, XingRong; Anhui Medical University Shcool of Health Service Management<br>Diao, MengJie; Anhui Medical University School of Health Service Management<br>Feng, Rui; Anhui Medical University, Library Department of Literature Retrieval and Analysis<br>Lu, ManMan ; Anhui Medical University School of Health Service Management<br>Zhang, PanPan ; Anhui Medical University School of Health Service Management<br>Jiang, Tao ; Anhui Medical University School of Health Service Management<br>Wang, DeBin; Anhui Medical University, School of Health Services Management
Keywords:	cost effectiveness, lung cancer, inpatient care, retrospective study, China

SCHOLARONE™
Manuscripts

For peer review only

Pathways and cost-effectiveness of routine lung cancer inpatient care in rural Anhui, China: a retrospective cohort study protocol

XingRong Shen*, MengJie Diao*, Rui Feng, ManMan Lu, PanPan Zhang, Tao Jiang, Debin Wang[#]

Corresponding author: Debing Wang.

Post address: 81 Meishan Road, Hefei, Anhui, China. 230032.

Email: dbwang@vip.sina.com

Telephone: (0086) 5116395

XingRong Shen:

School of Health Service Management, Anhui Medical University, Hefei, Anhui, China.

MengJie Diao:

School of Health Service Management, Anhui Medical University, Hefei, Anhui, China.

Rui Feng:

Department of Literature Review and Analysis, Library of Anhui Medical University, Hefei, Anhui, China.

ManMan Lu:

School of Health Service Management, Anhui Medical University, Hefei, Anhui, China.

PanPan Zhang:

School of Health Service Management, Anhui Medical University, Hefei, Anhui, China.

Tao Jiang:

School of Health Service Management, Anhui Medical University, Hefei, Anhui, China.

Word count：2791

For peer review only

ABSTRACT

Introduction: Routine inpatient care (RIC) for cancer patients forms various pathways of clinical procedures. Although most of the individual procedures comprising the pathways have been tested via clinical trials, little is known about the collective cost-effectiveness of the pathways as a whole. This study aims at identifying pathways of RIC procedures for lung cancer patients from rural Anhui, China and examining determinants of the pathways and their links to cost-effectiveness.

Methods and analysis: The study adopts a retrospective cohort study design and proceeds in 5 steps. Step 1 defines 4 main categories of study variables including clinical procedures, direct cost and effectiveness of procedures, and factors affecting use of these procedures and their cost and effectiveness. Step 2 selects a cohort of 5000 lung cancer patients diagnosed between July 1, 2014 and June 30, 2015 from rural Anhui by clustered-random sampling. Step 3 retrieves the records of all the inpatient care episodes due to the lung cancer and extracts data about RIC procedures, proximate patient outcomes (e.g., Karnofsky performance status, lung function score) and related factors (e.g., stage of cancer, age, gender) by 2 independent clinician researchers using a pre-developed worksheet. Step 4 estimates the direct cost of each of the RIC procedures using micro-costing and collects data about ultimate patient outcomes (survival and progression-free survival) through a follow up survey of patients and/or their close relatives. Step 5 analyzes data collected and explores pathways of RIC procedures and their relations with patient outcomes, costs, cost-effect ratios and a whole range of clinical and socio-demographic factors using multivariate regression and path models.

Ethics and dissemination: The study protocol has been approved by authorized ethics committee. Findings from the study will be disseminated through conventional academic routes such as peer-reviewed publications and presentations and regional, national and international conferences.

Trial registry

ISRCTN25595562

Key words: cost effectiveness, lung cancer, inpatient care, retrospective study, China

For peer review only

Strengths and limitations of this study

- The study adopts a retrospective cohort study design involving a large representative sample of community patients;
- It evaluates cost-effectiveness of pathways of clinical procedures as a whole rather than individual procedures;
- It examines pathways of routine inpatient care for a huge but understudied Chines rural population;
- It extracts data from routine records kept at different hospitals and thus suffers from discrepancies in performances and data qualities.

Introduction

Lung cancer has been the most common cancer in the world for several decades.[1] Estimated new cases of the disease was 1.8 million in 2012 (12.9% of the total), 58% of which occurred in less developed regions. Lung cancer was also the most common cause of death from cancer worldwide, being responsible for nearly one in five (1.59 million in absolute number) of the total. [2] In China, lung cancer incidence shows a slight decreasing trend in the past few years, particularly for males. However, it is still the top first cancer for males and second for females, accounting for 25.2% of all new cancer cases and 29.5% of all cancer deaths in 2012. [3]

Routine inpatient care (RIC) for lung cancer consists of a combination of procedures. Patients with possible lung cancer need a detailed history and physical examination first. Then they should undergo posterior-anterior and lateral chest radiographs as well as CT scans of the chest and abdomen. In order to further confirm and determine stage and histology of the lesion, other diagnostic methods needed include whole-body fluoro-deoxy-glucose positron emission tomography, endoscopic ultrasound, sputum cytology, fine-needle aspiration, bronchoscopy and others. Following diagnosis of lung cancer, the patients proceed with combined-modality therapies depending on stage of the disease and co-morbidity and complications. Historically, surgery provides the best chance for cure for patients whose lung cancers are limited to the hemithorax and can be totally encompassed by excision.[4 5]And surgery has been generally used in combination with external-beam radiotherapy for control of the primary tumor and regional lymphatics.[6] In addition, chemotherapy has also been advocated as an integral part of combined modality approaches to earlier stages of disease. [7 8] For unselected advanced none-small cell lung cancer, platinum-based combinations have become the standard of care; while cisplatin– or carboplatin-based doublets are standard for patients with stage IV disease.[9 10] More recently, EGFR tyrosine kinase inhibitors have been introduced in second- and third-line treatment of advanced disease and in first-line treatment for selected patients.[11]

Given the complex procedures, ensuring quality RIC for lung cancer patients has been most challenging and guidelines are widely used in addressing this challenge. Numerous

For peer review only - http://bmjopen.bmj.com/site/about/guidelines.xhtml

studies have documented positive relations between compliance with guidelines and patients outcomes.[12 13] However, researchers also have raised concerns about guidelines. One of such concerns refers to lack of adequate consideration of costs. Most clinical procedures not only affect disease outcomes but also incur considerable costs.[14 15] Yet guidelines are based on trials focused primarily on effectiveness (e.g., survival) with little attention being paid to economic consequences.[16] Another concern relates to incompatible population between clinical trials and RIC. Clinical trials on which guidelines are based use highly selected populations; while RIC serves a general lung cancer population with different age, performance status and comorbidities.[17 18] A third concern revolves uncertain interactions between procedures. Although most individual guideline recommended procedures (GRPs) have established evidences, they are not used in isolation but in conjunction with others forming various clinical pathways. Efforts systematically assessing and comparing these pathways are scarce.[19-22] A fourth concern originates from varied compliance with guidelines since RIC often deviates substantially from guidelines.[23 24] The cost-effectiveness of these "substandard" pathways or mixed combinations of procedures (partly from guidelines, partly from experiences of individual clinicians) falls far from well-understood.[25] These all points to a conclusion that guidelines may not necessarily secure expected outcomes and there is a clear need for monitoring RIC.

All the above mentioned concerns surrounding cancer care are most pertinent to China. First, China has a unique "dual" medical care system in which patients often receive western medical medicine and traditional Chinese medicine simultaneously or in turn.[26] Second, China lacks coordinated referral and follow up mechanisms and cancer patients often moves freely from one hospital to another for different rounds of inpatient care.[27] This makes it hard for clinicians in leveraging different inpatient care episodes at different time points and hospitals into continuous and synergetic service. Third, China has strong socio-cultural norms and financial incentives that hinder cost control and guideline compliance.[28]

Study aims

This study aims at identifying pathways of RIC procedures for lung cancer patients from rural Anhui, China and examining determinants of the pathways and their links to cost-effectiveness.

Methodology

Guiding framework

The study uses a retrospective cohort design. Content of the study is defined using a practical framework as depicted by Figure 1. The framework holds that: a) patient outcomes and costs jointly define the ultimate goal, cost-effectiveness, of RIC; b) clinical procedures affect final patient outcomes indirectly via modifying psycho-physio-

For peer review only

pathological factors of patient outcomes and incur costs simultaneously; c) decision-making determines selection of RIC procedures based on understanding and prediction of the status of all the other elements included in the framework. By excluding the two brown circles, Figure 1 becomes an outcome-oriented framework that represents typical current RIC for cancer patients. Given that all clinical procedures inevitably incur more or less cost which in turn directly or/and indirectly affects selection and implementation of clinical procedures, cost-effectiveness oriented approaches are more relevant than outcomes-focused ones.[29]

Identification of procedures

The study uses a self-designed data extraction form in identifying major clinical procedures described in any RIC record under concern. The form lists all major RIC procedures under two main domains, i.e., diagnostic procedures (e.g., chest X-ray, chest CT, neck ultrasonography; Part D of supplementary file 1) and treatment procedures (e.g., surgical therapy, chemotherapy, psycho-behavioral intervention; Part E of supplementary file 1).

Estimation of costs

The study estimates overall and categorical costs (direct costs only) for each of the RIC procedures (e.g., lung function examination, computed tomography, white blood cell count) identified above using micro-costing techniques.[30] Taking the example of lung function examination, categorical costs include costs on personnel, equipment, materials, regents and others need in completing the examination; while overall cost of the procedure equals the sum of all these categorical costs. In addition, the study also calculates overall cost on individual inpatient by adding up the overall costs on all the clinical procedures he/she has received.

Measurement of effectiveness

The study uses both proximate outcome (PO) and ultimate outcome (UO) measures of effectiveness of RIC procedures. The UO indicators derive from a follow up survey about 2 years and half after the first hospitalization and include survival and progression-free survival (PFS). The PO measures come from RIC records and include Eastern Cooperative Oncology Group (ECOG), Karnofsky performance status (KPS) and compiled scores of: a) symptoms (e.g., chronic cough, chest pain, wasting syndrome); b) lung functions (e.g., forced vital capacity, forced one second expiratory volume), c) image findings (e.g., number of nodules identified in the lung, size of the largest nodules, presence of pleura or pericardial effusion); d) biological test findings (e.g., value of CEA, CA125, proGRP); and e) complications and comorbidities (e.g., presence of superior vena cava syndrome, superior vena cava syndrome). Each of these domain specific PO scores equals weighted sum of all sub-indicators within the domain. For example, the compiled score of "lung functions" equals the sum of weighted values of forced vital capacity, forced one second expiratory volume etc. Here the weights come from the

coefficients of multivariate regression modeling using an UO indicator (e.g., survival) as the dependent variable; while forced vital capacity, forced one second expiratory volume etc. as the independent variables; and stage of disease, age, gender and others as the confounding variables.

Calculation of cost-effectiveness

The study adopts cost-effectiveness ratios (CERs) and incremental cost-effectiveness ratio (ICERs) as the main indicator for measuring cost-effectiveness. Here ICER is defined by the difference in cost between two possible set of RIC procedures, divided by the difference in their effect. More specifically, $\mathrm{ICER} = (\mathrm{C}_1 - \mathrm{C}_0)/(\mathrm{E}_1 - \mathrm{E}_0)$, where C_1 and E_1 is the cost and effect in the study group and C_0 and E_0, the cost and effect in the reference group.[31] ICER represents the average incremental cost associated with 1 additional unit of the measure of effect. It serves a useful rule in resource allocation or clinical decision-making.[32]

Identification of influencing factors

The study also extracts, from RIC records, data about patient factors commonly believed to be linked with disease progression, treatment response and outcomes and utilization of RIC procedures. These include socio-demographics (e.g., age, gender, body height and weight, education, employment, marital status, medical insurance), risk behaviors and histories (e.g., smoking, alcohol drinking, history of cancer among family members), and clinical characteristics (e.g., stage of disease, historical findings, biomarkers).

Selection of participants

The study is implemented in Anhui, an inland province located in middle and east China. It has a population of 61.4 million and its per capita GDP and income rank in the middle (14th) among all provinces in the nation.[33 34] The social, cultural and economic background of Anhui is representative of over 80% of the whole population in China.[33 34] The province has 68 rural counties and each of them divides into 10 to 20 townships. Selection of participating counties, townships, patients and RIC case records uses a clustered random sampling which proceeds in 5 steps. Step 1 classifies all the counties in Anhui into southern, northern and middle areas. Step 2 randomly selects 3 counties from each of these areas (12 counties in total). Step 3 randomly draws 4 townships from each of the counties selected (48 townships in total). Step 4 searches the provincial reimbursement database of the New Rural Medical System (NRMS) and identifies all the patients within the selected townships who had been first diagnosed with primary lung cancer during July 1, 2014 and June 30, 2015. Step 5 searches the database again for all episodes of hospitalization due to the lung cancer for the patients identified in step 4. NRMS covers 98% of the rural residents and the estimated number of patients and admission episodes is about 5,000 and 25,000 respectively.

For peer review only

Data collection

The study obtains data through follow-up survey and data extraction. The follow-up survey applies to all the lung cancer patients identified above. It solicits information about the patient's: a) disease progression (i.e., died, alive with or without progression); b) if died, date of death; c) additional admissions duo to the lung cancer not included in the above mentioned NRMS database. The survey uses a short structured questionnaire (supplementary file 2). Administration of the questionnaire starts with a telephone interview (of the patient under concern or his/her close relatives for up to 5 time attempts) followed by a face-to-face interview (of the same respondents for up to 2 attempts) if the telephone contacts failed. The data extraction applies to records of all the hospital admission episodes identified via the NRMS database and the follow up survey. It uses a structured form (supplementary file 1) and extracts data about the clinical procedures, costs, effectiveness and influencing factors described above. Two experienced clinicians on care of lung cancer perform the data extraction. They visit (on one-by-one base) all the relevant hospitals, ask for permission to examine the full records and fill the worksheet independently first followed by discussions, if applicable, to solve discrepancies.

Data analysis

The data collected above allow a variety of descriptive and multivariate analysis. In particular, the data analysis centers on effectives, costs and pathway-based cost-effectives of RIC. Effectiveness analysis comprises mainly: a) description of UO indicators (e.g., survival rate) at different time points after first diagnosis by disease stage, age range etc. (Figure 2); b) multivariate regression models using UO indicators as dependent and socio-demographics, disease stage, selected RIC procedures and others as independent variables; c) path models using similar independent variables in b as exogenous, PO indices as direct endogenous, and UP indicators as indirect endogenous variables.

Similarly, cost analysis includes mainly: a) description of overall and categorical costs on different rounds of hospitalization by socio-demographic and selected clinical conditions (Figure 3); b) scatter plot of RIC procedures using the occurrence rate and unit cost of individual procedures as the coordinates; and c) multivariate models of overall and selected categorical costs.

Pathway-based cost-effectiveness analysis focuses primarily on constructing a pathway tree showing different combinations of RIC procedures starting from the first to the last episode of inpatient care and estimated cost-effectives ratios (CERs/ICERs) for each branches of the tree (Figure 4). It also performs multivariate regression analysis exploring potential factors affecting the flow of RIC among different branches.

For peer review only

Ethics and dissemination

The study involves retrieving RIC records and recruiting patients or their relatives. So it adheres to rigorous human subject protection principles. The study protocol had been reviewed and approved by the Biomedical Ethics Committee of Anhui Medical University (reference number: 20170312). Participation of hospitals, patients and their relatives are voluntary and written informed consent is sought from all participants. Findings from the study will be disseminated through conventional academic routes such as peer-reviewed publications and presentations and regional, national and international conferences.

Discussion

This study addresses RIC for lung cancer at hospitals in China from a range of meaningful perspectives. The study reinforces the concepts introduced in the landmark studies of Fisher et al and Wennberg et al, which convincingly demonstrated that high quality was not necessarily associated with high cost.[35] Describing inpatient lung cancer care in a view that its value is directly proportional to outcomes and inversely proportional to costs helps in guiding quality improvement by either better outcomes and/or lower costs. [36] The study calculates and compares the collective cost-effectiveness of different RIC pathways as a whole and thus informs coordinated inpatient care episodes and procedures at different time points and hospitals. The study enables ICERs estimation for specific guideline recommended procedures (GRPs) using various combinations of real and uncontrollable RIC procedures as the reference and thus enhances understanding and application of GRPs established through well-controlled studies in routine practice contexts.

Perhaps the most noteworthy findings of the current study may be the description of the pathways of RIC procedures and their links with cost-effectiveness (Figure 4). These pathways will provide easily understandable means for estimating and identifying, among others, the following: a) which pathways or combinations of procedures happen most or least in routine practice during different rounds of hospitalization for inpatients suffering from lung cancer in rural China; b) which pathways (from the first to last round of hospitalization) incur the highest or lowest direct costs; c) which pathways result in the best or worst patient outcome in terms of different PO and UO measures; d) which pathways are most or least cost-effective in terms of e.g., per unit cost gains in PFS, KPS, symptoms, lung functions, image findings, biological test findings, complications and comorbidities. These have important implications for clinical decision-making as well as policy-making.

Another point worth mentioning in particular refers to the links between the domain specific proximate outcome (PO) indices to key ultimate outcome (UO) indicators (e.g., survival) generated via a large scale (involving 5000 lung cancer patients) retrospective

For peer review only - http://bmjopen.bmj.com/site/about/guidelines.xhtml

cohort study. They provide useful references for clinicians on care of lung cancer patients in selecting appropriate procedures to achieve optimal collective contributions to UO.[37] At present, although PO indicators are routinely observed, they are presented to clinicians as individual indicators rather than compiled indices. And given the large number of PO indicators involved and the complex relations between RIC procedures and PO indicators and then UO indicators, it is difficult for practicing clinicians to make balanced decisions upon their personal experiences.[38]

The study also has limitations. First, different hospitals use different equipment, reagents and medicines. Their quality of case records may also vary substantially. These raise compatibility concerns in pooling data from different hospitals together and performing aggregate analysis. Second, the study considers only inpatient care; while patients may use various self-treatment and outpatient treatment in addition to inpatient care.[39 40] And inpatient and non-inpatient treatment may substitute each other to some extent. These may result in under-estimation of the effectiveness of RIC procedures. Third, more server or complicated cases of lung cancer patients may be more likely to use inpatient care. This may again lead to false reduced efficacy of inpatient care. Fourth, study uses only direct costs rather than full costs taking both direct and indirect costs into consideration.

Competing interests

The authors declare no competing interests.

Authors' contributions

XS and MD contributed equally in conceiving this project, facilitating protocol and instrument development, and drafting this manuscript. RF, ML, PZ and TJ are kore researchers for cost estimation, record extraction, follow up survey and data analysis respectively. DW provided expertise for overall design of the study, and revised and finalized the manuscript. All authors have read and approved the final submission.

Acknowledgements

Development of the primitive protocol was supported by the Natural Science Foundation of China (grant number: 81172201). Refinement and implementation of the protocol is lead and supported by Collaboration Center for Cancer Control of Anhui Medical University, Anhui and Luan Center for Diseases Control and Prevention.

References

1. McErlean A, Ginsberg MS. Epidemiology of lung cancer. *Semin Roentgenol* 2011;46 (3):173-7.

For peer review only

2. World Health Organization. GLOBOCAN 2012: Estimated Cancer Incidence, Mortality and Prevalence Worldwide in 2012. http://globocan.iarc.fr/Default.aspx.
3. Kong J, Xu F, He M, Chen K, et al. The incidence of lung cancer by histological type: a population-based study in Tianjin, China during 1981-2005.*Respirology* 2014;19(8):1222-8.
4. Woodard GA, Jablons DM. The Latest in Surgical Management of Stage IIIA Non-Small Cell Lung Cancer: Video-Assisted Thoracic Surgery and Tumor Molecular Profiling. *Am Soc Clin Oncol Educ Book* 2015;35:e435-41.
5. Grunenwald DH. The role of surgery in non-small-cell lung cancers. *Ann Oncol 2005*;16 Suppl 2:ii220-2.
6. Ricardi U, Badellino S, Filippi AR. Stereotactic radiotherapy for early stage non-small cell lung cancer. *Radiat Oncol J*. 2015;33(2):57-65.
7. Mangal S, Gao W, Li T1, Zhou QT. Pulmonary delivery of nanoparticle chemotherapy for the treatment of lung cancers: challenges and opportunities. *Acta Pharmacol Sin*. 2017. doi: 10.1038/aps.2017.34.
8. Khan I, Morris S, Hackshaw A, et al. Cost-effectiveness of first-line erlotinib in patients with advanced non-small-cell lung cancer unsuitable for chemotherapy. *BMJ Open* 2015; 5(7):e006733.
9. Garon EB, Ciuleanu TE, Arrieta O, et al. Ramucirumab plus docetaxel versus placebo plus docetaxel for second-line treatment of stage IV non-small-cell lung cancer after disease progression on platinum-based therapy (REVEL): a multicentre, double-blind, randomised phase 3 trial. *Lancet* 2014;384(9944):665-73.
10. Spigel DR, Luft A, Depenbrock H, et al. An Open-Label, Randomized, Controlled Phase II Study of Paclitaxel-Carboplatin Chemotherapy With Necitumumab Versus Paclitaxel-Carboplatin Alone in First-Line Treatment of Patients With Stage IV Squamous Non-Small-Cell Lung Cancer. *Clin Lung Cancer* 2017; pii: S1525-7304(17)30045-1.
11. Stinchcombe TE. The Use of EGFR Tyrosine Kinase Inhibitors in EGFR Wild-Type Non-Small-Cell Lung Cancer. *Curr Treat Options Oncol* 2016; 17(4):18.
12. Ahmed HZ, Liu Y, O'Connell K, et al. Guideline-concordant Care Improves Overall Survival for Locally Advanced Non-Small-cell Lung Carcinoma Patients: A National Cancer Database Analysis. *Clin Lung Cancer* 2017; pii: S1525-7304(17)30114-6.
13. Nadpara P, Madhavan SS, Tworek C. Guideline-concordant timely lung cancer care and prognosis among elderly patients in the United States: A population-based study. *Cancer Epidemiol*; 39(6):1136-44.
14. Hinde S, McKenna C, Whyte S, et al. Modelling the cost-effectiveness of public awareness campaigns for the early detection of non-small-cell lung cancer. *Br J Cancer* 2015; 113(1):135-41.
15. Kumar G, Woods B, Hess LM, et al. Cost-effectiveness of first-line induction and maintenance treatment sequences in non-squamous non-small cell lung cancer (NSCLC) in the U.S. *Lung Cancer* 2015. pii: S0169-5002(15)00281-0.
16. Warren JL, Harlan LC, Trimble EL, et al. Trends in the receipt of guideline care and survival for women with ovarian cancer: A population-based study. *Gynecol Oncol* 2017;145(3):486-492.
17. Jennens RR, Giles GG, Fox RM. Increasing underrepresentation of elderly patients with advanced colorectal or non-small-cell lung cancer in chemotherapy trials. *Int Med J* 2006;36: 216e220.

For peer review only

18. Murthy VH, Krumholtz HM, Gross CP. Participation in cancer clinical trials; race-, sex-, and age-based disparities. *JAMA* 2004;22(291): 2720-2726.
19. Tong Y, Huang C, Zhang J. A novel EGFR-TKI inhibitor (cAMP-H3BO3 complex) combined with thermal therapy is a promising strategy to improve lung cancer treatment outcomes. *Oncotarget.* Published Online First: 04 May 2017.doi: 10.18632/oncotarget.17628.
20. National Health and Family Planning Commission of China. Guideline for Chinese primary lung cancer diagnosis and treatment (2015 edition). *Chinese Journal of Oncology* 2015.37(1):67-78.
21. Xing Wang, Shi Yan, Yaqi Wang, et al.Surgical Quality Surveillance and Sustaining Improvement of Lung Cancer Surgery Based On Standard Operation Procedure(SOP) : Experience of Single Surgical Team. *Chinese Journal of Lung Cancer;* 20(4): 253-258.
22. Jackman DM, Zhang Y, Dalby C, et al. Cost and Survival Analysis Before and After Implementation of Dana-Farber Clinical Pathways for Patients With Stage IV Non-Small-Cell Lung Cancer. *J Oncol Pract* 2017;13(4):e346-e352.
23. Okita A, Yamashita M, Abe K, et al. Variance analysis of a clinical pathway of video-assisted single lobectomy for lung cancer. *Surg Today* 2009;39(2):104-9.
24. Duggan KJ, Descallar J, Vinod SK. Application of Guideline Recommended Treatment in Routine Clinical Practice: A Population-based Study of Stage I-IIIB Non-small Cell Lung Cancer. *Clin Oncol (R Coll Radiol)* 2016;28(10):639-47.
25. Heins MJ, de Jong JD, Spronk I, et al. Adherence to cancer treatment guidelines: influence of general and cancer-specific guideline characteristics. *Eur J Public Health* 2016; pii: ckw234. doi: 10.1093/eurpub/ckw234.
26. Yang S, Cui M, Li HY, et al. Meta-analysis of the effectiveness of Chinese and Western integrative medicine on medium and advanced lung cancer. *Chin J Integr Med* 2012;18(11):862-7.
27. XX L, YW Chen, KS Bi. Resolution of Two- way Recerral Problem in China by Studying British National Health Service System. *Chinese General Practice* 2013; 31(16):2926-29.
28. Y Sun, J Wu, SB Xie, et al. Evaluation of the medical staff clinical pathway adherence: Based on comparison of before and after provider payment reform in Henan Province. *Chinese Journal of Health Policy* 2013;6(5):37-43.
29. Kern H, Kox WJ. Impact of standard procedures and clinical standards on cost-effectiveness and intensive care unit performance in adult patients after cardiac surgery. *Intensive care medicine*. 25(12):1367-1373.
30. Henry SG, Ness RM, Stiles RA, Shintani AK, Dittus RS. A cost analysis of colonoscopy using microcosting and time-and-motion techniques. *J Gen Intern Med* 2007, 22(10):1415-21.
31. SN York. Incremental Cost-Effectiveness Ratio. *Handbook of Disease Burdens and Quality of Life Measures* 2010: 4235-4235.
32. Wong CK, Lang BH, Guo VY, et al. Possible Impact of Incremental Cost-Effectiveness Ratio (ICER) on Decision Making for Cancer Screening in Hong Kong: A Systematic Review. *Appl Health Econ Health Policy* 2016;14(6):647-657.
33. National Bureau of Statistics of China. China Statistical Yearbook 2016. http://www.stats.gov.cn/tjsj/ndsj/2016/indexch.htm (accessed 1 July 2017).
34. Statistics Bureau of Anhui Province. Statistical yearbook of Anhui Province in 2016.

For peer review only

http://www.ahtjj.gov.cn/tjj/web/tjnj_view.jsp?strColId=13787135717978521&_index=1# (accessed 1 July 2017).

35. Wennberg JE, Fisher ES. Finding high quality, efficient providers for value purchasing: cohort methods better than methods based on events. Finding high quality, efficient providers for value purchasing: cohort methods better than methods based on events. *Med Care* 2002,40(10):853-5.
36. Chen LW, Wilson FA, Gregg A, et al. Measuring the Cost and Value of Quality Improvement Initiatives for Local Health Departments. J Public Health Manag Pract. Published Online First: 1 Mar 2017. doi: 10.1097/PHH.0000000000000552.
37. Darling G, Malthaner R, Dickie J, et al. Quality indicators for non-small cell lung cancer operations with use of a modified Delphi consensus process. *Ann Thorac Surg* 2014;98(1):183-90.
38. Fisher A, Manicavasagar V, Sharpe L, et al. A Qualitative Exploration of Clinician Views and Experiences of Treatment Decision-Making in Bipolar II Disorder. *Community Ment Health J.* Published Online First: 19 Jan 2017. doi: 10.1007/s10597-016-0077-4.
39. O' Regan P, Hegarty J. The importance of self-care for fatigue amongst patients undergoing chemotherapy for primary cancer. *Eur J Oncol Nurs* 2017;28:47-55.
40. Dionne-Odom JN, Demark-Wahnefried W, Taylor RA, et al. The self-care practices of family caregivers of persons with poor prognosis cancer: differences by varying levels of caregiver well-being and preparedness. *Support Care Cancer* 2017; 25(8):2437-2444.

For peer review only

Figure 1 Guiding framework for cost-effectiveness evaluation

Figure2 Simulated survival after first diagnosis of lung cancer

Figure 3 Simulated cost by selected socio-demographics and clinical characteristics (TC=total cost; KRMB=1000 Chinese yuan)

Figure 4 Anticipated “procedure-outcome” tree of inpatient lung cancer care (Tx = the xth round of hospitalization; Cx = the xth combination of clinical procedures; Px = possibility of using the xth combinations of clinical procedures; Ox = the xth patient outcome index/indicator)

For peer review only

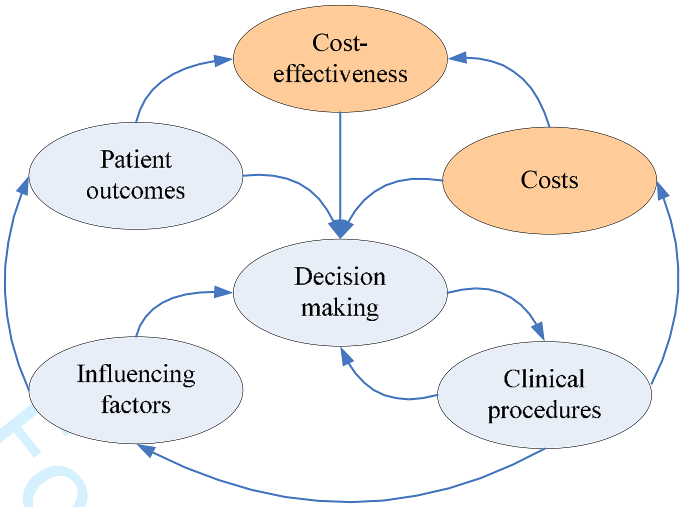

Figure 1 Guiding framework for cost-effectiveness evaluation

For peer review only

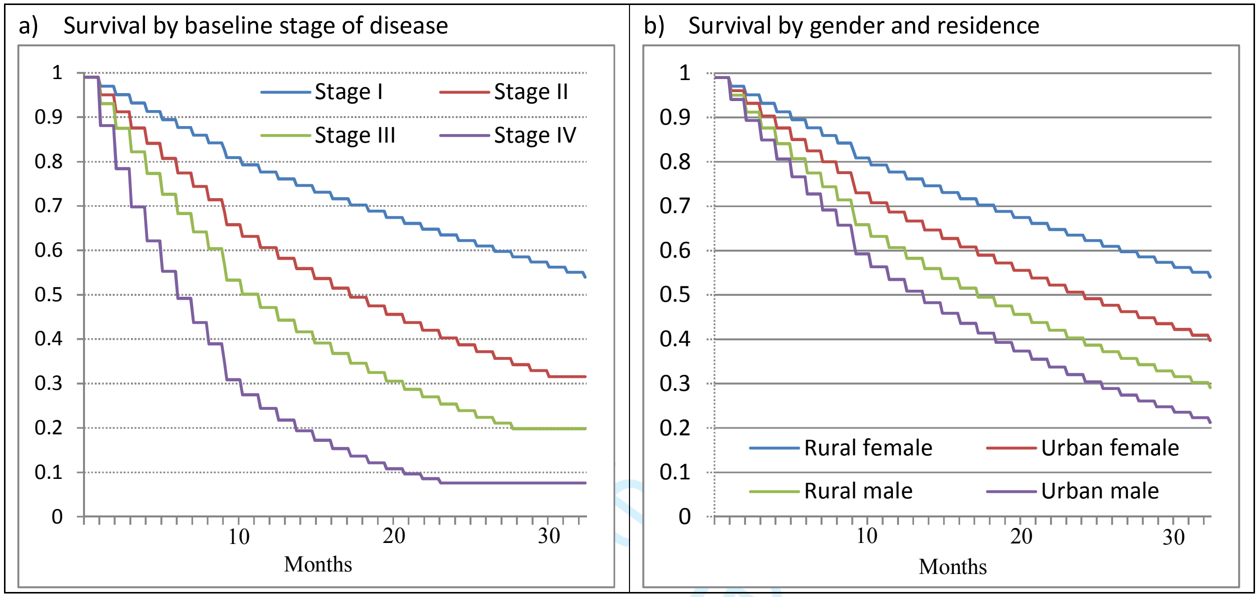

Figure 2 Simulated survival after first diagnosis of lung cancer

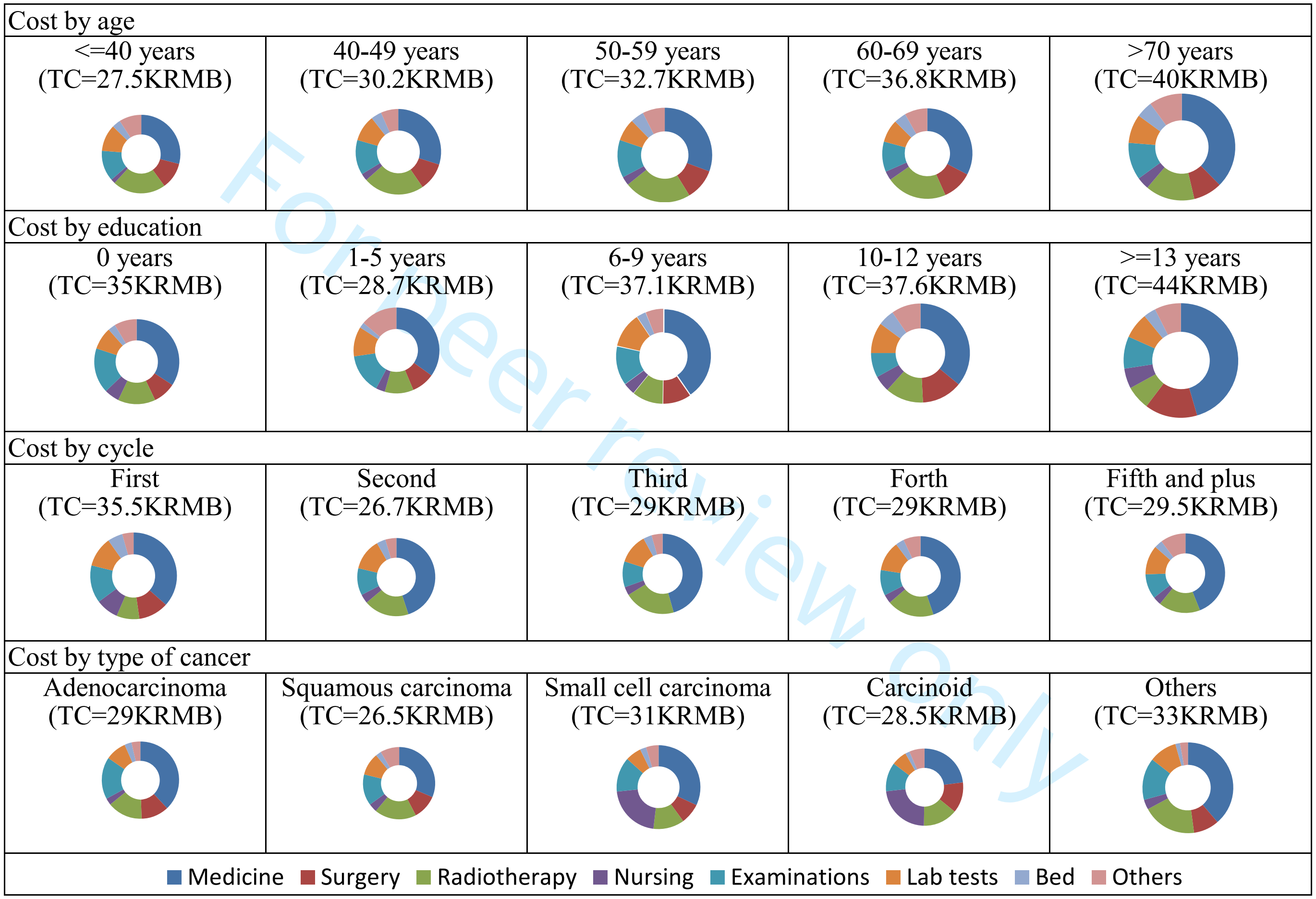

Figure 3 Simulated cost by selected socio-demographics and clinical characteristics (TC=total cost; KRMB=1000 Chinese yuan)

For peer review only

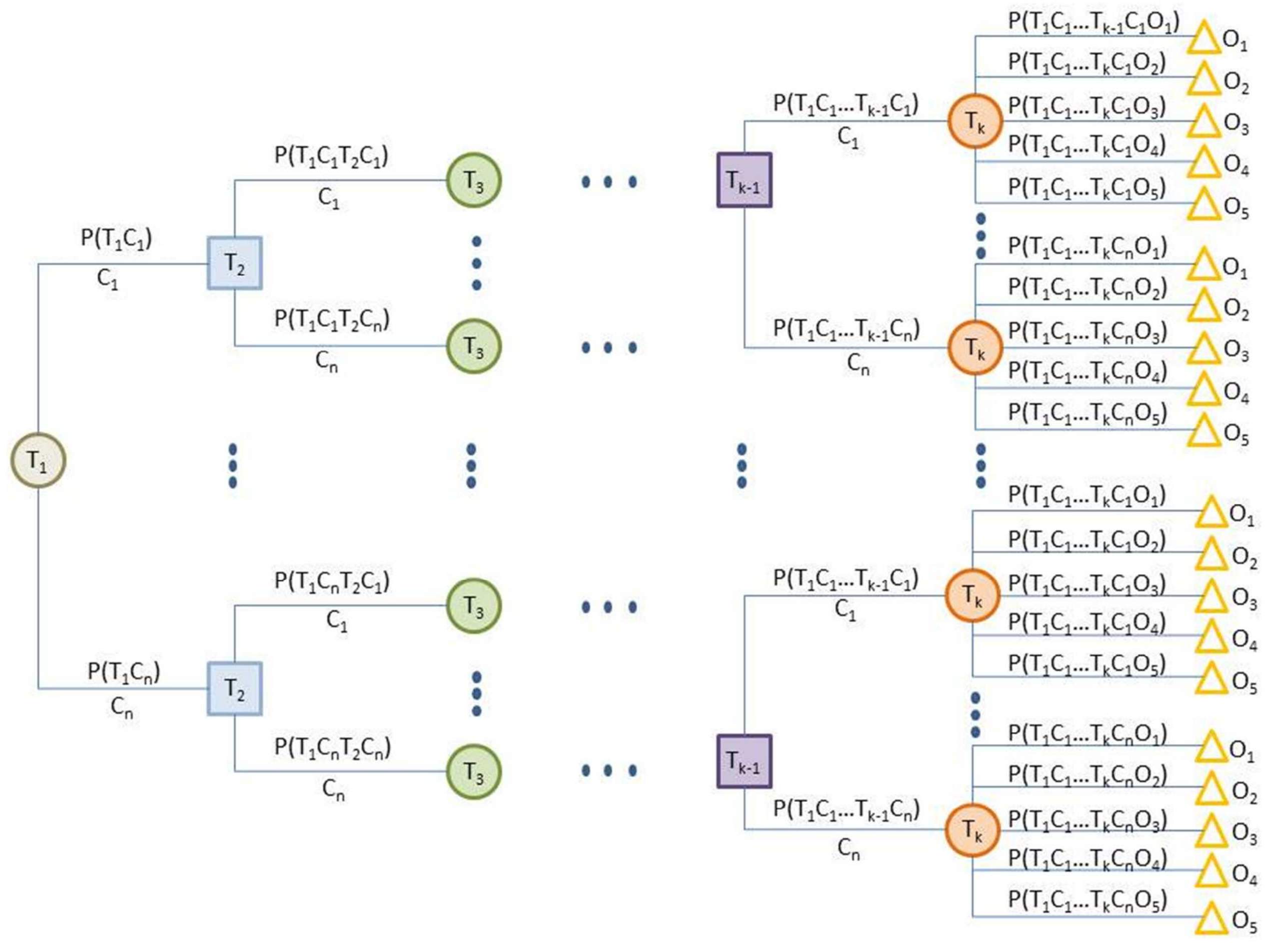

Figure 4 Anticipated "procedure-outcome" tree of inpatient lung cancer care (T_x = the xth round of hospitalization; C_x = the xth combination of clinical procedures; P_x = possibility of using the xth combinations of clinical procedures; O_x = the xth patient outcome index/indicator)

Annex 1 Lung cancer inpatient care data extraction form

Reference Number: |__|__|-|__|__|__|__|__|

Part A: Patient's social demographics

1.1 Case record number: |__|__|__|__|__|__|__|__|
1.2 Patient identification number: |__|__|__|__|__|__|__|__|__|__|__|__|__|__|__|__|
1.3 Sex: [1]Male [2]Female
1.4 Birth date (dd-mm-yyyy, first case record only): |__|__|-|__|__|-|__|__|__|__|
1.5 Body height (centimeter, first case record only): |__|__|__|
1.6 Body weight (kilogram): |__|__|__|.|__|
1.7 Education (first case record only):
[1] No formal education [2] Primary school [3] Middle school
[4] High school [5] College [6] Graduate or higher
[9] Not clear
1.8 Occupation (first case record only):
[1] Staff of public entities [2] Employee of firms [3] Self-employed
[4] Peasant [5] Un-employed [6] Retired
[7] Army member [9]Not clear
1.9 Marital status:
[1] Unmarried [2] Married [3] Divorced
[4] Widowed [5] Other [9] Not clear
1.10 Medical insurance:
[1] Essential medical insurance for urban employees
[2] Medical insurance for urban citizens
[3] New rural cooperative medical care systems
[4] Commercial medical insurance
[5] Public medical care system
[6] Out-of-pocket care
[7] Other
[9] Not clear

Part B: Patient's behavior and disease history (first case record only)

2.1 Smoking:
[1] Current smoker [2] Former smoker [3] Non-smoker
[9] Not clear (skip to 2.2)
2.1.1 Number of cigarettes smoked per day: |__|__|__|
2.1.2 Number of years smoked: |__|__|
2.1.3 Number of years ceased smoking: |__|__|

For peer review only

2.2 Previous diagnosis of the following respiratory diseases:

[1] Tuberculosis [2] Chronic bronchitis [3] Emphysema
[4] Asthma [5] Silicosis/pneumonoconiosis
[6] Other(specify)

2.3 Previous diagnosis of the following cardio-cerebrovascular/endocrine diseases:

[1] Hypertension [2] Coronary heart disease [3] Cerebral thrombosis
[4]Cerebral hemorrhage [5] Hyperlipemia [6] Diabetes
[7] Other(specify)

2.4 Previous diagnosis of cancer (enter location of cancer, if applicable, e.g., breast cancer, colorectal cancer)

[1]	[2]	[3]
[4]	[5]	[6]
[7]	[8]	[9]

(Please add more cells as needed)

2.5 Previous diagnosis of cancer among relatives

Number	Type of relatives	Location of cancer
[1]		
[2]		
[3]		

(Please add more rows as needed)

Part C: Patient's current symptoms/sings

3.1 Respiratory symptoms/signs

[1] Chronic coughing [2] Sputum with blood [3] Chest suppression
[4] Chest pain [5] Difficult breathing [6] Repeated bronchitis
[7] Hoarseness [8]Other (specify)
[9] None

3.2 Symptoms/signs of metabolism or immunity dysfunction:

[1] None [2] Hippocratic fingers/toes [3] Amyasthenia
[4] Hyponatremia [5] Blacken skin folds
[6] Other (specify)

3.3 Symptoms/signs relating to lung cancer metastasis:

[1] None [2] Topical pain [3] Headache
[4] Dizzy [5] Sudden dyskinesia [6] Facial swelling
[7] Other (specify)

3.4 Cancer-related non-specific symptoms/signs:

[1] None [2] Apparent emaciation [3] Weakness
[4] Mild/moderate fever [5] Other (specify)

3.5 Karnofsky score:

[1] |__|__|__|
[2] Not available

3.6 Body surface examination findings:

[1] None

[2] Enlargement of lymph nodes in the neck or supraclavicular region
[3] Lymph node enlargement in other areas
[4] Subcutaneous nodule
[5] Horner syndrome
[6] Facial swelling
[7] Other (specify)
[9] Not clear

Part D: Diagnostic procedures and findings

4 Imaging diagnosis

4.1 Chest X-ray examination:
[1] Not performed (skip to 4.2)
[2] Performed
4.1.1 Date of performance (dd-mm-yyyy): |__|__|-|__|__|-|__|__|__|__|
4.1.2 Abnormalities iddentified
[1] None
[2] Pulmonary nodules/mass
[3] Hilar / mediastinal abnormalities
[4] Pleural effusion
[5]Pericardial effusion
[6] Other (specify)
4.1.2.1 If [2], please specify the largest nodules/mass: |__|__|.|__|*|__|__|.|__|cm
4.2 Chest CT examination:
[1] Not performed (skip to 4.3)
[2] Performed
4.2.1 Date of performance (dd-mm-yyyy): |__|__|-|__|__|-|__|__|__|__|
4.2.2 Type of CT performed
[1] Plain [2] Enhanced scan [3] Plain + enhanced
4.2.3 Layer thickness: |__|__|.|__|cm
4.2.4 Multiple plane reconstruction (MPR):
[1] Yes [2] No
4.2.5 Locations scanned
[1] Chest [2] Chest and abdomen [3] Neck and chest
[4] Neck+chest+abdomen
4.2.6 Abnormalities identified
4.2.6.1 Diagnosis from chest CT
[1] No abnormalities [2] Affirmative benign [3] Suspected benign
[4] Suspected malignant [5] Affirmative malignant
[6] Others (specify)
[9] Not clear
4.2.6.2 Abnormalities identified

For peer review only

[1] Pneumonia [2] Bronchial abnormality [3] Single nodules/mass
[4] Multiple nodules/mass [5] Pleural effusion [6] Pericardial effusion
[7] Other (specify)
4.2.6.2.1 If [3] or[4], size of the largest nodules/mass: |__|__|.|__|*|__|__|.|__|cm

4.3 Head CT examination:
[1] Not performed (skip to 4.4)
[2] Performed
4.3.1 Date of performance (dd-mm-yyyy): |__|__|-|__|__|-|__|__|__|__|
4.3.2 Type of CT performed
[1] Plain [2] Enhanced scan [3] Plain + enhanced
4.3.3 Diagnosis from head CT
[1] No abnormalities [2] Confirmed/suspected brain metastases
[3] Others (specify)

4.4 Head MR examination
[1] Not performed (skip to 4.5)
[2] Performed
4.4.1 Date of performance (dd-mm-yyyy): |__|__|-|__|__|-|__|__|__|__|
4.4.2 Diagnosis from head MR
[1] No abnormalities [2] Single brain metastases [3] Multiple brain metastases
[4] Others (specify)
4.4.2.1 If [2] or [3], size of the largest nodules/mass: |__|__|.|__|*|__|__|.|__|cm

4.5 Chest MR examination
[1] Not performed (skip to 4.6)
[2] Performed
4.5.1 Date of performance (dd-mm-yyyy): |__|__|-|__|__|-|__|__|__|__|
4.5.2 Diagnosis from chest MR
[1] No abnormalities [2] Hilar/mediastinal lymph nodes [3] Lung nodules/mass
[4] Bone metastases [5] Thoracic/pericardial effusion
[6] Others (specify)
4.5.2.1 If [3], size of the largest nodules/mass: |__|__|.|__|*|__|__|.|__|cm
4.5.2.2 If [4], location metastases

4.6 Bone MR examination
[1] Not performed (skip to 4.7)
[2] Performed
4.6.1 Date of performance (dd-mm-yyyy): |__|__|-|__|__|-|__|__|__|__|
4.6.2 Diagnosis from bone MR
[1] No abnormalities [2] Bone metastases
[3] Others (specify)
4.6.2.1 If [2], location of metastases

4.7 Neck ultrasonography
[1] Not performed (skip to 4.8)
[2] Performed
4.7.1 Date of performance (dd-mm-yyyy): |__|__|-|__|__|-|__|__|__|__|
4.7.2 Diagnosis from neck ultrasonography

For peer review only

[1] No abnormalities [2] Neck /supraclavicular lymph nodes
[3] Others (specify)

4.8 Chest ultrasonography
[1] Not performed (skip to 4.9)
[2] Performed
4.8.1 Date of performance (dd-mm-yyyy): |__|__|-|__|__|-|__|__|__|__|
4.8.2 Diagnosis from chest ultrasonography
[1] No abnormalities [2] Pleural effusion [3] Pericardial effusion
[4] Others (specify)
4.9 Abdominal ultrasonography
[1] Not performed (skip to 4.10)
[2] Performed
4.9.1 Date of performance (dd-mm-yyyy): |__|__|-|__|__|-|__|__|__|__|
4.9.2 Diagnosis from abdominal ultrasonography
[1] No abnormalities [2] Liver metastases [3] Adrenal gland transfer
[4] Peritoneal/retroperitoneal lymphadenopathy
[5] Others (specify)
4.10 Bone scans
[1] Not performed (skip to 4.11)
[2] Performed
4.10.1 Date of performance (dd-mm-yyyy): |__|__|-|__|__|-|__|__|__|__|
4.10.2 Diagnosis from bone scans
[1] No abnormalities [2] confirmed metastases [3] Suspected metastases
[4]Others (specify)
4.10.2.1 If [2] or [3], location of metastases
4.11 PET-CT examination
[1] Not performed (skip to 5.1)
[2] Performed
4.11.1 Date of performance (dd-mm-yyyy): |__|__|-|__|__|-|__|__|__|__|
4.11.2 Diagnosis from PET-CT examination
[1] No abnormalities [2] Lung nodules/mass(Primary lesion)
[3] Pulmonary metastasis [4] Lymph node metastasis
[5] Adrenal gland transfer [6] Bone transfer
[7] Other site transfer [8] Thoracic / pericardial effusion
[9] Others (specify)
4.11.3.1 If [2], location of lung nodules/mass
4.11.3.1.1 Size of the largest nodules/mass: |__|__|.|__|*|__|__|.|__|cm
4.11.3.1.2 SUV
4.11.3.1.3 Nature of the nodules/mass identified:
[1] Affirmative benign [2] Suspected benign [3] Suspected malignant
[4] Affirmative malignant [5] Not clear [6] Others (specify)
4.11.3.2 If [3], location of pulmonary metastasis
4.11.3.2.1 SUV

For peer review only

4.11.3.3 If [4], location of lymph node metastasis
4.11.3.3.1SUV
4.11.3.4 If [5], location of adrenal gland metastasis
4.11.3.4.1SUV
4.11.3.5 If [6], location of bone metastases
4.11.3.5.1 SUV
4.11.3.6 If [7], location of other metastases
4.11.3.6.1 SUV

5 Endoscopic examinations

5.1 Fiberoptic bronchoscopy
[1] Not performed (skip to 5.2)
[2] Performed
5.1.1 Date of performance (dd-mm-yyyy): |__|__|-|__|__|-|__|__|__|__|
5.1.2 Diagnosis from fiberoptic bronchoscopy
[1] No abnormalities [2] Tumor
[3] Others (specify)
[4] Not clear
5.2 Lavage cytology/brushing
[1] Not performed (skip to 5.3)
[2] Not clear (skip to 5.3)
[3] Performed
5.2.1 Date of performance (dd-mm-yyyy): |__|__|-|__|__|-|__|__|__|__|
5.3 Bronchoscopy clamp biopsy
[1] Not performed (skip to 5.4)
[2] Not clear (skip to 5.4)
[3] Performed
5.3.1 Date of performance (dd-mm-yyyy): |__|__|-|__|__|-|__|__|__|__|
5.4 Bronchoscopy aspiration biopsy
[1] Not performed (skip to 5.5)
[2] Not clear (skip to 5.5)
[3] Performed
5.4.1 Date of performance (dd-mm-yyyy): |__|__|-|__|__|-|__|__|__|__|
5.4.2 Type of bronchoscopy aspiration biopsy
[1] Endobroncheal ultrasonography [2] Electromagnetic-guided
[3] Transbronchial needle aspiration [4] Not clear
[5] Others (specify)

For peer review only

6 Laboratory/biological tests

6.0 Date of performance (dd-mm-yyyy): |__|__|-|__|__|-|__|__|__|__|
6.1 CEA
[1] Not performed (skip to 6.2)
[2] Not clear (skip to 6.2)
[3] Performed
6.1.1 Date of performance if different from 6.0
(dd-mm-yyyy): |__|__|-|__|__|-|__|__|__|__|
6.1.2 Test result (value-unit):______-______
6.2 CA125
[1] Not performed (skip to 6.3)
[2] Not clear (skip to 6.3)
[3] Performed
6.2.1 Date of performance if different from 6.0
(dd-mm-yyyy): |__|__|-|__|__|-|__|__|__|__|
6.2.2 Test result (value-unit):______-______
6.3 proGRP
[1] Not performed (skip to 6.4)
[2] Not clear (skip to 6.4)
[3] Performed
6.3.1 Date of performance if different from 6.0
(dd-mm-yyyy): |__|__|-|__|__|-|__|__|__|__|
6.3.2 Test result (value-unit):______-______
6.4 SCC
[1] Not performed (skip to 6.5)
[2] Not clear (skip to 6.5)
[3] Performed
6.4.1 Date of performance if different from 6.0
(dd-mm-yyyy): |__|__|-|__|__|-|__|__|__|__|
6.4.2 Test result (value-unit):______-______
6.5 NSE
[1] Not performed (skip to 6)
[2] Not clear (skip to 6.6)
[3] Performed
6.5.1 Date of performance if different from 6.0
(dd-mm-yyyy): |__|__|-|__|__|-|__|__|__|__|
6.5.2 Test result (value-unit):______-______
6.6 CYFRA21-1
[1] Not performed (skip to 6.7)
[2] Not clear (skip to 6.7)
[3] Performed
6.6.1 Date of performance if different from 6.0

For peer review only

(dd-mm-yyyy): |__|__|-|__|__|-|__|__|__|__|
6.6.2 Test result (value-unit):_______-_______

6.7 WBC
[1] Not performed (skip to 6.8)
[2] Not clear (skip to 6.8)
[3] Performed
6.7.1 Date of performance if different from 6.0
(dd-mm-yyyy): |__|__|-|__|__|-|__|__|__|__|
6.7.2 Test result (value-unit):_______-_______

6.8 PLT
[1] Not performed (skip to 6.9)
[2] Not clear (skip to 6.9)
[3] Performed
6.8.1 Date of performance if different from 6.0
(dd-mm-yyyy): |__|__|-|__|__|-|__|__|__|__|
6.8.2 Test result (value-unit):_______-_______

6.9 Hb
[1] Not performed (skip to 6.10)
[2] Not clear (skip to 6.10)
[3] Performed
6.9.1 Date of performance if different from 6.0
(dd-mm-yyyy): |__|__|-|__|__|-|__|__|__|__|
6.9.2 Test result (value-unit):_______-_______

6.10 ALB
[1] Not performed (skip to 6.11)
[2] Not clear (skip to 6.11)
[3] Performed
6.10.1 Date of performance if different from 6.0
(dd-mm-yyyy): |__|__|-|__|__|-|__|__|__|__|
6.10.2 Test result (value-unit):_______-_______

6.11 Pre-ALB
[1] Not performed (skip to 6.12)
[2] Not clear (skip to 6.12)
[3] Performed
6.11.1 Date of performance if different from 6.0
(dd-mm-yyyy): |__|__|-|__|__|-|__|__|__|__|
6.11.2 Test result (value-unit):_______-_______

6.12 Ca
[1] Not performed (skip to 6.13)
[2] Not clear (skip to 6.13)
[3] Performed
6.12.1 Date of performance if different from 6.0
(dd-mm-yyyy): |__|__|-|__|__|-|__|__|__|__|
6.12.2 Test result (value-unit):_______-_______

For peer review only

6.13 Fe
[1] Not performed (skip to 6.14)
[2] Not clear (skip to 6.14)
[3] Performed
6.13.1 Date of performance if different from 6.0
(dd-mm-yyyy): |__|__|-|__|__|-|__|__|__|__|
6.13.2 Test result (value-unit):______-______
6.14 FIB
[1] Not performed (skip to 6.15)
[2] Not clear (skip to 6.15)
[3] Performed
6.14.1 Date of performance if different from 6.0
(dd-mm-yyyy): |__|__|-|__|__|-|__|__|__|__|
6.14.2 Test result (value-unit):______-______
6.15 D-D
[1] Not performed (skip to 6.16)
[2] Not clear (skip to 6.16)
[3] Performed
6.15.1 Date of performance if different from 6.0
(dd-mm-yyyy): |__|__|-|__|__|-|__|__|__|__|
6.15.2 Test result (value-unit):______-______
6.16 Na
[1] Not performed (skip to 6.17)
[2] Not clear (skip to 6.17)
[3] Performed
6.16.1 Date of performance if different from 6.0
(dd-mm-yyyy): |__|__|-|__|__|-|__|__|__|__|
6.16.2 Test result (value-unit):______-______
6.17 LDL
[1] Not performed (skip to 6.18)
[2] Not clear (skip to 6.18)
[3] Performed
6.17.1 Date of performance if different from 6.0
(dd-mm-yyyy): |__|__|-|__|__|-|__|__|__|__|
6.17.2 Test result (value-unit):______-______
6.18 LDL
[1] Not performed (skip to 6.19)
[2] Not clear (skip to 6.19)
[3] Performed
6.18.1 Date of performance if different from 6.0
(dd-mm-yyyy): |__|__|-|__|__|-|__|__|__|__|
6.18.2 Test result (value-unit):______-______

For peer review only

6.19 TG
[1] Not performed (skip to 6.20)
[2] Not clear (skip to 6.20)
[3] Performed
6.19.1 Date of performance if different from 6.0
(dd-mm-yyyy): |__|__|-|__|__|-|__|__|__|__|
6.19.2 Test result (value-unit):______-______

6.20 TCHOL
[1] Not performed (skip to 7.1)
[2] Not clear (skip to 7.1)
[3] Performed
6.20.1 Date of performance if different from 6.0
(dd-mm-yyyy): |__|__|-|__|__|-|__|__|__|__|
6.20.2 Test result (value-unit):______-______

7 Heart and lung function examinations

7.1 Electrocardiogram examination
[1] Not performed (skip to 7.2)
[2] Performed
7.1.1 Date of performance (dd-mm-yyyy): |__|__|-|__|__|-|__|__|__|__|
7.1.2 Heart rate: |__|__|__| times/minutes
7.1.3 Diagnosis from electrocardiogram examination
[1] No abnormalities
[2] Abnormalities(specify)

7.2 Lung function examinations
[1] Not performed (skip to 8.1)
[2] Not clear (skip to 8.1)
[3] Performed
7.2.1 Date of performance (dd-mm-yyyy): |__|__|-|__|__|-|__|__|__|__|
7.2.2 FVC (Tested/predicted value): /
7.2.3 FEV1(Tested/predicted value): /
7.2.4 FEV1/FVC%(Tested/predicted value): /
7.2.5 TLCO SB(Tested/predicted value): /
7.2.6 Ventilation function assessment:
[1] No abnormalities [2] Mildly reduced [3] Moderately reduced
[4] Severely reduced [5] Restrictive [6] Obstruction
[7] Mixed [8] Not clear
7.2.7 Lung capacity
[1] No abnormalities [2] Increased total residue ratio [3] Low lung capacity
[4] Not clear
7.2.8 Breath diffusion
[1] No abnormalities [2] Reduced [3] Not clear

For peer review only

8 Histological/cytological examination

8.1 Preoperative cytological
[1] Not performed (skip to 8.2)
[2] Not clear (skip to 8.2)
[3] Performed
8.1.1 If [3], preoperative cytological method:
[1] Needle biopsy [2] Sputum specimen examination [3] Bronchial lavage
[4] Others (specify)
8.1.2 If [3], preoperative cytological result:
[1] With cancer cells [2] Without cancer cells [3] Uncertain lesion
[4] Not clear
8.1.2.1 If [1], cytological type
[1] Adenocarcinoma [2] Squamous cell carcinoma
[3] Small cell carcinoma [4] Carcinoid
[5] Large cell carcinoma [6] Squamous cell carcinoma
[7] Sarcomatoid carcinoma [8] carcinoma from sialaden
[9] Not clear [10] Others (specify)
8.1.2.1.1 If [1], first class subtype code
[1] Pre-invasion lesion [2] Microinvasive adenocarcinoma
[3] Invasive adenocarcinoma [4] Variant invasive adenocarcinoma
[5] Others (specify)
[6] Not clear
8.1.2.1.1.1 If [1], second class subtype code
[1] Atypical adenocarcinoma like hyperplasia
[2] Adenocarcinoma in situ
[6] Not clear
8.1.2.1.1.2 If [3], second class subtype code
[1] Accumbens dominated [2] Acinar dominated
[3] Papillary dominated [4] Micro papillae dominated
[5] Entities with mucus dominated
[6] Not clear
8.1.2.1.1.3 If [4], second class subtype code
[1] Mucinous invasive adenocarcinoma
[2] Colloid [3] Fetal
[4] Intestinal [5] Others (specify)
[6] Not clear

8.2 Preoperative histological
[1] Not performed (skip to 10.4)
[2] Not clear (skip to 10.4)
[3] Performed
8.2.1 If [3], method of preoperative histological biopsy:
[1] Ultrasound guided aspiration biopsy [2] CT guided aspiration biopsy
[3] Bronchoscopic biopsy [4] Nuclear magnetic puncture

For peer review only

[5] Not clear [6] Others (specify)

8.2.1.1 If [3], results of preoperative histological biopsy:

[1] With cancer cells [2] Without cancer cells [3] Uncertain lesion

[4] Not clear

8.2.2.1 If [1], histological type:

[1] Adenocarcinoma [2] Squamous cell carcinoma

[3] Small cell carcinoma [4] Carcinoid

[5] Large cell carcinoma [6] Squamous cell carcinoma

[7] Sarcomatoid carcinoma [8] carcinoma from sialaden

[9] Not clear [10] Others (specify)

8.2.2.1.1.1 If [1], second class subtype code

[1] Atypical adenocarcinoma like hyperplasia

[2] Adenocarcinoma in situ

[6] Not clear

8.2.2.1.1.2 If [3], second class subtype code

[1] Accumbens dominated [2] Acinar dominated

[3] Papillary dominated [4] Micro papillae dominated

[5] Entities with mucus dominated

[6] Not clear

8.2.2.1.1.3 If [4], second class subtype code

[1] Mucinous invasive adenocarcinoma

[2] Colloid [3] Fetal

[4] Intestinal [5] Others (specify)

[6] Not clear

8.2.2.2 If 8.2.2.1 information not available, please tick in histology type:

[1] Small cell lung cancer [2] Non-small cell lung cancer [3] Benign lesion

[4] Not clear [5] Others (specify)

8.3 Biopsy of frozen mass:

[1] Not performed (skip to 8.4)

[2] Not clear (skip to 8.4)

[3] Performed

8.3.1 If [3], diagnosis of frozen mass biopsy:

[1] Adenocarcinoma [2] Squamous cell carcinoma

[3] Small cell carcinoma [4] Carcinoid

[5] Large cell carcinoma [6] Squamous cell carcinoma

[7] Sarcomatoid carcinoma [8] carcinoma from sialaden

[9] Not clear [10] Others (specify)

8.3.2.1.1.1 If [1], second class subtype code

[1] Atypical adenocarcinoma like hyperplasia

[2] Adenocarcinoma in situ

[6] Not clear

8.3.2.1.1.2 If [3], second class subtype code

[1] Accumbens dominated [2] Acinar dominated

[3] Papillary dominated [4] Micro papillae dominated

For peer review only

[5] Entities with mucus dominated
[6] Not clear
8.3.2.1.1.3 If [4], second class subtype code
[1] Mucinous invasive adenocarcinoma
[2] Colloid [3] Fetal
[4] Intestinal [5] Others (specify)
[6] Not clear

8.4 Biopsy of lymph node:
[1] Not performed (skip to 8.5)
[2] Not clear (skip to 8.5)
[3] Performed
8.4.1 If [3], result of lymph node biopsy:
[1] Metastasis [2] No metastasis

8.5 Biopsy of frozen margin of bronchus:
[1] Not performed (skip to 8.6)
[2] Not clear (skip to 8.6)
[3] Performed
8.5.1 If [3], result of frozen margin of bronchus:
[1] Margin tumor [2] No margin tumor

8.6 Postoperative histological
[1] Not performed (skip to 9)
[2] Not clear (skip to 9)
[3] Performed
8.6.1 If [3], number of tumors:
[1] Solitary tumor [2] More than 2 nodules [3] Not clear
8.6.1.1 The largest tumor size: |__|__|*|__|__|*|__|__|cm
8.6.1.2 If multiple tumor, the smallest tumor size: |__|__|*|__|__|*|__|__|cm
8.6.2 Pathologic diagnosis
[1] Adenocarcinoma [2] Squamous cell carcinoma
[3] Small cell carcinoma [4] Carcinoid
[5] Large cell carcinoma [6] Squamous cell carcinoma
[7] Sarcomatoid carcinoma [8] carcinoma from sialaden
[9] Not clear [10] Others (specify)
8.6.2.1 If [1], second class subtype code
[1] Atypical adenocarcinoma like hyperplasia
[2] Adenocarcinoma in situ
[6] Not clear
8.6.2.1.1 If [3], second class subtype code
[1] Accumbens dominated [2] Acinar dominated
[3] Papillary dominated [4] Micro papillae dominated
[5] Entities with mucus dominated
[6] Not clear
8.6.2.1.2 If [4], second class subtype code
[1] Mucinous invasive adenocarcinoma

For peer review only

[2] Colloid [3] Fetal
[4] Intestinal [5] Others (specify)
[6] Not clear

8.6.3 Differentiation degree:
[1] Well differentiated [2] Well and moderately differentiated
[3] Moderately differentiated [4] Poorly differentiated
[5] Middle and low differentiation [6] Undifferentiated
[7] Not clear

8.6.4 Associated with intrapulmonary metastasis
[1] Yes [2] No (skip to 10.11) [3] Not clear(skip to 10.11)

10.10.1 Invasion of pleura?
[1] Yes [2] No [3] Not clear

8.6.4.1 Invasion of the main bronchi?
[1] Yes, distance is less than 2cm [2] Yes, distance is more than 2cm
[3] No [3] Not clear

8.6.4.2 Invasion of chest wall/septum/mediastinum/pericardium?
[1] Yes(specify) [2] No [3] Not clear

8.6.4.3 Invasion of mediastinum/heart/trachea/esophagus/vertebral body/carina?
[1] Yes(specify) [2] No [3] Not clear

8.7 Resection margin positive?
[1] Not performed (skip to 10.6)
[2] Not clear (skip to 10.6)
[3] Positive
[4] Negative

8.8 The total number of lymph nodes detected

8.9 The total number of lymph node metastasis

8.10 Lymph node metastasis site
[1] No metastasis [2] Ipsilateral bronchi or hilum
[3] Ipsilateral mediastinum or carina [4] Contralateral mediastinum or hilum of lung, clavicle
[5] Not clear

9 Tumor maker

9.1 Her-2(C-erbB-2) detection
[1] Not performed (skip to 9.2)
[2] Not clear (skip to 9.2)
[3] Performed (skip to 9.2)

9.1.1 If [3], method of detection
[1] Immunohistochemistry [2] FISH [3] Other(Specify)

9.1.2 If [3], result of detection
[1] Positive [2] Negative [3] Other(Specify) [4] Not clear

9.2 Anaplastic lymphoma kinase detection
[1] Not performed (skip to 9.3)
[2] Not clear (skip to 9.3)
[3] Performed (skip to 9.3)

9.2.1 If [3], method of detection

For peer review only

[1] Immunohistochemistry [2] Genetic testing [3] Other(Specify)
9.2.2 If [3], result of detection
[1] Positive [2] Negative [3] Other(Specify) [4] Not clear
9.3 Epidermal growth factor receptor detection
[1] Not performed (skip to 9.4)
[2] Not clear (skip to 9.4)
[3] Performed (skip to 9.4)
9.4.1 If [3], method of detection
[1] Immunohistochemistry [2] Genetic testing [3] Other(Specify)
9.3.2 If [3], result of detection
[1] Positive [2] Negative [3] Other(Specify) [4] Not clear
9.4 K-ras detection
[1] Not performed (skip to 9.5)
[2] Not clear (skip to 9.5)
[3] Performed (skip to 9.5)
9.4.1 If [3], method of detection
[1] Immunohistochemistry [2] Gene mutation detection [3] Other(Specify)
9.4.2 If [3], result of detection
[1] Positive [2] Negative [3] Other(Specify) [4] Not clear
9.5 Other factor types detection
[1] Not performed (skip to 9.6)
[2] Not clear (skip to 9.6)
[3] Performed (skip to 9.6)
9.6.1 If [3], method of detection
[1] Immunohistochemistry [2] Gene mutation detection [3] Other(Specify)
9.6.2 If [3], result of detection
[1] Positive [2] Negative [3] Other(Specify) [4] Not clear

9 Staging of lung cancer

9.1 Type of staging available
[1] Clinical stage [2] Pathological staging [3] Not staging
[4] Not clear
9.2 Staging methods
[1] Clinical imaging [2] Pathological staging [3] Postoperative pathology
[4] No [5] Not clear
9.3 If staged, details of TNM staging
9.3.1 Staging system
[1] The 6th edition of UICC/AJCC staging, published in 2002
[2] The 7th edition of AHCC staging, published in 2009
9.3.2 T staging
[1] T1; [2] T2; [3] T3; [4] T4; [5] Tx; [6] Not clear
9.3.3 N staging
[1] N1; [2] N2; [3] N3; [4] N0; [5] Not clear

For peer review only

9.3.4 M staging

[1] M1; [2] Mx; [3]M0; [4] Not clear

9.3.5 TNM staging

[1] Stage I; [2] Stage IIA; [3] Stage IIB; [4] Stage IIIA;
[5] Stage IIIB; [6] Stage IV; [7] Others (specify); [8] Not clear

9.4 Type of lung cancer:

[1] Small cell lung cancer [2] Non-Small cell lung cancer
[3] Mixed small cell lung cancer [4] Not clear
[5] Others (specify)

9.4.1 If [1], state of lesion

[1] Restricted [2] Pervasive
[3] Other (specify)

9.4.2 If [2], state of lesion

[1] Early stage [2] Locally advanced
[3] Advanced [4] Not clear

Part E: Treatment procedures and findings/results

9.1 Surgical treatment

[1] Not performed (skip to 9.2)
[2] Thoracotomy
[3] Video-assisted thoracoscopic surgery
[4] Thoracoscope assisted small incision surgery
[5] Others (specify)
[6] Not clear(skip to 9.2)

9.1.1 Details of resection:

[1] Lobectomy [2] Segmental resection
[3] Combined lobectomy [4] Completely pneumonectomy
[5] Sleeve lobectomy [6] Resection and reconstruction of carina
[7] Others (specify) [8] Not clear

9.1.1.1 If [2], name of the segment

9.1.1.2 If [4], treatment of pulmonary arteriovenous in pericardium

[1] Yes [2] No [3] Not clear

9.1.2 If [3], type of thoracoscope assistance:

[1] Single hole [2] Double holes [3] Three holes
[4] Multiple holes [5] Not clear

9.1.2.1 Conversion from video-assisted thoracoscopic surgery to Thoracotomy

[1] Yes [2] No [3] Not clear

9.1.3 Performance of rapid pathology

[1] Yes [2] No [3] Not clear

9.1.4 Findings from intraoperative exploration

9.1.4.1 Tumor site

[1] Left [2] Right [3] Upper lobes
[4] Bottom lobes [5] Middle lobes [6] Not clear

For peer review only

9.1.4.2 Cross lobes
[1] Yes [2] No [3] Not clear
9.1.4.3 Pleural involvement/ Shrinkage
[1] Yes [2] No [3] Not clear
9.1.4.4 Largest diameter of tumor: |__|__|.|__|cm
9.1.4.5 Pleural metastasis
[1] Yes [2] No [3] Not clear
9.1.4.6 Intrapulmonary metastasis
[1] Yes [2] No [3] Not clear
9.1.4.7 Foreign invasion
[1] Yes [2] No [3] Not clear
9.1.4.7.1 If [1], name of invaded tissue:
9.1.4.8 Dual(Multiple) primary tumor
[1] Yes [2] No [3] Not clear
9.1.5 Lymph node dissection
[1] Systematicness [2] Sampling [3] Not cleaned [4] Not Clear
9.1.6 Classification of surgery
[1] Radical cure [2] Palliative treatment [3] Not clear

9.2 Radiation therapy
[1] Not performed (skip to 9.3)
[2] Not clear (skip to 9.3)
[3] Performed
9.2.1 If [3], type of radiation therapy:
[1] Preoperative radiotherapy [2] Postoperative radiotherapy
[3] Radical radiation therapy
9.2.1.1 Combined with chemotherapy:
[1] Not performed (skip to 10.1.3)
[2] Not clear (skip to 10.1.3)
[3] Performed
9.2.1.1.1 If [3], type of chemo-radiotherapy:
[1] Sequence chemoradiotherapy [2] Concurrent chemoradiotherapy
9.2.1.1.2 If [2], name of the chemotherapy drugs
9.2.1.1.3 If [2], chemotherapy cycles:
[1] Every week [2] Biweekly [3] Every 3 weeks
[4] Every 4 weeks [5] Not clear
9.2.1.2 Radiotherapy technique
[1] Routine radiotherapy [2] Three-dimensional conformal radiotherapy
[3] Tomo treatment [4] Static intensity modulated radiotherapy
[5] Stereotactic radiotherapy [6] Rotational intensity modulated radiotherapy
[7] Not clear [8] Others (specify)
9.2.1.3 Polarization
[1] Conventional simulator [2] CT simulation [3] 4D-CT
[4] Not clear
9.2.1.4 Methods of pretreatment position verification

For peer review only

[1] No methods [2] Image guide radiation therapy
[3] Not clear [4] Electronic Portal Imaging Device
[5] Others (specify)

9.2.1.5 Radiation target area (multiple choice)

[1] Primary foci [2] Postoperative stump and tumor bed
[3] Involving lymph node irradiation [4] Choose lymph node irradiation
[5] Metastatic lesions [6] Not clear

9.2.1.6 Radiotherapy dose division program

No	Radiation energy	Total dose Gy	Number of times	Treatment time (days)
[1]				
[2]				
[3]				

9.3 Chemotherapy

[1] Not performed (skip to 9.4)
[2] Not clear (skip to 9.4)
[3] Performed

9.3.1 If [3], type of chemotherapy:

[1] Neoadjuvant chemotherapy [2] Postoperative adjuvant chemotherapy
[3] Advanced chemotherapy [4] Others (specify)

9.3.1.1 If [1], neoadjuvant chemotherapy regimen

[1] Vinorelbin/Cisplatin+Vinorelbin/Carboplatin+Vinorelbin/Other platinum
[2] Paclitaxel/Cisplatin+Paclitaxel/Carboplatin+Paclitaxel/Other platinum
[3] Docetaxel/Cisplatin+ Docetaxel/Carboplatin +Docetaxel/Other platinum
[4] Pemetrexed/Cisplatin+Pemetrexed/Carboplatin+ Pemetrexed/Other platinum
[5] Gemcitabine/Cisplatin +Gemcitabine/Carboplatin +Gemcitabine/Other platinum
[6] Others (specify)
[7] Not clear

9.3.1.2 If [2], postoperative adjuvant chemotherapy regimen:

[1] Vinorelbin/Cisplatin+Vinorelbin/Carboplatin+Vinorelbin/Other platinum
[2] Paclitaxel/Cisplatin+Paclitaxel/Carboplatin+Paclitaxel/Other platinum
[3] Docetaxel/Cisplatin+Docetaxel/Carboplatin+Docetaxel/Other platinum
[4] Pemetrexed/Cisplatin+Pemetrexed/Carboplatin+Pemetrexed/Other platinum
[5] Gemcitabine/Cisplatin+Gemcitabine/Carboplatin+Gemcitabine/Other platinum
[6] Etoposide/Cisplatin+Etoposide/Carboplatin+Cyclophosphamide/Adriamycin/ Vincristine
[7] Others (specify)
[8] Not clear

9.3.1.3 If [3], advanced chemotherapy regimen:

[1] Cisplatin+Carboplatin+Other platinum
[2] Paclitaxel+Docetaxel
[3] Emcitabine
[4] Pemetrexed
[5] Vinorelbine+Vincristine
[6] Irinotecan+Topotecan

For peer review only

[7] Tegafur
[8] Etoposide
[9] Cytoxan+Ifosfamide
[10] Adriamycin
[11] Others(specify)
[12] Not clear

9.4 Complication treatment

9.4.1 Superior vena cava syndrome
[1] Not appeared(skip to 9.4.2) [2] Not clear(skip to 9.4.2) [3] Appeared
9.4.1.1 If [3], duration (month):
9.4.1.2 If [3], treatment:
[1] No (skip to 9.4.2) [2] Not clear(skip to 9.4.2) [3] Yes
9.4.1.2.1 If[3], treatment effect:
[1] Improved [2] Progressed [3] Stable [4] Not clear

9.4.2 Spinal cord compression syndrome
[1] Not appeared (skip to 9.4.3) [2] Not clear(skip to 9.4.3) [3] Appear
9.4.2.1 If [3], duration (month):
9.4.2.2 If [3], treatment:
[1] No (skip to 9.4.3) [2] Not clear(skip to 9.4.3) [3] Yes
9.4.2.2.1 If [3], treatment effect:
[1] Improved [2] Progressed [3] Stable [4] Not clear

9.4.3 Brain metastases
[1] Not appeared (skip to 9.4.4) [2] Not clear(skip to 9.4.4) [3] Appear
9.4.3.1 If [3], duration (month):
9.4.3.2 If [3], treatment:
[1] No (skip to 9.4.4) [2] Not clear(skip to 9.4.4) [3] Yes
9.4.3.2.1 If [3], treatment effect:
[1] Improved [2] Progressed [3] Stable [4] Not clear

9.4.4 Meningeal metastases
[1] Not appeared (skip to 9.4.5) [2] Not clear(skip to 9.4.5) [3] Appear
9.4.4.1 If [3], duration (month):
9.4.4.2 If [3], treatment:
[1] No (skip to 9.4.5) [2] Not clear(skip to 9.4.5) [3] Yes
9.4.4.2.1 If [3], treatment effect:
[1] Improved [2] Progressed [3] Stable [4] Not clear

9.4.5 Pleural effusion
[1] Not appeared (skip to 9.4.6) [2] Not clear(skip to 9.4.6) [3] Appear
9.4.5.1 If [3], duration (month):
9.4.5.2 If [3], treatment:
[1] No (skip to 9.4.6) [2] Not clear(skip to 9.4.6) [3] Yes
9.4.5.2.1 If [3], treatment effect:
[1] Improved [2] Progressed [3] Stable [4] Not clear

9.4.6 Pyoperitoneum
[1] Not appeared (skip to 9.4.7) [2] Not clear(skip to 9.4.7) [3] Appear

For peer review only

9.4.6.1 If [3], duration (month):

9.4.6.2 If [3], treatment:

[1] No (skip to 9.4.7) [2] Not clear(skip to 9.4.7) [3] Yes

9.4.6.2.1 If [3], treatment effect:

[1] Improved [2] Progressed [3] Stable [4] Not clear

9.4.7 Pericardial effusion

[1] Not appeared(skip to 9.4.8) [2] Not clear(skip to 9.4.8) [3] Appear

9.4.7.1 If [3], duration (month):

9.4.7.2 If [3], treatment:

[1] No (skip to 9.4.8) [2] Not clear(skip to 9.4.8) [3] Yes

9.4.7.2.1 If [3], treatment effect:

[1] Improved [2] Progressed [3] Stable [4] Not clear

9.4.8 Intestinal obstruction

[1] Not appeared(skip to 9.4.9) [2] Not clear(skip to 9.4.9) [3] Appear

9.4.8.1 If [3], duration (month):

9.4.8.2 If [3], treatment:

[1] No (skip to 9.4.9) [2] Not clear(skip to 9.4.9) [3] Yes

9.4.8.2.1 If [3], treatment effect:

[1] Improved [2] Progressed [3] Stable [4] Not clear

9.4.9 Pain

[1] Not appeared (skip to 9.4.10) [2] Not clear(skip to 9.4.10) [3] Appear

9.4.9.1 If [3], duration (month):

9.4.9.2 If [3], treatment:

[1] No (skip to 9.4.10) [2] Not clear(skip to 9.4.10) [3] Yes

9.4.9.2.1 If [3], treatment effect (site and score):

9.4.10 Cerebral thrombosis/ hemorrhage

[1] Not appeared (skip to 9.4.11) [2] Not clear(skip to 9.4.11) [3] Appear

9.4.10.1 If [3], duration (month):

9.4.10.2 If [3], treatment:

[1] No (skip to 9.4.11) [2] Not clear(skip to 9.4.11) [3] Yes

9.4.10.2.1 If [3], treatment effect:

[1] Improved [2] Progressed [3] Stable [4] Not clear

9.4.11 Interstitial pneumonia

[1] Not appeared(skip to 9.4.12) [2] Not clear(skip to 9.4.12) [3] Appear

9.4.11.1 If [3], duration (month):

9.4.11.2 If [3], treatment:

[1] No (skip to 9.4.12) [2] Not clear(skip to 9.4.12) [3] Yes

9.4.11.2.1 If [3], treatment effect:

[1] Improved [2] Progressed [3] Stable [4] Not clear

9.4.12 Pulmonary embolism

[1] Not appeared(skip to 9.4.13) [2] Not clear(skip to 9.4.13) [3] Appear

9.4.12.1 If [3], duration (month):

9.4.12.2 If [3], treatment:

[1] No (skip to 9.4.13) [2] Not clear(skip to 9.4.13) [3] Yes

For peer review only

9.4.12.2.1 If [3], treatment effect:
[1] Improved [2] Progressed [3] Stable [4] Not clear

9.4.13 Cardiac insufficiency
[1] Not appeared(skip to 9.4.14) [2] Not clear(skip to 9.4.14) [3] Appear
9.4.13.1 If [3], duration (month):
9.4.13.2 If [3], treatment:
[1] No (skip to 9.4.14) [2] Not clear(skip to 9.4.14) [3] Yes
9.4.13.2.1 If [3], treatment effect:
[1] Improved [2] Progressed [3] Stable [4] Not clear

9.4.14 Arrhythmia
[1] Not appeared(skip to 9.4.15) [2] Not clear(skip to 9.4.15) [3] Appear
9.4.14.1 If [3], duration (month):
9.4.14.2 If [3], treatment:
[1] No (skip to 9.4.15) [2] Not clear(skip to 9.4.15) [3] Yes
9.4.14.2.1 If [3], treatment effect:
[1] Improved [2] Progressed [3] Stable [4] Not clear

9.4.15 Hypercoagulable state
[1] Not appeared (skip to 9.5) [2] Not clear(skip to 9.5) [3] Appear
9.4.15.1 If [3], duration (month):
9.4.15.2 If [3], treatment:
[1] No (skip to 9.5) [2] Not clear(skip to 9.5) [3] Yes
9.4.15.2.1 If [3], treatment effect:
[1] Improved [2] Progressed [3] Stable [4] Not clear

9.5 Other procedures

9.5.1 Interdisciplinary consultation
[1] No (skip to 9.5.2) [2] Not clear(skip to 9.5.2) [3] Yes
9.5.1.1 Disciplines involved
[1] Neurology [2] Infectious diseases [3] Nephrology
[4] Endocrinology [5] Cardiovascular diseases
[6] Others (specify)
9.5.1.2 Total times of consultation:

9.5.2 Psychological/behavioral intervention
[1] No (skip to 9.5.3) [2] Not clear(skip to 9.5.3) [3] Yes
9.5.2.1 Type of interventions performed
[1] Neurology [2] Infectious diseases [3] Nephrology
[4] Endocrinology [5] Cardiovascular diseases
[6] Others (specify)
9.5.2.2 Total sessions of intervention performed:

9.5.3 Traditional Chinese medicine used
[1] No (skip to 10.1) [2] Not clear(skip to 10.1) [3] Yes
9.5.2.1 Regimen of TCM used (specify):

9.5.2.2 Duration of TCM use (days):

For peer review only

Part F: Charges on the inpatient care

10.1 Total inpatient care fee:
10.2 Registration fee
10.3 Bed fee
10.4 Examination fee
10.5 Treatment fee
10.6 Operation fee
10.7 Laboratory fee
10.8 Nursing fee
10.9 Medicines fee
10.10 Other fee

Name of data extractor:
Date of data extraction(dd-mm-yyyy): |__|__|-|__|__|-|__|__|__|__|

For peer review only

Annex 2: Lung cancer patient follow up interview Questionnaire

Reference Number: |__|__|-|__|__|__|__|__|
Patient identification number: |__|__|__|__|__|__|__|__|__|__|__|__|__|__|__|__|__|__|
Patient's relationship with the interviewee

[1] Patient himself/herself   [2] Spouse
[3] Parent   [4] Son/daughter
[5] Brother/sister   [6] Other (specify)

1. When were you (or was he/she) first diagnosed with lung cancer?
   Date of diagnosis (dd-mm-yyyy): |__|__|-|__|__|-|__|__|__|__|
2. Have you (or Has he/she) been hospitalized due to the lung cancer?
   [1] Yes   [2] No (skip to 3)   [3] Not clear (skip to 3)

2.1. If yes, please tell me, one-by-one, where and when were (or was) you (or he/she) hospitalized due to the lung cancer and how much it costed respectively.

No.	Name of hospital	Admission Date (mm-yyyy)	Total expenditure(RMB)
[1]			
[2]			
[3]			
[4]			
[5]			
[6]			
[7]			
[8]			
[9]			

(Please add more lines as necessary)

3. Have you (or Has he/she) sought medical checkups for monitoring development of the lung cancer?
   [1] Yes   [2] No (skip to 4)   [3] Not clear (skip to 4)

3.1. If yes, please tell me, one-by-one, where and when did the checkup happen and what were the findings respectively

No.	Name of hospital	Date checkup (mm-yyyy)	Reoccurrence	Metastasis
[1]				
[2]				
[3]				
[4]				
[5]				
[6]				
[7]				
[8]				
[9]				

(Please add more lines as necessary)

For peer review only

4. How are you (is he/she) now?

[1] Alive [2] Deceased

4.1. If [2], when did it happen (dd-mm-yyyy) ? |__|__|-|__|__|-|__|__|__|__|

5. In addition to the inpatient care and medical checkups mentioned above, have you (or has he/she) tried other measures to cure the lung cancer?

[1] Yes [2] No (skip to ending) [3] Not clear (skip to ending)

5.1. If yes, please tell me, one-by-one, what is it and how often it has/had been?

No.	Name of practice	Description of practice	Frequency	Length (months)
[1]				
[2]				
[3]				
[4]				
[5]				
[6]				
[7]				
[8]				
[9]				

(Please add more lines as necessary)

Name of data extractor:

Date of data extraction(dd-mm-yyyy): |__|__|-|__|__|-|__|__|__|__|:

For peer review only

BMJ Open

Pathways and cost-effectiveness of routine lung cancer inpatient care in rural Anhui, China: a retrospective cohort study protocol

Journal:	*BMJ Open*
Manuscript ID	bmjopen-2017-018519.R1
Article Type:	Protocol
Date Submitted by the Author:	05-Oct-2017
Complete List of Authors:	Shen, XingRong; Anhui Medical University Shcool of Health Service Management<br>Diao, MengJie; Anhui Medical University School of Health Service Management<br>Feng, Rui; Anhui Medical University, Library Department of Literature Retrieval and Analysis<br>Lu, ManMan ; Anhui Medical University School of Health Service Management<br>Zhang, PanPan ; Anhui Medical University School of Health Service Management<br>Jiang, Tao ; Anhui Medical University School of Health Service Management<br>Wang, DeBin; Anhui Medical University, School of Health Services Management
**Primary Subject Heading**:	Health economics
Secondary Subject Heading:	Health services research
Keywords:	cost effectiveness, lung cancer, inpatient care, retrospective study, China

SCHOLARONE™
Manuscripts

Pathways and cost-effectiveness of routine lung cancer inpatient care in rural Anhui, China: a retrospective cohort study protocol

XingRong Shen*, MengJie Diao*, Rui Feng, ManMan Lu, PanPan Zhang, Tao Jiang, Debin Wang[#]

Corresponding author: Debing Wang.

Post address: 81 Meishan Road, Hefei, Anhui, China. 230032.

Email: dbwang@vip.sina.com

Telephone: (0086) 5116395

XingRong Shen:

School of Health Service Management, Anhui Medical University, Hefei, Anhui, China.

MengJie Diao:

School of Health Service Management, Anhui Medical University, Hefei, Anhui, China.

Rui Feng:

Department of Literature Review and Analysis, Library of Anhui Medical University, Hefei, Anhui, China.

ManMan Lu:

School of Health Service Management, Anhui Medical University, Hefei, Anhui, China.

PanPan Zhang:

School of Health Service Management, Anhui Medical University, Hefei, Anhui, China.

Tao Jiang:

School of Health Service Management, Anhui Medical University, Hefei, Anhui, China.

Word count：3686

For peer review only

ABSTRACT

Introduction: Routine inpatient care (RIC) for cancer patients forms various pathways of clinical procedures. Although most of the individual procedures comprising the pathways have been tested via clinical trials, little is known about the collective cost-effectiveness of the pathways as a whole. This study aims at identifying pathways of RIC procedures for lung cancer patients from rural Anhui, China and examining determinants of the pathways and their links to cost-effectiveness.

Methods and analysis: The study adopts a retrospective cohort study design and proceeds in 5 steps. Step 1 defines 4 main categories of study variables including clinical procedures, direct cost and effectiveness of procedures, and factors affecting use of these procedures and their cost and effectiveness. Step 2 selects a cohort of 5000 lung cancer patients diagnosed between July 1, 2014 and June 30, 2015 from rural Anhui by clustered-random sampling. Step 3 retrieves the records of all the inpatient care episodes due to the lung cancer and extracts data about RIC procedures, proximate patient outcomes (e.g., Karnofsky performance status, lung function score) and related factors (e.g., stage of cancer, age, gender) by 2 independent clinician researchers using a pre-developed worksheet. Step 4 estimates the direct cost of each of the RIC procedures using micro-costing and collects data about ultimate patient outcomes (survival and progression-free survival) through a follow up survey of patients and/or their close relatives. Step 5 analyzes data collected and explores pathways of RIC procedures and their relations with patient outcomes, costs, cost-effect ratios and a whole range of clinical and socio-demographic factors using multivariate regression and path models.

Ethics and dissemination: The study protocol has been approved by authorized ethics committee. Findings from the study will be disseminated through conventional academic routes such as peer-reviewed publications and presentations and regional, national and international conferences.

Trial registry

ISRCTN25595562

Key words: cost effectiveness, lung cancer, inpatient care, retrospective study, China

For peer review only

Strengths and limitations of this study

- The study adopts a retrospective cohort study design involving a large representative sample of community patients;
- It evaluates cost-effectiveness of pathways of clinical procedures as a whole rather than individual procedures;
- It examines pathways of routine inpatient care for a huge but understudied Chinese rural population;
- It extracts data from routine records kept at different hospitals and thus suffers from discrepancies in performances and data qualities.

Introduction

Lung cancer has been the most common cancer in the world for several decades.[1] Estimated new cases of the disease was 1.8 million in 2012 (12.9% of the total), 58% of which occurred in less developed regions. Lung cancer was also the most common cause of death from cancer worldwide, being responsible for nearly one in five (1.59 million in absolute number) of the total. [2] In China, lung cancer incidence shows a slight decreasing trend in the past few years, particularly for males. However, it is still the top first cancer for males and second for females, accounting for 25.2% of all new cancer cases and 29.5% of all cancer deaths in 2012. [3]

Routine inpatient care (RIC) for lung cancer consists of a combination of procedures. Patients with possible lung cancer need a detailed history and physical examination first. Then they should undergo posterior-anterior and lateral chest radiographs as well as CT scans of the chest and abdomen. In order to further confirm and determine stage and histology of the lesion, other diagnostic methods needed include whole-body fluoro-deoxy-glucose positron emission tomography, endoscopic ultrasound, sputum cytology, fine-needle aspiration, bronchoscopy and others. Following diagnosis of lung cancer, the patients proceed with combined-modality therapies depending on stage of the disease and co-morbidity and complications. Historically, surgery provides the best chance for cure for patients whose lung cancers are limited to the hemithorax and can be totally encompassed by excision.[4 5]And surgery has been generally used in combination with external-beam radiotherapy for control of the primary tumor and regional lymphatics.[6] In addition, chemotherapy has also been advocated as an integral part of combined modality approaches to earlier stages of disease. [7 8] For unselected advanced none-small cell lung cancer, platinum-based combinations have become the standard of care; while cisplatin– or carboplatin-based doublets are standard for patients with stage IV disease.[9 10] More recently, EGFR tyrosine kinase inhibitors have been introduced in second- and third-line treatment of advanced disease and in first-line treatment for selected patients.[11]

Given the complex procedures, ensuring quality RIC for lung cancer patients has been most challenging and guidelines are widely used in addressing this challenge. Numerous

For peer review only

studies have documented positive relations between compliance with guidelines and patient outcomes.[12 13] However, researchers have also raised concerns about guidelines. One of such concerns refers to lack of adequate consideration of costs. Most clinical procedures not only affect disease outcomes but also incur considerable costs.[14 15] Yet guidelines are based on trials focused primarily on effectiveness (e.g., survival) with little attention being paid to economic consequences.[16] Another concern relates to incompatible population between clinical trials and RIC. Clinical trials on which guidelines are based use highly selected populations; while RIC serves a general lung cancer population with different age, performance status and comorbidities.[17 18] A third concern revolves uncertain interactions between procedures. Although most individual guideline recommended procedures (GRPs) have established evidences, they are not used in isolation but in conjunction with others forming various clinical combinations. Efforts systematically assessing and comparing these combinations are scarce.[19-22] A fourth concern originates from varied compliance with guidelines since RIC often deviates substantially from guidelines.[23 24] The cost-effectiveness of these "substandard" or mixed combinations of procedures (partly from guidelines, partly from experiences of individual clinicians) falls far from well-understood.[25] These all points to a clear need for evaluating RIC even though guidelines are widely available.

All the above mentioned concerns surrounding cancer care are most pertinent to China. First, China has a unique "dual" medical care system in which patients often receive western medicine and traditional Chinese medicine simultaneously or in turn.[26] Second, China lacks coordinated referral and follow up mechanisms and cancer patients often moves freely from one hospital to another for different rounds of inpatient care.[27] This makes it hard for clinicians in leveraging different inpatient care episodes at different time points and hospitals into continuous and synergetic service. Third, China has strong socio-cultural norms and financial incentives that hinder cost control and guideline compliance.[28]

Study aims

This study aims at identifying pathways of RIC procedures for lung cancer patients from rural Anhui, China and examining determinants of the pathways and their links to cost-effectiveness. Specific questions to be addressed include: a) what combinations of diagnosis and treatment procedures (or pathways for short) an individual patient may experience during all his/her hospitalization episodes due to lung cancer-related problems; b) what are the most and least frequent pathways; c) what determines the flow among these pathways; d) how cost-effective is each of the pathways; and e) what factors are associated with the cost-effectiveness.

The above "pathways" of inpatient care means combinations of diagnosis and treatment procedures an individual patient may experience during all his/her hospitalization

For peer review only

episodes due to lung cancer-related problems. Suppose a lung cancer patient experienced 6 times/rounds of hospitalized care and during each of these hospitalization episodes, the patient underwent several diagnosis and treatment procedures. Putting together, all these procedures form the "pathway" of this particular patient.

Methodology

Identification of procedures

The study uses a self-designed data extraction form in identifying major clinical procedures described in any RIC record under concern. The form lists all major RIC procedures under two main domains, i.e., diagnostic procedures (e.g., chest X-ray, chest CT, neck ultrasonography; Part D of supplementary file 1) and treatment procedures (e.g., surgical therapy, chemotherapy, psycho-behavioral intervention; Part E of supplementary file 1).

Estimation of costs

The study estimates overall and categorical costs (direct costs only) for each of the RIC procedures (e.g., lung function examination, computed tomography, white blood cell count) identified above using micro-costing techniques.[29 30]Taking the example of lung function examination, categorical costs include costs on personnel, equipment, materials, regents and others need in completing the examination; while overall cost of the procedure equals the sum of all these categorical costs. In addition, the study also calculates overall cost on individual inpatient by adding up the overall costs on all the clinical procedures he/she has received.

Measurement of effectiveness

The study uses both proximal variables of outcome (PV) and ultimate outcome (UO) measures of effectiveness of RIC procedures. The UO indicators derive from a follow up survey about 2 years and half after the first hospitalization and include survival, progression-free survival (PFS), quality of life, and quality adjusted life years (QALYs). Here, quality of life is assessed using the widely recognized EQ-5D instrument.[31]

The PV measures come from RIC records and include Eastern Cooperative Oncology Group (ECOG), Karnofsky performance status (KPS) and compiled scores of: a) symptoms (e.g., chronic cough, chest pain, wasting syndrome); b) lung functions (e.g., forced vital capacity, forced one second expiratory volume), c) image findings (e.g., number of nodules identified in the lung, size of the largest nodules, presence of pleura or pericardial effusion). Each of these domain specific PV scores equals weighted sum of all sub-indicators within the domain. For example, the compiled score of "lung functions" equals the sum of weighted values of forced vital capacity, forced one second expiratory volume etc. Here the weights come from the coefficients of multivariate regression modeling using an UO indicator (e.g., survival) as the dependent variable; while forced

For peer review only

vital capacity, forced one second expiratory volume etc. as the independent variables; and stage of disease, age, gender and others as the confounding variables.

Calculation of cost-effectiveness

The study adopts cost-effectiveness ratios (CERs) and relative cost-effectiveness ratio (RCERs) as the main indicators for measuring cost-effectiveness. Here RCER is defined by the difference in cost between two selected sets of RIC procedures, divided by the difference in their effect. More specifically, $\mathrm{RCER} = (\mathrm{C}_{\mathrm{r+x}} - \mathrm{C}_{\mathrm{r}})/(\mathrm{E}_{\mathrm{r+x}} - \mathrm{E}_{\mathrm{r}})$, where $\mathrm{C_r}$ and $\mathrm{E_r}$ is the cost and effect in the reference group and $\mathrm{C_{r+x}}$ and $\mathrm{E_{r+x}}$, the cost and effect in the group who have underwent all the procedures in the reference group plus x, a specific procedure under concern.[32] Suppose, x represents a commonly used traditional Chinese medicine (TCM) which incurs 100 dollars; while r, a typical combination of diagnosis and treatment procedures without the TCM. The combination costs 1000 dollars and the survival time of patients who have adopted this combination is 1.5 years on average; while the same figure for patients who have used the combination plus the TCM is 1.51. Then the $\mathrm{C_{r+x}} = 1000 + 100 = 1100$ dollars and the ICER of the TCM = (1100-100)/(1.51-1.5)=10000 dollars.

Identification of influencing factors

The study also extracts, from RIC records, data about patient factors commonly believed to be linked with disease progression, treatment response and outcomes and utilization of RIC procedures. These include: a) socio-demographics (e.g., age, gender, body height and weight, education, employment, marital status, medical insurance); b) risk behaviors and histories (e.g., smoking, alcohol drinking, history of cancer among family members); c) historical and biological test findings (e.g., value of ALK, KRAS, EGFR, PDL1, CEA, CA125, proGRP); d) comorbidities and complications (e.g., presence of superior vena cava syndrome, brain metastases) and stage of disease. Here, disease staging uses TNM system and this staging will be treated as the most important factor throughout the data analysis especially in its effects on the flow of different pathways and their cost-effectiveness.

Selection of participants

The study is implemented in Anhui, an inland province located in middle and east China. It has a population of 61.4 million and its per capita GDP and income rank in the middle (14th) among all provinces in the nation.[33 34] The social, cultural and economic background of Anhui is representative of over 80% of the whole population in China.[33 34] The province has 68 rural counties and each of them divides into 10 to 20 townships. Selection of participating counties, townships, patients and RIC case records uses a clustered random sampling which proceeds in 5 steps. Step 1 classifies all the counties in Anhui into southern, northern and middle areas. Step 2 randomly selects 3 counties from each of these areas (12 counties in total). Step 3 randomly draws 4 townships from each of the counties selected (48 townships in total). Step 4 searches the provincial

For peer review only

reimbursement database of the New Rural Cooperative Medical System (NRCMS) and identifies all the patients within the selected townships who had been first diagnosed with primary lung cancer during July 1, 2014 and June 30, 2015. Step 5 searches the database again for all episodes of hospitalization due to the lung cancer for the patients identified in step 4. NRCMS covers 98% of the rural residents and the estimated number of patients and admission episodes is about 5,000 and 25,000 respectively.

The above sample size was determined by our study purpose of building multivariate models of factors affecting the cost-effectiveness of specific routine inpatient care (RIC) pathways. Lung cancer patients generally receive 4 to 6 rounds of inpatient care. Given the various diagnostic and treatment procedures available, there are hundreds of potential RIC pathways (combinations of diagnosis and treatment procedures from the first to the last round of RIC). We plan to group these pathways into manageable (around 20) categories depending on the resultant distribution of the actual pathways and we aim to enter 20-30 factors into the cost-effectiveness model for each of these categorical pathways. Based on these pre-conditions and that the sample size of a multi-variable model should generally be 10 times the number of independent variables, we need 250 patients for each pathway. This translates into 5000 patients in total.

Data collection

The study obtains data through follow-up survey and data extraction. The follow-up survey applies to all the lung cancer patients identified above. It solicits information about the patient's: a) disease progression (i.e., died, alive with or without progression); b) if died, date of death; c) additional admissions due to the lung cancer not included in the above mentioned NRCMS database. The survey uses a short structured questionnaire (supplementary file 2). Administration of the questionnaire starts with a telephone interview (of the patient under concern or his/her close relatives for up to 5 time attempts) followed by a face-to-face interview (of the same respondents for up to 2 attempts) if the telephone contacts failed. The recruitment strives to reach over 85% rate of participation. And the researchers are trained to keep detailed record of reasons they lose some of the patients so as to allow for assessing potential biases. The data extraction applies to records of all the hospital admission episodes identified via the NRCMS database and the follow up survey. It uses a structured form (supplementary file 1) and extracts data about the clinical procedures, costs, effectiveness and influencing factors described above. Two experienced clinicians on care of lung cancer perform the data extraction. They visit (on one-by-one base) all the relevant hospitals, ask for permission to examine the full records and fill the worksheet independently first followed by discussions, if applicable, to solve discrepancies.

Data analysis

The data collected above allow a variety of descriptive and multivariate analysis centering on the effectiveness, costs and cost-effectiveness of RIC. The effectiveness

For peer review only

analysis comprises all the UO indicators mentioned above including progression free survival, overall survival, quality of life and DALYs. For each of these UO indicators, the analysis will produce: a) estimation of average rates or values with 95% confidence intervals at different time points after first diagnosis by disease stage, PV indicators, RIC pathways, non-hospital care categories, age range etc.; b) multivariate regression models using similar variables as independent variables; and c) path models using as disease stage, RIC pathways, non-hospital care categories, age range etc. as exogenous, complied PV indices as direct endogenous, and individual PV indicators as indirect endogenous variables (Figure 1a). Area under ROC (receiver operating characteristic) curve will be calculated for assessing the predictability of models using binary classifier as the dependent variable (e.g., models of progression free survival, overall survival).

The cost analysis explores mainly: a) overall and categorical costs on different rounds of hospitalization by socio-demographic and selected clinical conditions (Figure 2); b) scatter plot of RIC procedures using the occurrence rate and unit cost of individual procedures as the coordinates; c) multivariate regression models of overall and selected categorical costs using disease stage, PV indicators, RIC pathways, non-hospital care categories, age range etc. as independent variables; and d) Markov models of mean cost for managing lung cancer patients (Figure 1b).

The cost-effectiveness analysis focuses primarily on constructing a pathway tree to help identify the most and the least cost-effective pathways and estimate expected overall and pathway specific cost, effectiveness and cost-effectiveness ratios. The tree consists of different branches of combinations of RIC procedures starting from the first to the last episode of inpatient care labeled with estimated cost, effectiveness and cost-effectives ratios (CERs) (Figure 3). Relevance of the pathway tree is tested by means of, for instance, varying the percentage of patient flowing among the different pathways or the cost of major diagnostic and treatment procedures consisting the braches and then examine changes in the ranking of most or least cost-effective pathways. The analysis also pays particular attention to identifying as many as comparable pairs of RIC pathways as possible and calculating RCERs accordingly in a hope to uncover potential pathways with practice, policy and research implications.

The pathway tree construction will use TreeAge[35]; while the descriptive and multi-variate model analysis, SPSS 16. Cases with missing data about a specific item will be excluded from the analysis involving the item and where applicable, the statistical null hypothesis is be rejected at the significance level of $\alpha = 0.05$.

Ethics and dissemination

The study involves retrieving RIC records and recruiting patients or their relatives. So it adheres to rigorous human subject protection principles. The study protocol had been reviewed and approved by the Biomedical Ethics Committee of Anhui Medical

For peer review only - http://bmjopen.bmj.com/site/about/guidelines.xhtml

University (reference number: 20170312). Participation of hospitals, patients and their relatives are voluntary and written informed consent is required for all participants. Findings from the study will be disseminated through conventional academic routes such as peer-reviewed publications and presentations and regional, national and international conferences.

Discussion

The study would share the experience of lung cancer care from the rural Chinese perspective. It is an important sharing of knowledge on population-based lung cancer care, especially since most economic evidence comes from Europe and North America. As mentioned earlier in introduction, China has a unique clinical care system. In China, traditional Chinese medicine is used to complement or replace western medicine. This results in quite different pathways of lung cancer care that have seldom been well explored in published literatures. China has a long history of almost no charges being made for clinical consultations and most patients are used to paying only for medicines, laboratory tests and equipment-based examinations. This forms a perverse financial incentive for clinicians for ordering more sophisticated examinations and tests and for over prescribing. China's lack of referral and follow up mechanisms also merits particular attention. As an individual patient changes from one hospital (say for the first round of treatment) to another (for the second round treatment), he/she may receive different treatment regimens. Discontinued treatment and follow up may make it hard for clinicians to base their treatment decisions on observed effects.

Perhaps the most noteworthy findings of the current study may be the description of the pathways of RIC procedures and their links with cost-effectiveness (Figure 2). These pathways will provide easily understandable means for estimating and identifying, among others, the following: a) which pathways or combinations of procedures happen most or least in routine practice during different rounds of hospitalization for inpatients suffering from lung cancer in rural China; b) which pathways (from the first to last round of hospitalization) incur the highest or lowest direct costs; c) which pathways result in the best or worst patient outcome in terms of different PV and UO measures; d) which pathways are most or least cost-effective in terms of e.g., per unit cost gains in PFS, KPS, symptoms, lung functions, image findings, biological test findings, complications and comorbidities. These have important implications for clinical decision-making as well as policy-making.

Another point worth mentioning in particular refers to the links between the domain specific proximate outcome (PV) indices to key ultimate outcome (UO) indicators (e.g., survival) generated via a large scale (involving 5000 lung cancer patients) retrospective cohort study. They provide useful references for clinicians on care of lung cancer patients in selecting appropriate procedures to achieve optimal collective contributions to UO.[36]

For peer review only - http://bmjopen.bmj.com/site/about/guidelines.xhtml

At present, although PV indicators are observed routinely, they are presented to clinicians as individual indicators rather than compiled indices. And given the large number of PV indicators involved and the complex relations between RIC procedures and PV indicators and then UO indicators, it is difficult for practicing clinicians to make balanced decisions upon their personal experiences.[37]

In addition, this study addresses RIC for lung cancer at hospitals in China from a range of meaningful perspectives. The study reinforces the concepts introduced in the landmark studies of Fisher et al and Wennberg et al, which convincingly demonstrated that high quality was not necessarily associated with high cost.[38] Describing inpatient lung cancer care in a view that its value is directly proportional to outcomes and inversely proportional to costs helps in guiding quality improvement by either better outcomes and/or lower costs.[39] The study calculates and compares the collective cost-effectiveness of different RIC pathways as a whole and thus informs coordinated inpatient care episodes and procedures at different time points and hospitals. The study enables RCERs estimation for specific guideline recommended procedures (GRPs) using various combinations of real and uncontrollable RIC procedures as the reference and thus enhances understanding and application of GRPs established through well-controlled studies in routine practice contexts.

The study also has limitations. The first limit concerns data reliability. Although the majority of data will be extracted from RIC records kept at hospitals, the study uses self-reported data about quality of life and inpatient, outpatient and home care. Self-reports are prone to various biases including recall issues particularly among the elderly, over or under reporting by the respondents for reasons like perceived expectations from the researchers or for fearing of potential worries or distress. These biases may be reduced to a minimum in our study by means of interviewer training, use of chorological recall and probing techniques, and cross-checks of findings from patient interviews, health insurance database and hospital records. More importantly, the study uses EQ-5D in assessing quality of life. It has already been tested with adequate reliability both internationally and in China. Regarding non-hospitalized care, the study asks only simple questions about what kind of care the patients have experienced and when and for how long. These questions are relatively memorable and easily to answer. The second limit relates to selective study content. The study considers only inpatient care; while patients may use various self-treatment and outpatient treatment in addition to inpatient care.[40 41] And inpatient and non-inpatient treatment may substitute each other to some extent. These may result in under-estimation of the effectiveness of RIC procedures. Fortunately, this under-estimation may be offset to a large extent by treating non-hospital care as confounders and the study data to be collected allow this exercise. Third, the study considers only direct costs rather than full costs taking both direct and indirect costs into consideration. In addition, different hospitals use different equipment, reagents and medicines. Their quality of case records may also vary substantially. These raise

For peer review only

compatibility concerns in pooling data from different hospitals together and performing aggregate analysis. Finally, readers may raise concerns about representativeness of inpatients to the large cancer patients. Hospitalization rates documented from other countries vary greatly;[42] while similar data from China are scarce. Our estimation, using the dataset of the lasted province-wide Household Health Survey of Anhui, of the proportion of lung cancer patients who had been admitted to hospitals at least once was as high as 89%.[43]

Competing interests

The authors declare no competing interests.

Authors' contributions

XS and MD contributed equally in conceiving this project, facilitating protocol and instrument development, and drafting this manuscript. RF, ML, PZ and TJ are kore researchers for cost estimation, record extraction, follow up survey and data analysis respectively. DW provided expertise for overall design of the study, and revised and finalized the manuscript. All authors have read and approved the final submission.

Acknowledgements

Development of the primitive protocol was supported by the Natural Science Foundation of China (grant number: 81172201). Refinement and implementation of the protocol is lead and supported by Collaboration Center for Cancer Control of Anhui Medical University, Anhui and Luan Center for Diseases Control and Prevention.

References

1. McErlean A, Ginsberg MS. Epidemiology of lung cancer. *Semin Roentgenol* 2011;46 (3):173-7.
2. World Health Organization. GLOBOCAN 2012: Estimated Cancer Incidence, Mortality and Prevalence Worldwide in 2012. http://globocan.iarc.fr/Default.aspx.
3. Kong J, Xu F, He M, Chen K, et al. The incidence of lung cancer by histological type: a population-based study in Tianjin, China during 1981-2005.*Respirology* 2014;19(8):1222-8.
4. Woodard GA, Jablons DM. The Latest in Surgical Management of Stage IIIA Non-Small Cell Lung Cancer: Video-Assisted Thoracic Surgery and Tumor Molecular Profiling. *Am Soc Clin Oncol Educ Book* 2015;35:e435-41.
5. Grunenwald DH. The role of surgery in non-small-cell lung cancers. *Ann Oncol 2005*;16 Suppl 2:ii220-2.
6. Ricardi U, Badellino S, Filippi AR. Stereotactic radiotherapy for early stage non-small cell lung cancer. *Radiat Oncol J*. 2015;33(2):57-65.

For peer review only

7. Mangal S, Gao W, Li T1, Zhou QT. Pulmonary delivery of nanoparticle chemotherapy for the treatment of lung cancers: challenges and opportunities. *Acta Pharmacol Sin*. 2017. doi: 10.1038/aps.2017.34.
8. Khan I, Morris S, Hackshaw A, et al. Cost-effectiveness of first-line erlotinib in patients with advanced non-small-cell lung cancer unsuitable for chemotherapy. *BMJ Open* 2015; 5(7):e006733.
9. Garon EB, Ciuleanu TE, Arrieta O, et al. Ramucirumab plus docetaxel versus placebo plus docetaxel for second-line treatment of stage IV non-small-cell lung cancer after disease progression on platinum-based therapy (REVEL): a multicentre, double-blind, randomised phase 3 trial. *Lancet* 2014;384(9944):665-73.
10. Spigel DR, Luft A, Depenbrock H, et al. An Open-Label, Randomized, Controlled Phase II Study of Paclitaxel-Carboplatin Chemotherapy With Necitumumab Versus Paclitaxel-Carboplatin Alone in First-Line Treatment of Patients With Stage IV Squamous Non-Small-Cell Lung Cancer. *Clin Lung Cancer* 2017; pii: S1525-7304(17)30045-1.
11. Stinchcombe TE. The Use of EGFR Tyrosine Kinase Inhibitors in EGFR Wild-Type Non-Small-Cell Lung Cancer. *Curr Treat Options Oncol* 2016; 17(4):18.
12. Ahmed HZ, Liu Y, O'Connell K, et al. Guideline-concordant Care Improves Overall Survival for Locally Advanced Non-Small-cell Lung Carcinoma Patients: A National Cancer Database Analysis. *Clin Lung Cancer* 2017; pii: S1525-7304(17)30114-6.
13. Nadpara P, Madhavan SS, Tworek C. Guideline-concordant timely lung cancer care and prognosis among elderly patients in the United States: A population-based study. *Cancer Epidemiol*; 39(6):1136-44.
14. Hinde S, McKenna C, Whyte S, et al. Modelling the cost-effectiveness of public awareness campaigns for the early detection of non-small-cell lung cancer. *Br J Cancer* 2015; 113(1):135-41.
15. Kumar G, Woods B, Hess LM, et al. Cost-effectiveness of first-line induction and maintenance treatment sequences in non-squamous non-small cell lung cancer (NSCLC) in the U.S. *Lung Cancer* 2015. pii: S0169-5002(15)00281-0.
16. Warren JL, Harlan LC, Trimble EL, et al. Trends in the receipt of guideline care and survival for women with ovarian cancer: A population-based study. *Gynecol Oncol* 2017;145(3):486-492.
17. Jennens RR, Giles GG, Fox RM. Increasing underrepresentation of elderly patients with advanced colorectal or non-small-cell lung cancer in chemotherapy trials. *Int Med J* 2006;36: 216e220.
18. Murthy VH, Krumholtz HM, Gross CP. Participation in cancer clinical trials; race-, sex-, and age-based disparities. *JAMA* 2004;22(291): 2720-2726.
19. Tong Y, Huang C, Zhang J. A novel EGFR-TKI inhibitor (cAMP-H3BO3 complex) combined with thermal therapy is a promising strategy to improve lung cancer treatment outcomes. *Oncotarget.* Published Online First: 04 May 2017.doi: 10.18632/oncotarget.17628.
20. National Health and Family Planning Commission of China. Guideline for Chinese primary lung cancer diagnosis and treatment (2015 edition). *Chinese Journal of Oncology* 2015.37(1):67-78.
21. Xing Wang, Shi Yan, Yaqi Wang, et al.Surgical Quality Surveillance and Sustaining Improvement of Lung Cancer Surgery Based On Standard Operation Procedure(SOP) : Experience of Single Surgical Team. *Chinese Journal of Lung*

For peer review only

Cancer; 20(4): 253-258.
22. Jackman DM, Zhang Y, Dalby C, et al. Cost and Survival Analysis Before and After Implementation of Dana-Farber Clinical Pathways for Patients With Stage IV Non-Small-Cell Lung Cancer. *J Oncol Pract* 2017;13(4):e346-e352.
23. Okita A, Yamashita M, Abe K, et al. Variance analysis of a clinical pathway of video-assisted single lobectomy for lung cancer. *Surg Today* 2009;39(2):104-9.
24. Duggan KJ, Descallar J, Vinod SK. Application of Guideline Recommended Treatment in Routine Clinical Practice: A Population-based Study of Stage I-IIIB Non-small Cell Lung Cancer. *Clin Oncol (R Coll Radiol)* 2016;28(10):639-47.
25. Heins MJ, de Jong JD, Spronk I, et al. Adherence to cancer treatment guidelines: influence of general and cancer-specific guideline characteristics. *Eur J Public Health* 2016; pii: ckw234. doi: 10.1093/eurpub/ckw234.
26. Yang S, Cui M, Li HY, et al. Meta-analysis of the effectiveness of Chinese and Western integrative medicine on medium and advanced lung cancer. *Chin J Integr Med* 2012;18(11):862-7.
27. XX L, YW Chen, KS Bi. Resolution of Two- way Recerral Problem in China by Studying British National Health Service System. *Chinese General Practice* 2013; 31(16):2926-29.
28. Y Sun, J Wu, SB Xie, et al. Evaluation of the medical staff clinical pathway adherence: Based on comparison of before and after provider payment reform in Henan Province. *Chinese Journal of Health Policy* 2013;6(5):37-43.
29. Henry SG, Ness RM, Stiles RA, Shintani AK, Dittus RS. A cost analysis of colonoscopy using microcosting and time-and-motion techniques. *J Gen Intern Med* 2007, 22(10):1415-21.
30. Cressman S, Lam S, Tammemagi MC,et al. Resource utilization and costs during the initial years of lung cancer screening with computed tomography in Canada. *J Thorac Oncol* 2014, 9(10):1449-58.
31. Khan I,Morris S, Pashayan N, et al. Comparing the mapping between EQ-5D-5L, EQ-5D-3L and the EORTC-QLQ-C30 in non-small cell lung cancer patients. *Health Qual Life Outcomes* 2016, 14:60.
32. SN York. Incremental Cost-Effectiveness Ratio. *Handbook of Disease Burdens and Quality of Life Measures* 2010: 4235-4235.
33. National Bureau of Statistics of China. China Statistical Yearbook 2016. http://www.stats.gov.cn/tjsj/ndsj/2016/indexch.htm (accessed 1 July 2017).
34. Statistics Bureau of Anhui Province. Statistical yearbook of Anhui Province in 2016. http://www.ahtjj.gov.cn/tjj/web/tjnj_view.jsp?strColId=13787135717978521&_index=1# (accessed 1 July 2017).
35. Treeage: Hollman C, Paulden M, Pechlivanoglou P, et al. A Comparison of Four Software Programs for Implementing Decision Analytic Cost-Effectiveness Models. *Pharmacoeconomics* 2017. doi: 10.1007/s40273-017-0510-8.
36. Darling G, Malthaner R, Dickie J, et al. Quality indicators for non-small cell lung cancer operations with use of a modified Delphi consensus process. *Ann Thorac Surg* 2014;98(1):183-90.
37. Fisher A, Manicavasagar V, Sharpe L, et al. A Qualitative Exploration of Clinician Views and Experiences of Treatment Decision-Making in Bipolar II Disorder. *Community Ment Health J.* Published Online First: 19 Jan 2017. doi: 10.1007/s10597-016-0077-4.

38. Wennberg JE, Fisher ES. Finding high quality, efficient providers for value purchasing: cohort methods better than methods based on events. Finding high quality, efficient providers for value purchasing: cohort methods better than methods based on events. *Med Care* 2002,40(10):853-5.
39. Chen LW, Wilson FA, Gregg A, et al. Measuring the Cost and Value of Quality Improvement Initiatives for Local Health Departments. J Public Health Manag Pract. Published Online First: 1 Mar 2017. doi: 10.1097/PHH.0000000000000552.
40. O' Regan P, Hegarty J. The importance of self-care for fatigue amongst patients undergoing chemotherapy for primary cancer. *Eur J Oncol Nurs* 2017;28:47-55.
41. Dionne-Odom JN, Demark-Wahnefried W, Taylor RA, et al. The self-care practices of family caregivers of persons with poor prognosis cancer: differences by varying levels of caregiver well-being and preparedness. *Support Care Cancer* 2017; 25(8):2437-2444.
42. Prince RM, Atenafu EG, Krzyzanowska MK. Hospitalizations During Systemic Therapy for Metastatic Lung Cancer: A Systematic Review of Real World vs Clinical Trial Outcomes. JAMA Oncol 2015, 1(9):1333-9.
43. Zhao T, Cheng J, Chai J, et al. Inpatient care burden due to cancers in Anhui, China: a cross-sectional household survey. *BMC Public Health* 2016, 16:308.

For peer review only

Figure 1 Schematic structure of sample multivariate models to be built

Figure 2 Simulated cost by selected socio-demographics and clinical characteristics (TC=total cost; KRMB=1000 Chinese yuan)

Figure 3 Anticipated “procedure-outcome” tree of inpatient lung cancer care (Tx = the xth round of hospitalization; Cx = the xth combination of clinical procedures; Px = possibility of using the xth combinations of clinical procedures; Ox = the xth patient outcome index/indicator)

For peer review only

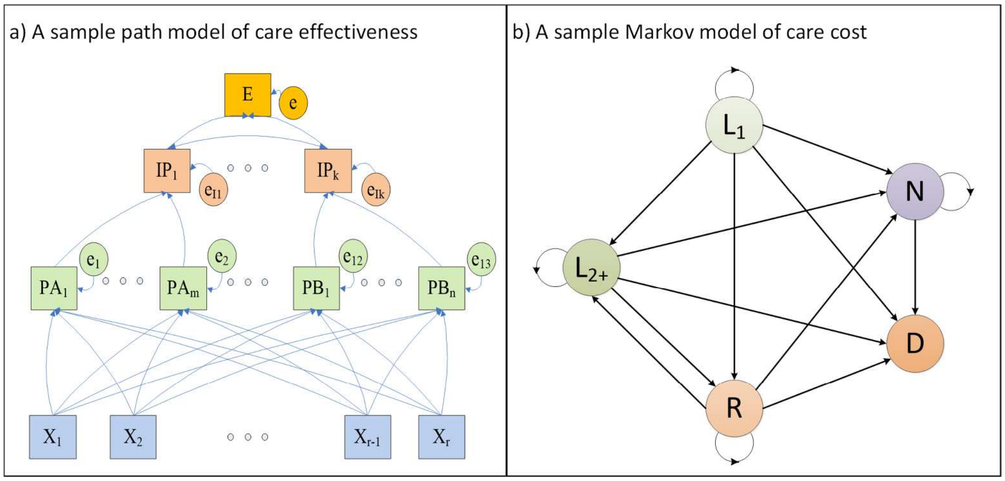

Figure 1 Schematic structure of sample multivariate models to be built/ X=independent variables; PA or PB=domain A or proximate indicators of effectiveness; IP=index of proximate variables; e=systematic error; and E= effectiveness, e.g., overall survival, QALYs; L_1=first line treatment; L_2+=second or third line treatment; R=remission; N=no active treatment; D=death.

188x88mm (300 x 300 DPI)

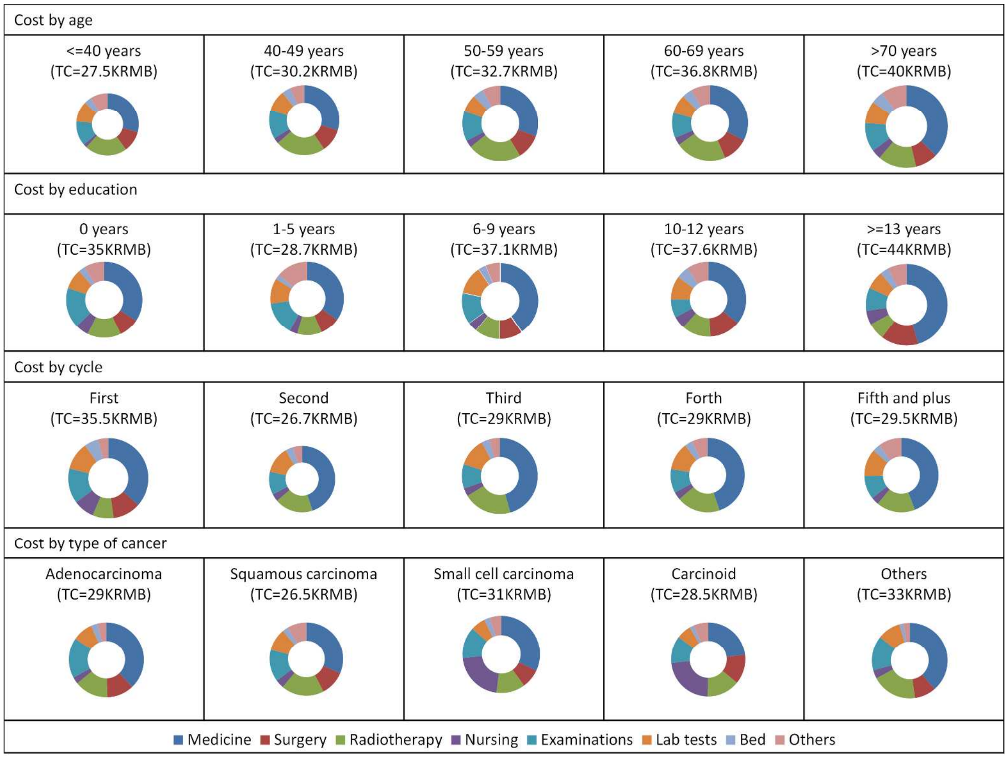

Figure 2 Simulated cost by selected socio-demographics and clinical characteristics (TC=total cost; KRMB=1000 Chinese yuan)

249x187mm (300 x 300 DPI)

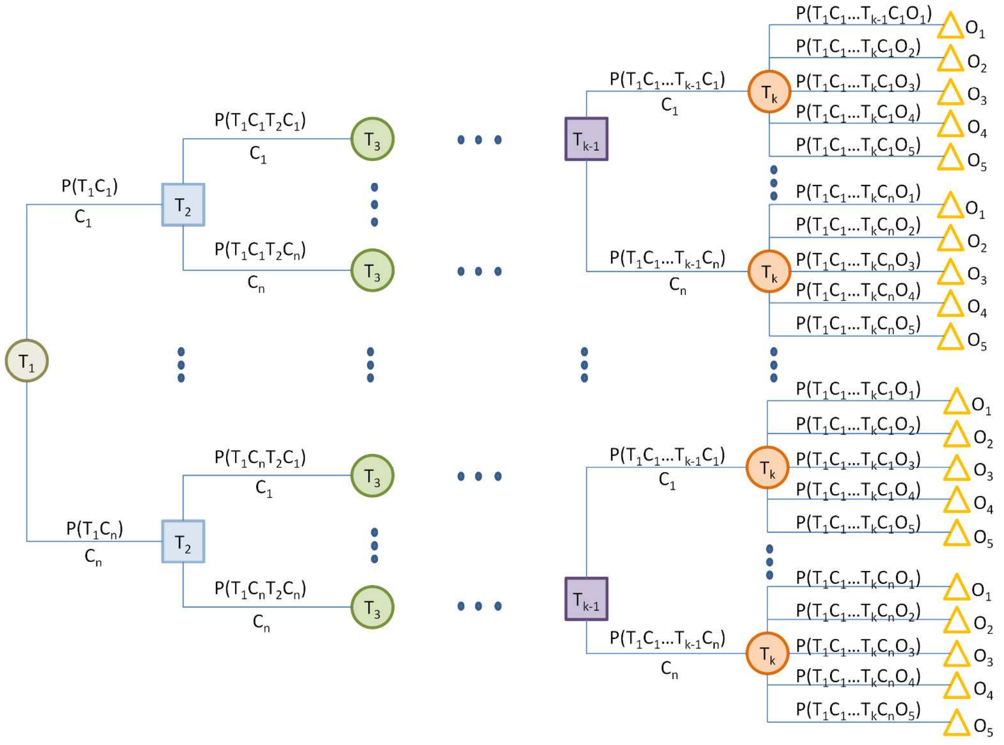

Figure 3 Anticipated “procedure-outcome” tree of inpatient lung cancer care (T_x = the xth round of hospitalization; C_x = the xth combination of clinical procedures; P_x = possibility of using the xth combinations of clinical procedures; O_x = the xth patient outcome index/indicator)

242x183mm (300 x 300 DPI)

Annex 1 Lung cancer inpatient care data extraction form

Reference Number: |__|__|-|__|__|__|__|__|

Part A: Patient's social demographics

1.1 Case record number: |__|__|__|__|__|__|__|__|
1.2 Patient identification number: |__|__|__|__|__|__|__|__|__|__|__|__|__|__|__|__|__|
1.3 Sex: [1]Male [2]Female
1.4 Birth date (dd-mm-yyyy, first case record only): |__|__|-|__|__|-|__|__|__|__|
1.5 Body height (centimeter, first case record only): |__|__|__|
1.6 Body weight (kilogram): |__|__|__|.|__|
1.7 Education (first case record only):
[1] No formal education [2] Primary school [3] Middle school
[4] High school [5] College [6] Graduate or higher
[7] Not clear
1.8 Occupation (first case record only):
[1] Staff of public entities [2] Employee of firms [3] Self-employed
[4] Peasant [5] Un-employed [6] Retired
[7] Army member [8]Not clear
1.9 Marital status:
[1] Unmarried [2] Married [3] Divorced
[4] Widowed [5] Other [6] Not clear
1.10 Medical insurance:
[1] Essential medical insurance for urban employees
[2] Medical insurance for urban citizens
[3] New rural cooperative medical care systems
[4] Commercial medical insurance
[5] Public medical care system
[6] Out-of-pocket care
[7] Other
[8] Not clear

Part B: Patient's behavior and disease history (first case record only)

2.1 Smoking:
[1] Current smoker [2] Former smoker [3] Non-smoker
[4] Smoker [9] Not clear (skip to 2.2)
2.1.1 Number of cigarettes smoked per day: |__|__|__|
2.1.2 Number of years smoked: |__|__|
2.1.3 Number of years ceased smoking: |__|__|

For peer review only

2.2 Previous diagnosis of the following respiratory diseases:

[1] Tuberculosis [2] Chronic bronchitis [3] Emphysema
[4] Asthma [5] Silicosis/pneumonoconiosis
[6] Other(specify)

2.3 Previous diagnosis of the following cardio-cerebrovascular/endocrine diseases:

[1] Hypertension [2] Coronary heart disease [3] Cerebral thrombosis
[4]Cerebral hemorrhage [5] Hyperlipemia [6] Diabetes
[7] Other(specify)

2.4 Previous diagnosis of cancer (enter location of cancer, if applicable, e.g., breast cancer, colorectal cancer)

[1] [2] [3]
[4] [5] [6]
[7] [8] [9]

(Please add more cells as needed)

2.5 Previous diagnosis of cancer among relatives

Number	Type of relatives	Location of cancer
[1]		
[2]		
[3]		

(Please add more rows as needed)

Part C: Patient's current symptoms/sings

3.1 Respiratory symptoms/signs

[1] Chronic coughing [2] Sputum with blood [3] Chest suppression
[4] Chest pain [5] Difficult breathing [6] Repeated bronchitis
[7] Hoarseness [8]Other (specify)
[9] None

3.2 Symptoms/signs of metabolism or immunity dysfunction:

[1] None [2] Hippocratic fingers/toes [3] Amyasthenia
[4] Hyponatremia [5] Blacken skin folds
[6] Other (specify)

3.3 Symptoms/signs relating to lung cancer metastasis:

[1] None [2] Topical pain [3] Headache
[4] Dizzy [5] Sudden dyskinesia [6] Facial swelling
[7] Other (specify)

3.4 Cancer-related non-specific symptoms/signs:

[1] None [2] Apparent emaciation [3] Weakness
[4] Mild/moderate fever [5] Other (specify)

3.5 Karnofsky score:

[1] |__|__|__|
[2] Not available

3.6 Body surface examination findings:

[1] None

For peer review only

[2] Enlargement of lymph nodes in the neck or supraclavicular region
[3] Lymph node enlargement in other areas
[4] Subcutaneous nodule
[5] Horner syndrome
[6] Facial swelling
[7] Other (specify)
[9] Not clear

Part D: Diagnostic procedures and findings

a) Imaging diagnosis

4a.1 Chest X-ray examination:
[1] Not performed (skip to 4a.2)
[2] Performed
4a.1.1 Date of performance (dd-mm-yyyy): |__|__|-|__|__|-|__|__|__|__|
4a.1.2 Abnormalities iddentified
[1] None
[2] Pulmonary nodules/mass
[3] Hilar / mediastinal abnormalities
[4] Pleural effusion
[5]Pericardial effusion
[6] Other (specify)
4a.1.2.1 If [2], please specify the largest nodules/mass: |__|__|.|__|*|__|__|.|__|cm
4a.2 Chest CT examination:
[1] Not performed (skip to 4a.3)
[2] Performed
4a.2.1 Date of performance (dd-mm-yyyy): |__|__|-|__|__|-|__|__|__|__|
4a.2.2 Type of CT performed
[1] Plain [2] Enhanced scan [3] Plain + enhanced
4a.2.3 Layer thickness: |__|__|.|__|cm
4a.2.4 Multiple plane reconstruction (MPR):
[1] Yes [2] No
4a.2.5 Locations scanned
[1] Chest [2] Chest and abdomen [3] Neck and chest
[4] Neck+chest+abdomen
4a.2.6 Abnormalities identified
4a.2.6.1 Diagnosis from chest CT
[1] No abnormalities [2] Affirmative benign [3] Suspected benign
[4] Suspected malignant [5] Affirmative malignant
[6] Others (specify)
[9] Not clear
4a.2.6.2 Abnormalities identified

For peer review only

[1] Pneumonia [2] Bronchial abnormality [3] Single nodules/mass
[4] Multiple nodules/mass [5] Pleural effusion [6] Pericardial effusion
[7] Other (specify)
4a.2.6.2.1 If [3] or[4], size of the largest nodules/mass: |__|__|.|__|*|__|__|.|__|cm
4a.3 Head CT examination:
[1] Not performed (skip to 4a.4)
[2] Performed
4a.3.1 Date of performance (dd-mm-yyyy): |__|__|-|__|__|-|__|__|__|__|
4a.3.2 Type of CT performed
[1] Plain [2] Enhanced scan [3] Plain + enhanced
4.3.3 Diagnosis from head CT
[1] No abnormalities [2] Confirmed/suspected brain metastases
[3] Others (specify)
4a.4 Head MR examination
[1] Not performed (skip to 4a.5)
[2] Performed
4a.4.1 Date of performance (dd-mm-yyyy): |__|__|-|__|__|-|__|__|__|__|
4a.4.2 Diagnosis from head MR
[1] No abnormalities [2] Single brain metastases [3] Multiple brain metastases
[4] Others (specify)
4a.4.2.1 If [2] or [3], size of the largest nodules/mass: |__|__|.|__|*|__|__|.|__|cm
4a.5 Chest MR examination
[1] Not performed (skip to 4a.6)
[2] Performed
4a.5.1 Date of performance (dd-mm-yyyy): |__|__|-|__|__|-|__|__|__|__|
4a.5.2 Diagnosis from chest MR
[1] No abnormalities [2] Hilar/mediastinal lymph nodes [3] Lung nodules/mass
[4] Bone metastases [5] Thoracic/pericardial effusion
[6] Others (specify)
4a.5.2.1 If [3], size of the largest nodules/mass: |__|__|.|__|*|__|__|.|__|cm
4a.5.2.2 If [4], location metastases
4a.6 Bone MR examination
[1] Not performed (skip to 4a.7)
[2] Performed
4a.6.1 Date of performance (dd-mm-yyyy): |__|__|-|__|__|-|__|__|__|__|
4a.6.2 Diagnosis from bone MR
[1] No abnormalities [2] Bone metastases
[3] Others (specify)
4a.6.2.1 If [2], location of metastases
4a.7 Neck ultrasonography
[1] Not performed (skip to 4a.8)
[2] Performed
4a.7.1 Date of performance (dd-mm-yyyy): |__|__|-|__|__|-|__|__|__|__|
4a.7.2 Diagnosis from neck ultrasonography

For peer review only

[1] No abnormalities [2] Neck /supraclavicular lymph nodes
[3] Others (specify)

4a.8 Chest ultrasonography
[1] Not performed (skip to 4a.9)
[2] Performed
4a.8.1 Date of performance (dd-mm-yyyy): |__|__|-|__|__|-|__|__|__|__|
4a.8.2 Diagnosis from chest ultrasonography
[1] No abnormalities [2] Pleural effusion [3] Pericardial effusion
[4] Others (specify)
4a.9 Abdominal ultrasonography
[1] Not performed (skip to 4a.10)
[2] Performed
4a.9.1 Date of performance (dd-mm-yyyy): |__|__|-|__|__|-|__|__|__|__|
4a.9.2 Diagnosis from abdominal ultrasonography
[1] No abnormalities [2] Liver metastases [3] Adrenal gland transfer
[4] Peritoneal/retroperitoneal lymphadenopathy
[5] Others (specify)
4a.10 Bone scans
[1] Not performed (skip to 4a.11)
[2] Performed
4a.10.1 Date of performance (dd-mm-yyyy): |__|__|-|__|__|-|__|__|__|__|
4a.10.2 Diagnosis from bone scans
[1] No abnormalities [2] confirmed metastases [3] Suspected metastases
[4]Others (specify)
4a.10.2.1 If [2] or [3], location of metastases
4a.11 PET-CT examination
[1] Not performed (skip to 4b.1)
[2] Performed
4a.11.1 Date of performance (dd-mm-yyyy): |__|__|-|__|__|-|__|__|__|__|
4a.11.2 Diagnosis from PET-CT examination
[1] No abnormalities [2] Lung nodules/mass(Primary lesion)
[3] Pulmonary metastasis [4] Lymph node metastasis
[5] Adrenal gland transfer [6] Bone transfer
[7] Other site transfer [8] Thoracic / pericardial effusion
[9] Others (specify)
4a.11.3.1 If [2], location of lung nodules/mass
4a.11.3.1.1 Size of the largest nodules/mass: |__|__|.|__|*|__|__|.|__|cm
4a.11.3.1.2 SUV
4a.11.3.1.3 Nature of the nodules/mass identified:
[1] Affirmative benign [2] Suspected benign [3] Suspected malignant
[4] Affirmative malignant [5] Not clear [6] Others (specify)
4a.11.3.2 If [3], location of pulmonary metastasis
4a.11.3.2.1 SUV

For peer review only

4a.11.3.3 If [4], location of lymph node metastasis
4a.11.3.3.1SUV
4a.11.3.4 If [5], location of adrenal gland metastasis
4a.11.3.4.1SUV
4a.11.3.5 If [6], location of bone metastases
4a.11.3.5.1 SUV
4a.11.3.6 If [7], location of other metastases
4a.11.3.6.1 SUV

b) Endoscopic examinations

4b.1 Fiberoptic bronchoscopy
[1] Not performed (skip to 4b.2)
[2] Performed
4b.1.1 Date of performance (dd-mm-yyyy): |__|__|-|__|__|-|__|__|__|__|
4b.1.2 Diagnosis from fiberoptic bronchoscopy
[1] No abnormalities [2] Tumor
[3] Others (specify)
[4] Not clear
4b.2 Lavage cytology/brushing
[1] Not performed (skip to 4b.3)
[2] Not clear (skip to 4b.3)
[3] Performed
4b.2.1 Date of performance (dd-mm-yyyy): |__|__|-|__|__|-|__|__|__|__|
4b.3 Bronchoscopy clamp biopsy
[1] Not performed (skip to 4b.4)
[2] Not clear (skip to 4b.4)
[3] Performed
4b.3.1 Date of performance (dd-mm-yyyy): |__|__|-|__|__|-|__|__|__|__|
4b.4 Bronchoscopy aspiration biopsy
[1] Not performed (skip to 4c.0)
[2] Not clear (skip to 4c.0)
[3] Performed
4b.4.1 Date of performance (dd-mm-yyyy): |__|__|-|__|__|-|__|__|__|__|
4b.4.2 Type of bronchoscopy aspiration biopsy
[1] Endobroncheal ultrasonography [2] Electromagnetic-guided
[3] Transbronchial needle aspiration [4] Not clear
[5] Others (specify)

For peer review only

c) Laboratory/biological tests

4c.0 Date of performance (dd-mm-yyyy): |__|__|-|__|__|-|__|__|__|__|

4c.1 CEA

[1] Not performed (skip to 4c.2)
[2] Not clear (skip to 4c.2)
[3] Performed
4c.1.1 Date of performance if different from 4c.0
(dd-mm-yyyy): |__|__|-|__|__|-|__|__|__|__|
4c.1.2 Test result (value-unit):______-______

4c.2 CA125

[1] Not performed (skip to 4c.3)
[2] Not clear (skip to 4c.3)
[3] Performed
4c.2.1 Date of performance if different from 6.0
(dd-mm-yyyy): |__|__|-|__|__|-|__|__|__|__|
4c.2.2 Test result (value-unit):______-______

4c.3 proGRP

[1] Not performed (skip to 4c.4)
[2] Not clear (skip to 4c.4)
[3] Performed
4c.3.1 Date of performance if different from 6.0
(dd-mm-yyyy): |__|__|-|__|__|-|__|__|__|__|
4c.3.2 Test result (value-unit):______-______

4c.4 SCC

[1] Not performed (skip to 4c.5)
[2] Not clear (skip to 4c.5)
[3] Performed
4c.4.1 Date of performance if different from 6.0
(dd-mm-yyyy): |__|__|-|__|__|-|__|__|__|__|
4c.4.2 Test result (value-unit):______-______

4c.5 NSE

[1] Not performed (skip to 4c.6)
[2] Not clear (skip to 4c.6)
[3] Performed
4c.5.1 Date of performance if different from 6.0
(dd-mm-yyyy): |__|__|-|__|__|-|__|__|__|__|
4c.5.2 Test result (value-unit):______-______

4c.6 CYFRA21-1

[1] Not performed (skip to 4c.7)
[2] Not clear (skip to 4c.7)
[3] Performed
4c.6.1 Date of performance if different from 6.0

For peer review only

(dd-mm-yyyy): |__|__|-|__|__|-|__|__|__|__|
4c.6.2 Test result (value-unit):______-______
4c.7 WBC
[1] Not performed (skip to 4c.8)
[2] Not clear (skip to 4c.8)
[3] Performed
4c.7.1 Date of performance if different from 6.0
(dd-mm-yyyy): |__|__|-|__|__|-|__|__|__|__|
4c.7.2 Test result (value-unit):______-______
4c.8 PLT
[1] Not performed (skip to 4c.9)
[2] Not clear (skip to 4c.9)
[3] Performed
4c.8.1 Date of performance if different from 6.0
(dd-mm-yyyy): |__|__|-|__|__|-|__|__|__|__|
4c.8.2 Test result (value-unit):______-______
4c.9 Hb
[1] Not performed (skip to 4c.10)
[2] Not clear (skip to 4c.10)
[3] Performed
4c.9.1 Date of performance if different from 6.0
(dd-mm-yyyy): |__|__|-|__|__|-|__|__|__|__|
4c.9.2 Test result (value-unit):______-______
4c.10 ALB
[1] Not performed (skip to 4c.11)
[2] Not clear (skip to 4c.11)
[3] Performed
4c.10.1 Date of performance if different from 6.0
(dd-mm-yyyy): |__|__|-|__|__|-|__|__|__|__|
4c.10.2 Test result (value-unit):______-______
4c.11 Pre-ALB
[1] Not performed (skip to 4c.12)
[2] Not clear (skip to 4c.12)
[3] Performed
4c.11.1 Date of performance if different from 6.0
(dd-mm-yyyy): |__|__|-|__|__|-|__|__|__|__|
4c.11.2 Test result (value-unit):______-______
4c.12 Ca
[1] Not performed (skip to 4c.13)
[2] Not clear (skip to 4c.13)
[3] Performed
4c.12.1 Date of performance if different from 6.0
(dd-mm-yyyy): |__|__|-|__|__|-|__|__|__|__|
4c.12.2 Test result (value-unit):______-______

For peer review only

4c.13 Fe
[1] Not performed (skip to 4c.14)
[2] Not clear (skip to 4c.14)
[3] Performed
4c.13.1 Date of performance if different from 6.0
(dd-mm-yyyy): |__|__|-|__|__|-|__|__|__|__|
4c.13.2 Test result (value-unit):______-______

4c.14 FIB
[1] Not performed (skip to 4c.15)
[2] Not clear (skip to 4c.15)
[3] Performed
4c.14.1 Date of performance if different from 6.0
(dd-mm-yyyy): |__|__|-|__|__|-|__|__|__|__|
4c.14.2 Test result (value-unit):______-______

4c.15 D-D
[1] Not performed (skip to 4c.16)
[2] Not clear (skip to 4c.16)
[3] Performed
4c.15.1 Date of performance if different from 6.0
(dd-mm-yyyy): |__|__|-|__|__|-|__|__|__|__|
4c.15.2 Test result (value-unit):______-______

4c.16 Na
[1] Not performed (skip to 4c.17)
[2] Not clear (skip to 4c.17)
[3] Performed
4c.16.1 Date of performance if different from 6.0
(dd-mm-yyyy): |__|__|-|__|__|-|__|__|__|__|
4c.16.2 Test result (value-unit):______-______

4c.17 LDL
[1] Not performed (skip to 4c.18)
[2] Not clear (skip to 4c.18)
[3] Performed
4c.17.1 Date of performance if different from 6.0
(dd-mm-yyyy): |__|__|-|__|__|-|__|__|__|__|
4c.17.2 Test result (value-unit):______-______

4c.18 LDL
[1] Not performed (skip to 4c.19)
[2] Not clear (skip to 4c.19)
[3] Performed
4c.18.1 Date of performance if different from 6.0
(dd-mm-yyyy): |__|__|-|__|__|-|__|__|__|__|
4c.18.2 Test result (value-unit):______-______

For peer review only

4c.19 TG
[1] Not performed (skip to 4c.20)
[2] Not clear (skip to 4c.20)
[3] Performed
4c.19.1 Date of performance if different from 6.0
(dd-mm-yyyy): |__|__|-|__|__|-|__|__|__|__|
4c.19.2 Test result (value-unit):______-______
4c.20 TCHOL
[1] Not performed (skip to 4d.1)
[2] Not clear (skip to 4d.1)
[3] Performed
4c.20.1 Date of performance if different from 6.0
(dd-mm-yyyy): |__|__|-|__|__|-|__|__|__|__|
4c.20.2 Test result (value-unit):______-______

d) Heart and lung function examinations

4d.1 Electrocardiogram examination
[1] Not performed (skip to 4d.2)
[2] Performed
4d.1.1 Date of performance (dd-mm-yyyy): |__|__|-|__|__|-|__|__|__|__|
4d.1.2 Heart rate: |__|__|__| times/minutes
4d.1.3 Diagnosis from electrocardiogram examination
[1] No abnormalities
[2] Abnormalities(specify)
4d.2 Lung function examinations
[1] Not performed (skip to 4e.1)
[2] Not clear (skip to 4e.1)
[3] Performed
4d.2.1 Date of performance (dd-mm-yyyy): |__|__|-|__|__|-|__|__|__|__|
4d.2.2 FVC (Tested/predicted value): /
4d.2.3 FEV1(Tested/predicted value): /
4d.2.4 FEV1/FVC%(Tested/predicted value): /
4d.2.5 TLCO SB(Tested/predicted value): /
4d.2.6 Ventilation function assessment:
[1] No abnormalities [2] Mildly reduced [3] Moderately reduced
[4] Severely reduced [5] Restrictive [6] Obstruction
[7] Mixed [8] Not clear
4d.2.7 Lung capacity
[1] No abnormalities [2] Increased total residue ratio [3] Low lung capacity
[4] Not clear
4d.2.8 Breath diffusion
[1] No abnormalities [2] Reduced [3] Not clear

For peer review only

e) Histological/cytological examination

4e.1 Preoperative cytological
[1] Not performed (skip to 4e.2)
[2] Not clear (skip to 4e.2)
[3] Performed
4e.1.1 If [3], preoperative cytological method:
[1] Needle biopsy [2] Sputum specimen examination [3] Bronchial lavage
[4] Others (specify)
4e.1.2 If [3], preoperative cytological result:
[1] With cancer cells [2] Without cancer cells [3] Uncertain lesion
[4] Not clear
4e.1.2.1 If '4e.1.2' selected [1], cytological type
[1] Adenocarcinoma [2] Squamous cell carcinoma
[3] Small cell carcinoma [4] Carcinoid
[5] Large cell carcinoma [6] Squamous cell carcinoma
[7] Sarcomatoid carcinoma [8] carcinoma from sialaden
[9] Not clear [10] Others (specify)
4e.1.2.1.1 If '4e.1.2.1' selected [1], first class subtype code
[1] Pre-invasion lesion [2] Microinvasive adenocarcinoma
[3] Invasive adenocarcinoma [4] Variant invasive adenocarcinoma
[5] Others (specify)
[6] Not clear
4e.1.2.1.1.1 If '4e.1.2.1.1' selected [1], second class subtype code
[1] Atypical adenocarcinoma like hyperplasia
[2] Adenocarcinoma in situ
[3] Not clear
4e.1.2.1.1.2 If '4e.1.2.1.1' selected [3], second class subtype code
[1] Accumbens dominated [2] Acinar dominated
[3] Papillary dominated [4] Micro papillae dominated
[5] Entities with mucus dominated
[6] Not clear
4e.1.2.1.1.3 If '4e.1.2.1.1' selected [4], second class subtype code
[1] Mucinous invasive adenocarcinoma
[2] Colloid [3] Fetal
[4] Intestinal [5] Others (specify)
[6] Not clear

4e.2 Preoperative histological
[1] Not performed (skip to 4e.3)
[2] Not clear (skip to 4e.3)
[3] Performed
4e.2.1 If [3], method of preoperative histological biopsy:
[1] Ultrasound guided aspiration biopsy [2] CT guided aspiration biopsy

For peer review only

[3] Bronchoscopic biopsy [4] Nuclear magnetic puncture
[5] Not clear [6] Others (specify)

4e.2.2 If [3], results of preoperative histological biopsy:
[1] With cancer cells [2] Without cancer cells [3] Uncertain lesion
[4] Not clear

4e.2.2.1 If [1], histological type:
[1] Adenocarcinoma [2] Squamous cell carcinoma
[3] Small cell carcinoma [4] Carcinoid
[5] Large cell carcinoma [6] Squamous cell carcinoma
[7] Sarcomatoid carcinoma [8] carcinoma from sialaden
[9] Not clear [10] Others (specify)

4e.2.2.1.1 If '4e.2.2.1' selected [1], first class subtype code
[1] Pre-invasion lesion [2] Microinvasive adenocarcinoma
[3] Invasive adenocarcinoma [4] Variant invasive adenocarcinoma
[5] Others (specify)
[6] Not clear

4e.2.2.1.1 If '4e.2.2.1.1' selected [1], second class subtype code
[1] Atypical adenocarcinoma like hyperplasia
[2] Adenocarcinoma in situ
[3] Not clear

4e.2.2.1.1.2 If '4e.2.2.1.1' selected [3], second class subtype code
[1] Accumbens dominated [2] Acinar dominated
[3] Papillary dominated [4] Micro papillae dominated
[5] Entities with mucus dominated
[6] Not clear

4e.2.2.1.1.3 If '4e.2.2.1.1' selected [4], second class subtype code
[1] Mucinous invasive adenocarcinoma
[2] Colloid [3] Fetal
[4] Intestinal [5] Others (specify)
[6] Not clear

4e.2.2.2 If 4e.2.2.1 information not available, please tick in histology type:
[1] Small cell lung cancer [2] Non-small cell lung cancer [3] Benign lesion
[4] Not clear [5] Others (specify)

4e.3 Intraoperative biopsy of frozen mass:
[1] Not performed (skip to 4e.4)
[2] Not clear (skip to 4e.4)
[3] Performed

4e.3.1 If [3], diagnosis of frozen mass biopsy:
[1] Adenocarcinoma [2] Squamous cell carcinoma
[3] Small cell carcinoma [4] Carcinoid
[5] Large cell carcinoma [6] Squamous cell carcinoma
[7] Sarcomatoid carcinoma [8] carcinoma from sialaden
[9] Not clear [10] Others (specify)

4e.3.1.1 If '4e.3.1' selected [1], first class subtype code

For peer review only

[1] Pre-invasion lesion [2] Micro invasive adenocarcinoma
[3] Invasive adenocarcinoma [4] Variant invasive adenocarcinoma
[5] Others (specify)
[6] Not clear

4e.3.1.1.1 If '4e.3.1.1' selected [1], second class subtype code
[1] Atypical adenocarcinoma like hyperplasia
[2] Adenocarcinoma in situ
[3] Not clear

4e.3.1.1.2 If '4e.3.1.1' selected [3], second class subtype code
[1] Accumbens dominated [2] Acinar dominated
[3] Papillary dominated [4] Micro papillae dominated
[5] Entities with mucus dominated
[6] Not clear

4e.3.1.1.3 If '4e.3.1.1' selected [4], second class subtype code
[1] Mucinous invasive adenocarcinoma
[2] Colloid [3] Fetal
[4] Intestinal [5] Others (specify)
[6] Not clear

4e.4 Intraoperative biopsy of lymph node:
[1] Not performed (skip to 4e.5)
[2] Not clear (skip to 4e.5)
[3] Performed

4e.4.1 If [3], result of lymph node biopsy:
[1] Metastasis [2] No metastasis

4e.5 Intraoperative biopsy of frozen margin of bronchus:
[1] Not performed (skip to 4e.6)
[2] Not clear (skip to 4e.6)
[3] Performed

4e.5.1 If [3], result of frozen margin of bronchus:
[1] Margin tumor [2] No margin tumor

4e.6 Postoperative histological
[1] Not performed (skip to 4e.7.1)
[2] Not clear (skip to 4e.7.1)
[3] Performed

4e.6.1 If [3], number of tumors:
[1] Solitary tumor [2] More than 2 nodules [3] Not clear

4e.6.1.1 The largest tumor size: |__|__|*|__|__|*|__|__|cm

4e.6.1.2 If multiple tumor, the smallest tumor size: |__|__|*|__|__|*|__|__|cm

4e.6.2 Pathologic diagnosis
[1] Adenocarcinoma [2] Squamous cell carcinoma
[3] Small cell carcinoma [4] Carcinoid
[5] Large cell carcinoma [6] Squamous cell carcinoma
[7] Sarcomatoid carcinoma [8] carcinoma from sialaden

For peer review only

[9] Not clear [10] Others (specify)

4e.6.2.1 If '4e.6.2' selected [1], first class subtype code

[1] Pre-invasion lesion [2] Micro invasive adenocarcinoma

[3] Invasive adenocarcinoma [4] Variant invasive adenocarcinoma

[5] Others (specify)

[6] Not clear

4e.6.2.1.1 If '4e.6.2.1' selected [1], second class subtype code

[1] Atypical adenocarcinoma like hyperplasia

[2] Adenocarcinoma in situ

[6] Not clear

4e.6.2.1.2 If '4e.6.2.1' selected [3], second class subtype code

[1] Accumbens dominated [2] Acinar dominated

[3] Papillary dominated [4] Micro papillae dominated

[5] Entities with mucus dominated

[6] Not clear

4e.6.2.1.3 If '4e.6.2.1' selected [4], second class subtype code

[1] Mucinous invasive adenocarcinoma

[2] Colloid [3] Fetal

[4] Intestinal [5] Others (specify)

[6] Not clear

4e.6.3 Differentiation degree:

[1] Well differentiated [2] Well and moderately differentiated

[3] Moderately differentiated [4] Poorly differentiated

[5] Middle and low differentiation [6] Undifferentiated

[7] Not clear

4e.6.4 Associated with intrapulmonary metastasis

[1] Yes [2] No (skip to 4e.6.9) [3] Not clear(skip to 4e.6.9)

4e.6.5 Invasion of pleura?

[1] Yes [2] No [3] Not clear

4e.6.6 Invasion of the main bronchi?

[1] Yes, distance is less than 2cm [2] Yes, distance is more than 2cm

[3] No [3] Not clear

4e.6.7 Invasion of chest wall/septum/mediastinum/pericardium?

[1] Yes(specify) [2] No [3] Not clear

4e.6.8 Invasion of mediastinum/heart/trachea/esophagus/vertebral body/carina?

[1] Yes(specify) [2] No [3] Not clear

4e.6.9 Resection margin positive?

[1] Not performed (skip to 4e.6.10)

[2] Not clear (skip to 4e.6.10)

[3] Positive

[4] Negative

4e.6.10 The total number of lymph nodes detected

4e.6.11 The total number of lymph node metastasis

4e.6.12 Lymph node metastasis site

For peer review only

[1] No metastasis [2] Ipsilateral bronchi or hilum
[3] Ipsilateral mediastinum or carina
[4] Contralateral mediastinum or hilum of lung, clavicle
[5] Not clear

4e.7 Tumor maker
4e.7.1 Her-2(C-erbB-2) detection
[1] Not performed (skip to 4e.7.2)
[2] Not clear (skip to 4e.7.2)
[3] Performed (skip to 4e.7.2)
4e.7.1.1 If [3], method of detection
[1] Immunohistochemistry [2] FISH [3] Other(Specify)
4e.7.1.2 If [3], result of detection
[1] Positive [2] Negative [3] Other(Specify) [4] Not clear
4e.7.2 Anaplastic lymphoma kinase(ALK) detection
[1] Not performed (skip to 4e.7.3)
[2] Not clear (skip to 4e.7.3)
[3] Performed (skip to 4e.7.3)
4e.7.2.1 If [3], method of detection
[1] Immunohistochemistry [2] Genetic testing [3] Other(Specify)
4e.7.2.2 If [3], result of detection
[1] Positive [2] Negative [3] Other(Specify) [4] Not clear
4e.7.3 Epidermal growth factor receptor(EGFR) detection
[1] Not performed (skip to 4e.7.4)
[2] Not clear (skip to 4e.7.4)
[3] Performed (skip to 4e.7.4)
4e.7.4.1 If [3], method of detection
[1] Immunohistochemistry [2] Genetic testing [3] Other(Specify)
4e.7.3.2 If [3], result of detection
[1] Positive [2] Negative [3] Other(Specify) [4] Not clear
4e.7.4 K-ras detection
[1] Not performed (skip to 4e.7.5)
[2] Not clear (skip to 4e.7.5)
[3] Performed (skip to 4e.7.5)
4e.7.4.1 If [3], method of detection
[1] Immunohistochemistry [2] Gene mutation detection [3] Other(Specify)
4e.7.4.2 If [3], result of detection
[1] Positive [2] Negative [3] Other(Specify) [4] Not clear
4e.7.5 Other gene factor types detection
[1] Not performed (skip to 4f.1)
[2] Not clear (skip to 4f.1)
[3] Performed (skip to 4f.1)
4e.7.6.1 If [3], method of detection
[1] Immunohistochemistry [2] Gene mutation detection [3] Other(Specify)
4e.7.6.2 If [3], result of detection

For peer review only

[1] Positive [2] Negative [3] Other(Specify) [4] Not clear

f) Staging of lung cancer

4f.1 Type of staging available
[1] Clinical stage [2] Pathological staging [3] Not staging
[4] Not clear
4f.2 Staging methods
[1] Clinical imaging [2] Pathological staging [3] Postoperative pathology
[4] No [5] Not clear
4f.3 If staged, details of TNM staging
4f.3.1 Staging system
[1] The 6th edition of UICC/AJCC staging, published in 2002
[2] The 7th edition of AHCC staging, published in 2009
4f.3.2 T staging
[1] T1; [2] T2; [3] T3; [4] T4; [5] Tx; [6] Not clear
4f.3.3 N staging
[1] N1; [2] N2; [3] N3; [4] N0; [5] Not clear
4f.3.4 M staging
[1] M1; [2] Mx; [3]M0; [4] Not clear
4f.3.5 TNM staging
[1] Stage I; [2] Stage IIA; [3] Stage IIB; [4] Stage IIIA;
[5] Stage IIIB; [6] Stage IV; [7] Others (specify); [8] Not clear
4f.4 Type of lung cancer:
[1] Small cell lung cancer [2] Non-Small cell lung cancer
[3] Mixed small cell lung cancer [4] Not clear
[5] Others (specify)
4f.4.1 If [1], state of lesion
[1] Restricted [2] Pervasive
[3] Other (specify)
4f.4.2 If [2], state of lesion
[1] Early stage [2] Locally advanced
[3] Advanced [4] Not clear

Part E: Treatment procedures and findings/results

5.1 Surgical treatment
[1] Not performed (skip to 5.2)
[2] Thoracotomy
[3] Video-assisted thoracoscopic surgery
[4] Thoracoscope assisted small incision surgery
[5] Others (specify)
[6] Not clear(skip to 5.2)

For peer review only

5.1.1 Details of resection:

[1] Lobectomy [2] Segmental resection
[3] Combined lobectomy [4] Completely pneumonectomy
[5] Sleeve lobectomy [6] Resection and reconstruction of carina
[7] Others (specify) [8] Not clear

5.1.1.1 If [2], name of the segment

5.1.1.2 If [4], treatment of pulmonary arteriovenous in pericardium

[1] Yes [2] No [3] Not clear

5.1.2 If [3], type of thoracoscope assistance:

[1] Single hole [2] Double holes [3] Three holes
[4] Multiple holes [5] Not clear

5.1.2.1 Conversion from video-assisted thoracoscopic surgery to Thoracotomy

[1] Yes [2] No [3] Not clear

5.1.3 Performance of rapid pathology

[1] Yes [2] No [3] Not clear

5.1.4 Findings from intraoperative exploration

5.1.4.1 Tumor site

[1] Left [2] Right [3] Upper lobes
[4] Bottom lobes [5] Middle lobes [6] Not clear

5.1.4.2 Cross lobes

[1] Yes [2] No [3] Not clear

5.1.4.3 Pleural involvement/ Shrinkage

[1] Yes [2] No [3] Not clear

5.1.4.4 Largest diameter of tumor: |__|__|.|__|cm

5.1.4.5 Pleural metastasis

[1] Yes [2] No [3] Not clear

5.1.4.6 Intrapulmonary metastasis

[1] Yes [2] No [3] Not clear

5.1.4.7 Foreign invasion

[1] Yes [2] No [3] Not clear

5.1.4.7.1 If [1], name of invaded tissue:

5.1.4.8 Dual(Multiple) primary tumor

[1] Yes [2] No [3] Not clear

5.1.5 Lymph node dissection

[1] Systematicness [2] Sampling [3] Not cleaned [4] Not Clear

5.1.6 Classification of surgery

[1] Radical cure [2] Palliative treatment [3] Not clear

5.2 Radiation therapy

[1] Not performed (skip to 5.3)
[2] Not clear (skip to 5.3)
[3] Performed

5.2.1 If [3], type of radiation therapy:

[1] Preoperative radiotherapy [2] Postoperative radiotherapy
[3] Radical radiation therapy

For peer review only

5.2.1.1 Combined with chemotherapy:
[1] Not performed (skip to 10.1.3)
[2] Not clear (skip to 10.1.3)
[3] Performed
5.2.1.1.1 If [3], type of chemo-radiotherapy:
[1] Sequence chemoradiotherapy [2] Concurrent chemoradiotherapy
5.2.1.1.2 If [2], name of the chemotherapy drugs
5.2.1.1.3 If [2], chemotherapy cycles:
[1] Every week [2] Biweekly [3] Every 3 weeks
[4] Every 4 weeks [5] Not clear
5.2.1.2 Radiotherapy technique
[1] Routine radiotherapy [2] Three-dimensional conformal radiotherapy
[3] Tomo treatment [4] Static intensity modulated radiotherapy
[5] Stereotactic radiotherapy [6] Rotational intensity modulated radiotherapy
[7] Not clear [8] Others (specify)
5.2.1.3 Polarization
[1] Conventional simulator [2] CT simulation [3] 4D-CT
[4] Not clear
5.2.1.4 Methods of pretreatment position verification
[1] No methods [2] Image guide radiation therapy
[3] Not clear [4] Electronic Portal Imaging Device
[5] Others (specify)
5.2.1.5 Radiation target area (multiple choice)
[1] Primary foci [2] Postoperative stump and tumor bed
[3] Involving lymph node irradiation [4] Choose lymph node irradiation
[5] Metastatic lesions [6] Not clear
5.2.1.6 Radiotherapy dose division program

No	Radiation energy	Total dose Gy	Number of times	Treatment time (days)
[1]				
[2]				
[3]				

5.3 Chemotherapy
[1] Not performed (skip to 5.4)
[2] Not clear (skip to 5.4)
[3] Performed
5.3.1 If [3], type of chemotherapy:
[1] Neoadjuvant chemotherapy [2] Postoperative adjuvant chemotherapy
[3] Advanced chemotherapy [4] Others (specify)
5.3.1.1 If [1], neoadjuvant chemotherapy regimen
[1] Vinorelbin/Cisplatin+Vinorelbin/Carboplatin+Vinorelbin/Other platinum
[2] Paclitaxel/Cisplatin+Paclitaxel/Carboplatin+Paclitaxel/Other platinum
[3] Docetaxel/Cisplatin+ Docetaxel/Carboplatin +Docetaxel/Other platinum
[4] Pemetrexed/Cisplatin+Pemetrexed/Carboplatin+ Pemetrexed/Other platinum
[5] Gemcitabine/Cisplatin +Gemcitabine/Carboplatin +Gemcitabine/Other platinum

For peer review only

[6] Others (specify)
[7] Not clear

5.3.1.2 If [2], postoperative adjuvant chemotherapy regimen:

[1] Vinorelbin/Cisplatin+Vinorelbin/Carboplatin+Vinorelbin/Other platinum
[2] Paclitaxel/Cisplatin+Paclitaxel/Carboplatin+Paclitaxel/Other platinum
[3] Docetaxel/Cisplatin+Docetaxel/Carboplatin+Docetaxel/Other platinum
[4] Pemetrexed/Cisplatin+Pemetrexed/Carboplatin+Pemetrexed/Other platinum
[5] Gemcitabine/Cisplatin+Gemcitabine/Carboplatin+Gemcitabine/Other platinum
[6] Etoposide/Cisplatin+Etoposide/Carboplatin+Cyclophosphamide/Adriamycin/ Vincristine
[7] Others (specify)
[8] Not clear

5.3.1.3 If [3], advanced chemotherapy regimen:

[1] Cisplatin+Carboplatin+Other platinum
[2] Paclitaxel+Docetaxel
[3] Emcitabine
[4] Pemetrexed
[5] Vinorelbine+Vincristine
[6] Irinotecan+Topotecan
[7] Tegafur
[8] Etoposide
[9] Cytoxan+Ifosfamide
[10] Adriamycin
[11] Others(specify)
[12] Not clear

5.4 Complication/comorbidities treatment

5.4.1 Superior vena cava syndrome

[1] Not appeared(skip to 5.4.2) [2] Not clear(skip to 5.4.2) [3] Appeared

5.4.1.1 If [3], duration (month):

5.4.1.2 If [3], treatment:

[1] No (skip to 5.4.2) [2] Not clear(skip to 5.4.2) [3] Yes

5.4.1.2.1 If[3], treatment effect:

[1] Improved [2] Progressed [3] Stable [4] Not clear

5.4.2 Spinal cord compression syndrome

[1] Not appeared (skip to 5.4.3) [2] Not clear(skip to 5.4.3) [3] Appear

5.4.2.1 If [3], duration (month):

5.4.2.2 If [3], treatment:

[1] No (skip to 5.4.3) [2] Not clear(skip to 5.4.3) [3] Yes

5.4.2.2.1 If [3], treatment effect:

[1] Improved [2] Progressed [3] Stable [4] Not clear

5.4.3 Brain metastases

[1] Not appeared (skip to 5.4.4) [2] Not clear(skip to 5.4.4) [3] Appear

5.4.3.1 If [3], duration (month):

5.4.3.2 If [3], treatment:

For peer review only

[1] No (skip to 5.4.4) [2] Not clear(skip to 5.4.4) [3] Yes
5.4.3.2.1 If [3], treatment effect:
[1] Improved [2] Progressed [3] Stable [4] Not clear
5.4.4 Meningeal metastases
[1] Not appeared (skip to 5.4.5) [2] Not clear(skip to 5.4.5) [3] Appear
5.4.4.1 If [3], duration (month):
5.4.4.2 If [3], treatment:
[1] No (skip to 5.4.5) [2] Not clear(skip to 5.4.5) [3] Yes
5.4.4.2.1 If [3], treatment effect:
[1] Improved [2] Progressed [3] Stable [4] Not clear
5.4.5 Pleural effusion
[1] Not appeared (skip to 5.4.6) [2] Not clear(skip to 5.4.6) [3] Appear
5.4.5.1 If [3], duration (month):
5.4.5.2 If [3], treatment:
[1] No (skip to 5.4.6) [2] Not clear(skip to 5.4.6) [3] Yes
5.4.5.2.1 If [3], treatment effect:
[1] Improved [2] Progressed [3] Stable [4] Not clear
5.4.6 Pyoperitoneum
[1] Not appeared (skip to 5.4.7) [2] Not clear(skip to 5.4.7) [3] Appear
5.4.6.1 If [3], duration (month):
5.4.6.2 If [3], treatment:
[1] No (skip to 5.4.7) [2] Not clear(skip to 5.4.7) [3] Yes
5.4.6.2.1 If [3], treatment effect:
[1] Improved [2] Progressed [3] Stable [4] Not clear
5.4.7 Pericardial effusion
[1] Not appeared(skip to 5.4.8) [2] Not clear(skip to 5.4.8) [3] Appear
5.4.7.1 If [3], duration (month):
5.4.7.2 If [3], treatment:
[1] No (skip to 5.4.8) [2] Not clear(skip to 5.4.8) [3] Yes
5.4.7.2.1 If [3], treatment effect:
[1] Improved [2] Progressed [3] Stable [4] Not clear
5.4.8 Intestinal obstruction
[1] Not appeared(skip to 5.4.9) [2] Not clear(skip to 5.4.9) [3] Appear
5.4.8.1 If [3], duration (month):
5.4.8.2 If [3], treatment:
[1] No (skip to 5.4.9) [2] Not clear(skip to 5.4.9) [3] Yes
5.4.8.2.1 If [3], treatment effect:
[1] Improved [2] Progressed [3] Stable [4] Not clear
5.4.9 Pain
[1] Not appeared (skip to 5.4.10) [2] Not clear(skip to 5.4.10) [3] Appear
5.4.9.1 If [3], duration (month):
5.4.9.2 If [3], treatment:
[1] No (skip to 5.4.10) [2] Not clear(skip to 5.4.10) [3] Yes
5.4.9.2.1 If [3], treatment effect (site and score):

For peer review only

5.4.10 Cerebral thrombosis/ hemorrhage
[1] Not appeared (skip to 5.4.11) [2] Not clear(skip to 5.4.11) [3] Appear
5.4.10.1 If [3], duration (month):
5.4.10.2 If [3], treatment:
[1] No (skip to 5.4.11) [2] Not clear(skip to 5.4.11) [3] Yes
5.4.10.2.1 If [3], treatment effect:
[1] Improved [2] Progressed [3] Stable [4] Not clear
5.4.11 Interstitial pneumonia
[1] Not appeared(skip to 5.4.12) [2] Not clear(skip to 5.4.12) [3] Appear
5.4.11.1 If [3], duration (month):
5.4.11.2 If [3], treatment:
[1] No (skip to 5.4.12) [2] Not clear(skip to 5.4.12) [3] Yes
5.4.11.2.1 If [3], treatment effect:
[1] Improved [2] Progressed [3] Stable [4] Not clear
5.4.12 Pulmonary embolism
[1] Not appeared(skip to 5.4.13) [2] Not clear(skip to 5.4.13) [3] Appear
5.4.12.1 If [3], duration (month):
5.4.12.2 If [3], treatment:
[1] No (skip to 5.4.13) [2] Not clear(skip to 5.4.13) [3] Yes
5.4.12.2.1 If [3], treatment effect:
[1] Improved [2] Progressed [3] Stable [4] Not clear
5.4.13 Cardiac insufficiency
[1] Not appeared(skip to 5.4.14) [2] Not clear(skip to 5.4.14) [3] Appear
5.4.13.1 If [3], duration (month):
5.4.13.2 If [3], treatment:
[1] No (skip to 5.4.14) [2] Not clear(skip to 5.4.14) [3] Yes
5.4.13.2.1 If [3], treatment effect:
[1] Improved [2] Progressed [3] Stable [4] Not clear
5.4.14 Arrhythmia
[1] Not appeared(skip to 5.4.15) [2] Not clear(skip to 5.4.15) [3] Appear
5.4.14.1 If [3], duration (month):
5.4.14.2 If [3], treatment:
[1] No (skip to 5.4.15) [2] Not clear(skip to 5.4.15) [3] Yes
5.4.14.2.1 If [3], treatment effect:
[1] Improved [2] Progressed [3] Stable [4] Not clear
5.4.15 Hypercoagulable state
[1] Not appeared (skip to 5.5) [2] Not clear(skip to 5.5) [3] Appear
5.4.15.1 If [3], duration (month):
5.4.15.2 If [3], treatment:
[1] No (skip to 5.5) [2] Not clear(skip to 5.5) [3] Yes
5.4.15.2.1 If [3], treatment effect:
[1] Improved [2] Progressed [3] Stable [4] Not clear
5.5 Other procedures
5.5.1 Interdisciplinary consultation

For peer review only

[1] No (skip to 5.5.2) [2] Not clear(skip to 5.5.2) [3] Yes

5.5.1.1 Disciplines involved

[1] Neurology [2] Infectious diseases [3] Nephrology
[4] Endocrinology [5] Cardiovascular diseases
[6] Others (specify)

5.5.1.2 Total times of consultation:

5.5.2 Psychological/behavioral intervention

[1] No (skip to 5.5.3) [2] Not clear(skip to 5.5.3) [3] Yes

5.5.2.1 Type of interventions performed

[1] Neurology [2] Infectious diseases [3] Nephrology
[4] Endocrinology [5] Cardiovascular diseases
[6] Others (specify)

5.5.2.2 Total sessions of intervention performed:

5.5.3 Traditional Chinese medicine used

[1] No (skip to 10.1) [2] Not clear(skip to 10.1) [3] Yes

5.5.2.1 Regimen of TCM used (specify):

5.5.2.2 Duration of TCM use (days):

Part F: Charges on the inpatient care

6.1 Total inpatient care fee:
6.2 Registration fee
6.3 Bed fee
6.4 Examination fee
6.5 Treatment fee
6.6 Operation fee
6.7 Laboratory fee
6.8 Nursing fee
6.9 Medicines fee
6.10 Other fee

Name of data extractor:
Date of data extraction(dd-mm-yyyy): |__|__|-|__|__|-|__|__|__|__|

For peer review only

Annex 2: Lung cancer patient follow up interview Questionnaire

Reference Number: |__|__|-|__|__|__|__|__|__|

Patient's relationship with the interviewee

[1] Patient himself/herself [2] Spouse
[3] Parent [4] Son/daughter
[5] Brother/sister [6] Other (specify)

Part A: Patient's social demographics and behavior and disease history

1.1: Patient identification number: |__|__|__|__|__|__|__|__|__|__|__|__|__|__|__|__|__|__|

1.2: Patient sex: [1]Male [2]Female

1.3: Patient birth date (dd-mm-yyyy, first case record only): |__|__|-|__|__|-|__|__|__|__|

1.4: Patient education (first case record only):

[1] No formal education [2] Primary school [3] Middle school
[4] High school [5] College [6] Graduate or higher
[7] Not clear

1.5: Patient's occupation (first case record only):

[1] Staff of public entities [2] Employee of firms [3] Self-employed
[4] Peasant [5] Un-employed [6] Retired
[7] Army member [8]Not clear

1.6:Patient's marital status:

[1] Unmarried [2] Married [3] Divorced
[4] Widowed [5] Other [6] Not clear

1.7:Patient's medical insurance:

[1] Essential medical insurance for urban employees
[2] Medical insurance for urban citizens
[3] New rural cooperative medical care systems
[4] Commercial medical insurance
[5] Public medical care system
[6] Out-of-pocket care
[7] Other
[8] Not clear

1.8: Patient's smoking history:

[1] Current smoker [2] Former smoker [3] Non-smoker
[4] Smoker [5] Not clear (skip to 2.1)

1.8.1: Number of cigarettes smoked per day: |__|__|__|
1.8.2: Number of years smoked: |__|__|
1.8.3: Number of years ceased smoking: |__|__|

For peer review only

Part B: Patient's diagnostic and treatment procedures

2.1: When were you (or was he/she) first diagnosed with lung cancer?

Date of diagnosis (dd-mm-yyyy): |__|__|-|__|__|-|__|__|__|__|

2.2: Have you (or Has he/she) been hospitalized due to the lung cancer?

[1] Yes [2] No (skip to 3) [3] Not clear (skip to 3)

2.3: If yes, please tell me, one-by-one, where and when were (or was) you (or he/she) hospitalized due to the lung cancer and how much it costed respectively.

No.	Name of hospital	Admission Date (mm-yyyy)	Total expenditure(RMB)
[1]			
[2]			
[3]			
[4]			
[5]			
[6]			
[7]			
[8]			
[9]			

(Please add more lines as necessary)

2.4: Have you (or Has he/she) sought outpatient treatment for the lung cancer?

[1] Yes [2] No (skip to 3) [3] Not clear (skip to 3)

2.5: If yes, please tell me, one-by-one, where and when had (or was) you (or he/she) received outpatient treatment; what type of treat and how much it costed respectively.

No.	Name of hospital	Date (mm-yyyy)	Type of treatment	Total expenditure(RMB)
[1]				
[2]				
[3]				
[4]				
[5]				
[6]				
[7]				
[8]				
[9]				

(Please add more lines as necessary)

2.6: Have you (or Has he/she) sought medical checkups for monitoring development of the lung cancer?

[1] Yes [2] No (skip to 4) [3] Not clear (skip to 4)

2.7: If yes, please tell me, one-by-one, where and when did the checkup happen and what were the findings respectively

No.	Name of hospital	Date of checkup (mm-yyyy)	Reoccurrence	Metastasis
[1]				
[2]				
[3]				

For peer review only

[4]
[5]
[6]
[7]
[8]
[9]

(Please add more lines as necessary)

2.8: How are you (is he/she) now?

[1] Alive [2] Deceased

2.6.1: If [2], when did it happen (dd-mm-yyyy) ? |__|__|-|__|__|-|__|__|__|__|

2.9: In addition to the inpatient care and medical checkups mentioned above, have you (or has he/she) tried other measures to cure the lung cancer?

[1] Yes [2] No (skip to ending) [3] Not clear (skip to ending)

3.0: If yes, please tell me, one-by-one, what is it and how often it has/had been?

No.	Name of practice	Description of practice	Frequency	Length (months)
[1]				
[2]				
[3]				
[4]				
[5]				
[6]				
[7]				
[8]				
[9]				

(Please add more lines as necessary)

Name of data extractor:

Date of data extraction(dd-mm-yyyy): |__|__|-|__|__|-|__|__|__|__|:

For peer review only

BMJ Open

Pathways and cost-effectiveness of routine lung cancer inpatient care in rural Anhui, China: a retrospective cohort study protocol

Journal:	*BMJ Open*
Manuscript ID	bmjopen-2017-018519.R2
Article Type:	Protocol
Date Submitted by the Author:	13-Nov-2017
Complete List of Authors:	Shen, XingRong; Anhui Medical University Shcool of Health Service Management<br>Diao, MengJie; Anhui Medical University School of Health Service Management<br>Lu, ManMan ; Anhui Medical University School of Health Service Management<br>Feng, Rui; Anhui Medical University, Library Department of Literature Retrieval and Analysis<br>Zhang, PanPan ; Anhui Medical University School of Health Service Management<br>Jiang, Tao ; Anhui Medical University School of Health Service Management<br>Wang, DeBin; Anhui Medical University, School of Health Services Management
**Primary Subject Heading**:	Health economics
Secondary Subject Heading:	Health services research
Keywords:	cost effectiveness, lung cancer, inpatient care, retrospective study, China

SCHOLARONE™
Manuscripts

Pathways and cost-effectiveness of routine lung cancer inpatient care in rural Anhui, China: a retrospective cohort study protocol

XingRong Shen*, MengJie Diao*, ManMan Lu, Rui Feng, PanPan Zhang, Tao Jiang, Debin Wang[#]

Corresponding author: Debing Wang.

Post address: 81 Meishan Road, Hefei, Anhui, China. 230032.

Email: dbwang@vip.sina.com

Telephone: (0086) 5116395

XingRong Shen:

School of Health Service Management, Anhui Medical University, Hefei, Anhui, China.

MengJie Diao:

School of Health Service Management, Anhui Medical University, Hefei, Anhui, China.

ManMan Lu:

School of Health Service Management, Anhui Medical University, Hefei, Anhui, China.

Rui Feng:

Department of Literature Review and Analysis, Library of Anhui Medical University, Hefei, Anhui, China.

PanPan Zhang:

School of Health Service Management, Anhui Medical University, Hefei, Anhui, China.

Tao Jiang:

School of Health Service Management, Anhui Medical University, Hefei, Anhui, China.

Word count：4085

For peer review only

ABSTRACT

Introduction: Routine inpatient care (RIC) for cancer patients forms various pathways of clinical procedures. Although most of the individual procedures comprising the pathways have been tested via clinical trials, little is known about the collective cost and effectiveness of the pathways as a whole. This study aims at examining RIC pathways for lung cancer patients from rural Anhui, China and their determinants and economic impacts.

Methods and analysis: The study adopts a retrospective cohort study design and proceeds in 5 steps. Step 1 defines 4 main categories of study variables including clinical procedures, direct cost and effectiveness of procedures, and factors affecting use of these procedures and their cost and effectiveness. Step 2 selects a cohort of 5000 lung cancer patients diagnosed between July 1, 2014 and June 30, 2015 from rural Anhui by clustered-random sampling. Step 3 retrieves the records of all the inpatient care episodes due to the lung cancer and extracts data about RIC procedures, proximate variables (e.g., Karnofsky performance status, lung function score) of patient outcomes and related factors (e.g., stage of cancer, age, gender) by 2 independent clinician researchers using a web-based form. Step 4 estimates the direct cost of each of the RIC procedures using micro-costing and collects data about ultimate patient outcomes (survival and progression-free survival) through a follow up survey of patients and/or their close relatives. Step 5 analyzes data collected and explores pathways of RIC procedures and their relations with patient outcomes, costs, cost-effect ratios and a whole range of clinical and socio-demographic factors using multivariate regression and path models.

Ethics and dissemination: The study protocol has been approved by authorized ethics committee of Anhui Medical University (reference number: 20170312). Findings from the study will be disseminated through conventional academic routes such as peer-reviewed publications and presentations at regional, national and international conferences.

Trial registry

ISRCTN25595562

Key words: cost effectiveness, lung cancer, inpatient care, retrospective study, China

For peer review only

Strengths and limitations of this study

- The study adopts a retrospective cohort study design involving a large representative sample of community patients;
- It provides data for determining the cost-effectiveness of different treatment approaches as a whole rather than individual procedures;
- It examines pathways of routine inpatient care for a huge but understudied Chinese rural population;
- It extracts data from routine records kept at different hospitals and thus suffers from discrepancies in performances and data qualities.

Introduction

Lung cancer has been the most common cancer in the world for several decades.[1] Estimated new cases of the disease was 1.8 million in 2012 (12.9% of the total), 58% of which occurred in less developed regions. It was also the most common cause of death from cancer worldwide, being responsible for nearly one in five (1.59 million in absolute number) of the total. [2] In China, lung cancer incidence showed a slight decreasing trend in the past few years, particularly for males. However, it is still the top first cancer for males and second for females, accounting for 25.2% of all new cancer cases and 29.5% of all cancer deaths in 2012. [3]

Routine inpatient care (RIC) for lung cancer consists of a combination of procedures. Patients with possible lung cancer need a detailed history and physical examination first. Then they should undergo posterior-anterior and lateral chest radiographs as well as CT scans of the chest and abdomen. In order to further confirm and determine stage and histology of the lesion, other diagnostic methods needed include whole-body fluoro-deoxy-glucose positron emission tomography, endoscopic ultrasound, sputum cytology, fine-needle aspiration, bronchoscopy etc. Following diagnosis of lung cancer, the patients proceed with combined-modality therapies depending on stage of the disease and co-morbidities and complications. Historically, surgery provides the best chance for cure for patients whose lung cancers are limited to the hemithorax and can be totally encompassed by excision.[4 5] Surgery has been generally used in combination with external-beam radiotherapy for control of the primary tumor and regional lymphatics.[6] In addition, chemotherapy has also been advocated as an integral part of combined modality approaches to earlier stages of disease. [7 8] For unselected advanced none-small cell lung cancer, platinum-based combinations have become the standard of care; while cisplatin– or carboplatin-based doublets are standard for patients with stage IV disease.[9 10] More recently, EGFR tyrosine kinase inhibitors have been introduced in second- and third-line treatment of advanced disease and in first-line treatment for selected patients.[11]

Given the complex procedures, ensuring quality RIC for lung cancer patients has been most challenging and guidelines are widely used in addressing this challenge. Numerous

For peer review only

studies have documented positive relations between compliance with guidelines and patient outcomes.[12 13] However, researchers have also raised concerns about guidelines. One of such concerns refers to lack of adequate consideration of costs. Most clinical procedures not only affect disease outcomes but also incur considerable costs.[14 15] Yet guidelines are based on trials focused primarily on effectiveness (e.g., survival) with little attention being paid to economic consequences.[16] Another concern relates to incompatible population between clinical trials and RIC. Clinical trials on which guidelines are based use highly selected populations; while RIC serves a general lung cancer population with different age, performance status and comorbidities.[17 18] A third concern revolves uncertain interactions between procedures. Although most individual guideline recommended procedures (GRPs) have established evidences, they are not used in isolation but in conjunction with others forming various clinical combinations. Efforts systematically assessing and comparing these combinations are scarce.[19-22] A fourth concern originates from varied compliance with guidelines since RIC often deviates substantially from guidelines.[23 24] The cost-effectiveness of these "substandard" or mixed combinations of procedures (partly from guidelines, partly from experiences of individual clinicians) falls far from well-understood.[25] These all point to a clear need for evaluating RIC even though guidelines are widely available.

All the above concerns are most pertinent to China. First, China has a unique "dual" medical care system in which patients often receive western medicine and traditional Chinese medicine simultaneously or in turn.[26] Second, China lacks coordinated referral and follow up mechanisms and cancer patients often moves freely from one hospital to another for different rounds of inpatient cancer care.[27] This makes it hard for clinicians in leveraging different inpatient care episodes at different time points and hospitals into continuous and synergetic service. Third, China has strong socio-cultural norms and financial incentives that hinder cost control and guideline compliance.[28]

Study aims

This study aims at identifying main pathways of RIC procedures for lung cancer patients from rural Anhui, China and examining determinants of the pathways and economic impacts. Specific questions to be addressed include: a) what combinations of diagnosis and treatment procedures (or pathways for short) an individual patient may experience during all his/her hospitalization episodes due to lung cancer-related problems; b) which are the most and least frequent pathways; c) what determines the flow among these pathways; d) which are the most and least cost-effective pathways in relation to the other pathways; and e) what factors are associated with the relative cost-effectiveness.

The above "pathways" of inpatient care means combinations of diagnosis and treatment procedures an individual patient may experience during all his/her hospitalization episodes due to lung cancer-related problems. Suppose a lung cancer patient experienced

For peer review only - http://bmjopen.bmj.com/site/about/guidelines.xhtml

6 times/rounds of hospitalized care and during each of these hospitalization episodes, the patient underwent several diagnosis and treatment procedures, all these procedures form the “pathway” of this particular patient.

Methodology

Identification of procedures

The study uses a self-designed and web-based data extraction form in identifying major clinical procedures described in any RIC record under concern. The form lists all major RIC procedures under two main domains, i.e., diagnostic procedures (e.g., chest X-ray, chest CT, neck ultrasonography) and treatment procedures (e.g., surgical therapy, chemotherapy, psycho-behavioral intervention).

Estimation of costs

The study estimates overall and categorical costs (direct costs only) for each of the RIC procedures (e.g., lung function examination, computed tomography, white blood cell count) identified above using micro-costing techniques.[29 30]Taking the example of lung function examination, categorical costs include costs on personnel, equipment, materials, regents and others needed in completing the examination; while overall cost of the procedure equals the sum of all these categorical costs. In addition, the study also calculates overall cost on individual inpatient by adding up the overall costs on all the clinical procedures he/she has received.

Measurement of effectiveness

The study uses both proximal variables of outcomes (PV) and ultimate outcome (UO) measures of effectiveness of RIC procedures. The UO indicators derive from a follow up survey about 2 years and half after the first hospitalization and include overall survival (OS), progression-free survival (PFS), quality of life (QoL), and quality adjusted life years (QALYs). Here, OoL is assessed using the widely recognized EQ-5D-5L instrument.[31]

The PV measures come from RIC records and include Eastern Cooperative Oncology Group (ECOG), Karnofsky performance status (KPS) and compiled scores of: a) symptoms (e.g., chronic cough, chest pain, wasting syndrome); b) lung functions (e.g., forced vital capacity, forced one second expiratory volume), c) image findings (e.g., number of nodules identified in the lung, size of the largest nodules, presence of pleura or pericardial effusion). Each of these domain specific PV scores equals weighted sum of all sub-indicators within the domain. For example, the compiled score of “lung functions” equals the sum of weighted values of forced vital capacity, forced one second expiratory volume etc. Here the weights come from the coefficients of multivariate regression modeling using an UO indicator (e.g., OS) as the dependent variable; while forced vital

For peer review only

capacity, forced one second expiratory volume etc. as the independent variables; and stage of disease, age, gender and others as the confounding variables.

Calculation of cost-effectiveness

The study adopts relative cost-effectiveness ratios (RCERs) and incremental cost-effectiveness rations (ICERs) as the main indicators for measuring cost-effectiveness. Here ICER is defined by the difference in cost between two selected sets of RIC procedures, divided by the difference in their effect. More specifically, $\mathrm{ICER} = (\mathrm{C}_{\mathrm{r+x}} - \mathrm{C}_{\mathrm{r}})/(\mathrm{E}_{\mathrm{r+x}} - \mathrm{E}_{\mathrm{r}})$, where C_{r} and E_{r} is the cost and effect in the reference group and $\mathrm{C}_{\mathrm{r+x}}$ and $\mathrm{E}_{\mathrm{r+x}}$, the cost and effect in the group who have underwent all the procedures in the reference group plus x, a specific procedure under concern.[32] Suppose, x represents a commonly used traditional Chinese medicine (TCM) which incurs 100 dollars; while r, a typical combination of diagnosis and treatment procedures without the TCM. The combination without the TCM costs 1000 dollars and the survival time of patients who have adopted this combination is 1.5 years on average; while the same figure for patients who have used the combination plus the TCM is 1.51. Then the $\mathrm{C}_{\mathrm{r+x}} = 1000 + 100 = 1100$ dollars and the ICER of the TCM = (1100-100)/(1.51-1.5)=10000 dollars per life year saved. Similarly, $\mathrm{RCER} = (\mathrm{C}_{\mathrm{r+x}}/\mathrm{E}_{\mathrm{r+x}})/(\mathrm{C}_{\mathrm{r}}/\mathrm{E}_{\mathrm{r}}) = (1100/1.51)/\,(1000/1.50) = 1.09$.

Identification of influencing factors

The study also extracts, from RIC records, data about patient factors commonly believed to be linked with disease progression, treatment response and outcomes and utilization of RIC procedures. These include: a) socio-demographics (e.g., age, gender, body height and weight, education, employment, marital status, medical insurance); b) risk behaviors and histories (e.g., smoking, alcohol drinking, history of cancer among family members); c) historical and biological test findings (e.g., value of ALK, KRAS, EGFR, PDL1, CEA, CA125, proGRP); d) comorbidities and complications (e.g., presence of superior vena cava syndrome, brain metastases) and stage of disease. Here, disease staging uses TNM system and this staging will be treated as the most important factor throughout the data analysis especially in its effects on the flow of different pathways and their RCER/ICER.

Selection of participants

The study is implemented in Anhui, an inland province located in middle and east China. It has a population of 61.4 million and its per capita GDP and income rank in the middle (the 14th) among all provinces in the nation.[33 34] Its social, cultural and economic background is representative of over 80% of the whole population in China.[33 34] The province has 68 rural counties and each of them divides into 10 to 20 townships. Selection of participating counties, townships, patients and RIC case records uses a clustered random sampling which proceeds in 5 steps. Step 1 classifies all the counties in Anhui into southern, northern and middle areas. Step 2 randomly selects 3 counties from each of these areas (12 counties in total). Step 3 randomly draws 4 townships from each

For peer review only

of the counties selected (48 townships in total). Step 4 searches the provincial reimbursement database of the New Rural Cooperative Medical System (NRCMS) and identifies all the patients within the selected townships who had been first diagnosed with primary lung cancer during July 1, 2014 and June 30, 2015. Step 5 searches the database again for all episodes of hospitalization due to the lung cancer for the patients identified in step 4. NRCMS covers 98% of the rural residents and the estimated number of patients and admission episodes is about 5,000 and 25,000 respectively.

The above sample size was determined by our study purpose of building multivariate models of factors affecting the flow among and RCER/ICER of specific RIC pathways. Lung cancer patients generally receive 4 to 6 rounds of inpatient care. Given the various diagnostic and treatment procedures available, there are hundreds of potential RIC pathways (combinations of diagnosis and treatment procedures from the first to the last round of RIC). We plan to group these pathways into manageable (around 20) categories depending on the resultant distribution of the actual pathways and we aim to enter 20-30 factors into the multivariate models for each of these categorical pathways. Based on these pre-conditions and that the sample size of a multi-variable model should generally be 10 times the number of independent variables, we need 250 patients for each pathway. This translates into 5000 patients in total.

Data collection

The study obtains data through follow-up survey and data extraction. The follow-up survey applies to all the lung cancer patients identified above. It solicits information about the patient's: a) disease progression (i.e., died, alive with or without progression); b) if died, date of death; c) additional admissions due to the lung cancer not included in the above mentioned NRCMS database. The survey uses a short structured questionnaire. Administration of the questionnaire starts with a telephone interview (of the patient under concern or his/her close relatives for up to 5 time attempts) followed by a face-to-face interview (of the same respondents for up to 2 attempts) if the telephone contacts have failed. The recruitment strives to reach over 85% rate of participation. And the researchers are trained to record reasons of attrition for each of the patients they have lost so as to allow for assessing potential biases. The data extraction applies to records of all the hospital admission episodes identified via the NRCMS database and the follow up survey. It uses a structured web-based form and extracts data about the clinical procedures, costs, effectiveness and influencing factors described above. Two experienced clinicians on care of lung cancer perform the data extraction. They visit (on one-by-one base) all the relevant hospitals, ask for permission to examine the full records and fill the worksheet independently first followed by discussions, if applicable, to solve discrepancies.

For peer review only

Data analysis

The data collected above allow a variety of descriptive and multivariate analysis concerning the costs and effectiveness of RIC. The effectiveness analysis comprises all the UO indicators mentioned above including progression free survival, overall survival, quality of life and DALYs. For each of these UO indicators, the analysis will produce: a) estimation of average rates or values with 95% confidence intervals at different time points after first diagnosis by disease stage, PV indicators, RIC pathways, non-hospital care categories, age range etc.; b) multivariate regression models using similar variables as independent variables; and c) path models using disease stage, RIC pathways, non-hospital care categories, age range etc. as exogenous, complied PV indices as direct endogenous, and individual PV indicators as indirect endogenous variables (Figure 1a). Area under ROC (receiver operating characteristic) curve will be calculated for assessing the predictability of models using binary classifier as the dependent variable (e.g., models of progression free survival, overall survival).

The cost analysis explores mainly: a) overall and categorical costs on different rounds of hospitalization by socio-demographic and selected clinical conditions (Figure 2); b) scatter plot of RIC procedures using the occurrence rate and unit cost of individual procedures as the coordinates; c) multivariate regression models of overall and selected categorical costs using disease stage, PV indicators, RIC pathways, non-hospital care categories, age range etc. as independent variables; and d) Markov models of mean cost for managing lung cancer patients (Figure 1b).

The cost-effectiveness analysis focuses primarily on constructing a pathway tree to help estimate expected overall and pathway specific cost, effectiveness and identify pathways with the highest or lowest RCER/ICER. The tree consists of different branches of combinations of RIC procedures starting from the first to the last episode of inpatient care labeled with estimated costs and possibilities along the pathways and outcomes at the end of the pathways (Figure 3). Relevance of the pathway tree is tested by means of, for instance, varying the percentage of patient flowing among the different pathways or the costs of major diagnostic and treatment procedures consisting the braches and then examining changes in the ranking of the pathways in terms of relative cost-effectiveness. The analysis also pays particular attention to identifying as many as comparable pairs of RIC pathways as possible and calculating RCERs/ICERs accordingly in a hope to uncover potential pathways with practice, policy and research implications.

The pathway tree construction will use TreeAge[35]; while the descriptive and multi-variate model analysis, SPSS 16. Cases with missing data about a specific item will be excluded from the analysis involving the item and where applicable, the statistical null hypothesis is be rejected at the significance level of $\alpha = 0.05$.

Ethics and dissemination

The study protocol had been reviewed and approved by the Biomedical Ethics Committee of Anhui Medical University (reference number: 20170312). Participation of hospitals, patients and their relatives are voluntary and written informed consent is required for all participants. Findings from the study will be disseminated through conventional academic routes such as peer-reviewed publications and presentations and regional, national and international conferences.

Discussion

The study would share the experience of lung cancer care from the rural Chinese perspective. It is an important sharing of knowledge on population-based lung cancer care, since most economic evidence comes from Europe and North America. In China, traditional Chinese medicine is used to complement or replace western medicine. This results in quite different pathways of lung cancer care that have seldom been well explored in published literatures. China has a long history of almost no charges being made for clinical consultations and most patients are used to paying only for medicines, laboratory tests and equipment-based examinations. This forms a perverse financial incentive for clinicians for ordering more sophisticated examinations and tests and for over prescribing. China's lack of referral and follow up mechanisms also merits particular attention. As an individual patient changes from one hospital (say for the first round of treatment) to another (for the second round treatment), he/she may receive different treatment regimens. Discontinued treatment and follow up may make it hard for clinicians to base their treatment decisions on observed effects.

Perhaps the most noteworthy findings of the current study may be the description of the pathways of RIC procedures and their economic impacts (Figure 2). These pathways will provide easily understandable means for estimating and identifying, among others, the following: a) which pathways or combinations of procedures happen most or least in routine practice during different rounds of hospitalization for inpatients suffering from lung cancer in rural China; b) which pathways (from the first to last round of hospitalization) incur the highest or lowest direct costs; and c) which pathways result in the best or worst patient outcome in terms of different UO measures. These have important implications for clinical decision-making as well as policy-making.

Another point worth mentioning in particular refers to the links between the domain specific proximate (PV) indices to key ultimate outcome (UO) indicators (e.g., OS, PFS, QALYs) generated via a large scale (involving 5000 lung cancer patients) retrospective cohort study. They provide useful references for clinicians on care of lung cancer patients in selecting appropriate procedures to achieve optimal collective contributions to UO.[36] At present, although PV indicators are observed routinely, they are presented to clinicians as individual indicators rather than compiled indices. And given the large

For peer review only - http://bmjopen.bmj.com/site/about/guidelines.xhtml

number of PV indicators involved and the complex relations between RIC procedures and PV indicators and then UO indicators, it is difficult for practicing clinicians to make balanced decisions upon their personal experiences.[37]

In addition, this study addresses RIC for lung cancer at hospitals in China from a range of meaningful perspectives. The study reinforces the concepts introduced in the landmark studies of Fisher et al and Wennberg et al, which convincingly demonstrated that high quality was not necessarily associated with high cost.[38] Describing inpatient lung cancer care in a view that its value is directly proportional to outcomes and inversely proportional to costs helps in guiding quality improvement by either better outcomes and/or lower costs. [39] The study calculates and compares the collective costs and effectiveness of different RIC pathways as a whole and thus informs coordinated inpatient care episodes and procedures at different time points and hospitals. The study enables RCER/ICER estimation for specific guideline recommended procedures (GRPs) using various combinations of real and uncontrollable RIC procedures as the reference and thus enhances understanding and application of GRPs established through well-controlled studies in routine practice contexts.

The study also has limitations. The first limit concerns data reliability. Although the majority of data are extracted from RIC records kept at hospitals, the study uses self-reported data about quality of life and inpatient, outpatient and home care. Self-reports are prone to various biases including recall problems particularly among the elderly, over or under reporting by the respondents for reasons like perceived expectations from the researchers or for fearing of potential worries or distress. These biases may be reduced to a minimum in our study by means of interviewer training, use of chorological recall and probing techniques, and cross-checks of findings from patient interviews, health insurance database and hospital records. More importantly, the study uses EQ-5D-5L in assessing quality of life. It has already been tested with adequate reliability both internationally and in China. Regarding non-hospitalized care, the study asks only simple questions about what kind of care the patients have experienced and when and for how long. These questions are relatively memorable and easy to answer. The second limit relates to selective study content. The study considers only inpatient care; while patients may use various self-treatment and outpatient treatment in addition to inpatient care.[40 41] Inpatient and non-inpatient treatment may substitute each other to some extent. These may result in under-estimation of the effectiveness of RIC procedures. Fortunately, this under-estimation may be offset to a large extent by treating non-hospital care as confounders and the study data to be collected allow this exercise. Third, the study considers only direct costs rather than full costs taking both direct and indirect costs into consideration. In addition, different hospitals use different equipment, reagents and medicines. Their quality of case records may also vary substantially. These raise compatibility concerns in pooling data from different hospitals together and performing aggregate analysis. Finally, readers may raise concerns about representativeness of

For peer review only - http://bmjopen.bmj.com/site/about/guidelines.xhtml

inpatients to the larger cancer patients. Hospitalization rates documented from other countries vary greatly;[42] while similar data from China are scarce. Our estimation, using the dataset of the last province-wide Household Health Survey of Anhui, of the proportion of lung cancer patients who had been admitted to hospitals at least once was as high as 89%.[43]

Competing interests

The authors declare no competing interests.

Authors' contributions

XS and MD contributed equally in conceiving this project, facilitating protocol and instrument development, and drafting this manuscript. RF, ML, PZ and TJ are kore researchers for cost estimation, record extraction, follow up survey and data analysis respectively. DW provided expertise for overall design of the study, and revised and finalized the manuscript. All authors have read and approved the final submission.

Acknowledgements

Development of the primitive protocol was supported by the Natural Science Foundation of China (grant number: 81172201). Refinement and implementation of the protocol is lead and supported by Collaboration Center for Cancer Control of Anhui Medical University, Anhui and Luan Center for Diseases Control and Prevention.

References

1. McErlean A, Ginsberg MS. Epidemiology of lung cancer. *Semin Roentgenol* 2011;46 (3):173-7.
2. World Health Organization. GLOBOCAN 2012: Estimated Cancer Incidence, Mortality and Prevalence Worldwide in 2012. http://globocan.iarc.fr/Default.aspx.
3. Kong J, Xu F, He M, Chen K, et al. The incidence of lung cancer by histological type: a population-based study in Tianjin, China during 1981-2005.*Respirology* 2014;19(8):1222-8.
4. Woodard GA, Jablons DM. The Latest in Surgical Management of Stage IIIA Non-Small Cell Lung Cancer: Video-Assisted Thoracic Surgery and Tumor Molecular Profiling. *Am Soc Clin Oncol Educ Book* 2015;35:e435-41.
5. Grunenwald DH. The role of surgery in non-small-cell lung cancers. *Ann Oncol 2005*;16 Suppl 2:ii220-2.
6. Ricardi U, Badellino S, Filippi AR. Stereotactic radiotherapy for early stage non-small cell lung cancer. *Radiat Oncol J*. 2015;33(2):57-65.
7. Mangal S, Gao W, Li T1, Zhou QT. Pulmonary delivery of nanoparticle chemotherapy for the treatment of lung cancers: challenges and opportunities. *Acta Pharmacol Sin*. 2017. doi: 10.1038/aps.2017.34.

For peer review only

8. Khan I, Morris S, Hackshaw A, et al. Cost-effectiveness of first-line erlotinib in patients with advanced non-small-cell lung cancer unsuitable for chemotherapy. *BMJ Open* 2015; 5(7):e006733.
9. Garon EB, Ciuleanu TE, Arrieta O, et al. Ramucirumab plus docetaxel versus placebo plus docetaxel for second-line treatment of stage IV non-small-cell lung cancer after disease progression on platinum-based therapy (REVEL): a multicentre, double-blind, randomised phase 3 trial. *Lancet* 2014;384(9944):665-73.
10. Spigel DR, Luft A, Depenbrock H, et al. An Open-Label, Randomized, Controlled Phase II Study of Paclitaxel-Carboplatin Chemotherapy With Necitumumab Versus Paclitaxel-Carboplatin Alone in First-Line Treatment of Patients With Stage IV Squamous Non-Small-Cell Lung Cancer. *Clin Lung Cancer* 2017; pii: S1525-7304(17)30045-1.
11. Stinchcombe TE. The Use of EGFR Tyrosine Kinase Inhibitors in EGFR Wild-Type Non-Small-Cell Lung Cancer. *Curr Treat Options Oncol* 2016; 17(4):18.
12. Ahmed HZ, Liu Y, O'Connell K, et al. Guideline-concordant Care Improves Overall Survival for Locally Advanced Non-Small-cell Lung Carcinoma Patients: A National Cancer Database Analysis. *Clin Lung Cancer* 2017; pii: S1525-7304(17)30114-6.
13. Nadpara P, Madhavan SS, Tworek C. Guideline-concordant timely lung cancer care and prognosis among elderly patients in the United States: A population-based study. *Cancer Epidemiol*; 39(6):1136-44.
14. Hinde S, McKenna C, Whyte S, et al. Modelling the cost-effectiveness of public awareness campaigns for the early detection of non-small-cell lung cancer. *Br J Cancer* 2015; 113(1):135-41.
15. Kumar G, Woods B, Hess LM, et al. Cost-effectiveness of first-line induction and maintenance treatment sequences in non-squamous non-small cell lung cancer (NSCLC) in the U.S. *Lung Cancer* 2015. pii: S0169-5002(15)00281-0.
16. Warren JL, Harlan LC, Trimble EL, et al. Trends in the receipt of guideline care and survival for women with ovarian cancer: A population-based study. *Gynecol Oncol* 2017;145(3):486-492.
17. Jennens RR, Giles GG, Fox RM. Increasing underrepresentation of elderly patients with advanced colorectal or non-small-cell lung cancer in chemotherapy trials. *Int Med J* 2006;36: 216e220.
18. Murthy VH, Krumholtz HM, Gross CP. Participation in cancer clinical trials; race-, sex-, and age-based disparities. *JAMA* 2004;22(291): 2720-2726.
19. Tong Y, Huang C, Zhang J. A novel EGFR-TKI inhibitor (cAMP-H3BO3 complex) combined with thermal therapy is a promising strategy to improve lung cancer treatment outcomes. *Oncotarget.* Published Online First: 04 May 2017.doi: 10.18632/oncotarget.17628.
20. National Health and Family Planning Commission of China. Guideline for Chinese primary lung cancer diagnosis and treatment (2015 edition). *Chinese Journal of Oncology* 2015.37(1):67-78.
21. Xing Wang, Shi Yan, Yaqi Wang, et al.Surgical Quality Surveillance and Sustaining Improvement of Lung Cancer Surgery Based On Standard Operation Procedure(SOP) : Experience of Single Surgical Team. *Chinese Journal of Lung Cancer;* 20(4): 253-258.
22. Jackman DM, Zhang Y, Dalby C, et al. Cost and Survival Analysis Before and After Implementation of Dana-Farber Clinical Pathways for Patients With Stage IV Non-

For peer review only

Small-Cell Lung Cancer. *J Oncol Pract* 2017;13(4):e346-e352.
23. Okita A, Yamashita M, Abe K, et al. Variance analysis of a clinical pathway of video-assisted single lobectomy for lung cancer. *Surg Today* 2009;39(2):104-9.
24. Duggan KJ, Descallar J, Vinod SK. Application of Guideline Recommended Treatment in Routine Clinical Practice: A Population-based Study of Stage I-IIIB Non-small Cell Lung Cancer. *Clin Oncol (R Coll Radiol)* 2016;28(10):639-47.
25. Heins MJ, de Jong JD, Spronk I, et al. Adherence to cancer treatment guidelines: influence of general and cancer-specific guideline characteristics. *Eur J Public Health* 2016; pii: ckw234. doi: 10.1093/eurpub/ckw234.
26. Yang S, Cui M, Li HY, et al. Meta-analysis of the effectiveness of Chinese and Western integrative medicine on medium and advanced lung cancer. *Chin J Integr Med* 2012;18(11):862-7.
27. XX L, YW Chen, KS Bi. Resolution of Two- way Recerral Problem in China by Studying British National Health Service System. *Chinese General Practice* 2013; 31(16):2926-29.
28. Y Sun, J Wu, SB Xie, et al. Evaluation of the medical staff clinical pathway adherence: Based on comparison of before and after provider payment reform in Henan Province. *Chinese Journal of Health Policy* 2013;6(5):37-43.
29. Henry SG, Ness RM, Stiles RA, Shintani AK, Dittus RS. A cost analysis of colonoscopy using microcosting and time-and-motion techniques. *J Gen Intern Med* 2007, 22(10):1415-21.
30. Cressman S, Lam S, Tammemagi MC,et al. Resource utilization and costs during the initial years of lung cancer screening with computed tomography in Canada. *J Thorac Oncol* 2014, 9(10):1449-58.
31. Khan I,Morris S, Pashayan N, et al. Comparing the mapping between EQ-5D-5L, EQ-5D-3L and the EORTC-QLQ-C30 in non-small cell lung cancer patients. *Health Qual Life Outcomes* 2016, 14:60.
32. SN York. Incremental Cost-Effectiveness Ratio. *Handbook of Disease Burdens and Quality of Life Measures* 2010: 4235-4235.
33. National Bureau of Statistics of China. China Statistical Yearbook 2016. http://www.stats.gov.cn/tjsj/ndsj/2016/indexch.htm (accessed 1 July 2017).
34. Statistics Bureau of Anhui Province. Statistical yearbook of Anhui Province in 2016. http://www.ahtjj.gov.cn/tjj/web/tjnj_view.jsp?strColId=13787135717978521&_index=1# (accessed 1 July 2017).
35. Treeage: Hollman C, Paulden M, Pechlivanoglou P, et al. A Comparison of Four Software Programs for Implementing Decision Analytic Cost-Effectiveness Models. *Pharmacoeconomics* 2017. doi: 10.1007/s40273-017-0510-8.
36. Darling G, Malthaner R, Dickie J, et al. Quality indicators for non-small cell lung cancer operations with use of a modified Delphi consensus process. *Ann Thorac Surg* 2014;98(1):183-90.
37. Fisher A, Manicavasagar V, Sharpe L, et al. A Qualitative Exploration of Clinician Views and Experiences of Treatment Decision-Making in Bipolar II Disorder. *Community Ment Health J*. Published Online First: 19 Jan 2017. doi: 10.1007/s10597-016-0077-4.
38. Wennberg JE, Fisher ES. Finding high quality, efficient providers for value purchasing: cohort methods better than methods based on events. Finding high quality, efficient providers for value purchasing: cohort methods better than methods

For peer review only

based on events. *Med Care* 2002,40(10):853-5.
39. Chen LW, Wilson FA, Gregg A, et al. Measuring the Cost and Value of Quality Improvement Initiatives for Local Health Departments. J Public Health Manag Pract. Published Online First: 1 Mar 2017. doi: 10.1097/PHH.0000000000000552.
40. O' Regan P, Hegarty J. The importance of self-care for fatigue amongst patients undergoing chemotherapy for primary cancer. *Eur J Oncol Nurs* 2017;28:47-55.
41. Dionne-Odom JN, Demark-Wahnefried W, Taylor RA, et al. The self-care practices of family caregivers of persons with poor prognosis cancer: differences by varying levels of caregiver well-being and preparedness. *Support Care Cancer* 2017; 25(8):2437-2444.
42. Prince RM, Atenafu EG, Krzyzanowska MK. Hospitalizations During Systemic Therapy for Metastatic Lung Cancer: A Systematic Review of Real World vs Clinical Trial Outcomes. JAMA Oncol 2015, 1(9):1333-9.
43. Zhao T, Cheng J, Chai J, et al. Inpatient care burden due to cancers in Anhui, China: a cross-sectional household survey. *BMC Public Health* 2016, 16:308.

For peer review only

Figure 1 Schematic structure of sample multivariate models to be built

Figure 2 Simulated cost by selected socio-demographics and clinical characteristics (TC=total cost; KRMB=1000 Chinese yuan)

Figure 3 Anticipated “procedure-outcome” tree of inpatient lung cancer care (Tx = the xth round of hospitalization; Cx = the xth combination of clinical procedures; Px = possibility of using the xth combinations of clinical procedures; Ox = the xth patient outcome index/indicator)

For peer review only

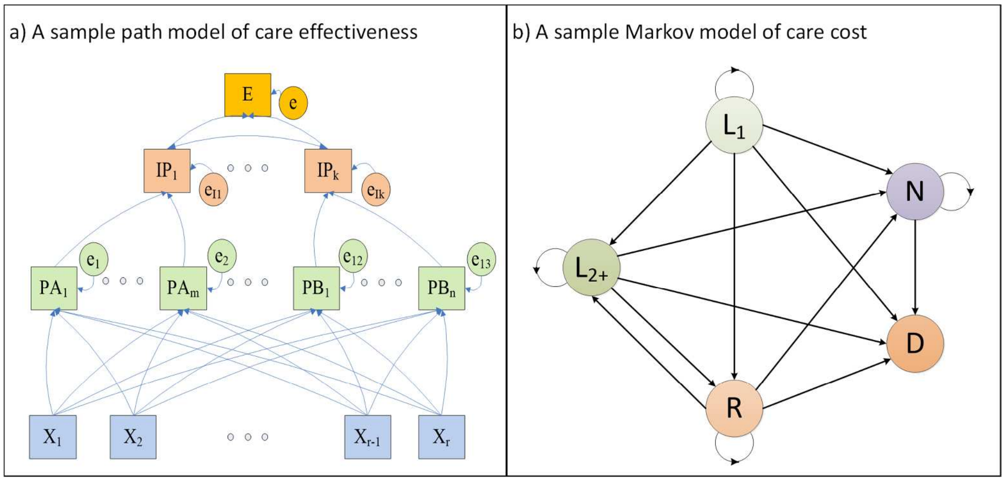

Figure 1 Schematic structure of sample multivariate models to be built/ X=independent variables; PA or PB=domain A or proximate indicators of effectiveness; IP=index of proximate variables; e=systematic error; and E= effectiveness, e.g., overall survival, QALYs; L_1=first line treatment; L_2+=second or third line treatment; R=remission; N=no active treatment; D=death.

188x88mm (300 x 300 DPI)

review only

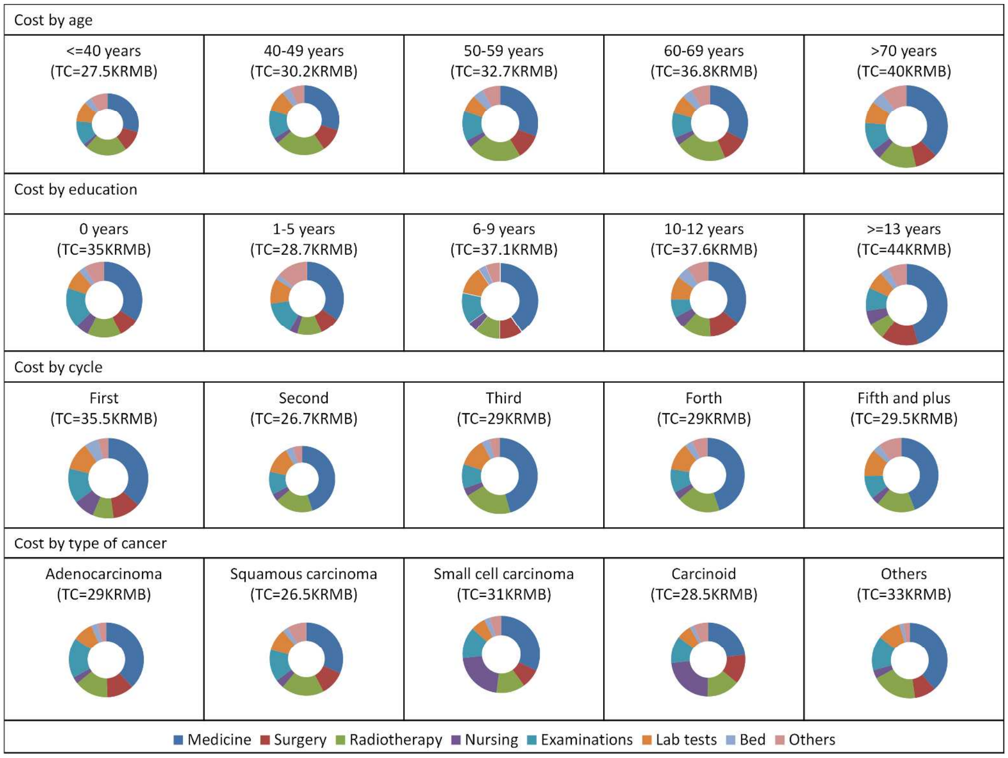

Figure 2 Simulated cost by selected socio-demographics and clinical characteristics (TC=total cost; KRMB=1000 Chinese yuan)

249x187mm (300 x 300 DPI)

w only

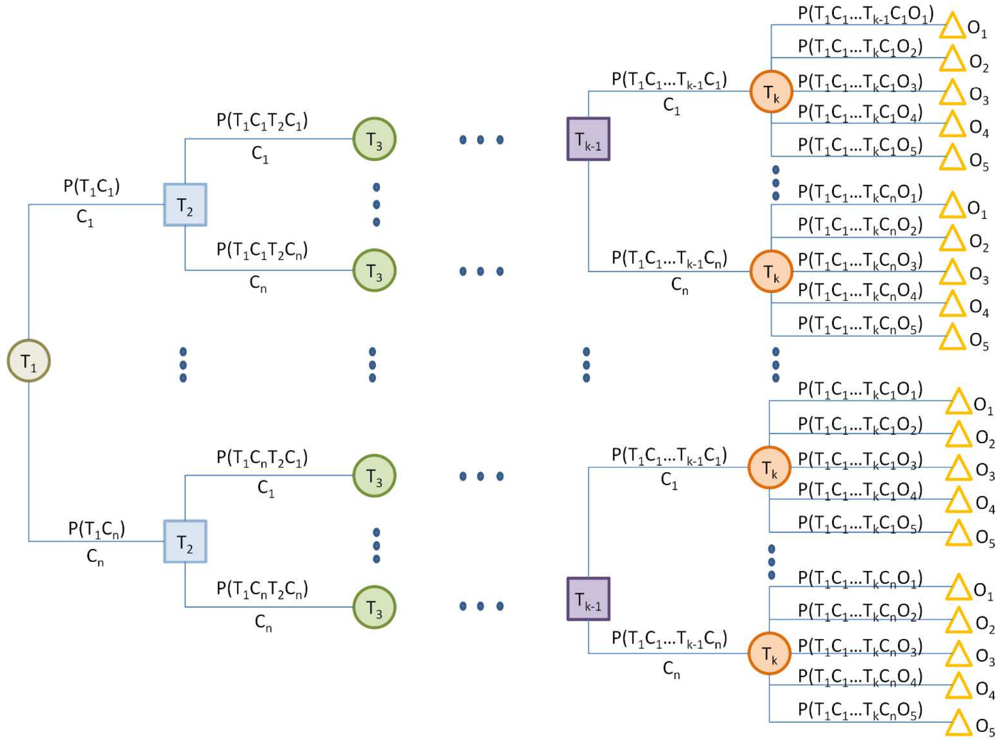

Figure 3 Anticipated "procedure-outcome" tree of inpatient lung cancer care (T_x = the xth round of hospitalization; C_x = the xth combination of clinical procedures; P_x = possibility of using the xth combinations of clinical procedures; O_x = the xth patient outcome index/indicator)

242x183mm (300 x 300 DPI)

BMJ Open

Pathways and cost-effectiveness of routine lung cancer inpatient care in rural Anhui, China: a retrospective cohort study protocol

Journal:	*BMJ Open*
Manuscript ID	bmjopen-2017-018519.R3
Article Type:	Protocol
Date Submitted by the Author:	04-Dec-2017
Complete List of Authors:	Shen, XingRong; Anhui Medical University Shcool of Health Service Management<br>Diao, MengJie; Anhui Medical University School of Health Service Management<br>Lu, ManMan ; Anhui Medical University School of Health Service Management<br>Feng, Rui; Anhui Medical University, Library Department of Literature Retrieval and Analysis<br>Zhang, PanPan ; Anhui Medical University School of Health Service Management<br>Jiang, Tao ; Anhui Medical University School of Health Service Management<br>Wang, DeBin; Anhui Medical University, School of Health Services Management
**Primary Subject Heading**:	Health economics
Secondary Subject Heading:	Health services research
Keywords:	cost effectiveness, lung cancer, inpatient care, retrospective study, China

SCHOLARONE™
Manuscripts

Pathways and cost-effectiveness of routine lung cancer inpatient care in rural Anhui, China: a retrospective cohort study protocol

XingRong Shen*, MengJie Diao*, ManMan Lu, Rui Feng, PanPan Zhang, Tao Jiang, Debin Wang[#]

Corresponding author: Debing Wang.

Post address: 81 Meishan Road, Hefei, Anhui, China. 230032.

Email: dbwang@vip.sina.com

Telephone: (0086) 5116395

XingRong Shen:

School of Health Service Management, Anhui Medical University, Hefei, Anhui, China.

MengJie Diao:

School of Health Service Management, Anhui Medical University, Hefei, Anhui, China.

ManMan Lu:

School of Health Service Management, Anhui Medical University, Hefei, Anhui, China.

Rui Feng:

Department of Literature Review and Analysis, Library of Anhui Medical University, Hefei, Anhui, China.

PanPan Zhang:

School of Health Service Management, Anhui Medical University, Hefei, Anhui, China.

Tao Jiang:

School of Health Service Management, Anhui Medical University, Hefei, Anhui, China.

Word count：4085

For peer review only

ABSTRACT

Introduction: Routine inpatient care (RIC) for cancer patients forms various pathways of clinical procedures. Although most of the individual procedures comprising the pathways have been tested via clinical trials, little is known about the collective cost and effectiveness of the pathways as a whole. This study aims at examining RIC pathways for lung cancer patients from rural Anhui, China and their determinants and economic impacts.

Methods and analysis: The study adopts a retrospective cohort study design and proceeds in 5 steps. Step 1 defines 4 main categories of study variables including clinical procedures, direct cost and effectiveness of procedures, and factors affecting use of these procedures and their cost and effectiveness. Step 2 selects a cohort of 5000 lung cancer patients diagnosed between July 1, 2014 and June 30, 2015 from rural Anhui by clustered-random sampling. Step 3 retrieves the records of all the inpatient care episodes due to the lung cancer and extracts data about RIC procedures, proximate variables (e.g., Karnofsky performance status, lung function score) of patient outcomes and related factors (e.g., stage of cancer, age, gender) by 2 independent clinician researchers using a web-based form. Step 4 estimates the direct cost of each of the RIC procedures using micro-costing and collects data about ultimate patient outcomes (survival and progression-free survival) through a follow up survey of patients and/or their close relatives. Step 5 analyzes data collected and explores pathways of RIC procedures and their relations with patient outcomes, costs, cost-effect ratios and a whole range of clinical and socio-demographic factors using multivariate regression and path models.

Ethics and dissemination: The study protocol has been approved by authorized ethics committee of Anhui Medical University (reference number: 20170312). Findings from the study will be disseminated through conventional academic routes such as peer-reviewed publications and presentations at regional, national and international conferences.

Trial registry

ISRCTN25595562

Key words: cost effectiveness, lung cancer, inpatient care, retrospective study, China

For peer review only

Strengths and limitations of this study

- The study adopts a retrospective cohort study design involving a large representative sample of community patients;
- It provides data for determining the cost-effectiveness of different treatment approaches as a whole rather than individual procedures;
- It examines pathways of routine inpatient care for a huge but understudied Chinese rural population;
- It extracts data from routine records kept at different hospitals and thus suffers from discrepancies in performances and data qualities.

Introduction

Lung cancer has been the most common cancer in the world for several decades.[1] Estimated new cases of the disease was 1.8 million in 2012 (12.9% of the total), 58% of which occurred in less developed regions. It was also the most common cause of death from cancer worldwide, being responsible for nearly one in five (1.59 million in absolute number) of the total. [2] In China, lung cancer incidence showed a slight decreasing trend in the past few years, particularly for males. However, it is still the top first cancer for males and second for females, accounting for 25.2% of all new cancer cases and 29.5% of all cancer deaths in 2012. [3]

Routine inpatient care (RIC) for lung cancer consists of a combination of procedures. Patients with possible lung cancer need a detailed history and physical examination first. Then they should undergo posterior-anterior and lateral chest radiographs as well as CT scans of the chest and abdomen. In order to further confirm and determine stage and histology of the lesion, other diagnostic methods needed include whole-body fluoro-deoxy-glucose positron emission tomography, endoscopic ultrasound, sputum cytology, fine-needle aspiration, bronchoscopy etc. Following diagnosis of lung cancer, the patients proceed with combined-modality therapies depending on stage of the disease and co-morbidities and complications. Historically, surgery provides the best chance for cure for patients whose lung cancers are limited to the hemithorax and can be totally encompassed by excision.[4 5] Surgery has been generally used in combination with external-beam radiotherapy for control of the primary tumor and regional lymphatics.[6] In addition, chemotherapy has also been advocated as an integral part of combined modality approaches to earlier stages of disease. [7 8] For unselected advanced none-small cell lung cancer, platinum-based combinations have become the standard of care; while cisplatin– or carboplatin-based doublets are standard for patients with stage IV disease.[9 10] More recently, EGFR tyrosine kinase inhibitors have been introduced in second- and third-line treatment of advanced disease and in first-line treatment for selected patients.[11]

Given the complex procedures, ensuring quality RIC for lung cancer patients has been most challenging and guidelines are widely used in addressing this challenge. Numerous

For peer review only

studies have documented positive relations between compliance with guidelines and patient outcomes.[12 13] However, researchers have also raised concerns about guidelines. One of such concerns refers to lack of adequate consideration of costs. Most clinical procedures not only affect disease outcomes but also incur considerable costs.[14 15] Yet guidelines are based on trials focused primarily on effectiveness (e.g., survival) with little attention being paid to economic consequences.[16] Another concern relates to incompatible population between clinical trials and RIC. Clinical trials on which guidelines are based use highly selected populations; while RIC serves a general lung cancer population with different age, performance status and comorbidities.[17 18] A third concern revolves uncertain interactions between procedures. Although most individual guideline recommended procedures (GRPs) have established evidences, they are not used in isolation but in conjunction with others forming various clinical combinations. Efforts systematically assessing and comparing these combinations are scarce.[19-22] A fourth concern originates from varied compliance with guidelines since RIC often deviates substantially from guidelines.[23 24] The cost-effectiveness of these "substandard" or mixed combinations of procedures (partly from guidelines, partly from experiences of individual clinicians) falls far from well-understood.[25] These all point to a clear need for evaluating RIC even though guidelines are widely available.

All the above concerns are most pertinent to China. First, China has a unique "dual" medical care system in which patients often receive western medicine and traditional Chinese medicine simultaneously or in turn.[26] Second, China lacks coordinated referral and follow up mechanisms and cancer patients often moves freely from one hospital to another for different rounds of inpatient cancer care.[27] This makes it hard for clinicians in leveraging different inpatient care episodes at different time points and hospitals into continuous and synergetic service. Third, China has strong socio-cultural norms and financial incentives that hinder cost control and guideline compliance.[28]

Study aims

This study aims at identifying main pathways of RIC procedures for lung cancer patients from rural Anhui, China and examining determinants of the pathways and economic impacts. Specific questions to be addressed include: a) what combinations of diagnosis and treatment procedures (or pathways for short) an individual patient may experience during all his/her hospitalization episodes due to lung cancer-related problems; b) which are the most and least frequent pathways; c) what determines the flow among these pathways; d) which are the most and least cost-effective pathways in relation to the other pathways; and e) what factors are associated with the relative cost-effectiveness.

The above "pathways" of inpatient care means combinations of diagnosis and treatment procedures an individual patient may experience during all his/her hospitalization episodes due to lung cancer-related problems. Suppose a lung cancer patient experienced

For peer review only

6 times/rounds of hospitalized care and during each of these hospitalization episodes, the patient underwent several diagnosis and treatment procedures, all these procedures form the "pathway" of this particular patient.

Methodology

Identification of procedures

The study uses a self-designed and web-based data extraction form in identifying major clinical procedures described in any RIC record under concern. The form lists all major RIC procedures under two main domains, i.e., diagnostic procedures (e.g., chest X-ray, chest CT, neck ultrasonography) and treatment procedures (e.g., surgical therapy, chemotherapy, psycho-behavioral intervention).

Estimation of costs

The study estimates overall and categorical costs (direct costs only) for each of the RIC procedures (e.g., lung function examination, computed tomography, white blood cell count) identified above using micro-costing techniques.[29 30]Taking the example of lung function examination, categorical costs include costs on personnel, equipment, materials, regents and others needed in completing the examination; while overall cost of the procedure equals the sum of all these categorical costs. In addition, the study also calculates overall cost on individual inpatient by adding up the overall costs on all the clinical procedures he/she has received.

Measurement of effectiveness

The study uses both proximal variables of outcomes (PV) and ultimate outcome (UO) measures of effectiveness of RIC procedures. The UO indicators derive from a follow up survey about 2 years and half after the first hospitalization and include overall survival (OS), progression-free survival (PFS), quality of life (QoL), and quality adjusted life years (QALYs). Here, OoL is assessed using the widely recognized EQ-5D-5L instrument.[31]

The PV measures come from RIC records and include Eastern Cooperative Oncology Group (ECOG), Karnofsky performance status (KPS) and compiled scores of: a) symptoms (e.g., chronic cough, chest pain, wasting syndrome); b) lung functions (e.g., forced vital capacity, forced one second expiratory volume), c) image findings (e.g., number of nodules identified in the lung, size of the largest nodules, presence of pleura or pericardial effusion). Each of these domain specific PV scores equals weighted sum of all sub-indicators within the domain. For example, the compiled score of "lung functions" equals the sum of weighted values of forced vital capacity, forced one second expiratory volume etc. Here the weights come from the coefficients of multivariate regression modeling using an UO indicator (e.g., OS) as the dependent variable; while forced vital

For peer review only - http://bmjopen.bmj.com/site/about/guidelines.xhtml

capacity, forced one second expiratory volume etc. as the independent variables; and stage of disease, age, gender and others as the confounding variables.

Calculation of cost-effectiveness

The study adopts relative cost-effectiveness ratios (RCERs) as the main indicators for measuring cost-effectiveness. Here RCER is defined by the difference in cost between two selected sets of RIC procedures, divided by the difference in their effect. More specifically, $\mathrm{RCER} = (\mathrm{C}_{\mathrm{r+x}} - \mathrm{C}_{\mathrm{r}})/(\mathrm{E}_{\mathrm{r+x}} - \mathrm{E}_{\mathrm{r}})$, where $\mathrm{C_r}$ and $\mathrm{E_r}$ is the cost and effect in the reference group and $\mathrm{C_{r+x}}$ and $\mathrm{E_{r+x}}$, the cost and effect in the group who have underwent all the procedures in the reference group plus x, a specific procedure under concern.[32] Suppose, x represents a commonly used traditional Chinese medicine (TCM) which incurs 100 dollars; while r, a typical combination of diagnosis and treatment procedures without the TCM. The combination without the TCM costs 1000 dollars and the survival time of patients who have adopted this combination is 1.5 years on average; while the same figure for patients who have used the combination plus the TCM is 1.51. Then the $\mathrm{C_{r+x}} = 1000 + 100 = 1100$ dollars and the RCER of the TCM = (1100-100)/(1.51-1.5)=10000 dollars per life year saved. Similarly, $\mathrm{RCER} = (\mathrm{C_{r+x}}/\mathrm{E_{r+x}})/(\mathrm{C_r}/\mathrm{E_r}) = (1100/1.51)/\ (1000/1.50) = 1.09$.

Identification of influencing factors

The study also extracts, from RIC records, data about patient factors commonly believed to be linked with disease progression, treatment response and outcomes and utilization of RIC procedures. These include: a) socio-demographics (e.g., age, gender, body height and weight, education, employment, marital status, medical insurance); b) risk behaviors and histories (e.g., smoking, alcohol drinking, history of cancer among family members); c) historical and biological test findings (e.g., value of ALK, KRAS, EGFR, PDL1, CEA, CA125, proGRP); d) comorbidities and complications (e.g., presence of superior vena cava syndrome, brain metastases) and stage of disease. Here, disease staging uses TNM system and this staging will be treated as the most important factor throughout the data analysis especially in its effects on the flow of different pathways and their RCER.

Selection of participants

The study is implemented in Anhui, an inland province located in middle and east China. It has a population of 61.4 million and its per capita GDP and income rank in the middle (the 14th) among all provinces in the nation.[33 34] Its social, cultural and economic background is representative of over 80% of the whole population in China.[33 34] The province has 68 rural counties and each of them divides into 10 to 20 townships. Selection of participating counties, townships, patients and RIC case records uses a clustered random sampling which proceeds in 5 steps. Step 1 classifies all the counties in Anhui into southern, northern and middle areas. Step 2 randomly selects 3 counties from each of these areas (12 counties in total). Step 3 randomly draws 4 townships from each of the counties selected (48 townships in total). Step 4 searches the provincial

For peer review only

reimbursement database of the New Rural Cooperative Medical System (NRCMS) and identifies all the patients within the selected townships who had been first diagnosed with primary lung cancer during July 1, 2014 and June 30, 2015. Step 5 searches the database again for all episodes of hospitalization due to the lung cancer for the patients identified in step 4. NRCMS covers 98% of the rural residents and the estimated number of patients and admission episodes is about 5,000 and 25,000 respectively.

The above sample size was determined by our study purpose of building multivariate models of factors affecting the flow among and RCER of specific RIC pathways. Lung cancer patients generally receive 4 to 6 rounds of inpatient care. Given the various diagnostic and treatment procedures available, there are hundreds of potential RIC pathways (combinations of diagnosis and treatment procedures from the first to the last round of RIC). We plan to group these pathways into manageable (around 20) categories depending on the resultant distribution of the actual pathways and we aim to enter 20-30 factors into the multivariate models for each of these categorical pathways. Based on these pre-conditions and that the sample size of a multi-variable model should generally be 10 times the number of independent variables, we need 250 patients for each pathway. This translates into 5000 patients in total.

Data collection

The study obtains data through follow-up survey and data extraction. The follow-up survey applies to all the lung cancer patients identified above. It solicits information about the patient's: a) disease progression (i.e., died, alive with or without progression); b) if died, date of death; c) additional admissions due to the lung cancer not included in the above mentioned NRCMS database. The survey uses a short structured questionnaire. Administration of the questionnaire starts with a telephone interview (of the patient under concern or his/her close relatives for up to 5 time attempts) followed by a face-to-face interview (of the same respondents for up to 2 attempts) if the telephone contacts have failed. The recruitment strives to reach over 85% rate of participation. And the researchers are trained to record reasons of attrition for each of the patients they have lost so as to allow for assessing potential biases. The data extraction applies to records of all the hospital admission episodes identified via the NRCMS database and the follow up survey. It uses a structured web-based form and extracts data about the clinical procedures, costs, effectiveness and influencing factors described above. Two experienced clinicians on care of lung cancer perform the data extraction. They visit (on one-by-one base) all the relevant hospitals, ask for permission to examine the full records and fill the worksheet independently first followed by discussions, if applicable, to solve discrepancies.

Data analysis

The data collected above allow a variety of descriptive and multivariate analysis concerning the costs and effectiveness of RIC. The effectiveness analysis comprises all

For peer review only

the UO indicators mentioned above including progression free survival, overall survival, quality of life and DALYs. For each of these UO indicators, the analysis will produce: a) estimation of average rates or values with 95% confidence intervals at different time points after first diagnosis by disease stage, PV indicators, RIC pathways, non-hospital care categories, age range etc.; b) multivariate regression models using similar variables as independent variables; and c) path models using disease stage, RIC pathways, non-hospital care categories, age range etc. as exogenous, complied PV indices as direct endogenous, and individual PV indicators as indirect endogenous variables (Figure 1a). Area under ROC (receiver operating characteristic) curve will be calculated for assessing the predictability of models using binary classifier as the dependent variable (e.g., models of progression free survival, overall survival).

The cost analysis explores mainly: a) Markov models of mean cost for managing lung cancer patients (Figure 1b); b) overall and categorical costs on different rounds of hospitalization by socio-demographic and selected clinical conditions (Figure 2); c) scatter plot of RIC procedures using the occurrence rate and unit cost of individual procedures as the coordinates; and d) multivariate regression models of overall and selected categorical costs using disease stage, PV indicators, RIC pathways, non-hospital care categories, age range etc. as independent variables.

The cost-effectiveness analysis focuses primarily on constructing a pathway tree to help estimate expected overall and pathway specific cost, effectiveness and identify pathways with the highest or lowest RCER. The tree consists of different branches of combinations of RIC procedures starting from the first to the last episode of inpatient care labeled with estimated costs and possibilities along the pathways and outcomes at the end of the pathways (Figure 3). Relevance of the pathway tree is tested by means of, for instance, varying the percentage of patient flowing among the different pathways or the costs of major diagnostic and treatment procedures consisting the braches and then examining changes in the ranking of the pathways in terms of relative cost-effectiveness. The analysis also pays particular attention to identifying as many as comparable pairs of RIC pathways as possible and calculating RCER accordingly in a hope to uncover potential pathways with practice, policy and research implications.

The pathway tree construction will use TreeAge[35]; while the descriptive and multi-variate model analysis, SPSS 16. Cases with missing data about a specific item will be excluded from the analysis involving the item and where applicable, the statistical null hypothesis is be rejected at the significance level of $\alpha = 0.05$.

Ethics and dissemination

The study protocol had been reviewed and approved by the Biomedical Ethics Committee of Anhui Medical University (reference number: 20170312). Participation of hospitals, patients and their relatives are voluntary and written informed consent is

For peer review only - http://bmjopen.bmj.com/site/about/guidelines.xhtml

required for all participants. Findings from the study will be disseminated through conventional academic routes such as peer-reviewed publications and presentations and regional, national and international conferences.

Discussion

The study would share the experience of lung cancer care from the rural Chinese perspective. It is an important sharing of knowledge on population-based lung cancer care, since most economic evidence comes from Europe and North America. In China, traditional Chinese medicine is used to complement or replace western medicine. This results in quite different pathways of lung cancer care that have seldom been well explored in published literatures. China has a long history of almost no charges being made for clinical consultations and most patients are used to paying only for medicines, laboratory tests and equipment-based examinations. This forms a perverse financial incentive for clinicians for ordering more sophisticated examinations and tests and for over prescribing. China's lack of referral and follow up mechanisms also merits particular attention. As an individual patient changes from one hospital (say for the first round of treatment) to another (for the second round treatment), he/she may receive different treatment regimens. Discontinued treatment and follow up may make it hard for clinicians to base their treatment decisions on observed effects.

Perhaps the most noteworthy findings of the current study may be the description of the pathways of RIC procedures and their economic impacts (Figure 2). These pathways will provide easily understandable means for estimating and identifying, among others, the following: a) which pathways or combinations of procedures happen most or least in routine practice during different rounds of hospitalization for inpatients suffering from lung cancer in rural China; b) which pathways (from the first to last round of hospitalization) incur the highest or lowest direct costs; and c) which pathways result in the best or worst patient outcome in terms of different UO measures. These have important implications for clinical decision-making as well as policy-making.

Another point worth mentioning in particular refers to the links between the domain specific proximate (PV) indices to key ultimate outcome (UO) indicators (e.g., OS, PFS, QALYs) generated via a large scale (involving 5000 lung cancer patients) retrospective cohort study. They provide useful references for clinicians on care of lung cancer patients in selecting appropriate procedures to achieve optimal collective contributions to UO.[36] At present, although PV indicators are observed routinely, they are presented to clinicians as individual indicators rather than compiled indices. And given the large number of PV indicators involved and the complex relations between RIC procedures and PV indicators and then UO indicators, it is difficult for practicing clinicians to make balanced decisions upon their personal experiences.[37]

For peer review only

In addition, this study addresses RIC for lung cancer at hospitals in China from a range of meaningful perspectives. The study reinforces the concepts introduced in the landmark studies of Fisher et al and Wennberg et al, which convincingly demonstrated that high quality was not necessarily associated with high cost.[38] Describing inpatient lung cancer care in a view that its value is directly proportional to outcomes and inversely proportional to costs helps in guiding quality improvement by either better outcomes and/or lower costs. [39] The study calculates and compares the collective costs and effectiveness of different RIC pathways as a whole and thus informs coordinated inpatient care episodes and procedures at different time points and hospitals. The study enables RCER estimation for specific guideline recommended procedures (GRPs) using various combinations of real and uncontrollable RIC procedures as the reference and thus enhances understanding and application of GRPs established through well-controlled studies in routine practice contexts.

The study also has limitations. The first limit concerns data reliability. Although the majority of data are extracted from RIC records kept at hospitals, the study uses self-reported data about quality of life and inpatient, outpatient and home care. Self-reports are prone to various biases including recall problems particularly among the elderly, over or under reporting by the respondents for reasons like perceived expectations from the researchers or for fearing of potential worries or distress. These biases may be reduced to a minimum in our study by means of interviewer training, use of chorological recall and probing techniques, and cross-checks of findings from patient interviews, health insurance database and hospital records. More importantly, the study uses EQ-5D-5L in assessing quality of life. It has already been tested with adequate reliability both internationally and in China. Regarding non-hospitalized care, the study asks only simple questions about what kind of care the patients have experienced and when and for how long. These questions are relatively memorable and easy to answer. The second limit relates to selective study content. The study considers only inpatient care; while patients may use various self-treatment and outpatient treatment in addition to inpatient care.[40 41] Inpatient and non-inpatient treatment may substitute each other to some extent. These may result in under-estimation of the effectiveness of RIC procedures. Fortunately, this under-estimation may be offset to a large extent by treating non-hospital care as confounders and the study data to be collected allow this exercise. Third, the study considers only direct costs rather than full costs taking both direct and indirect costs into consideration. In addition, different hospitals use different equipment, reagents and medicines. Their quality of case records may also vary substantially. These raise compatibility concerns in pooling data from different hospitals together and performing aggregate analysis. Finally, readers may raise concerns about representativeness of inpatients to the larger cancer patients. Hospitalization rates documented from other countries vary greatly; [42] while similar data from China are scarce. Our estimation, using the dataset of the last province-wide Household Health Survey of Anhui, of the

For Peer Review Only

proportion of lung cancer patients who had been admitted to hospitals at least once was as high as 89%.[43]

Competing interests

The authors declare no competing interests.

Authors' contributions

XS and MD contributed equally in conceiving this project, facilitating protocol and instrument development, and drafting this manuscript. RF, ML, PZ and TJ are kore researchers for cost estimation, record extraction, follow up survey and data analysis respectively. DW provided expertise for overall design of the study, and revised and finalized the manuscript. All authors have read and approved the final submission.

Acknowledgements

Development of the primitive protocol was supported by the Natural Science Foundation of China (grant number: 81172201). Refinement and implementation of the protocol is lead and supported by Collaboration Center for Cancer Control of Anhui Medical University, Anhui and Luan Center for Diseases Control and Prevention.

References

1. McErlean A, Ginsberg MS. Epidemiology of lung cancer. *Semin Roentgenol* 2011;46 (3):173-7.
2. World Health Organization. GLOBOCAN 2012: Estimated Cancer Incidence, Mortality and Prevalence Worldwide in 2012. http://globocan.iarc.fr/Default.aspx.
3. Kong J, Xu F, He M, Chen K, et al. The incidence of lung cancer by histological type: a population-based study in Tianjin, China during 1981-2005.*Respirology* 2014;19(8):1222-8.
4. Woodard GA, Jablons DM. The Latest in Surgical Management of Stage IIIA Non-Small Cell Lung Cancer: Video-Assisted Thoracic Surgery and Tumor Molecular Profiling. *Am Soc Clin Oncol Educ Book* 2015;35:e435-41.
5. Grunenwald DH. The role of surgery in non-small-cell lung cancers. *Ann Oncol 2005*;16 Suppl 2:ii220-2.
6. Ricardi U, Badellino S, Filippi AR. Stereotactic radiotherapy for early stage non-small cell lung cancer. *Radiat Oncol J*. 2015;33(2):57-65.
7. Mangal S, Gao W, Li T1, Zhou QT. Pulmonary delivery of nanoparticle chemotherapy for the treatment of lung cancers: challenges and opportunities. *Acta Pharmacol Sin*. 2017. doi: 10.1038/aps.2017.34.
8. Khan I, Morris S, Hackshaw A, et al. Cost-effectiveness of first-line erlotinib in patients with advanced non-small-cell lung cancer unsuitable for chemotherapy. *BMJ Open* 2015; 5(7):e006733.

For peer review only

9. Garon EB, Ciuleanu TE, Arrieta O, et al. Ramucirumab plus docetaxel versus placebo plus docetaxel for second-line treatment of stage IV non-small-cell lung cancer after disease progression on platinum-based therapy (REVEL): a multicentre, double-blind, randomised phase 3 trial. *Lancet* 2014;384(9944):665-73.
10. Spigel DR, Luft A, Depenbrock H, et al. An Open-Label, Randomized, Controlled Phase II Study of Paclitaxel-Carboplatin Chemotherapy With Necitumumab Versus Paclitaxel-Carboplatin Alone in First-Line Treatment of Patients With Stage IV Squamous Non-Small-Cell Lung Cancer. *Clin Lung Cancer* 2017; pii: S1525-7304(17)30045-1.
11. Stinchcombe TE. The Use of EGFR Tyrosine Kinase Inhibitors in EGFR Wild-Type Non-Small-Cell Lung Cancer. *Curr Treat Options Oncol* 2016; 17(4):18.
12. Ahmed HZ, Liu Y, O'Connell K, et al. Guideline-concordant Care Improves Overall Survival for Locally Advanced Non-Small-cell Lung Carcinoma Patients: A National Cancer Database Analysis. *Clin Lung Cancer* 2017; pii: S1525-7304(17)30114-6.
13. Nadpara P, Madhavan SS, Tworek C. Guideline-concordant timely lung cancer care and prognosis among elderly patients in the United States: A population-based study. *Cancer Epidemiol*; 39(6):1136-44.
14. Hinde S, McKenna C, Whyte S, et al. Modelling the cost-effectiveness of public awareness campaigns for the early detection of non-small-cell lung cancer. *Br J Cancer* 2015; 113(1):135-41.
15. Kumar G, Woods B, Hess LM, et al. Cost-effectiveness of first-line induction and maintenance treatment sequences in non-squamous non-small cell lung cancer (NSCLC) in the U.S. *Lung Cancer* 2015. pii: S0169-5002(15)00281-0.
16. Warren JL, Harlan LC, Trimble EL, et al. Trends in the receipt of guideline care and survival for women with ovarian cancer: A population-based study. *Gynecol Oncol* 2017;145(3):486-492.
17. Jennens RR, Giles GG, Fox RM. Increasing underrepresentation of elderly patients with advanced colorectal or non-small-cell lung cancer in chemotherapy trials. *Int Med J* 2006;36: 216e220.
18. Murthy VH, Krumholtz HM, Gross CP. Participation in cancer clinical trials; race-, sex-, and age-based disparities. *JAMA* 2004;22(291): 2720-2726.
19. Tong Y, Huang C, Zhang J. A novel EGFR-TKI inhibitor (cAMP-H3BO3 complex) combined with thermal therapy is a promising strategy to improve lung cancer treatment outcomes. *Oncotarget.* Published Online First: 04 May 2017.doi: 10.18632/oncotarget.17628.
20. National Health and Family Planning Commission of China. Guideline for Chinese primary lung cancer diagnosis and treatment (2015 edition). *Chinese Journal of Oncology* 2015.37(1):67-78.
21. Xing Wang, Shi Yan, Yaqi Wang, et al.Surgical Quality Surveillance and Sustaining Improvement of Lung Cancer Surgery Based On Standard Operation Procedure(SOP) : Experience of Single Surgical Team. *Chinese Journal of Lung Cancer;* 20(4): 253-258.
22. Jackman DM, Zhang Y, Dalby C, et al. Cost and Survival Analysis Before and After Implementation of Dana-Farber Clinical Pathways for Patients With Stage IV Non-Small-Cell Lung Cancer. *J Oncol Pract* 2017;13(4):e346-e352.
23. Okita A, Yamashita M, Abe K, et al. Variance analysis of a clinical pathway of video-assisted single lobectomy for lung cancer. *Surg Today* 2009;39(2):104-9.

For peer review only

24. Duggan KJ, Descallar J, Vinod SK. Application of Guideline Recommended Treatment in Routine Clinical Practice: A Population-based Study of Stage I-IIIB Non-small Cell Lung Cancer. *Clin Oncol (R Coll Radiol)* 2016;28(10):639-47.
25. Heins MJ, de Jong JD, Spronk I, et al. Adherence to cancer treatment guidelines: influence of general and cancer-specific guideline characteristics. *Eur J Public Health* 2016; pii: ckw234. doi: 10.1093/eurpub/ckw234.
26. Yang S, Cui M, Li HY, et al. Meta-analysis of the effectiveness of Chinese and Western integrative medicine on medium and advanced lung cancer. *Chin J Integr Med* 2012;18(11):862-7.
27. XX L, YW Chen, KS Bi. Resolution of Two- way Recerral Problem in China by Studying British National Health Service System. *Chinese General Practice* 2013; 31(16):2926-29.
28. Y Sun, J Wu, SB Xie, et al. Evaluation of the medical staff clinical pathway adherence: Based on comparison of before and after provider payment reform in Henan Province. *Chinese Journal of Health Policy* 2013;6(5):37-43.
29. Henry SG, Ness RM, Stiles RA, Shintani AK, Dittus RS. A cost analysis of colonoscopy using microcosting and time-and-motion techniques. *J Gen Intern Med* 2007, 22(10):1415-21.
30. Cressman S, Lam S, Tammemagi MC,et al. Resource utilization and costs during the initial years of lung cancer screening with computed tomography in Canada. *J Thorac Oncol* 2014, 9(10):1449-58.
31. Khan I,Morris S, Pashayan N, et al. Comparing the mapping between EQ-5D-5L, EQ-5D-3L and the EORTC-QLQ-C30 in non-small cell lung cancer patients. *Health Qual Life Outcomes* 2016, 14:60.
32. SN York. Incremental Cost-Effectiveness Ratio. *Handbook of Disease Burdens and Quality of Life Measures* 2010: 4235-4235.
33. National Bureau of Statistics of China. China Statistical Yearbook 2016. http://www.stats.gov.cn/tjsj/ndsj/2016/indexch.htm (accessed 1 July 2017).
34. Statistics Bureau of Anhui Province. Statistical yearbook of Anhui Province in 2016. http://www.ahtjj.gov.cn/tjj/web/tjnj_view.jsp?strColId=13787135717978521&_index=1# (accessed 1 July 2017).
35. Treeage: Hollman C, Paulden M, Pechlivanoglou P, et al. A Comparison of Four Software Programs for Implementing Decision Analytic Cost-Effectiveness Models. *Pharmacoeconomics* 2017. doi: 10.1007/s40273-017-0510-8.
36. Darling G, Malthaner R, Dickie J, et al. Quality indicators for non-small cell lung cancer operations with use of a modified Delphi consensus process. *Ann Thorac Surg* 2014;98(1):183-90.
37. Fisher A, Manicavasagar V, Sharpe L, et al. A Qualitative Exploration of Clinician Views and Experiences of Treatment Decision-Making in Bipolar II Disorder. *Community Ment Health J*. Published Online First: 19 Jan 2017. doi: 10.1007/s10597-016-0077-4.
38. Wennberg JE, Fisher ES. Finding high quality, efficient providers for value purchasing: cohort methods better than methods based on events. Finding high quality, efficient providers for value purchasing: cohort methods better than methods based on events. *Med Care* 2002,40(10):853-5.
39. Chen LW, Wilson FA, Gregg A, et al. Measuring the Cost and Value of Quality Improvement Initiatives for Local Health Departments. J Public Health Manag Pract.

For peer review only

Published Online First: 1 Mar 2017. doi: 10.1097/PHH.0000000000000552.
40. O' Regan P, Hegarty J. The importance of self-care for fatigue amongst patients undergoing chemotherapy for primary cancer. *Eur J Oncol Nurs* 2017;28:47-55.
41. Dionne-Odom JN, Demark-Wahnefried W, Taylor RA, et al. The self-care practices of family caregivers of persons with poor prognosis cancer: differences by varying levels of caregiver well-being and preparedness. *Support Care Cancer* 2017; 25(8):2437-2444.
42. Prince RM, Atenafu EG, Krzyzanowska MK. Hospitalizations During Systemic Therapy for Metastatic Lung Cancer: A Systematic Review of Real World vs Clinical Trial Outcomes. JAMA Oncol 2015, 1(9):1333-9.
43. Zhao T, Cheng J, Chai J, et al. Inpatient care burden due to cancers in Anhui, China: a cross-sectional household survey. *BMC Public Health* 2016, 16:308.

For peer review only

Figure 1 Schematic structure of sample multivariate models to be built

Figure 2 Simulated cost by selected socio-demographics and clinical characteristics (TC=total cost; KRMB=1000 Chinese yuan)

Figure 3 Anticipated "procedure-outcome" tree of inpatient lung cancer care (Tx = the xth round of hospitalization; Cx = the xth combination of clinical procedures; Px = possibility of using the xth combinations of clinical procedures; Ox = the xth patient outcome index/indicator)

For peer review only

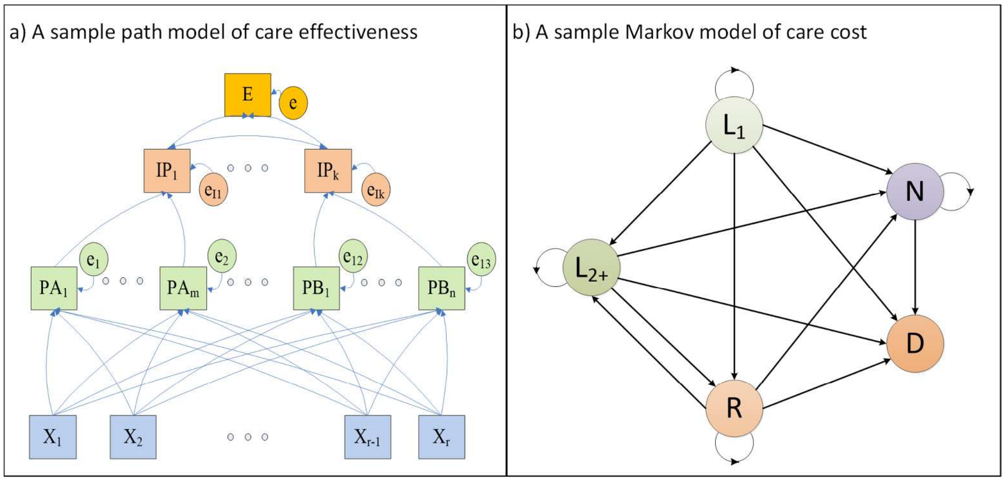

Figure 1 Schematic structure of sample multivariate models to be built/ X=independent variables; PA or PB=domain A or proximate indicators of effectiveness; IP=index of proximate variables; e=systematic error; and E= effectiveness, e.g., overall survival, QALYs; L_1=first line treatment; L_2+=second or third line treatment; R=remission; N=no active treatment; D=death.

188x88mm (300 x 300 DPI)

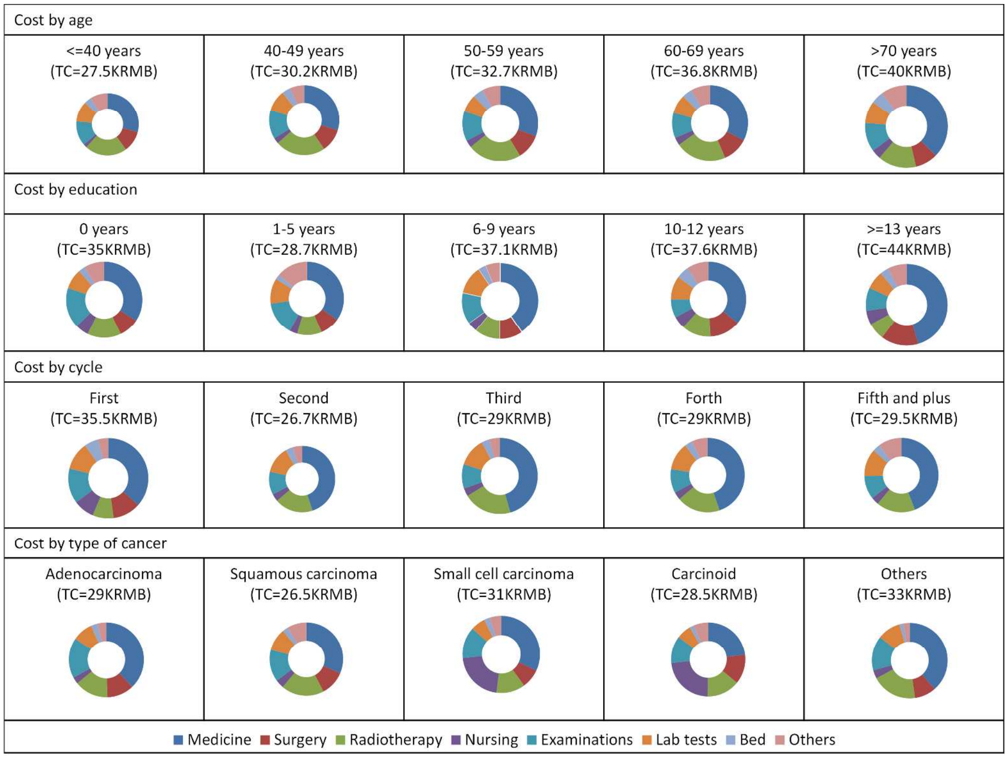

Figure 2 Simulated cost by selected socio-demographics and clinical characteristics (TC=total cost; KRMB=1000 Chinese yuan)

249x187mm (300 x 300 DPI)

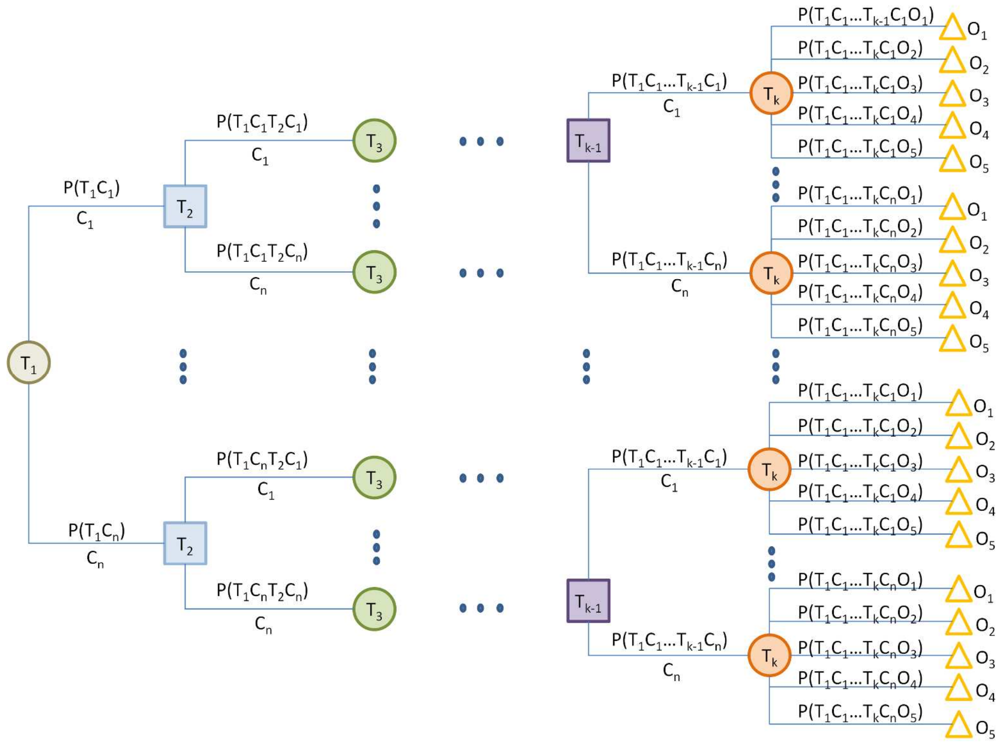

Figure 3 Anticipated “procedure-outcome” tree of inpatient lung cancer care (T_x = the xth round of hospitalization; C_x = the xth combination of clinical procedures; P_x = possibility of using the xth combinations of clinical procedures; O_x = the xth patient outcome index/indicator)

242x183mm (300 x 300 DPI)

BMJ Open

Pathways and cost-effectiveness of routine lung cancer inpatient care in rural Anhui, China: a retrospective cohort study protocol

Journal:	*BMJ Open*
Manuscript ID	bmjopen-2017-018519.R4
Article Type:	Protocol
Date Submitted by the Author:	19-Jan-2018
Complete List of Authors:	Shen, XingRong; Anhui Medical University Shcool of Health Service Management<br>Diao, MengJie; Anhui Medical University School of Health Service Management<br>Lu, ManMan ; Anhui Medical University School of Health Service Management<br>Feng, Rui; Anhui Medical University, Library Department of Literature Retrieval and Analysis<br>Zhang, PanPan ; Anhui Medical University School of Health Service Management<br>Jiang, Tao ; Anhui Medical University School of Health Service Management<br>Wang, DeBin; Anhui Medical University, School of Health Services Management
**Primary Subject Heading**:	Health economics
Secondary Subject Heading:	Health informatics
Keywords:	cost effectiveness, lung cancer, inpatient care, retrospective study, China

SCHOLARONE™
Manuscripts

Pathways and cost-effectiveness of routine lung cancer inpatient care in rural Anhui, China: a retrospective cohort study protocol

XingRong Shen*, MengJie Diao*, ManMan Lu, Rui Feng, PanPan Zhang, Tao Jiang, Debin Wang#

Corresponding author: Debing Wang.

Post address: 81 Meishan Road, Hefei, Anhui, China. 230032.

Email: dbwang@vip.sina.com

Telephone: (0086) 5116395

XingRong Shen:

School of Health Service Management, Anhui Medical University, Hefei, Anhui, China.

MengJie Diao:

School of Health Service Management, Anhui Medical University, Hefei, Anhui, China.

ManMan Lu:

School of Health Service Management, Anhui Medical University, Hefei, Anhui, China.

Rui Feng:

Department of Literature Review and Analysis, Library of Anhui Medical University, Hefei, Anhui, China.

PanPan Zhang:

School of Health Service Management, Anhui Medical University, Hefei, Anhui, China.

Tao Jiang:

School of Health Service Management, Anhui Medical University, Hefei, Anhui, China.

Word count：4085

For peer review only

ABSTRACT

Introduction: Routine inpatient care (RIC) for cancer patients forms various pathways of clinical procedures. Although most individual procedures comprising the pathways have been tested via clinical trials, little is known about the collective cost and effectiveness of the pathways as a whole. This study aims at exploring RIC pathways for lung cancer patients from rural Anhui, China and their determinants and economic impacts.

Methods and analysis: The study adopts a retrospective cohort design and proceeds in 5 steps. Step 1 defines 4 main categories of study variables including clinical procedures, direct cost and effectiveness of procedures, and factors affecting use of these procedures and their cost and effectiveness. Step 2 selects a cohort of 5000 lung cancer patients diagnosed between July 1, 2014 and June 30, 2015 from rural Anhui by clustered-random sampling. Step 3 retrieves the records of all the inpatient care episodes due to the lung cancer and extracts data about RIC procedures, proximate variables (e.g., Karnofsky performance status, lung function score) of patient outcomes and related factors (e.g., stage of cancer, age, gender) by 2 independent clinician researchers using a web-based form. Step 4 estimates the direct cost of each of the RIC procedures using micro-costing and collects data about ultimate patient outcomes (survival and progression-free survival) through a follow up survey of patients and/or their close relatives. Step 5 analyzes data collected and explores pathways of RIC procedures and their relations with patient outcomes, costs, cost-effect ratios and a whole range of clinical and socio-demographic factors using multivariate regression and path models.

Ethics and dissemination: The study protocol has been approved by authorized ethics committee of Anhui Medical University (reference number: 20170312). Findings from the study will be disseminated through conventional academic routes such as peer-reviewed publications and presentations at regional, national and international conferences.

Trial registry

ISRCTN25595562

Key words: cost effectiveness, lung cancer, inpatient care, retrospective study, China

For peer review only

Strengths and limitations of this study

- The study adopts a retrospective cohort study design involving a large representative sample of community patients;
- It provides data for determining the cost-effectiveness of different treatment approaches as a whole rather than individual procedures;
- It informs our understanding of routine inpatient lung cancer care for rural Chinese, a huge yet understudied population;
- It extracts data from routine records kept at different hospitals and thus suffers from discrepancies in performances and data qualities.

Introduction

Lung cancer has been the most common cancer in the world for several decades.[1] Estimated new cases of the disease was 1.8 million in 2012 (12.9% of all cancers), 58% of which occurred in less developed regions. It was also the most common cause of death from cancer worldwide, being responsible for nearly one in five (1.59 million in absolute number) of the total. [2] In China, lung cancer incidence displayed a slight decreasing trend in the past few years, particularly for males. However, it is still the top first cancer for males and second for females, accounting for 25.2% of all new cancer cases and 29.5% of all cancer deaths in 2012. [3]

Routine inpatient care (RIC) for lung cancer consists of a combination of procedures. Patients with possible lung cancer need a detailed history and physical examination first. Then they should undergo posterior-anterior and lateral chest radiographs as well as CT scans of the chest and abdomen. In order to further confirm and determine stage and histology of the lesion, other diagnostic methods needed include whole-body fluoro-deoxy-glucose positron emission tomography, endoscopic ultrasound, sputum cytology, fine-needle aspiration, bronchoscopy etc. Following diagnosis of lung cancer, the patients proceed with combined-modality therapies depending on stage of the disease and co-morbidities and complications. Historically, surgery provides the best chance for cure for patients whose lung cancers are limited to the hemithorax and can be totally encompassed by excision.[4 5] Surgery has been generally used in combination with external-beam radiotherapy for control of the primary tumor and regional lymphatics.[6] In addition, chemotherapy has also been advocated as an integral part of combined modality approaches to earlier stages of disease. [7 8] For unselected advanced none-small cell lung cancer, platinum-based combinations have become the standard of care; while cisplatin– or carboplatin-based doublets are standard for patients with stage IV disease.[9 10] More recently, EGFR tyrosine kinase inhibitors have been introduced in second- and third-line treatment of advanced disease and in first-line treatment for selected patients.[11]

Given the complex procedures, ensuring quality RIC for lung cancer patients has been most challenging and guidelines are widely used in addressing this challenge. Numerous

For peer review only

studies have documented positive relations between compliance with guidelines and patient outcomes.[12 13] However, researchers have also raised concerns about guidelines. One of such concerns refers to lack of adequate consideration of costs. Most clinical procedures not only affect disease outcomes but also incur considerable costs.[14 15] Yet guidelines are based on trials focused primarily on effectiveness (e.g., survival) with little attention being paid to economic consequences.[16] Another concern relates to incompatible population between clinical trials and RIC. Clinical trials on which guidelines are based use highly selective populations; while RIC serves a general lung cancer population with different age, performance status and comorbidities.[17 18] A third concern revolves uncertain interactions between procedures. Although most individual guideline recommended procedures (GRPs) have established evidences, they are not used in isolation but in conjunction with others forming various clinical combinations. Efforts systematically assessing and comparing these combinations are scarce.[19-22] A fourth concern originates from varied compliance with guidelines since RIC often deviates substantially from guidelines.[23 24] The cost-effectiveness of these "substandard" or mixed combinations of procedures (partly from guidelines, partly from experiences of individual clinicians) falls far from well-understood.[25] These all point to a clear need for evaluating RIC even though guidelines are widely available.

All the above concerns are most pertinent to China. First, China has a unique "dual" medical care system in which patients often receive western medicine and traditional Chinese medicine simultaneously or in turn.[26] Second, China lacks coordinated referral and follow up mechanisms and cancer patients often moves freely from one hospital to another for different rounds of inpatient cancer care.[27] This makes it hard for clinicians in leveraging different inpatient care episodes at different time points and hospitals into continuous and synergetic service. Third, China has strong socio-cultural norms and financial incentives that hinder cost control and guideline compliance.[28]

Study aims

This study aims at identifying main pathways of RIC procedures for lung cancer patients from rural Anhui, China and exploring determinants of the pathways and their economic impacts. Specific questions to be addressed include: a) what combinations of diagnosis and treatment procedures (or pathways for short) an individual patient may experience during all his/her hospitalization episodes due to lung cancer-related problems; b) which are the most and least frequent pathways; c) what determines the flow among these pathways; d) which are the most and least cost-effective pathways in relation to the other pathways; and e) what factors are associated with the relative cost-effectiveness.

The above "pathways" of inpatient care means combinations of diagnosis and treatment procedures an individual patient may experience during all his/her hospitalization episodes due to lung cancer-related problems. Suppose a lung cancer patient experienced

For peer review only

6 times/rounds of hospitalized care and during each of these hospitalization episodes, the patient underwent several diagnosis and treatment procedures, all these procedures form the "pathway" of this particular patient. It is worth noting that findings of the cost-effectiveness analysis are exploratory rather than implying that they are of sufficient robustness to be used to inform policy changes.

Methodology

Identification of procedures

The study uses a self-designed and web-based data extraction form in identifying major clinical procedures described in any RIC record under concern. The form lists all major RIC procedures under two main domains, i.e., diagnostic procedures (e.g., chest X-ray, chest CT, neck ultrasonography) and treatment procedures (e.g., surgical therapy, chemotherapy, psycho-behavioral intervention).

Estimation of costs

The study estimates overall and categorical costs (direct costs only) for each of the RIC procedures (e.g., lung function examination, computed tomography, white blood cell count) identified above using micro-costing techniques.[29 30]Taking the example of lung function examination, categorical costs include costs on personnel, equipment, materials, regents and others needed in completing the examination; while overall cost of the procedure equals the sum of all these categorical costs. In addition, the study also calculates grand total cost on individual inpatient by adding up the overall costs on all the clinical procedures he/she has received.

Measurement of effectiveness

The study uses both proximal variables of outcomes (PV) and ultimate outcome (UO) measures of effectiveness of RIC procedures. The UO indicators derive from a follow up survey about 2 years and half after the first hospitalization and include overall survival (OS), progression-free survival (PFS), quality of life (QoL), and quality adjusted life years (QALYs). Here, OoL is assessed using the widely recognized EQ-5D-5L instrument.[31]

The PV measures come from RIC records and include Eastern Cooperative Oncology Group (ECOG), Karnofsky performance status (KPS) and compiled scores of: a) symptoms (e.g., chronic cough, chest pain, wasting syndrome); b) lung functions (e.g., forced vital capacity, forced one second expiratory volume), c) image findings (e.g., number of nodules identified in the lung, size of the largest nodules, presence of pleura or pericardial effusion). Each of these domain specific PV scores equals weighted sum of all sub-indicators within the domain. For example, the compiled score of "lung functions" equals the sum of weighted values of forced vital capacity, forced one second expiratory volume etc. Here the weights come from the coefficients of multivariate regression

For peer review only

modeling using an UO indicator (e.g., OS) as the dependent variable; while forced vital capacity, forced one second expiratory volume etc. as the independent variables; and stage of disease, age, gender and others as the confounding variables.

Calculation of cost-effectiveness

The study adopts relative cost-effectiveness ratios (RCERs) as the main indicators for measuring cost-effectiveness. Here RCER is defined by the difference in cost between two selected sets of RIC procedures, divided by the difference in their effectiveness. More specifically, $RCER = (C_{r+x} - C_r)/(E_{r+x} - E_r)$, where C_r and E_r is the cost and effectiveness in the reference group and C_{r+x} and E_{r+x}, the cost and effectiveness in the group who have underwent all the procedures in the reference group plus x, a specific procedure under concern.[32] Suppose, x represents a commonly used traditional Chinese medicine (TCM) which incurs 100 dollars; while r, a typical combination of diagnosis and treatment procedures without the TCM. The combination without the TCM costs 1000 dollars and the survival time of patients who have adopted this combination is 1.5 years on average; while the same figure for patients who have used the same combination plus the TCM is 1.51. Then the $C_{r+x} = 1000 + 100 = 1100$ dollars and the RCER of the TCM = (1100-100)/(1.51-1.5)=10000 dollars per life year saved. Similarly, RCER = $(C_{r+x}/E_{r+x})/(C_r/E_r) = (1100/1.51)/\,(1000/1.50) = 1.09$.

Identification of influencing factors

The study also extracts, from RIC records, data about patient factors commonly believed to be linked with disease progression, treatment response and outcomes and utilization of RIC procedures. These include: a) socio-demographics (e.g., age, gender, body height and weight, education, employment, marital status, medical insurance); b) risk behaviors and histories (e.g., smoking, alcohol drinking, history of cancer among family members); c) historical and biological test findings (e.g., value of ALK, KRAS, EGFR, PDL1, CEA, CA125, proGRP); d) comorbidities and complications (e.g., presence of superior vena cava syndrome, brain metastases) and stage of disease. Here, disease staging uses TNM system and this staging will be treated as the most important factor throughout the data analysis especially in its effects on the flow of different pathways and their RCER.

Selection of participants

The study is implemented in Anhui, an inland province located in middle and east China. It has a population of 61.4 million and its per capita GDP and income rank in the middle (the 14th) among all provinces in the nation.[33 34] Its social, cultural and economic background is representative of over 80% of the whole population in China.[33 34] The province has 68 rural counties and each of them divides into 10 to 20 townships. Selection of participating counties, townships, patients and RIC case records uses a clustered random sampling which proceeds in 5 steps. Step 1 classifies all the counties in Anhui into southern, northern and middle areas. Step 2 randomly selects 3 counties from each of these areas (12 counties in total). Step 3 randomly draws 4 townships from each

For peer review only

of the counties selected (48 townships in total). Step 4 searches the provincial reimbursement database of the New Rural Cooperative Medical System (NRCMS) and identifies all the patients within the selected townships who had been first diagnosed with primary lung cancer during July 1, 2015 and June 30, 2016. Step 5 searches the database again for all episodes of hospitalization due to the lung cancer for the patients identified in step 4. NRCMS covers 98% of the rural residents and the estimated number of patients and admission episodes is about 5,000 and 25,000 respectively.

The above sample size was determined by our study purpose of building multivariate models of factors affecting the flow among and RCER of specific RIC pathways. Lung cancer patients generally receive 4 to 6 rounds of inpatient care. Given the various diagnostic and treatment procedures available, there are hundreds of potential RIC pathways (combinations of diagnosis and treatment procedures from the first to the last round of RIC). We plan to group these pathways into manageable (around 20) categories depending on the resultant distribution of the actual pathways and we aim to enter 20-30 factors into the multivariate models for each of these categorical pathways. Based on these pre-conditions and that the sample size of a multi-variable model should generally be 10 times the number of independent variables, we need 250 patients for each pathway. This translates into 5000 patients in total.

Data collection

The study obtains data through follow-up survey and data extraction. The follow-up survey applies to all the lung cancer patients identified above. It solicits information about the patient's: a) disease progression (i.e., died, alive with or without progression); b) if died, date of death; c) additional admissions due to the lung cancer not included in the above mentioned NRCMS database. The survey uses a short structured questionnaire. Administration of the questionnaire starts with a telephone interview (of the patient under concern or his/her close relatives for up to 5 time attempts) followed by a face-to-face interview (of the same respondents for up to 2 attempts) if the telephone contacts have failed. The recruitment strives to reach over 85% rate of participation. The researchers are trained to record reasons of attrition for each of the patients they have lost so as to allow for assessing potential biases. The data extraction applies to records of all the hospital admission episodes identified via the NRCMS database and the follow up survey. It uses a structured web-based form and extracts data about the clinical procedures, costs, effectiveness and influencing factors described above. Two experienced clinicians on care of lung cancer perform the data extraction. They visit (on one-by-one base) all the relevant hospitals, ask for permission to examine the full records and fill the worksheet independently first followed by discussions, if applicable, to solve discordances.

Data analysis

The data collected above allow a variety of descriptive and multivariate analysis concerning the costs and effectiveness of RIC. The effectiveness analysis comprises all

For peer review only

the UO indicators including progression free survival, overall survival, quality of life and DALYs. For each of these UO indicators, the analysis will produce: a) estimation of average rates or values with 95% confidence intervals at different time points after first diagnosis by disease stage, PV indicators, RIC pathways, non-hospital care categories, age range etc.; b) multivariate regression models using similar variables as independent variables; and c) path models using disease stage, RIC pathways, non-hospital care categories, age range etc. as exogenous, complied PV indices as direct endogenous, and individual PV indicators as indirect endogenous variables (Figure 1a). Area under ROC (receiver operating characteristic) curve will be estimated for assessing the predictability of models using binary classifier as the dependent variable (e.g., models of progression free survival, overall survival).

The cost analysis explores mainly: a) Markov models of mean cost for managing lung cancer patients (Figure 1b); b) overall and categorical costs on different rounds of hospitalization by socio-demographic and selected clinical conditions (Figure 2); c) scatter plot of RIC procedures using the occurrence rate and unit cost of individual procedures as the coordinates; and d) multivariate regression models of overall and selected categorical costs using disease stage, PV indicators, RIC pathways, non-hospital care categories, age range etc. as independent variables.

The cost-effectiveness analysis focuses primarily on constructing a pathway tree to help estimate expected overall and pathway specific cost, effectiveness and identify pathways with the highest or lowest RCER. The tree consists of different branches of combinations of RIC procedures starting from the first to the last episode of inpatient care labeled with estimated costs and possibilities along the pathways and outcomes at the end of the pathways (Figure 3). Relevance of the pathway tree is tested by means of, for instance, varying the percentage of patient flowing among the different pathways or the costs of major diagnostic and treatment procedures consisting the braches and then examining changes in the ranking of the pathways in terms of relative cost-effectiveness. The analysis also pays particular attention to identifying as many as comparable pairs of RIC pathways as possible and calculating RCER accordingly in a hope to uncover potential pathways of practice, policy and research implications.

The pathway tree construction will use TreeAge[35]; while the descriptive and multi-variate model analysis, SPSS 16. Cases with missing data about a specific item will be excluded from the analysis involving the item and where applicable, the statistical null hypothesis is be rejected at the significance level of $\alpha = 0.05$.

Ethics and dissemination

The study protocol had been reviewed and approved by the Biomedical Ethics Committee of Anhui Medical University (reference number: 20170312). Participation of hospitals, patients and their relatives are voluntary and written informed consent is

For peer review only - http://bmjopen.bmj.com/site/about/guidelines.xhtml

required for all participants. Findings from the study will be disseminated through conventional academic routes such as peer-reviewed publications and presentations and regional, national and international conferences.

Discussion

The study would share the experience of lung cancer care from the rural Chinese perspective. It is an important sharing of knowledge on population-based lung cancer care, since most economic evidence comes from Europe and North America. In China, traditional Chinese medicine is used to complement or replace western medicine. This results in quite different pathways of lung cancer care that have seldom been well explored in published literatures. China has a long history of almost no charges being made for clinical consultations and most patients are used to paying only for medicines, laboratory tests and equipment-based examinations. This forms a perverse financial incentive for clinicians to order more sophisticated examinations and tests and to over prescribing. China's lack of referral and follow up mechanisms also merits particular attention. As an individual patient changes from one hospital (say for the first round of treatment) to another (for the second round treatment), he/she may receive different treatment regimens. Discontinued treatment and follow up may make it hard for clinicians to base their treatment decisions on observed effects.

Perhaps the most noteworthy findings of the current study may be the description of the pathways of RIC procedures and their economic impacts (Figure 2). These pathways will provide easily understandable means for estimating and identifying, among others, the following: a) which pathways or combinations of procedures happen most or least in routine practice during different rounds of hospitalization for inpatients suffering from lung cancer in rural China; b) which pathways (from the first to last round of hospitalization) incur the highest or lowest direct costs; and c) which pathways result in the best or worst patient outcome in terms of different UO measures. These have important implications for clinical decision-making as well as policy-making.

Another point worth mentioning refers to the links between the domain specific proximate (PV) indices to key ultimate outcome (UO) indicators (e.g., OS, PFS, QALYs) generated via a large scale (involving 5000 lung cancer patients) retrospective cohort study. They provide useful information for clinicians on care of lung cancer patients in selecting appropriate procedures to achieve optimal collective contributions to UO.[36] At present, although PV indicators are observed routinely, they are presented to clinicians as individual indicators rather than compiled indices. Given the large number of PV indicators involved and the complex relations between RIC procedures and PV indicators and then UO indicators, it is difficult for practicing clinicians to make balanced decisions upon their personal experiences.[37]

For peer review only

In addition, this study addresses RIC for lung cancer at hospitals in China from a range of meaningful perspectives. The study reinforces the concepts introduced in the landmark studies of Fisher et al and Wennberg et al, which convincingly demonstrated that high quality was not necessarily associated with high cost.[38] Describing inpatient lung cancer care in a view that its value is directly proportional to outcomes and inversely proportional to costs helps in guiding quality improvement by either better outcomes and/or lower costs. [39] The study calculates and compares the collective costs and effectiveness of different RIC pathways as a whole and thus informs coordinated inpatient care episodes and procedures at different time points and hospitals. The study enables RCER estimation for specific guideline recommended procedures (GRPs) using various combinations of real and uncontrolled RIC procedures as the reference and thus enhances understanding and application of GRPs established through well-controlled studies.

The study also has limitations. The first limitation concerns data reliability. Although the majority of data are extracted from RIC records kept at hospitals, the study uses self-reported data about quality of life and inpatient, outpatient and home care. Self-reports are prone to various biases including recall problems particularly among the elderly, over or under reporting by the respondents for reasons like perceived expectations from the researchers or for fearing of potential worries or distress. These biases may be reduced to a minimum in our study by means of interviewer training, use of chorological recall and probing techniques, and cross-checks of findings from patient interviews, health insurance database and hospital records. More importantly, the study uses EQ-5D-5L in assessing quality of life. It has already been tested with adequate reliability both internationally and in China. Regarding non-hospitalized care, the study asks only simple questions about what kind of care the patients have experienced and when and for how long. These questions are relatively memorable and easy to answer. The second limitation relates to selective study content. The study considers only inpatient care; while patients may use various self-treatment and outpatient treatment in addition to inpatient care.[40 41] Inpatient and non-inpatient treatment may substitute each other to some extent. These may result in under-estimation of the effectiveness of RIC procedures. Fortunately, this under-estimation may be offset to a large extent by treating non-hospital care as confounders and the study data to be collected allow this exercise. Third, the study considers only direct costs rather than full costs taking both direct and indirect costs into consideration. In addition, different hospitals use different equipment, reagents and medicines. Their quality of records may also vary substantially. These raise compatibility concerns in pooling data from different hospitals together and performing aggregate analysis. Finally, readers may raise concerns about representativeness of inpatients to the larger cancer patients. Hospitalization rates documented from other countries varied greatly; [42] while similar data from China are scarce. Our estimation, using the dataset of the last province-wide Household Health Survey of Anhui, of the

For peer review only

proportion of lung cancer patients who had been admitted to hospitals at least once was as high as 89%.[43]

Competing interests

The authors declare no competing interests.

Authors' contributions

XS and MD contributed equally in conceiving this project, facilitating protocol and instrument development, and drafting this manuscript. RF, ML, PZ and TJ are kore researchers for cost estimation, record extraction, follow up survey and data analysis respectively. DW provided expertise for overall design of the study, and revised and finalized the manuscript. All authors have read and approved the final submission.

Acknowledgements

Development of the primitive protocol was supported by the Natural Science Foundation of China (grant number: 71503009). Refinement and implementation of the protocol is lead and supported by Collaboration Center for Cancer Control of Anhui Medical University, Anhui and Luan Center for Diseases Control and Prevention.

References

1. McErlean A, Ginsberg MS. Epidemiology of lung cancer. *Semin Roentgenol* 2011;46 (3):173-7.
2. World Health Organization. GLOBOCAN 2012: Estimated Cancer Incidence, Mortality and Prevalence Worldwide in 2012. http://globocan.iarc.fr/Default.aspx.
3. Kong J, Xu F, He M, Chen K, et al. The incidence of lung cancer by histological type: a population-based study in Tianjin, China during 1981-2005.*Respirology* 2014;19(8):1222-8.
4. Woodard GA, Jablons DM. The Latest in Surgical Management of Stage IIIA Non-Small Cell Lung Cancer: Video-Assisted Thoracic Surgery and Tumor Molecular Profiling. *Am Soc Clin Oncol Educ Book* 2015;35:e435-41.
5. Grunenwald DH. The role of surgery in non-small-cell lung cancers. *Ann Oncol 2005*;16 Suppl 2:ii220-2.
6. Ricardi U, Badellino S, Filippi AR. Stereotactic radiotherapy for early stage non-small cell lung cancer. *Radiat Oncol J*. 2015;33(2):57-65.
7. Mangal S, Gao W, Li T1, Zhou QT. Pulmonary delivery of nanoparticle chemotherapy for the treatment of lung cancers: challenges and opportunities. *Acta Pharmacol Sin*. 2017. doi: 10.1038/aps.2017.34.
8. Khan I, Morris S, Hackshaw A, et al. Cost-effectiveness of first-line erlotinib in patients with advanced non-small-cell lung cancer unsuitable for chemotherapy. *BMJ Open* 2015; 5(7):e006733.

For peer review only

9. Garon EB, Ciuleanu TE, Arrieta O, et al. Ramucirumab plus docetaxel versus placebo plus docetaxel for second-line treatment of stage IV non-small-cell lung cancer after disease progression on platinum-based therapy (REVEL): a multicentre, double-blind, randomised phase 3 trial. *Lancet* 2014;384(9944):665-73.
10. Spigel DR, Luft A, Depenbrock H, et al. An Open-Label, Randomized, Controlled Phase II Study of Paclitaxel-Carboplatin Chemotherapy With Necitumumab Versus Paclitaxel-Carboplatin Alone in First-Line Treatment of Patients With Stage IV Squamous Non-Small-Cell Lung Cancer. *Clin Lung Cancer* 2017; pii: S1525-7304(17)30045-1.
11. Stinchcombe TE. The Use of EGFR Tyrosine Kinase Inhibitors in EGFR Wild-Type Non-Small-Cell Lung Cancer. *Curr Treat Options Oncol* 2016; 17(4):18.
12. Ahmed HZ, Liu Y, O'Connell K, et al. Guideline-concordant Care Improves Overall Survival for Locally Advanced Non-Small-cell Lung Carcinoma Patients: A National Cancer Database Analysis. *Clin Lung Cancer* 2017; pii: S1525-7304(17)30114-6.
13. Nadpara P, Madhavan SS, Tworek C. Guideline-concordant timely lung cancer care and prognosis among elderly patients in the United States: A population-based study. *Cancer Epidemiol*; 39(6):1136-44.
14. Hinde S, McKenna C, Whyte S, et al. Modelling the cost-effectiveness of public awareness campaigns for the early detection of non-small-cell lung cancer. *Br J Cancer* 2015; 113(1):135-41.
15. Kumar G, Woods B, Hess LM, et al. Cost-effectiveness of first-line induction and maintenance treatment sequences in non-squamous non-small cell lung cancer (NSCLC) in the U.S. *Lung Cancer* 2015. pii: S0169-5002(15)00281-0.
16. Warren JL, Harlan LC, Trimble EL, et al. Trends in the receipt of guideline care and survival for women with ovarian cancer: A population-based study. *Gynecol Oncol* 2017;145(3):486-492.
17. Jennens RR, Giles GG, Fox RM. Increasing underrepresentation of elderly patients with advanced colorectal or non-small-cell lung cancer in chemotherapy trials. *Int Med J* 2006;36: 216e220.
18. Murthy VH, Krumholtz HM, Gross CP. Participation in cancer clinical trials; race-, sex-, and age-based disparities. *JAMA* 2004;22(291): 2720-2726.
19. Tong Y, Huang C, Zhang J. A novel EGFR-TKI inhibitor (cAMP-H3BO3 complex) combined with thermal therapy is a promising strategy to improve lung cancer treatment outcomes. *Oncotarget.* Published Online First: 04 May 2017.doi: 10.18632/oncotarget.17628.
20. National Health and Family Planning Commission of China. Guideline for Chinese primary lung cancer diagnosis and treatment (2015 edition). *Chinese Journal of Oncology* 2015.37(1):67-78.
21. Xing Wang, Shi Yan, Yaqi Wang, et al.Surgical Quality Surveillance and Sustaining Improvement of Lung Cancer Surgery Based On Standard Operation Procedure(SOP) : Experience of Single Surgical Team. *Chinese Journal of Lung Cancer;* 20(4): 253-258.
22. Jackman DM, Zhang Y, Dalby C, et al. Cost and Survival Analysis Before and After Implementation of Dana-Farber Clinical Pathways for Patients With Stage IV Non-Small-Cell Lung Cancer. *J Oncol Pract* 2017;13(4):e346-e352.
23. Okita A, Yamashita M, Abe K, et al. Variance analysis of a clinical pathway of video-assisted single lobectomy for lung cancer. *Surg Today* 2009;39(2):104-9.

For peer review only

24. Duggan KJ, Descallar J, Vinod SK. Application of Guideline Recommended Treatment in Routine Clinical Practice: A Population-based Study of Stage I-IIIB Non-small Cell Lung Cancer. *Clin Oncol (R Coll Radiol)* 2016;28(10):639-47.
25. Heins MJ, de Jong JD, Spronk I, et al. Adherence to cancer treatment guidelines: influence of general and cancer-specific guideline characteristics. *Eur J Public Health* 2016; pii: ckw234. doi: 10.1093/eurpub/ckw234.
26. Yang S, Cui M, Li HY, et al. Meta-analysis of the effectiveness of Chinese and Western integrative medicine on medium and advanced lung cancer. *Chin J Integr Med* 2012;18(11):862-7.
27. XX L, YW Chen, KS Bi. Resolution of Two- way Recerral Problem in China by Studying British National Health Service System. *Chinese General Practice* 2013; 31(16):2926-29.
28. Y Sun, J Wu, SB Xie, et al. Evaluation of the medical staff clinical pathway adherence: Based on comparison of before and after provider payment reform in Henan Province. *Chinese Journal of Health Policy* 2013;6(5):37-43.
29. Henry SG, Ness RM, Stiles RA, Shintani AK, Dittus RS. A cost analysis of colonoscopy using microcosting and time-and-motion techniques. *J Gen Intern Med* 2007, 22(10):1415-21.
30. Cressman S, Lam S, Tammemagi MC,et al. Resource utilization and costs during the initial years of lung cancer screening with computed tomography in Canada. *J Thorac Oncol* 2014, 9(10):1449-58.
31. Khan I,Morris S, Pashayan N, et al. Comparing the mapping between EQ-5D-5L, EQ-5D-3L and the EORTC-QLQ-C30 in non-small cell lung cancer patients. *Health Qual Life Outcomes* 2016, 14:60.
32. SN York. Incremental Cost-Effectiveness Ratio. *Handbook of Disease Burdens and Quality of Life Measures* 2010: 4235-4235.
33. National Bureau of Statistics of China. China Statistical Yearbook 2016. http://www.stats.gov.cn/tjsj/ndsj/2016/indexch.htm (accessed 1 July 2017).
34. Statistics Bureau of Anhui Province. Statistical yearbook of Anhui Province in 2016. http://www.ahtjj.gov.cn/tjj/web/tjnj_view.jsp?strColId=13787135717978521&_index=1# (accessed 1 July 2017).
35. Treeage: Hollman C, Paulden M, Pechlivanoglou P, et al. A Comparison of Four Software Programs for Implementing Decision Analytic Cost-Effectiveness Models. *Pharmacoeconomics* 2017. doi: 10.1007/s40273-017-0510-8.
36. Darling G, Malthaner R, Dickie J, et al. Quality indicators for non-small cell lung cancer operations with use of a modified Delphi consensus process. *Ann Thorac Surg* 2014;98(1):183-90.
37. Fisher A, Manicavasagar V, Sharpe L, et al. A Qualitative Exploration of Clinician Views and Experiences of Treatment Decision-Making in Bipolar II Disorder. *Community Ment Health J.* Published Online First: 19 Jan 2017. doi:10.1007/s10597-016-0077-4.
38. Wennberg JE, Fisher ES. Finding high quality, efficient providers for value purchasing: cohort methods better than methods based on events. Finding high quality, efficient providers for value purchasing: cohort methods better than methods based on events. *Med Care* 2002,40(10):853-5.
39. Chen LW, Wilson FA, Gregg A, et al. Measuring the Cost and Value of Quality Improvement Initiatives for Local Health Departments. J Public Health Manag Pract.

For peer review only

Published Online First: 1 Mar 2017. doi: 10.1097/PHH.0000000000000552.

40. O' Regan P, Hegarty J. The importance of self-care for fatigue amongst patients undergoing chemotherapy for primary cancer. *Eur J Oncol Nurs* 2017;28:47-55.
41. Dionne-Odom JN, Demark-Wahnefried W, Taylor RA, et al. The self-care practices of family caregivers of persons with poor prognosis cancer: differences by varying levels of caregiver well-being and preparedness. *Support Care Cancer* 2017; 25(8):2437-2444.
42. Prince RM, Atenafu EG, Krzyzanowska MK. Hospitalizations During Systemic Therapy for Metastatic Lung Cancer: A Systematic Review of Real World vs Clinical Trial Outcomes. JAMA Oncol 2015, 1(9):1333-9.
43. Zhao T, Cheng J, Chai J, et al. Inpatient care burden due to cancers in Anhui, China: a cross-sectional household survey. *BMC Public Health* 2016, 16:308.

For peer review only

Figure 1 Schematic structure of sample multivariate models to be built

Figure 2 Simulated cost by selected socio-demographics and clinical characteristics (TC=total cost; KRMB=1000 Chinese yuan)

Figure 3 Anticipated "procedure-outcome" tree of inpatient lung cancer care (Tx = the xth round of hospitalization; Cx = the xth combination of clinical procedures; Px = possibility of using the xth combinations of clinical procedures; Ox = the xth patient outcome index/indicator)

For peer review only

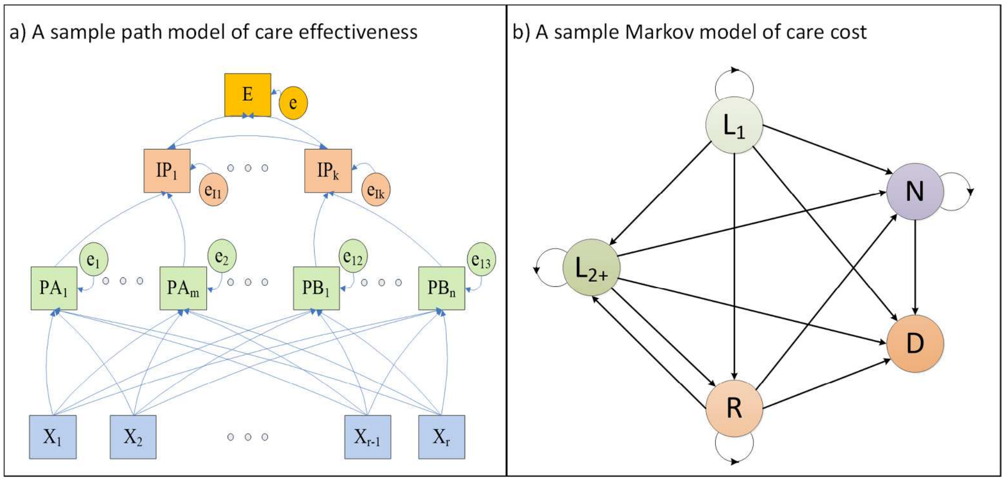

Figure 1 Schematic structure of sample multivariate models to be built/ X=independent variables; PA or PB=domain A or proximate indicators of effectiveness; IP=index of proximate variables; e=systematic error; and E= effectiveness, e.g., overall survival, QALYs; L_1=first line treatment; L_2+=second or third line treatment; R=remission; N=no active treatment; D=death.

188x88mm (300 x 300 DPI)

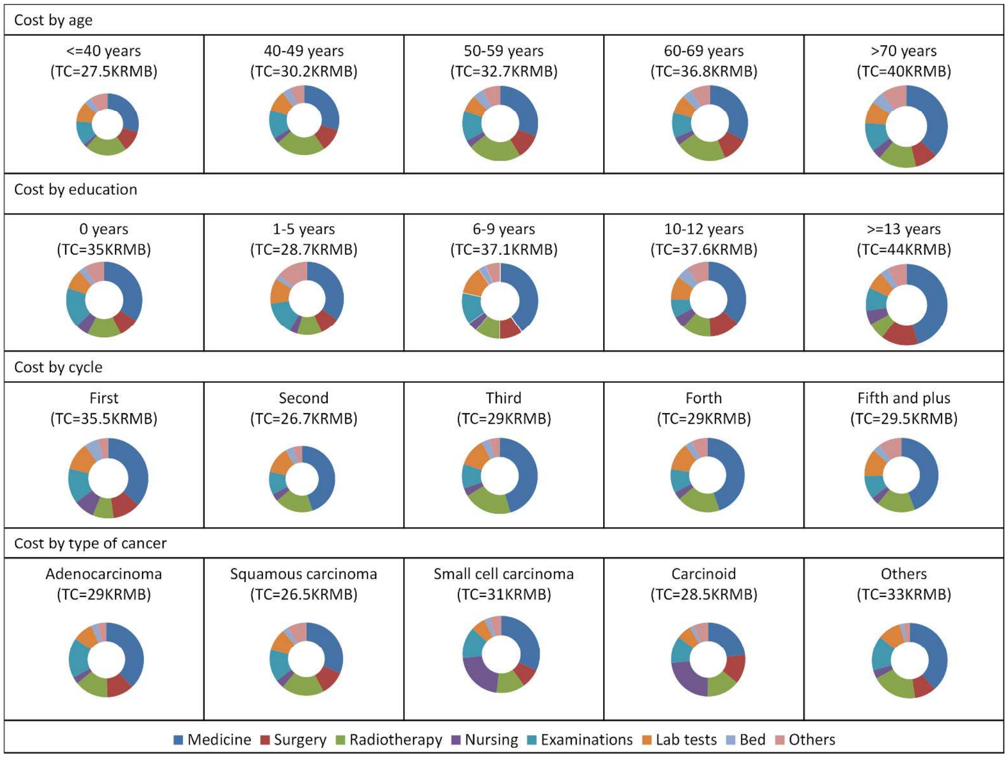

Figure 2 Simulated cost by selected socio-demographics and clinical characteristics (TC=total cost; KRMB=1000 Chinese yuan)

249x187mm (300 x 300 DPI)

For peer review only

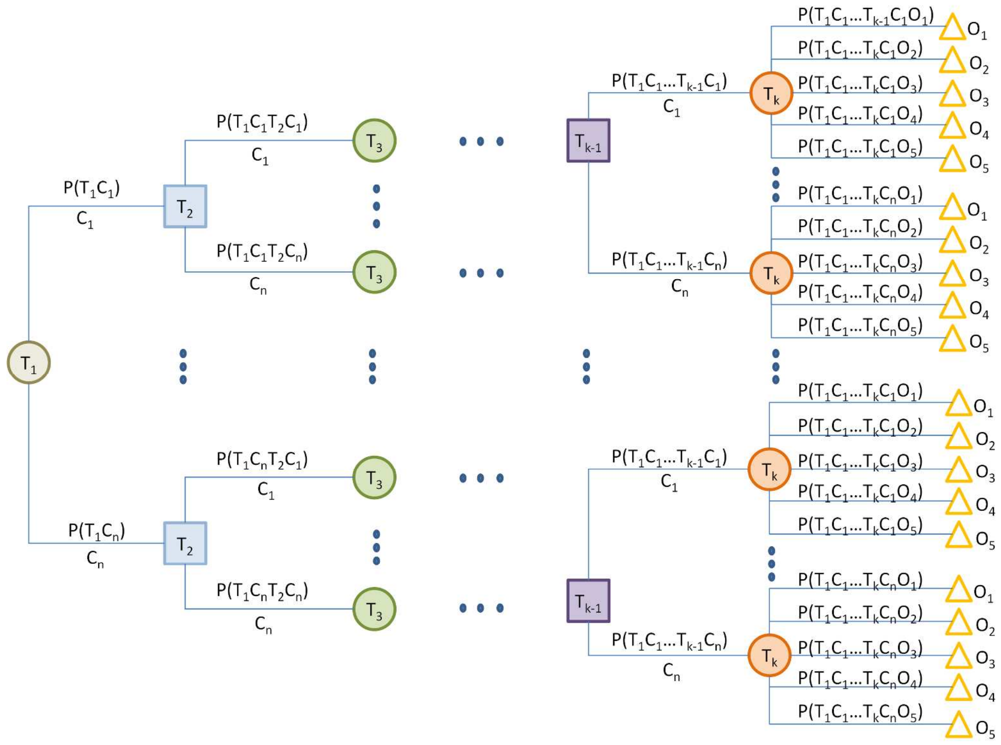

Figure 3 Anticipated "procedure-outcome" tree of inpatient lung cancer care (T_x = the xth round of hospitalization; C_x = the xth combination of clinical procedures; P_x = possibility of using the xth combinations of clinical procedures; O_x = the xth patient outcome index/indicator)

242x183mm (300 x 300 DPI)

BMJ Open

Is it feasible to conduct a randomised controlled trial of pre-transplant exercise (pre-habilitation) for multiple myeloma patients awaiting autologous haematopoietic stem cell transplantation? (PREeMPT study).

Journal:	*BMJ Open*
Manuscript ID	bmjopen-2017-021333
Article Type:	Protocol
Date Submitted by the Author:	10-Jan-2018

Complete List of Authors:	Keen, Carol; Sheffield Teaching Hospitals NHS Foundation Trust, Acute Therapy Services<br>Skilbeck, Julie; Sheffield Hallam University, Nursing<br>Ross, Helen; Sheffield Teaching Hospitals NHS Foundation Trust, Acute Therapy Services<br>Smith, Lauren; Sheffield Teaching Hospitals NHS Foundation Trust, Acute Therapy Services<br>Collins, Karen; Sheffield Hallam University, Centre for Health and Social Care Research<br>Dixey, Joanne; Sheffield Teaching Hospitals NHS Foundation Trust<br>Walters, Stephen; University of Sheffield, ScHARR<br>Greenfield, Diana; Sheffield Teaching Hospitals NHS Foundation Trust<br>Snowden, John; Sheffield Teaching Hospitals NHS Foundation Trust, Department of Haematology<br>Mawson, Susan; University of Sheffield, School of Health and Related Research

Keywords:	Myeloma < HAEMATOLOGY, Bone marrow transplantation < HAEMATOLOGY, Rehabilitation medicine < INTERNAL MEDICINE

SCHOLARONE™
Manuscripts

Title: **Is it feasible to conduct a randomised controlled trial of pre-transplant exercise (pre-habilitation) for multiple myeloma patients awaiting autologous haematopoietic stem cell transplantation? (PREeMPT study).**

FUNDING: This is an original research study funded by NIHR Research for Patient Benefit (RfPB) funding

AUTHORS: Carol Keen[(1)], Julie Skilbeck[(2)], Helen Ross[(1)], Lauren Smith[(1)], Karen Collins[(2)], Joanne Dixey[(1)], Stephen Walters[(3)], Diana M Greenfield[(1,3)], John A Snowden[(1,3)], Sue Mawson[(1,3)]

(1) Sheffield Teaching Hospitals NHS Foundation Trust
(2) Sheffield Hallam University
(3) University of Sheffield

CORRESPONDING AUTHOR:
Carol Keen - carol.keen@sth.nhs.uk

For peer review only

Abstract

Introduction:

While myeloma is an incurable malignancy, developments in disease management have led to increased life expectancy in recent years. Treatment typically involves stem-cell transplantation. Increased survival rates equates to more patients living with the burden of both the disease and its treatment for increasing numbers of years, rendering myeloma a long term condition.

Evidence exists to demonstrate the benefits of exercise for patients recovering from stem cell transplantation, and pre-habilitation – exercise before treatment - has been shown to be effective in other disease areas. To date there has been no research into pre-habitation in myeloma patients awaiting transplantation treatment.

Our objective is to determine whether it is feasible to conduct a randomised controlled trial into pre-transplant exercise for patients with multiple myeloma who are awaiting autologous stem cell transplantation.

Methods and analysis:

This mixed methods study identifies patients with diagnosis of multiple myeloma who have been assigned to the autologous transplantation list and invites them to participate in 6 weekly sessions of individualised, supervised exercise whilst awaiting transplantation.

Quantitative data to determine feasibility targets include rates of recruitment, adherence and adverse events, and outcome measures including minute walking distance (MWD) test and quality of life.

Qualitative interviews are undertaken with a purposive sample of patient to capture their experiences of the study and the intervention.

Ethics and dissemination:

Ethics committee approval has been obtained. Dissemination will be through open-access publication and presentation and will seek to reach multi-professional bases as well as patient and carer groups, addressing the widespread interest in this area of research.

The study is registered in the clinical trials registry at https://clinicaltrials.gov/show/NCT03135925.

Strengths and limitations

- To the best of our knowledge this will be the first research of its kind
- It will provide evidence of the acceptability of pre-habilitation to patients with myeloma and the potential for future studies
- It will not provide evidence of the effectiveness of pre-habilitation, but will inform future study design for evaluating effectiveness

Introduction

Myeloma is an incurable malignancy of antibody producing B lymphocytes and plasma cells. Equating to 7 new cases per 100,000 population in the UK, it represents 10% of all new haematological cancers.[1] Disease symptoms include anaemia and hypercalcaemia causing fatigue and weakness, immunosuppression and lytic lesions of bone increasing pathological fracture risk.[2]

Due to developments in disease management, life expectancy has increased significantly in the last 10 years.[3] The 5 year relative survival rate for England was 42.2% in 2011,[4] and is set to increase further due to earlier interventions in the disease process, more effective chemotherapies and increased use of autologous stem cell transplantation.[5]

Following diagnosis of multiple myeloma, the standard of care treatment for younger patients (generally, but not exclusively, under the age of 70) with adequate fitness consists of an intensive pathway commencing with induction treatment using a variety of regimens delivered as an outpatient or day case given to control disease until maximum response is achieved (usually reflected by a plateau in serum paraprotein).[6–8] This response is then consolidated with autologous

For peer review only - http://bmjopen.bmj.com/site/about/guidelines.xhtml

For peer review only

stem cell transplantation which permits the administration of high dose myeloablative melphalan chemotherapy, a procedure typically requiring around 3 weeks inpatient care, after which patients take several months to make a functional recovery.[6–8] The procedure is non-curative and relapse/progression of myeloma occurs after an average of 2-3 years, which requires re-institution of induction treatment, and, in many patients, consolidation with a second autologous transplant procedure.[9,10]

Rationale for the study

Increased survival rates equates to more patients living with the burden of both the disease and its treatment for increasing numbers of years, rendering myeloma a long term condition.[11] The cumulative effects of the disease, compounded with the debilitating toxic nature of the treatment, impact significantly on quality of life for patients beyond the end of treatment, with late-effects symptoms including infection, fatigue, metabolic, neurological and cardiovascular disorders, as well as pain, physical fitness and psychological concerns.[12]

Only 20% of myeloma patients meet national physical activity guidelines post-treatment[12] and activity declines through treatment due to perceived barriers to exercise including pain, fear of injury and fatigue.[13] Although research evidence in physical activity has been demonstrated to be limited, [15] evidence exists to demonstrate the benefits of exercise for patients recovering from stem cell transplantation.[14] Pre-habilitation after treatment in myeloma patients has been shown to improve symptoms of physical performance, muscle strength, aerobic capacity, psychological outcomes immunological function and fatigue.[16] Exercise training for myeloma survivors has been shown to be safe and feasible during treatment with high attendance and adherence[17] and has been implement widely in clinical practice.

Studies demonstrate that pre-transplant patients have reduced exercise capacity and increased co-morbidities compared with a normal population, yet most rehabilitative interventions occur during and after treatment.[14] Thus while exercise rehabilitation after treatment for myeloma can be effective, we must also consider rehabilitative interventions prior to the start of treatment: pre-habilitation, defined as,

> *"a process on the continuum of care that occurs between the time of cancer diagnosis and the beginning of acute treatment … provides targeted interventions that improve a patient's health to reduce the incidence and the severity of current and future impairments".*[18]

Examples of pre-habilitation exist in other clinical specialties: it has been used for some time in orthopaedic surgery to improve outcomes and postoperative recovery,[19] and its economic benefits have been demonstrated within colorectal surgery.[20] A review of pre-habilitation in pre-surgical cancer patients demonstrated the effective use of aerobic interventions in the management of patients undergoing thoracic surgery for lung cancer, identified the potential for its use in other oncology settings and called for further research to evaluate pre-habilitation for wider groups of cancer patients.[19]

Guidelines for the management of late and long terms effects of myeloma recommend that regular physical activity, including pre-habilitation and rehabilitation, and aspiration to a general healthy lifestyles, are integral to patient care pathways.[12]

Autologous stem cell transplantation in myeloma has become the commonest indication for transplantation, with, for example, over 1400 performed in the UK annually, and procedures are performed in what is normally considered an elderly patient population, many with comorbidities and frailty. It is an intensive toxic procedure, with a recovery period of at least 6 months and strategies to improve recovery are warranted, including pre-habilitation. A window of opportunity – usually a period of 4-6 months exists to offer pre-habilitation between diagnosis or relapse and the commencement of the autologous stem cell transplantation process. Coleman et al.[21] studied 24 multiple myeloma patients undergoing a home based exercise program during chemotherapy and stem cell transplantation and identified that no patient injured themselves and that the intervention

For peer review only - http://bmjopen.bmj.com/site/about/guidelines.xhtml

had positive effects on lean body weight, fatigue and sleep disturbance. Despite this, no evidence currently exists regarding the use of pre-habilitation exercise interventions in multiple myeloma.

This article describes the protocol for a study underway investigating the feasibility of research into the provision of an exercise intervention in patients with myeloma who are due to receive autologous stem cell transplantation.

Aims and Objectives

The aim of this study is to determine whether it is feasible to conduct a randomised controlled trial into pre-transplant exercise for patients with multiple myeloma who are awaiting autologous stem cell transplantation.

We will determine this through completion of the following objectives:

1. Assess the acceptability of the study to patients by measuring recruitment and retention to the study and through qualitative interview responses
2. Explore reasons for non-consent to study participation
3. Establish whether a target cohort of patients exists.
4. Determine the most appropriate recruitment points post diagnosis through steering group feedback, recruitment rate when compared with numbers invited to join the study and qualitative interview reports
5. Assess the suitability of inclusion and exclusion criteria by examining recruitment data
6. Assess the acceptability of the intervention through qualitative interviews and retention rates during the study
7. Determine duration of the intervention before transplantation commences by monitoring point of recruitment to the study and time to transplant
8. Explore the appropriateness of outcome measures/completeness by qualitative interview responses, completion rates, time to complete.

Methods and Analysis

Methodology

Mixed methods, combining qualitative and quantitative data collection and analysis, are used to achieve the described aims and objectives.

Design

This is a prospective feasibility study – see Figure 1 for study flow chart.

Setting

Assessments and exercise sessions take place in the physiotherapy outpatient department in an acute hospital trust, which is a regional specialist centre for haematological services. Patient interviews take place in private rooms in the physiotherapy department or over the telephone for patient convenience.

Feasibility

The feasibility of the intervention is determined through the following targets:

- Recruitment: based on patient numbers at the study site, the recruitment target is 24 patients in a 12 month period (i.e. 2 patients per month);
- Attendance: minimum average attendance at exercise sessions of 66% of the scheduled/invited sessions;
- Retention: 80% patient retention to 6-week follow up assessment;
- Adverse events: adverse events are closely monitored and use to inform decisions to proceed.

Acceptability of the intervention to patients is also determined through the qualitative data collection and analysis, described in a later section.

For peer review only - http://bmjopen.bmj.com/site/about/guidelines.xhtml

Quantitative Data Collection and Analysis

Data collection will take place between September 2016 and February 2018.

Sampling

Consecutive sampling is used to recruit patients to this study who have a diagnosis of multiple myeloma and have been assigned to the autologous transplantation list. The recruiting centre transplants approximately 70 myeloma patients per year: sampling all patients over a 12 month period will indicate study recruitment feasibility. This feasibility study did not have a formal sample size calculation to determine a priori the number of participants to recruit; it aimed to recruit for a fixed period of time (12 months) at a single centre and one of the outcomes was to estimate the recruitment rate per month.

Inclusion criteria

All patients with a diagnosis of multiple myeloma, assigned to the autologous transplantation waiting list for either a first or second transplant.[22]

Exclusion Criteria

To allow safe completion of initial objective assessments, patients with a history of unstable angina or heart attack in the previous month are excluded.[23] Medical stability is a pre-requisite for transplantation, therefore no patients are excluded on this basis.

Recruitment

Patients are screened at clinic appointments by the bone marrow transplant team during their preparation for transplant. Patients meeting the inclusion criteria are provided with verbal and written information and invited to be involved in the study. Follow-up takes place after 48 hours via a phone call from a study physiotherapist: any remaining questions are discussed and if the patient agrees to take part then written consent is obtained and an initial assessment appointment is made.

Patients who choose not to join the study are invited to take part in a qualitative interview to explore their reasoning (Figure 1). This is described in more detail under Qualitative Data Collection and Analysis.

Intervention:

Initial Assessment

Patients attend an initial assessment with a study physiotherapist who undertakes the following:

- explanation of the pre-habilitation programme
- documentation of written consent
- subjective history including co-morbidities and patient goals
- induction to the gym area equipment
- provision of booklet and DVD with physical activity advice
- baseline objective assessment (Table 1)
- design of individualised gym program in line with patient abilities and goals
- completion of an initial gym circuit with close supervision.

Weeks 2-5

Patients attend weekly 1 hour physiotherapist-led group gym sessions and complete their individualised program. Supervision is available as required and programs are progressed in line with patient ability and performance.

Week 6

Completion of final gym circuit and repeat of objective assessments (Table 1).

Follow up

Patients are followed up on admission for transplant, and again on transplant discharge, for further repeat of objective assessments (Table 1).

	Recruitment	Initial Assessment	Weeks 2-5	Week 6	Transplant Admission	Transplant Discharge

For peer review only - http://bmjopen.bmj.com/site/about/guidelines.xhtml

Screening data	✓					
Demographic data		✓				
6 minute walk distance		✓		✓	✓	✓
PROMs		✓		✓	✓	✓
Activity data		✓	✓	✓	✓	✓
Adverse Events		✓	✓	✓		

Table 1 - Study Data Collection

Outcome measures

The following data are captured for study participants.

Screening Data

Through initial screening and recruitment, data is collected on:

- number of patients meeting inclusion criteria
- patients accepting initial study information
- patients agreeing to attend for initial assessment
- reasons for non-participation.

Demographic data

The following demographic data is captured during the initial assessment:

- gender
- length of diagnosis
- baseline physical activity levels
- transplant history
- pre-transplant therapies received
- time to transplantation from decision to transplant
- other relevant information.

Functional measure

Patients undertake a 6 minute walk test (6MWD) before and after the exercise intervention. The six minute walk test is a useful field test of functional capacity, is safe to administer and although it has less correlation with peak oxygen capacity than the shuttle walk test, it is better tolerated by patients and is more reflective of activities of daily living as it is a submaximal exercise test.[23] The six minute walk test has been found to be a valid and reliable test in patients with cancer.[24]

Patient Reported Outcome Measures (PROMs)

As this is a feasibility study, it is useful to determine the feasibility and acceptability of outcomes to be used. For this reason, two different sets of patient reported outcome measures (PROMs) are issued to alternate patients taking part in the study (Table 2). The data collected in the outcome measures and in the qualitative interviews will determine their value in any future studies.

Group	Category	Measure
Physical activity/fitness	Group 1	International Physical Activity Questionnaire[25]
	Group 2	Godin Leisure Time[26]
Mental wellbeing	Group 1 and 2	Warwick and Edinburgh Mental Well-being Scale[27]
Quality of Life	Group 1	FACT-MM[28]
	Group 2	EORTC QLQ C30 MY20[29]
Self-efficacy for exercise	Group 1 and 2	Self-Efficacy for Exercise Scale[30]

For peer review only - http://bmjopen.bmj.com/site/about/guidelines.xhtml

For peer review only

Table 2 - Patient Reported Outcome Measures

Activity Data

The following activity data is collected for each participant:

- the number of gym attendances
- follow-up compliance
- withdrawals from the study and at which stage of the study these occur
- reasons for withdrawal or non-attendance.

Data Collection

Table 1 shows the full data collection schedule for the study.

Data Analysis

Flow of participants through the study is captured and the baseline clinical and demographic characteristics of consented participants assessed with appropriate summary statistics.

The data analysis for the feasibility objectives uses descriptive statistics and focuses on confidence interval estimation.

1. The feasibility of recruitment to main trial is assessed with the consent rate (defined as the ratio of no. of consented participants/no. of eligible participants) and its associated 95% confidence interval and the recruitment rate per month and its associated 95% confidence intervals. The target recruitment rate is a minimum of 2 participants per month.
2. Reporting of the number and characteristics of eligible patients approached for the study and reasons for refused consent
3. Reporting of study participant retention rates at six-week follow-up (e.g. participants with a valid 6-minute walk outcome – the probable primary outcome for the main trial) and its associated 95% confidence interval. The target is a minimum of 80% retention to 6-week follow up assessment.
4. Reporting of the number (and rate) of serious adverse events/incidents (and its associated 95% CI) experienced by the participants in the pre-transplantation period. A serious adverse event (SAE) is defined as any adverse event or adverse reaction that results in death, is life-threatening, requires hospitalisation or prolongation of existing hospitalisation, results in persistent or significant disability or incapacity, or is a congenital anomaly or birth defect.
5. Reporting of the decision on primary endpoint for any main trial (current estimate suggests 80% power, two-sided, with n=610 to detect 5% [18m] difference in 6 min walk test with 10% dropout at 12m).

Qualitative data collection and analysis

Sampling and Data Collection

The aim of the qualitative data collection and analysis is to explore in greater detail patients' perceptions of the study including its acceptability, as well as barriers and facilitators to participation.

Patients who decline to take part in the exercise trial are asked if they would undertake a short telephone interview to ascertain their reasons for not taking part in the study. Participants who have already consented to take part in the trial and are undertaking the exercise programme are approached by a member of the clinical team and asked if they would be interested in taking part in a series of face-to-face or telephone interviews (Figure 1).

The interview topic guide is informed by evidence regarding acceptability and barriers and facilitators to participation from previous studies in pre-habilitation and studies of exercise in patients with multiple myeloma.[17,21] It is also tailored to match developments and areas of interest that emerged from the quantitative data collection as the study progresses. The topic guide is flexible in order to enable exploration of individual experiences, for example, those who had fully completed the intervention compared to those who may have had only limited participation.

For peer review only - http://bmjopen.bmj.com/site/about/guidelines.xhtml

Topic areas include: reasons for non-participation, participants' characteristics and descriptive information regarding the nature of their disease management to date; the patient experience of the intervention, with reference to aspects that may impact the design of future study e.g. recruitment, ease or difficulty of attendance, timing and nature of data collection, suitability of outcome measures; barriers and enablers to participation in the study.

Qualitative Analysis

The Framework Approach is used to analyse the qualitative data.[31] This method is appropriate for identifying, analysing, and reporting themes and patterns within data. It is a flexible and useful research tool, which can potentially provide a rich and detailed, yet simple account of data. Early on in the analysis the transcripts are repeatedly read to develop an understanding of the breadth and depth of the data. During this process, data are labelled and coded in an iterative process whereby patterns and sequences of content over time are identified within and across all the participants. Emergent themes are further developed and refined by analysing similarities and divergences between and within the participants, to form a coherent pattern[32].

Ethics and Dissemination

Ethical Consideration

Ethical approval for this study was obtained from NHS Health Research Authority - Yorkshire and Humber reference 16/YH/0304.

Ethical issues relating to informed consent and confidentiality are addressed throughout. It is acknowledged that patients approached and participating in this study may be physically debilitated and experiencing anxiety, having received a new cancer diagnosis and awaiting a challenging programme of treatment. Due care and diligence are taken when consenting potential subjects and the option to withdraw from the study at any point is reiterated. In particular, the nature of qualitative interviews, focusing on personal experiences of illness and treatment, may result in some distress to some participants. The researchers have relevant experience in working with patients with life-threatening illness and are skilled at talking to them, as well as being able to recognise patient distress.

Dissemination

This study has involvement from, and relevance to, the professions of physiotherapy, medicine and nursing. Dissemination will incorporate each of these professions and reach into the wider healthcare community. We will seek to share the findings of the study through local, national and international channels.

Patient involvement in the project has been through representation in study design and on the project steering group from the North Trent Cancer Research Network Consumer Research Panel. We will liaise with this group to invite ideas regarding dissemination to study participants, patients and carers.

Where the findings of the study have implications for the provision of new or existing services to patients with myeloma, we will ensure dissemination to relevant key opinion leaders and stakeholders to support decision making.

The study is registered in the clinical trials registry at https://clinicaltrials.gov/show/NCT03135925.

Discussion

It is anticipated that this study will demonstrate the feasibility of conducting research into pre-habilitation physical activity programmes. Factors likely to affect feasibility may include: patient perception of role of physical activity; patient time commitments; patient wellness to take part; patient enjoyment of exercise.

For peer review only - http://bmjopen.bmj.com/site/about/guidelines.xhtml

If feasibility is confirmed then we will seek to establish a larger scale study to test the efficacy of the intervention. The findings from this study will be used to support power and sample size calculations and to establish suitable outcome measures for future studies.

If the feasibility criteria are not satisfied then there will be lessons to learn regarding the potential for future studies in the field, or modifications to the intervention or study design if further study is indicated. Since pre-habilitation is an area of growing interest in other clinical areas, including other cancer and non-cancer pathologies, then it is anticipated that the findings of this study will also be of interest to practitioners considering pre-habilitation outside of myeloma.

Establishing the feasibility of research in this field is important to explore the case for pre-habilitation. The effects of bone marrow transplantation can have a high cost to the individual and to health services. There is clearly value in exploring treatment options that may lessen the effects of treatment, particularly those with relatively low associated costs such as exercise pre-habilitation.

Contributors

JD conceived of the idea and secured funding with CK, JAS, KC, DG, SW and SM, who is the Chief Investigator. Ethics and research governance applications were made by SM, CK and HR. JS, HR and LS provided intellectual input and study design for the final protocol of the study.

Data Sharing Statement

As the paper relates to a study protocol, there are no additional data sets available as yet

Funding

The research was funded by the NIHR Research for Patient Benefit (RfPB) Programme PB-PG-0214-33067. The views expressed are those of the author(s) and not necessarily those of the NHS, the NIHR or the Department of Health.

Figure legend

Figure 1 - Recruitment and Intervention Flow Chart

References

1. Hameatological Malignancy Research Network. Myeloma Statistics. Statistics on Individual Disorders. https://www.hmrn.org/statistics/disorders/24. Published 2017.
2. Bird JM, Owen RG, D'Sa S, et al. Haemato-cncology Task Force of the British Committee for Standards in Haematology, UK Myeloma Forum: Guidelines for the Diagnosis and Management of Multiple Myeloma. *Br Jouenal Haematol*. 2011;154(1):32-75.
3. Renshaw C, Ketley N, Møller H, Davies EA. Trends in the incidence and survival of multiple myeloma in South East England 1985-2004. *BMC Cancer*. 2010;10(1):74. doi:10.1186/1471-2407-10-74.
4. Cancer Research UK. Myeloma Statistics. About Cancer. http://www.cancerresearchuk.org/health-professional/cancer-statistics/statistics-by-cancer-type/myeloma. Published 2017.
5. Kumar SK, Rajkumar S V., Dispenzieri A, et al. Improved survival in multiple myeloma and the impact of novel therapies. *Blood*. 2008;111(5):2516-2520. doi:10.1182/blood-2007-10-116129.
6. Snowden JA, Ahmedzai SH, Ashcroft J, et al. Guidelines for supportive care in multiple myeloma 2011. *Br J Haematol*. 2011;154(1):76-103. doi:10.1111/j.1365-2141.2011.08574.x.
7. Bird JM, Owen RG, D'Sa S, et al. Guidelines for the diagnosis and management of multiple myeloma 2011. *Br J Haematol*. 2011;154(1):32-75. doi:10.1111/j.1365-2141.2011.08573.x.
8. Pratt G, Jenner M, Owen R, et al. Updates to the guidelines for the diagnosis and

For peer review only - http://bmjopen.bmj.com/site/about/guidelines.xhtml

management of multiple myeloma. *Br J Haematol*. 2014;167(1):131-133. doi:10.1111/bjh.12926.

9. Cook G, Ashcroft AJ, Cairns DA, et al. The effect of salvage autologous stem-cell transplantation on overall survival in patients with relapsed multiple myeloma (final results from BSBMT/UKMF Myeloma X Relapse [Intensive]): a randomised, open-label, phase 3 trial. *Lancet Haematol*. 2016;3(7):e340-e351. doi:10.1016/S2352-3026(16)30049-7.
10. Cook G, Williams C, Brown JM, et al. High-dose chemotherapy plus autologous stem-cell transplantation as consolidation therapy in patients with relapsed multiple myeloma after previous autologous stem-cell transplantation (NCRI Myeloma X Relapse [Intensive trial]): a randomised, open-label,. *Lancet Oncol*. 2014;15(8):874-885. doi:10.1016/S1470-2045(14)70245-1.
11. Boland E, Eiser C, Ezaydi Y, Greenfield DM, Ahmedzai SH, Snowden JA. Living With Advanced But Stable Multiple Myeloma: A Study of the Symptom Burden and Cumulative Effects of Disease and Intensive (Hematopoietic Stem Cell Transplant-Based) Treatment on Health-Related Quality of Life. *J Pain Symptom Manage*. 2013;46(5):671-680. doi:10.1016/j.jpainsymman.2012.11.003.
12. Snowden JA, Greenfield DM, Bird JM, et al. Guidelines for screening and management of late and long-term consequences of myeloma and its treatment. *Br J Haematol*. 2017;176(6):888-907. doi:10.1111/bjh.14514.
13. Craike MJ, Hose K, Courneya KS, Harrison SJ, Livingston PM. Perceived benefits and barriers to exercise for recently treated patients with multiple myeloma: a qualitative study. *BMC Cancer*. 2013;13(1):319. doi:10.1186/1471-2407-13-319.
14. Hoffman R, Mooney K, Barton D, Rothwell E, Le Stayo P, Wong B. Exercise and stem cell transplantation. 2013.
15. Smith L, McCourt O, Henrich M, et al. Multiple myeloma and physical activity: a scoping review. *BMJ Open*. 2015;5(11):e009576. doi:10.1136/bmjopen-2015-009576.
16. Persoon S, Kersten MJ, ChinAPaw MJ, et al. Design of the EXercise Intervention after Stem cell Transplantation (EXIST) study: a randomized controlled trial to evaluate the effectiveness and cost-effectiveness of an individualized high intensity physical exercise program on fitness and fatigue in p. *BMC Cancer*. 2010;10(1):671. doi:10.1186/1471-2407-10-671.
17. Groeneveldt L, Mein G, Garrod R, et al. A mixed exercise training programme is feasible and safe and may improve quality of life and muscle strength in multiple myeloma survivors. *BMC Cancer*. 2013;13(1):31. doi:10.1186/1471-2407-13-31.
18. Silver JK. A journey to make cancer rehabilitation the standard of care. *Work*. 2013;46:473-475.
19. Silver JK, Baima J. Cancer Prehabilitation. *Am J Phys Med Rehabil*. 2013;92(8):715-727. doi:10.1097/PHM.0b013e31829b4afe.
20. Lee L, Li C, Landry T, et al. A Systematic Review of Economic Evaluations of Enhanced Recovery Pathways for Colorectal Surgery. *Ann Surg*. 2014;259(4):670-676. doi:10.1097/SLA.0b013e318295fef8.
21. Coleman E, Coon S, Hall-Barrow J, Richards K, Gaylor D, Stewart B. Feasibility of exercise during treatment for multiple myeloma. *Cancer Nurs*. 2003;26:410-419.
22. G. Cook; C. Williams; A. Szubert; K. Yong; J. Cavet; H. Hunter; J. Bird; S. Bell; S. O'Connor; J. Cavenagh; J. Snowden; C. Parrish; J. Ashcroft; J. Brown; C. Morris. A second autologous stem cell transplant induces superior response durability following bortezomib-containing reinduction therapy for relapsed multiple myeloma: results from the BSBMT/UKMF myeloma x (intensive) trial: O155. *Bone Marrow Transplant*. 2013;48(S17).

For peer review only - http://bmjopen.bmj.com/site/about/guidelines.xhtml

23. Enright PL. The Six-Minute Walk Test. *Respir Care*. 2003;48(8):783-785.

24. Schmidt, K., Vogt, L., Thiel, C. Jäger, E. Banzwer W. Validity of the six-minute walk test in cancer patients. *Int J Sports Med*. 2013;34(7):631-636.

25. Craig C, Marshall A, Bauman A, et al. International Physical Activity Questionnaire: 12-Country Reliability and Validity. *Med Sci Sport Exerc*. 2003;35(8):1381-1395. doi:10.1249/01.MSS.0000078924.61453.FB.

26. Godin G, Shephard RJ. Godin Leisure-Time Exercise Questionnaire. *Med Sci Sports Exerc*. 1997;29:36-38.

27. Tennant R, Hiller L, Fishwick R, et al. The Warwick-Edinburgh Mental Well-being Scale (WEMWBS): development and UK validation. *Health Qual Life Outcomes*. 2007;5(1):63. doi:10.1186/1477-7525-5-63.

28. Wagner LI, Robinson D, Weiss M, et al. Content Development for the Functional Assessment of Cancer Therapy-Multiple Myeloma (FACT-MM): Use of Qualitative and Quantitative Methods for Scale Construction. *J Pain Symptom Manage*. 2012;43(6):1094-1104. doi:10.1016/j.jpainsymman.2011.06.019.

29. European Organisation for Research and Treatment of Cancer. EORTC Quality of life Questionnaire. Quality of Life. http://groups.eortc.be/qol/eortc-qlq-c30. Published 2017.

30. Kroll T, Kehn M, Ho P-S, Groah S. The SCI Exercise Self-Efficacy Scale (ESES): development and psychometric properties. *Int J Behav Nutr Phys Act*. 2007;4(1):34. doi:10.1186/1479-5868-4-34.

31. Ritchie J, Lewis J. *Qualitative Research Practice*. London: Sage; 2003.

32. Sangster-Gormley E. How case-study research can help to explain implementation of the nurse practitioner role. *Nurse Res*. 2013;20(4):6-11. http://www.ncbi.nlm.nih.gov/pubmed/23520706.

Competing Interests

Professor Walters reports personal fees from Book Royalties, grants from NIHR and MRC, personal fees from External examining.

For peer review only

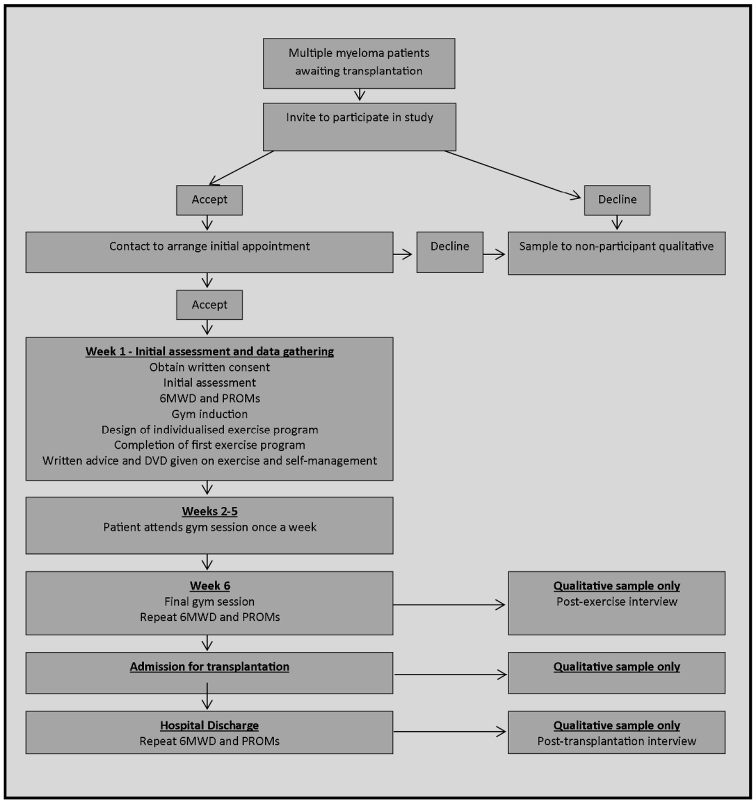

Retention and Intervention Flow Chart

209x297mm (300 x 300 DPI)

SPIRIT
STANDARD PROTOCOL ITEMS: RECOMMENDATIONS FOR INTERVENTIONAL TRIALS

SPIRIT 2013 Checklist: Recommended items to address in a clinical trial protocol and related documents*

Section/item	Item No	Description
**Administrative information**		
Title	1	Descriptive title identifying the study design, population, interventions, and, if applicable, trial acronym
Trial registration	2a	Trial identifier and registry name. If not yet registered, name of intended registry
	2b	All items from the World Health Organization Trial Registration Data Set
Protocol version	3	Date and version identifier
Funding	4	Sources and types of financial, material, and other support
Roles and responsibilities	5a	Names, affiliations, and roles of protocol contributors
	5b	Name and contact information for the trial sponsor
	5c	Role of study sponsor and funders, if any, in study design; collection, management, analysis, and interpretation of data; writing of the report; and the decision to submit the report for publication, including whether they will have ultimate authority over any of these activities
	5d	Composition, roles, and responsibilities of the coordinating centre, steering committee, endpoint adjudication committee, data management team, and other individuals or groups overseeing the trial, if applicable (see Item 21a for data monitoring committee)
**Introduction**		
Background and rationale	6a	Description of research question and justification for undertaking the trial, including summary of relevant studies (published and unpublished) examining benefits and harms for each intervention
	6b	Explanation for choice of comparators
Objectives	7	Specific objectives or hypotheses
Trial design	8	Description of trial design including type of trial (eg, parallel group, crossover, factorial, single group), allocation ratio, and framework (eg, superiority, equivalence, noninferiority, exploratory)

For peer review only

Methods: Participants, interventions, and outcomes

Study setting	9	Description of study settings (eg, community clinic, academic hospital) and list of countries where data will be collected. Reference to where list of study sites can be obtained
Eligibility criteria	10	Inclusion and exclusion criteria for participants. If applicable, eligibility criteria for study centres and individuals who will perform the interventions (eg, surgeons, psychotherapists)
Interventions	11a	Interventions for each group with sufficient detail to allow replication, including how and when they will be administered
	11b	Criteria for discontinuing or modifying allocated interventions for a given trial participant (eg, drug dose change in response to harms, participant request, or improving/worsening disease)
	11c	Strategies to improve adherence to intervention protocols, and any procedures for monitoring adherence (eg, drug tablet return, laboratory tests)
	11d	Relevant concomitant care and interventions that are permitted or prohibited during the trial
Outcomes	12	Primary, secondary, and other outcomes, including the specific measurement variable (eg, systolic blood pressure), analysis metric (eg, change from baseline, final value, time to event), method of aggregation (eg, median, proportion), and time point for each outcome. Explanation of the clinical relevance of chosen efficacy and harm outcomes is strongly recommended
Participant timeline	13	Time schedule of enrolment, interventions (including any run-ins and washouts), assessments, and visits for participants. A schematic diagram is highly recommended (see Figure)
Sample size	14	Estimated number of participants needed to achieve study objectives and how it was determined, including clinical and statistical assumptions supporting any sample size calculations
Recruitment	15	Strategies for achieving adequate participant enrolment to reach target sample size

Methods: Assignment of interventions (for controlled trials)

Allocation:

Sequence generation	16a	Method of generating the allocation sequence (eg, computer-generated random numbers), and list of any factors for stratification. To reduce predictability of a random sequence, details of any planned restriction (eg, blocking) should be provided in a separate document that is unavailable to those who enrol participants or assign interventions

For peer review only - http://bmjopen.bmj.com/site/about/guidelines.xhtml

Allocation concealment mechanism	16b	Mechanism of implementing the allocation sequence (eg, central telephone; sequentially numbered, opaque, sealed envelopes), describing any steps to conceal the sequence until interventions are assigned
Implementation	16c	Who will generate the allocation sequence, who will enrol participants, and who will assign participants to interventions
Blinding (masking)	17a	Who will be blinded after assignment to interventions (eg, trial participants, care providers, outcome assessors, data analysts), and how
	17b	If blinded, circumstances under which unblinding is permissible, and procedure for revealing a participant's allocated intervention during the trial
**Methods: Data collection, management, and analysis**		
Data collection methods	18a	Plans for assessment and collection of outcome, baseline, and other trial data, including any related processes to promote data quality (eg, duplicate measurements, training of assessors) and a description of study instruments (eg, questionnaires, laboratory tests) along with their reliability and validity, if known. Reference to where data collection forms can be found, if not in the protocol
	18b	Plans to promote participant retention and complete follow-up, including list of any outcome data to be collected for participants who discontinue or deviate from intervention protocols
Data management	19	Plans for data entry, coding, security, and storage, including any related processes to promote data quality (eg, double data entry; range checks for data values). Reference to where details of data management procedures can be found, if not in the protocol
Statistical methods	20a	Statistical methods for analysing primary and secondary outcomes. Reference to where other details of the statistical analysis plan can be found, if not in the protocol
	20b	Methods for any additional analyses (eg, subgroup and adjusted analyses)
	20c	Definition of analysis population relating to protocol non-adherence (eg, as randomised analysis), and any statistical methods to handle missing data (eg, multiple imputation)
**Methods: Monitoring**		
Data monitoring	21a	Composition of data monitoring committee (DMC); summary of its role and reporting structure; statement of whether it is independent from the sponsor and competing interests; and reference to where further details about its charter can be found, if not in the protocol. Alternatively, an explanation of why a DMC is not needed

	21b	Description of any interim analyses and stopping guidelines, including who will have access to these interim results and make the final decision to terminate the trial
Harms	22	Plans for collecting, assessing, reporting, and managing solicited and spontaneously reported adverse events and other unintended effects of trial interventions or trial conduct
Auditing	23	Frequency and procedures for auditing trial conduct, if any, and whether the process will be independent from investigators and the sponsor
**Ethics and dissemination**		
Research ethics approval	24	Plans for seeking research ethics committee/institutional review board (REC/IRB) approval
Protocol amendments	25	Plans for communicating important protocol modifications (eg, changes to eligibility criteria, outcomes, analyses) to relevant parties (eg, investigators, REC/IRBs, trial participants, trial registries, journals, regulators)
Consent or assent	26a	Who will obtain informed consent or assent from potential trial participants or authorised surrogates, and how (see Item 32)
	26b	Additional consent provisions for collection and use of participant data and biological specimens in ancillary studies, if applicable
Confidentiality	27	How personal information about potential and enrolled participants will be collected, shared, and maintained in order to protect confidentiality before, during, and after the trial
Declaration of interests	28	Financial and other competing interests for principal investigators for the overall trial and each study site
Access to data	29	Statement of who will have access to the final trial dataset, and disclosure of contractual agreements that limit such access for investigators
Ancillary and post-trial care	30	Provisions, if any, for ancillary and post-trial care, and for compensation to those who suffer harm from trial participation
Dissemination policy	31a	Plans for investigators and sponsor to communicate trial results to participants, healthcare professionals, the public, and other relevant groups (eg, via publication, reporting in results databases, or other data sharing arrangements), including any publication restrictions
	31b	Authorship eligibility guidelines and any intended use of professional writers
	31c	Plans, if any, for granting public access to the full protocol, participant-level dataset, and statistical code

For peer review only

Appendices

Informed consent materials	32	Model consent form and other related documentation given to participants and authorised surrogates
Biological specimens	33	Plans for collection, laboratory evaluation, and storage of biological specimens for genetic or molecular analysis in the current trial and for future use in ancillary studies, if applicable

*It is strongly recommended that this checklist be read in conjunction with the SPIRIT 2013 Explanation & Elaboration for important clarification on the items. Amendments to the protocol should be tracked and dated. The SPIRIT checklist is copyrighted by the SPIRIT Group under the Creative Commons "Attribution-NonCommercial-NoDerivs 3.0 Unported" license.

For peer review only

BMJ Open

Is it feasible to conduct a randomised controlled trial of pre-transplant exercise (pre-habilitation) for multiple myeloma patients awaiting autologous haematopoietic stem cell transplantation? Protocol for the PREeMPT study.

Journal:	*BMJ Open*
Manuscript ID	bmjopen-2017-021333.R1
Article Type:	Protocol
Date Submitted by the Author:	24-Jan-2018

Complete List of Authors:	Keen, Carol; Sheffield Teaching Hospitals NHS Foundation Trust, Acute Therapy Services<br>Skilbeck, Julie; Sheffield Hallam University, Nursing<br>Ross, Helen; Sheffield Teaching Hospitals NHS Foundation Trust, Acute Therapy Services<br>Smith, Lauren; Sheffield Teaching Hospitals NHS Foundation Trust, Acute Therapy Services<br>Collins, Karen; Sheffield Hallam University, Centre for Health and Social Care Research<br>Dixey, Joanne; Sheffield Teaching Hospitals NHS Foundation Trust<br>Walters, Stephen; University of Sheffield, ScHARR<br>Greenfield, Diana; Sheffield Teaching Hospitals NHS Foundation Trust<br>Snowden, John; Sheffield Teaching Hospitals NHS Foundation Trust, Department of Haematology<br>Mawson, Susan; University of Sheffield, School of Health and Related Research

**Primary Subject Heading**:	Haematology (incl blood transfusion)
Secondary Subject Heading:	Rehabilitation medicine
Keywords:	Myeloma < HAEMATOLOGY, Bone marrow transplantation < HAEMATOLOGY, Rehabilitation medicine < INTERNAL MEDICINE

SCHOLARONE™ Manuscripts

Title: Is it feasible to conduct a randomised controlled trial of pre-transplant exercise (pre-habilitation) for multiple myeloma patients awaiting autologous haematopoietic stem cell transplantation? Protocol for the PREeMPT study.

FUNDING: This is an original research study funded by NIHR Research for Patient Benefit (RfPB) funding

AUTHORS: Carol Keen[1], Julie Skilbeck(JSk)[2], Helen Ross[1], Lauren Smith[1], Karen Collins[2], Joanne Dixey[1], Stephen Walters[3], Diana M Greenfield[1,3], John A Snowden(JSn)[1,3], Susan Mawson[1,3]

(1) Sheffield Teaching Hospitals NHS Foundation Trust
(2) Sheffield Hallam University
(3) University of Sheffield

CORRESPONDING AUTHOR:
Carol Keen - carol.keen@sth.nhs.uk

For peer review only

For peer review only - http://bmjopen.bmj.com/site/about/guidelines.xhtml

Abstract

Introduction:

While myeloma is an incurable malignancy, developments in disease management have led to increased life expectancy in recent years. Treatment typically involves stem-cell transplantation. Increased survival rates equates to more patients living with the burden of both the disease and its treatment for increasing numbers of years, rendering myeloma a long term condition.

Evidence exists to demonstrate the benefits of exercise for patients recovering from stem cell transplantation, and pre-habilitation – exercise before treatment - has been shown to be effective in other disease areas. To date there has been no research into pre-habitation in myeloma patients awaiting transplantation treatment.

Our objective is to determine whether it is feasible to conduct a randomised controlled trial into pre-transplant exercise for patients with multiple myeloma who are awaiting autologous stem cell transplantation.

Methods and analysis:

This mixed methods study identifies patients with diagnosis of multiple myeloma who have been assigned to the autologous transplantation list and invites them to participate in 6 weekly sessions of individualised, supervised exercise whilst awaiting transplantation.

Quantitative data to determine feasibility targets include rates of recruitment, adherence and adverse events, and outcome measures including six minute walking distance (6MWD) test and quality of life.

Qualitative interviews are undertaken with a purposive sample of patient to capture their experiences of the study and the intervention.

Ethics and dissemination:

Ethics committee approval has been obtained. Dissemination will be through open-access publication and presentation and will seek to reach multi-professional bases as well as patient and carer groups, addressing the widespread interest in this area of research.

The study is registered in the clinical trials registry at https://clinicaltrials.gov/show/NCT03135925.

For peer review only

Strengths and limitations

- The sample size for the qualitative aspect of this study is likely to be small – it is intended to inform future study design rather than provide
- For practical reasons, and to encourage patient recruitment, time points for data collection are aligned with clinical interventions, rather than specifically for research purposes. They are therefore subject to variation, and not within the control of the study team.
- As a feasibility study, this will not provide evidence of the effectiveness of pre-habilitation, but will inform future study design for evaluating effectiveness

Introduction

Myeloma is an incurable malignancy of antibody producing B lymphocytes and plasma cells. Equating to 7 new cases per 100,000 population in the UK, it represents 10% of all new haematological cancers.[1] Disease symptoms include anaemia and hypercalcaemia causing fatigue and weakness, immunosuppression and lytic lesions of bone increasing pathological fracture risk.[2]

Due to developments in disease management, life expectancy has increased significantly in the last 10 years.[3] The 5 year relative survival rate for England was 42.2% in 2011,[4] and is set to increase further due to earlier interventions in the disease process, more effective chemotherapies and increased use of autologous stem cell transplantation.[5]

Following diagnosis of multiple myeloma, the standard of care treatment for younger patients (generally, but not exclusively, under the age of 70) with adequate fitness consists of an intensive pathway commencing with induction treatment using a variety of regimens delivered as an

For peer review only - http://bmjopen.bmj.com/site/about/guidelines.xhtml

outpatient or day case given to control disease until maximum response is achieved (usually reflected by a plateau in serum paraprotein).[6–8] This response is then consolidated with autologous stem cell transplantation which permits the administration of high dose myeloablative melphalan chemotherapy, a procedure typically requiring around 3 weeks inpatient care, after which patients take several months to make a functional recovery.[6–8] The procedure is non-curative and relapse/progression of myeloma occurs after an average of 2-3 years, which requires re-institution of induction treatment, and, in many patients, consolidation with a second autologous transplant procedure.[9,10]

Rationale for the study

Increased survival rates equates to more patients living with the burden of both the disease and its treatment for increasing numbers of years, rendering myeloma a long term condition.[11] The cumulative effects of the disease, compounded with the debilitating toxic nature of the treatment, impact significantly on quality of life for patients beyond the end of treatment, with late-effects symptoms including infection, fatigue, metabolic, neurological and cardiovascular disorders, as well as pain, physical fitness and psychological concerns.[12]

Only 20% of myeloma patients meet national physical activity guidelines post-treatment[12] and activity declines through treatment due to perceived barriers to exercise including pain, fear of injury and fatigue.[13] Although research evidence in physical activity has been demonstrated to be limited,[14] evidence exists to demonstrate the benefits of exercise for patients recovering from stem cell transplantation.[15] Pre-habilitation after treatment in myeloma patients has been shown to improve symptoms of physical performance, muscle strength, aerobic capacity, psychological outcomes immunological function and fatigue.[16] Exercise training for myeloma survivors has been shown to be safe and feasible during treatment with high attendance and adherence[17] and has been implement widely in clinical practice.

Studies demonstrate that pre-transplant patients have reduced exercise capacity and increased co-morbidities compared with a normal population, yet most rehabilitative interventions occur during and after treatment.[15] Thus while exercise rehabilitation after treatment for myeloma can be effective, we must also consider rehabilitative interventions prior to the start of treatment: pre-habilitation, defined as,

> *"a process on the continuum of care that occurs between the time of cancer diagnosis and the beginning of acute treatment … provides targeted interventions that improve a patient's health to reduce the incidence and the severity of current and future impairments".*[18]

Examples of pre-habilitation exist in other clinical specialties: it has been used for some time in orthopaedic surgery to improve outcomes and postoperative recovery,[19] and its economic benefits have been demonstrated within colorectal surgery.[20] A review of pre-habilitation in pre-surgical cancer patients demonstrated the effective use of aerobic interventions in the management of patients undergoing thoracic surgery for lung cancer, identified the potential for its use in other oncology settings and called for further research to evaluate pre-habilitation for wider groups of cancer patients.[19]

Guidelines for the management of late and long terms effects of myeloma recommend that regular physical activity, including pre-habilitation and rehabilitation, and aspiration to a general healthy lifestyles, are integral to patient care pathways.[12]

Autologous stem cell transplantation in myeloma has become the commonest indication for transplantation, with, for example, over 1400 performed in the UK annually, and procedures are performed in what is normally considered an elderly patient population, many with comorbidities and frailty. It is an intensive toxic procedure, with a recovery period of at least 6 months and strategies to improve recovery are warranted, including pre-habilitation. A window of opportunity – usually a period of 4-6 months exists to offer pre-habilitation between diagnosis or relapse and the commencement of the autologous stem cell transplantation process. Coleman et al.[21] studied 24

For peer review only - http://bmjopen.bmj.com/site/about/guidelines.xhtml

multiple myeloma patients undergoing a home based exercise program during chemotherapy and stem cell transplantation and identified that no patient injured themselves and that the intervention had positive effects on lean body weight, fatigue and sleep disturbance. Despite this, no evidence currently exists regarding the use of pre-habilitation exercise interventions in multiple myeloma.

This article describes the protocol for a study underway investigating the feasibility of research into the provision of an exercise intervention in patients with myeloma who are due to receive autologous stem cell transplantation.

Aims and Objectives

The aim of this study is to determine whether it is feasible to conduct a randomised controlled trial into pre-transplant exercise for patients with multiple myeloma who are awaiting autologous stem cell transplantation.

We will determine this through completion of the following objectives:

1. Assess the acceptability of the study to patients by measuring recruitment and retention to the study and through qualitative interview responses
2. Explore reasons for non-consent to study participation
3. Establish whether a target cohort of patients exists.
4. Determine the most appropriate recruitment points post diagnosis through steering group feedback, recruitment rate when compared with numbers invited to join the study and qualitative interview reports
5. Assess the suitability of inclusion and exclusion criteria by examining recruitment data
6. Assess the acceptability of the intervention through qualitative interviews and retention rates during the study
7. Determine duration of the intervention before transplantation commences by monitoring point of recruitment to the study and time to transplant
8. Explore the appropriateness of outcome measures/completeness by qualitative interview responses, completion rates, time to complete.

Methods and Analysis

Methodology

Mixed methods, combining qualitative and quantitative data collection and analysis, are used to achieve the described aims and objectives.

Design

This is a prospective feasibility study – see Figure 1 for study flow chart.

Setting

Assessments and exercise sessions take place in the physiotherapy outpatient department in an acute hospital trust, which is a regional specialist centre for haematological services. Patient interviews take place in private rooms in the physiotherapy department or over the telephone for patient convenience.

Feasibility

The feasibility of the intervention is determined through the following targets:

- Recruitment: based on patient numbers at the study site, the recruitment target is 24 patients in a 12 month period (i.e. 2 patients per month);
- Attendance: minimum average attendance at exercise sessions of 66% of the scheduled/invited sessions;
- Retention: 80% patient retention to 6-week follow up assessment;
- Adverse events: adverse events are closely monitored and use to inform decisions to proceed.

For peer review only - http://bmjopen.bmj.com/site/about/guidelines.xhtml

Acceptability of the intervention to patients is also determined through the qualitative data collection and analysis, described in a later section.

Quantitative Data Collection and Analysis

Data collection will take place between September 2016 and February 2018.

Sampling

Consecutive sampling is used to recruit patients to this study who have a diagnosis of multiple myeloma and have been assigned to the autologous transplantation list. The recruiting centre transplants approximately 70 myeloma patients per year: sampling all patients over a 12 month period will indicate study recruitment feasibility. This feasibility study did not have a formal sample size calculation to determine a priori the number of participants to recruit; it aimed to recruit for a fixed period of time (12 months) at a single centre and one of the outcomes was to estimate the recruitment rate per month.

Inclusion criteria

All patients with a diagnosis of multiple myeloma, assigned to the autologous transplantation waiting list for either a first or second transplant.[22]

Exclusion Criteria

To allow safe completion of initial objective assessments, patients with a history of unstable angina or heart attack in the previous month are excluded.[23] Medical stability is a pre-requisite for transplantation, therefore no patients are excluded on this basis.

Recruitment

Patients are screened at clinic appointments by the bone marrow transplant team during their preparation for transplant. Patients meeting the inclusion criteria are provided with verbal and written information and invited to be involved in the study. Follow-up takes place after 48 hours via a phone call from a study physiotherapist: any remaining questions are discussed and if the patient agrees to take part then written consent is obtained and an initial assessment appointment is made.

Patients who choose not to join the study are invited to take part in a qualitative interview to explore their reasoning (Figure 1). This is described in more detail under Qualitative Data Collection and Analysis.

Intervention:

Initial Assessment

Patients attend an initial assessment with a study physiotherapist who undertakes the following:

- explanation of the pre-habilitation programme
- documentation of written consent
- subjective history including co-morbidities and patient goals
- induction to the gym area equipment
- provision of booklet and DVD with physical activity advice
- baseline objective assessment (Table 1)
- design of individualised gym program in line with patient abilities and goals
- completion of an initial gym circuit with close supervision.

Weeks 2-5

Patients attend weekly 1 hour physiotherapist-led group gym sessions and complete their individualised program. Supervision is available as required and programs are progressed in line with patient ability and performance.

Week 6

Completion of final gym circuit and repeat of objective assessments (Table 1).

Follow up

Patients are followed up on admission for transplant, and again on transplant discharge, for further repeat of objective assessments (Table 1).

For peer review only - http://bmjopen.bmj.com/site/about/guidelines.xhtml

	Recruitment	Initial Assessment	Weeks 2-5	Week 6	Transplant Admission	Transplant Discharge
Screening data	✓					
Demographic data		✓				
6 minute walk distance		✓		✓	✓	✓
PROMs		✓		✓	✓	✓
Activity data		✓	✓	✓	✓	✓
Adverse Events		✓	✓	✓		

Table 1 - Study Data Collection

Outcome measures

The following data are captured for study participants.

Screening Data

Through initial screening and recruitment, data is collected on:

- number of patients meeting inclusion criteria
- patients accepting initial study information
- patients agreeing to attend for initial assessment
- reasons for non-participation.

Demographic data

The following demographic data is captured during the initial assessment:

- gender
- length of diagnosis
- baseline physical activity levels
- transplant history
- pre-transplant therapies received
- time to transplantation from decision to transplant
- other relevant information.

Functional measure

Patients undertake a 6 minute walk test (6MWD) before and after the exercise intervention. The six minute walk test is a useful field test of functional capacity, is safe to administer and although it has less correlation with peak oxygen capacity than the shuttle walk test, it is better tolerated by patients and is more reflective of activities of daily living as it is a submaximal exercise test.[23] The six minute walk test has been found to be a valid and reliable test in patients with cancer.[24]

Patient Reported Outcome Measures (PROMs)

As this is a feasibility study, it is useful to determine the feasibility and acceptability of outcomes to be used. For this reason, two different sets of patient reported outcome measures (PROMs) are issued to alternate patients taking part in the study (Table 2). The data collected in the outcome measures and in the qualitative interviews will determine their value in any future studies.

Group	Category	Measure
Physical activity/fitness	Group 1	International Physical Activity Questionnaire[25]
	Group 2	Godin Leisure Time[26]
Mental wellbeing	Group 1 and 2	Warwick and Edinburgh Mental Well-being Scale[27]
Quality of Life	Group 1	FACT-MM[28]

For peer review only - http://bmjopen.bmj.com/site/about/guidelines.xhtml

	Group 2	EORTC QLQ C30 MY20[29]
Self-efficacy for exercise	Group 1 and 2	Self-Efficacy for Exercise Scale[30]

Table 2 - Patient Reported Outcome Measures

Activity Data

The following activity data is collected for each participant:

- the number of gym attendances
- follow-up compliance
- withdrawals from the study and at which stage of the study these occur
- reasons for withdrawal or non-attendance.

Data Collection

Table 1 shows the full data collection schedule for the study.

Data Analysis

Flow of participants through the study is captured and the baseline clinical and demographic characteristics of consented participants assessed with appropriate summary statistics.

The data analysis for the feasibility objectives uses descriptive statistics and focuses on confidence interval estimation.

1. The feasibility of recruitment to main trial is assessed with the consent rate (defined as the ratio of no. of consented participants/no. of eligible participants) and its associated 95% confidence interval and the recruitment rate per month and its associated 95% confidence intervals. The target recruitment rate is a minimum of 2 participants per month.
2. Reporting of the number and characteristics of eligible patients approached for the study and reasons for refused consent
3. Reporting of study participant retention rates at six-week follow-up (e.g. participants with a valid 6-minute walk outcome – the probable primary outcome for the main trial) and its associated 95% confidence interval. The target is a minimum of 80% retention to 6-week follow up assessment.
4. Reporting of the number (and rate) of serious adverse events/incidents (and its associated 95% CI) experienced by the participants in the pre-transplantation period. A serious adverse event (SAE) is defined as any adverse event or adverse reaction that results in death, is life-threatening, requires hospitalisation or prolongation of existing hospitalisation, results in persistent or significant disability or incapacity, or is a congenital anomaly or birth defect.
5. Reporting of the decision on primary endpoint for any main trial (current estimate suggests 80% power, two-sided, with n=610 to detect 5% [18m] difference in 6 min walk test with 10% dropout at 12m).

Qualitative data collection and analysis

Sampling and Data Collection

The aim of the qualitative data collection and analysis is to explore in greater detail patients' perceptions of the study including its acceptability, as well as barriers and facilitators to participation.

Patients who decline to take part in the exercise trial are asked if they would undertake a short telephone interview to ascertain their reasons for not taking part in the study. Participants who have already consented to take part in the trial and are undertaking the exercise programme are approached by a member of the clinical team and asked if they would be interested in taking part in a series of face-to-face or telephone interviews (Figure 1).

The interview topic guide is informed by evidence regarding acceptability and barriers and facilitators to participation from previous studies in pre-habilitation and studies of exercise in patients with multiple myeloma.[17,21] It is also tailored to match developments and areas of interest

For peer review only - http://bmjopen.bmj.com/site/about/guidelines.xhtml

For peer review only

that emerged from the quantitative data collection as the study progresses. The topic guide is flexible in order to enable exploration of individual experiences, for example, those who had fully completed the intervention compared to those who may have had only limited participation.

Topic areas include: reasons for non-participation, participants' characteristics and descriptive information regarding the nature of their disease management to date; the patient experience of the intervention, with reference to aspects that may impact the design of future study e.g. recruitment, ease or difficulty of attendance, timing and nature of data collection, suitability of outcome measures; barriers and enablers to participation in the study.

Qualitative Analysis

The Framework Approach is used to analyse the qualitative data.[31] This method is appropriate for identifying, analysing, and reporting themes and patterns within data. It is a flexible and useful research tool, which can potentially provide a rich and detailed, yet simple account of data. Early on in the analysis the transcripts are repeatedly read to develop an understanding of the breadth and depth of the data. During this process, data are labelled and coded in an iterative process whereby patterns and sequences of content over time are identified within and across all the participants. Emergent themes are further developed and refined by analysing similarities and divergences between and within the participants, to form a coherent pattern[32].

Ethics and Dissemination

Ethical Consideration

Ethical approval for this study was obtained from NHS Health Research Authority - Yorkshire and Humber reference 16/YH/0304.

Ethical issues relating to informed consent and confidentiality are addressed throughout. It is acknowledged that patients approached and participating in this study may be physically debilitated and experiencing anxiety, having received a new cancer diagnosis and awaiting a challenging programme of treatment. Due care and diligence are taken when consenting potential subjects and the option to withdraw from the study at any point is reiterated. In particular, the nature of qualitative interviews, focusing on personal experiences of illness and treatment, may result in some distress to some participants. The researchers have relevant experience in working with patients with life-threatening illness and are skilled at talking to them, as well as being able to recognise patient distress.

Dissemination

This study has involvement from, and relevance to, the professions of physiotherapy, medicine and nursing. Dissemination will incorporate each of these professions and reach into the wider healthcare community. We will seek to share the findings of the study through local, national and international channels.

Patient involvement in the project has been through representation in study design and on the project steering group from the North Trent Cancer Research Network Consumer Research Panel. We will liaise with this group to invite ideas regarding dissemination to study participants, patients and carers.

Where the findings of the study have implications for the provision of new or existing services to patients with myeloma, we will ensure dissemination to relevant key opinion leaders and stakeholders to support decision making.

The study is registered in the clinical trials registry at https://clinicaltrials.gov/show/NCT03135925.

Discussion

It is anticipated that this study will demonstrate the feasibility of conducting research into pre-habilitation physical activity programmes. Factors likely to affect feasibility may include: patient

For peer review only - http://bmjopen.bmj.com/site/about/guidelines.xhtml

perception of role of physical activity; patient time commitments; patient wellness to take part; patient enjoyment of exercise.

If feasibility is confirmed then we will seek to establish a larger scale study to test the efficacy of the intervention. The findings from this study will be used to support power and sample size calculations and to establish suitable outcome measures for future studies.

If the feasibility criteria are not satisfied then there will be lessons to learn regarding the potential for future studies in the field, or modifications to the intervention or study design if further study is indicated. Since pre-habilitation is an area of growing interest in other clinical areas, including other cancer and non-cancer pathologies, then it is anticipated that the findings of this study will also be of interest to practitioners considering pre-habilitation outside of myeloma.

Establishing the feasibility of research in this field is important to explore the case for pre-habilitation. The effects of bone marrow transplantation can have a high cost to the individual and to health services. There is clearly value in exploring treatment options that may lessen the effects of treatment, particularly those with relatively low associated costs such as exercise pre-habilitation.

Contributors

JD conceived of the idea and secured funding with CK, JSn, KC, DG, SW and SM, who is the Chief Investigator. Ethics and research governance applications were made by SM, CK and HR. JSk, HR and LS provided intellectual input and study design for the final protocol of the study. All authors were involved in drafting or critically revising this work, and in final approval of the version to be published.

Data Sharing Statement

As the paper relates to a study protocol, there are no additional data sets available as yet.

Funding

The research was funded by the NIHR Research for Patient Benefit (RfPB) Programme PB-PG-0214-33067. The views expressed are those of the author(s) and not necessarily those of the NHS, the NIHR or the Department of Health.

Figure legend

Figure 1 - Recruitment and Intervention Flow Chart

References

1. Hameatological Malignancy Research Network. Myeloma Statistics. Statistics on Individual Disorders. https://www.hmrn.org/statistics/disorders/24. Published 2017.

2. Bird JM, Owen RG, D'Sa S, et al. Haemato-cncology Task Force of the British Committee for Standards in Haematology, UK Myeloma Forum: Guidelines for the Diagnosis and Management of Multiple Myeloma. *Br Jouenal Haematol*. 2011;154(1):32-75.

3. Renshaw C, Ketley N, Møller H, Davies EA. Trends in the incidence and survival of multiple myeloma in South East England 1985-2004. *BMC Cancer*. 2010;10(1):74. doi:10.1186/1471-2407-10-74.

4. Cancer Research UK. Myeloma Statistics. About Cancer. http://www.cancerresearchuk.org/health-professional/cancer-statistics/statistics-by-cancer-type/myeloma. Published 2017.

5. Kumar SK, Rajkumar S V., Dispenzieri A, et al. Improved survival in multiple myeloma and the impact of novel therapies. *Blood*. 2008;111(5):2516-2520. doi:10.1182/blood-2007-10-116129.

6. Snowden JA, Ahmedzai SH, Ashcroft J, et al. Guidelines for supportive care in multiple

For peer review only - http://bmjopen.bmj.com/site/about/guidelines.xhtml

myeloma 2011. *Br J Haematol*. 2011;154(1):76-103. doi:10.1111/j.1365-2141.2011.08574.x.

7. Bird JM, Owen RG, D'Sa S, et al. Guidelines for the diagnosis and management of multiple myeloma 2011. *Br J Haematol*. 2011;154(1):32-75. doi:10.1111/j.1365-2141.2011.08573.x.

8. Pratt G, Jenner M, Owen R, et al. Updates to the guidelines for the diagnosis and management of multiple myeloma. *Br J Haematol*. 2014;167(1):131-133. doi:10.1111/bjh.12926.

9. Cook G, Ashcroft AJ, Cairns DA, et al. The effect of salvage autologous stem-cell transplantation on overall survival in patients with relapsed multiple myeloma (final results from BSBMT/UKMF Myeloma X Relapse [Intensive]): a randomised, open-label, phase 3 trial. *Lancet Haematol*. 2016;3(7):e340-e351. doi:10.1016/S2352-3026(16)30049-7.

10. Cook G, Williams C, Brown JM, et al. High-dose chemotherapy plus autologous stem-cell transplantation as consolidation therapy in patients with relapsed multiple myeloma after previous autologous stem-cell transplantation (NCRI Myeloma X Relapse [Intensive trial]): a randomised, open-label,. *Lancet Oncol*. 2014;15(8):874-885. doi:10.1016/S1470-2045(14)70245-1.

11. Boland E, Eiser C, Ezaydi Y, Greenfield DM, Ahmedzai SH, Snowden JA. Living With Advanced But Stable Multiple Myeloma: A Study of the Symptom Burden and Cumulative Effects of Disease and Intensive (Hematopoietic Stem Cell Transplant-Based) Treatment on Health-Related Quality of Life. *J Pain Symptom Manage*. 2013;46(5):671-680. doi:10.1016/j.jpainsymman.2012.11.003.

12. Snowden JA, Greenfield DM, Bird JM, et al. Guidelines for screening and management of late and long-term consequences of myeloma and its treatment. *Br J Haematol*. 2017;176(6):888-907. doi:10.1111/bjh.14514.

13. Craike MJ, Hose K, Courneya KS, Harrison SJ, Livingston PM. Perceived benefits and barriers to exercise for recently treated patients with multiple myeloma: a qualitative study. *BMC Cancer*. 2013;13(1):319. doi:10.1186/1471-2407-13-319.

14. Smith L, McCourt O, Henrich M, et al. Multiple myeloma and physical activity: a scoping review. *BMJ Open*. 2015;5(11):e009576. doi:10.1136/bmjopen-2015-009576.

15. Hoffman R, Mooney K, Barton D, Rothwell E, Le Stayo P, Wong B. Exercise and stem cell transplantation. 2013.

16. Persoon S, Kersten MJ, ChinAPaw MJ, et al. Design of the EXercise Intervention after Stem cell Transplantation (EXIST) study: a randomized controlled trial to evaluate the effectiveness and cost-effectiveness of an individualized high intensity physical exercise program on fitness and fatigue in p. *BMC Cancer*. 2010;10(1):671. doi:10.1186/1471-2407-10-671.

17. Groeneveldt L, Mein G, Garrod R, et al. A mixed exercise training programme is feasible and safe and may improve quality of life and muscle strength in multiple myeloma survivors. *BMC Cancer*. 2013;13(1):31. doi:10.1186/1471-2407-13-31.

18. Silver JK. A journey to make cancer rehabilitation the standard of care. *Work*. 2013;46:473-475.

19. Silver JK, Baima J. Cancer Prehabilitation. *Am J Phys Med Rehabil*. 2013;92(8):715-727. doi:10.1097/PHM.0b013e31829b4afe.

20. Lee L, Li C, Landry T, et al. A Systematic Review of Economic Evaluations of Enhanced Recovery Pathways for Colorectal Surgery. *Ann Surg*. 2014;259(4):670-676. doi:10.1097/SLA.0b013e318295fef8.

21. Coleman E, Coon S, Hall-Barrow J, Richards K, Gaylor D, Stewart B. Feasibility of exercise during treatment for multiple myeloma. *Cancer Nurs*. 2003;26:410-419.

22. G. Cook; C. Williams; A. Szubert; K. Yong; J. Cavet; H. Hunter; J. Bird; S. Bell; S. O'Connor; J.

For peer review only - http://bmjopen.bmj.com/site/about/guidelines.xhtml

Cavenagh; J. Snowden; C. Parrish; J. Ashcroft; J. Brown; C. Morris. A second autologous stem cell transplant induces superior response durability following bortezomib-containing reinduction therapy for relapsed multiple myeloma: results from the BSBMT/UKMF myeloma x (intensive) trial: O155. *Bone Marrow Transplant*. 2013;48(S17).

23. Enright PL. The Six-Minute Walk Test. *Respir Care*. 2003;48(8):783-785.
24. Schmidt, K., Vogt, L., Thiel, C. Jäger, E. Banzwer W. Validity of the six-minute walk test in cancer patients. *Int J Sports Med*. 2013;34(7):631-636.
25. Craig C, Marshall A, Bauman A, et al. International Physical Activity Questionnaire: 12-Country Reliability and Validity. *Med Sci Sport Exerc*. 2003;35(8):1381-1395. doi:10.1249/01.MSS.0000078924.61453.FB.
26. Godin G, Shephard RJ. Godin Leisure-Time Exercise Questionnaire. *Med Sci Sports Exerc*. 1997;29:36-38.
27. Tennant R, Hiller L, Fishwick R, et al. The Warwick-Edinburgh Mental Well-being Scale (WEMWBS): development and UK validation. *Health Qual Life Outcomes*. 2007;5(1):63. doi:10.1186/1477-7525-5-63.
28. Wagner LI, Robinson D, Weiss M, et al. Content Development for the Functional Assessment of Cancer Therapy-Multiple Myeloma (FACT-MM): Use of Qualitative and Quantitative Methods for Scale Construction. *J Pain Symptom Manage*. 2012;43(6):1094-1104. doi:10.1016/j.jpainsymman.2011.06.019.
29. European Organisation for Research and Treatment of Cancer. EORTC Quality of life Questionnaire. Quality of Life. http://groups.eortc.be/qol/eortc-qlq-c30. Published 2017.
30. Kroll T, Kehn M, Ho P-S, Groah S. The SCI Exercise Self-Efficacy Scale (ESES): development and psychometric properties. *Int J Behav Nutr Phys Act*. 2007;4(1):34. doi:10.1186/1479-5868-4-34.
31. Ritchie J, Lewis J. *Qualitative Research Practice*. London: Sage; 2003.
32. Sangster-Gormley E. How case-study research can help to explain implementation of the nurse practitioner role. *Nurse Res*. 2013;20(4):6-11. http://www.ncbi.nlm.nih.gov/pubmed/23520706.

Competing Interests

Professor Walters reports personal fees from Book Royalties, grants from NIHR and MRC, personal fees from external examining.

For peer review only

For peer review only - http://bmjopen.bmj.com/site/about/guidelines.xhtml

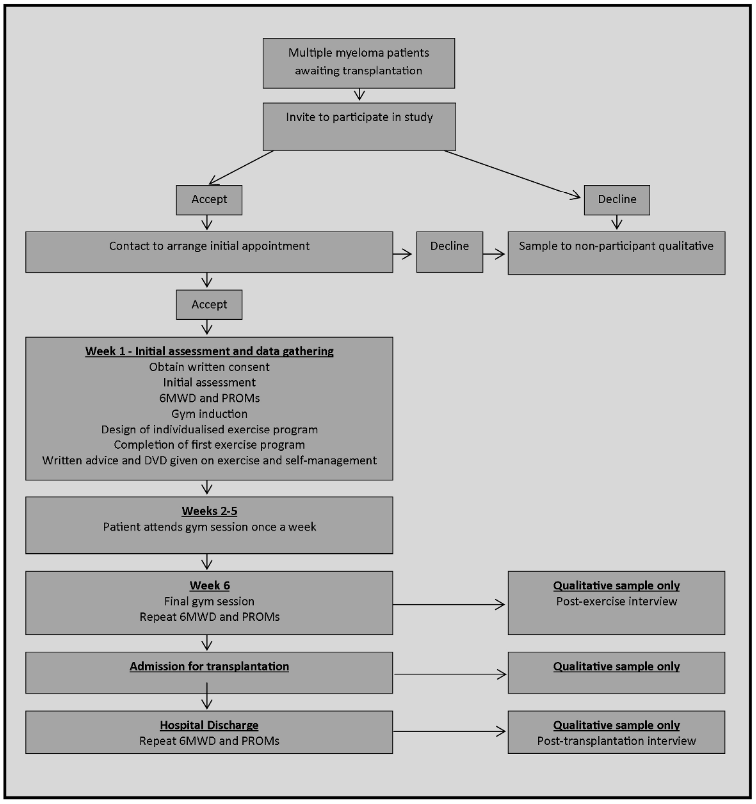

Retention and Intervention Flow Chart

209x297mm (300 x 300 DPI)

SPIRIT
STANDARD PROTOCOL ITEMS: RECOMMENDATIONS FOR INTERVENTIONAL TRIALS

SPIRIT 2013 Checklist: Recommended items to address in a clinical trial protocol and related documents*

Section/item	Item No	Description	Page No
**Administrative information**			
Title	1	Descriptive title identifying the study design, population, interventions, and, if applicable, trial acronym	1
Trial registration	2a	Trial identifier and registry name. If not yet registered, name of intended registry	2
	2b	All items from the World Health Organization Trial Registration Data Set	n/a
Protocol version	3	Date and version identifier	n/a
Funding	4	Sources and types of financial, material, and other support	9
Roles and responsibilities	5a	Names, affiliations, and roles of protocol contributors	9
	5b	Name and contact information for the trial sponsor	n/a
	5c	Role of study sponsor and funders, if any, in study design; collection, management, analysis, and interpretation of data; writing of the report; and the decision to submit the report for publication, including whether they will have ultimate authority over any of these activities	n/a
	5d	Composition, roles, and responsibilities of the coordinating centre, steering committee, endpoint adjudication committee, data management team, and other individuals or groups overseeing the trial, if applicable (see Item 21a for data monitoring committee)	n/a
**Introduction**			
Background and rationale	6a	Description of research question and justification for undertaking the trial, including summary of relevant studies (published and unpublished) examining benefits and harms for each intervention	2
	6b	Explanation for choice of comparators	n/a
Objectives	7	Specific objectives or hypotheses	4

For peer review only

Trial design	8	Description of trial design including type of trial (eg, parallel group, crossover, factorial, single group), allocation ratio, and framework (eg, superiority, equivalence, noninferiority, exploratory)	4

Methods: Participants, interventions, and outcomes

Study setting	9	Description of study settings (eg, community clinic, academic hospital) and list of countries where data will be collected. Reference to where list of study sites can be obtained	4
Eligibility criteria	10	Inclusion and exclusion criteria for participants. If applicable, eligibility criteria for study centres and individuals who will perform the interventions (eg, surgeons, psychotherapists)	5
Interventions	11a	Interventions for each group with sufficient detail to allow replication, including how and when they will be administered	5
	11b	Criteria for discontinuing or modifying allocated interventions for a given trial participant (eg, drug dose change in response to harms, participant request, or improving/worsening disease)	5
	11c	Strategies to improve adherence to intervention protocols, and any procedures for monitoring adherence (eg, drug tablet return, laboratory tests)	n/a
	11d	Relevant concomitant care and interventions that are permitted or prohibited during the trial	n/a
Outcomes	12	Primary, secondary, and other outcomes, including the specific measurement variable (eg, systolic blood pressure), analysis metric (eg, change from baseline, final value, time to event), method of aggregation (eg, median, proportion), and time point for each outcome. Explanation of the clinical relevance of chosen efficacy and harm outcomes is strongly recommended	6
Participant timeline	13	Time schedule of enrolment, interventions (including any run-ins and washouts), assessments, and visits for participants. A schematic diagram is highly recommended (see Figure)	6
Sample size	14	Estimated number of participants needed to achieve study objectives and how it was determined, including clinical and statistical assumptions supporting any sample size calculations	7
Recruitment	15	Strategies for achieving adequate participant enrolment to reach target sample size	7

Methods: Assignment of interventions (for controlled trials)

Allocation:

Sequence generation	16a	Method of generating the allocation sequence (eg, computer-generated random numbers), and list of any factors for stratification. To reduce predictability of a random sequence, details of any planned restriction (eg, blocking) should be provided in a separate document that is unavailable to those who enrol participants or assign interventions	n/a
Allocation concealment mechanism	16b	Mechanism of implementing the allocation sequence (eg, central telephone; sequentially numbered, opaque, sealed envelopes), describing any steps to conceal the sequence until interventions are assigned	n/a
Implementation	16c	Who will generate the allocation sequence, who will enrol participants, and who will assign participants to interventions	n/a
Blinding (masking)	17a	Who will be blinded after assignment to interventions (eg, trial participants, care providers, outcome assessors, data analysts), and how	n/a
	17b	If blinded, circumstances under which unblinding is permissible, and procedure for revealing a participant's allocated intervention during the trial	n/a
**Methods: Data collection, management, and analysis**			
Data collection methods	18a	Plans for assessment and collection of outcome, baseline, and other trial data, including any related processes to promote data quality (eg, duplicate measurements, training of assessors) and a description of study instruments (eg, questionnaires, laboratory tests) along with their reliability and validity, if known. Reference to where data collection forms can be found, if not in the protocol	7
	18b	Plans to promote participant retention and complete follow-up, including list of any outcome data to be collected for participants who discontinue or deviate from intervention protocols	n/a
Data management	19	Plans for data entry, coding, security, and storage, including any related processes to promote data quality (eg, double data entry; range checks for data values). Reference to where details of data management procedures can be found, if not in the protocol	n/a
Statistical methods	20a	Statistical methods for analysing primary and secondary outcomes. Reference to where other details of the statistical analysis plan can be found, if not in the protocol	7
	20b	Methods for any additional analyses (eg, subgroup and adjusted analyses)	n/a

	20c	Definition of analysis population relating to protocol non-adherence (eg, as randomised analysis), and any statistical methods to handle missing data (eg, multiple imputation)	n/a
**Methods: Monitoring**			
Data monitoring	21a	Composition of data monitoring committee (DMC); summary of its role and reporting structure; statement of whether it is independent from the sponsor and competing interests; and reference to where further details about its charter can be found, if not in the protocol. Alternatively, an explanation of why a DMC is not needed	n/a
	21b	Description of any interim analyses and stopping guidelines, including who will have access to these interim results and make the final decision to terminate the trial	n/a
Harms	22	Plans for collecting, assessing, reporting, and managing solicited and spontaneously reported adverse events and other unintended effects of trial interventions or trial conduct	7
Auditing	23	Frequency and procedures for auditing trial conduct, if any, and whether the process will be independent from investigators and the sponsor	n/a
**Ethics and dissemination**			
Research ethics approval	24	Plans for seeking research ethics committee/institutional review board (REC/IRB) approval	8
Protocol amendments	25	Plans for communicating important protocol modifications (eg, changes to eligibility criteria, outcomes, analyses) to relevant parties (eg, investigators, REC/IRBs, trial participants, trial registries, journals, regulators)	n/a
Consent or assent	26a	Who will obtain informed consent or assent from potential trial participants or authorised surrogates, and how (see Item 32)	5
	26b	Additional consent provisions for collection and use of participant data and biological specimens in ancillary studies, if applicable	7
Confidentiality	27	How personal information about potential and enrolled participants will be collected, shared, and maintained in order to protect confidentiality before, during, and after the trial	n/a
Declaration of interests	28	Financial and other competing interests for principal investigators for the overall trial and each study site	11
Access to data	29	Statement of who will have access to the final trial dataset, and disclosure of contractual agreements that limit such access for investigators	9

review only

Ancillary and post-trial care	30	Provisions, if any, for ancillary and post-trial care, and for compensation to those who suffer harm from trial participation	n/a
Dissemination policy	31a	Plans for investigators and sponsor to communicate trial results to participants, healthcare professionals, the public, and other relevant groups (eg, via publication, reporting in results databases, or other data sharing arrangements), including any publication restrictions	8
	31b	Authorship eligibility guidelines and any intended use of professional writers	n/a
	31c	Plans, if any, for granting public access to the full protocol, participant-level dataset, and statistical code	n/a
**Appendices**			
Informed consent materials	32	Model consent form and other related documentation given to participants and authorised surrogates	n/a
Biological specimens	33	Plans for collection, laboratory evaluation, and storage of biological specimens for genetic or molecular analysis in the current trial and for future use in ancillary studies, if applicable	n/a

*It is strongly recommended that this checklist be read in conjunction with the SPIRIT 2013 Explanation & Elaboration for important clarification on the items. Amendments to the protocol should be tracked and dated. The SPIRIT checklist is copyrighted by the SPIRIT Group under the Creative Commons "Attribution-NonCommercial-NoDerivs 3.0 Unported" license.

For peer review only

CONSORT 2010 checklist of information to include when reporting a pilot or feasibility trial*

Section/Topic	Item No	Checklist item	Reported on page No
**Title and abstract**			
	1a	Identification as a pilot or feasibility randomised trial in the title	1
	1b	Structured summary of pilot trial design, methods, results, and conclusions (for specific guidance see CONSORT abstract extension for pilot trials)	1
**Introduction**			
Background and objectives	2a	Scientific background and explanation of rationale for future definitive trial, and reasons for randomised pilot trial	3
	2b	Specific objectives or research questions for pilot trial	4
**Methods**			
Trial design	3a	Description of pilot trial design (such as parallel, factorial) including allocation ratio	4
	3b	Important changes to methods after pilot trial commencement (such as eligibility criteria), with reasons	n/a
Participants	4a	Eligibility criteria for participants	5
	4b	Settings and locations where the data were collected	4
	4c	How participants were identified and consented	5
Interventions	5	The interventions for each group with sufficient details to allow replication, including how and when they were actually administered	5
Outcomes	6a	Completely defined prespecified assessments or measurements to address each pilot trial objective specified in 2b, including how and when they were assessed	6
	6b	Any changes to pilot trial assessments or measurements after the pilot trial commenced, with reasons	n/a
	6c	If applicable, prespecified criteria used to judge whether, or how, to proceed with future definitive trial	4,7
Sample size	7a	Rationale for numbers in the pilot trial	7
	7b	When applicable, explanation of any interim analyses and stopping guidelines	n/a
Randomisation:			
Sequence generation	8a	Method used to generate the random allocation sequence	n/a
	8b	Type of randomisation(s); details of any restriction (such as blocking and block size)	n/a
Allocation concealment mechanism	9	Mechanism used to implement the random allocation sequence (such as sequentially numbered containers), describing any steps taken to conceal the sequence until interventions were assigned	n/a

For peer review only

Implementation	10	Who generated the random allocation sequence, who enrolled participants, and who assigned participants to interventions	n/a
Blinding	11a	If done, who was blinded after assignment to interventions (for example, participants, care providers, those assessing outcomes) and how	n/a
	11b	If relevant, description of the similarity of interventions	n/a
Statistical methods	12	Methods used to address each pilot trial objective whether qualitative or quantitative	7
**Results**			
Participant flow (a diagram is strongly recommended)	13a	For each group, the numbers of participants who were approached and/or assessed for eligibility, randomly assigned, received intended treatment, and were assessed for each objective	n/a
	13b	For each group, losses and exclusions after randomisation, together with reasons	n/a
Recruitment	14a	Dates defining the periods of recruitment and follow-up	n/a
	14b	Why the pilot trial ended or was stopped	n/a
Baseline data	15	A table showing baseline demographic and clinical characteristics for each group	n/a
Numbers analysed	16	For each objective, number of participants (denominator) included in each analysis. If relevant, these numbers should be by randomised group	n/a
Outcomes and estimation	17	For each objective, results including expressions of uncertainty (such as 95% confidence interval) for any estimates. If relevant, these results should be by randomised group	n/a
Ancillary analyses	18	Results of any other analyses performed that could be used to inform the future definitive trial	n/a
Harms	19	All important harms or unintended effects in each group (for specific guidance see CONSORT for harms)	n/a
	19a	If relevant, other important unintended consequences	n/a
**Discussion**			
Limitations	20	Pilot trial limitations, addressing sources of potential bias and remaining uncertainty about feasibility	n/a
Generalisability	21	Generalisability (applicability) of pilot trial methods and findings to future definitive trial and other studies	n/a
Interpretation	22	Interpretation consistent with pilot trial objectives and findings, balancing potential benefits and harms, and considering other relevant evidence	n/a
	22a	Implications for progression from pilot to future definitive trial, including any proposed amendments	n/a
**Other information**			
Registration	23	Registration number for pilot trial and name of trial registry	2
Protocol	24	Where the pilot trial protocol can be accessed, if available	n/a
Funding	25	Sources of funding and other support (such as supply of drugs), role of funders	9
	26	Ethical approval or approval by research review committee, confirmed with reference number	8

For peer review only

Citation: Eldridge SM, Chan CL, Campbell MJ, Bond CM, Hopewell S, Thabane L, et al. CONSORT 2010 statement: extension to randomised pilot and feasibility trials. BMJ. 2016;355.
*We strongly recommend reading this statement in conjunction with the CONSORT 2010, extension to randomised pilot and feasibility trials, Explanation and Elaboration for important clarifications on all the items. If relevant, we also recommend reading CONSORT extensions for cluster randomised trials, non-inferiority and equivalence trials, non-pharmacological treatments, herbal interventions, and pragmatic trials. Additional extensions are forthcoming: for those and for up to date references relevant to this checklist, see www.consort-statement.org.

For peer review only
