# Supplementary material for: Is it feasible to conduct a randomised controlled trial of pretransplant exercise (prehabilitation) for patients with multiple myeloma awaiting autologous haematopoietic stem cell transplantation? Protocol for the PREeMPT study
Source: BMJ Open. 2018 Mar 9;8(3):e021333. doi: 10.1136/bmjopen-2017-021333 (PMC5855173; doi:10.1136/bmjopen-2017-021333)
Supplement: Reviewer comments [file bmjopen-2017-021333.reviewer_comments.pdf]

PEER REVIEW HISTORY

BMJ Open publishes all reviews undertaken for accepted manuscripts. Reviewers are asked to complete a checklist review form (**http://bmjopen.bmj.com/site/about/resources/checklist.pdf**) and are provided with free text boxes to elaborate on their assessment. These free text comments are reproduced below.

ARTICLE DETAILS

**TITLE (PROVISIONAL)**	Is it feasible to conduct a randomised controlled trial of pre-transplant exercise (pre-habilitation) for multiple myeloma patients awaiting autologous haematopoietic stem cell transplantation? Protocol for the PREeMPT study.
**AUTHORS**	Keen, Carol<br>Skilbeck, Julie<br>Ross, Helen<br>Smith, Lauren<br>Collins, Karen<br>Dixey, Joanne<br>Walters, Stephen<br>Greenfield, Diana<br>Snowden, John<br>Mawson, Susan

This article was not externally reviewed at BMJ Open. Protocols that have been independently assessed prior to submission to BMJ Open are usually fast-tracked to publication on the grounds that further substantial changes will not be possible. This independent assessment will usually be external, independent review for both a substantial grant award from a non-commercial or government funder and ethics approval.
